# Site‐Selective Modification of Peptides and Proteins via Interception of Free‐Radical‐Mediated Dechalcogenation

**DOI:** 10.1002/anie.202006260

**Published:** 2020-10-19

**Authors:** Rhys C. Griffiths, Frances R. Smith, Jed E. Long, Huw E. L. Williams, Robert Layfield, Nicholas J. Mitchell

**Affiliations:** ^1^ School of Chemistry University of Nottingham University Park Nottingham NG7 2RD UK; ^2^ Biodiscovery Institute University of Nottingham University Park Nottingham NG7 2RD UK; ^3^ School of Life Sciences University of Nottingham University Park Nottingham NG7 2UH UK

**Keywords:** bioconjugation, deselenization, desulfurization, peptides, radical reactions

## Abstract

The development of site‐selective chemistry targeting the canonical amino acids enables the controlled installation of desired functionalities into native peptides and proteins. Such techniques facilitate the development of polypeptide conjugates to advance therapeutics, diagnostics, and fundamental science. We report a versatile and selective method to functionalize peptides and proteins through free‐radical‐mediated dechalcogenation. By exploiting phosphine‐induced homolysis of the C−Se and C−S bonds of selenocysteine and cysteine, respectively, we demonstrate the site‐selective installation of groups appended to a persistent radical trap. The reaction is rapid, operationally simple, and chemoselective. The resulting aminooxy linker is stable under a variety of conditions and selectively cleavable in the presence of a low‐oxidation‐state transition metal. We have explored the full scope of this reaction using complex peptide systems and a recombinantly expressed protein.

## Graphical abstract

Functionalization of peptides and proteins through the trapping of peptide radical species is described. This novel and versatile strategy exploits the process of desulfurization and deselenization of cysteine and selenocystine residues to enable the site‐selective installation of a broad range of groups appended to a persistent radical trap.

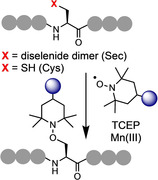

## Introduction

The diverse array of chemical functionality displayed by the 20 canonical amino acids presents both a challenge and an opportunity for the site‐selective functionalization of peptides and proteins. A broad range of reactions have been reported to modify the majority of the proteinogenic residues,[^1^, ^2^, ^3^, ^4^] providing tools to enable the study and manipulation of biological systems, and the preparation of therapeutic and diagnostic agents. To be widely applicable to peptide/protein bioconjugation such reactions must be chemoselective, high yielding, rapid, and operationally simple. The number of modified isoforms produced by a technique will be dictated by both the chemoselectivity of the chemistry and the relative abundance of the target amino acid. Reactions that select the more abundant amino acids, such as lysine (Lys), which accounts for approximately 6 % of residues across the human proteome, are liable to produce a mixture of isoforms which can be challenging to purify.[^2^, ^5^] Conversely, the amino acid cysteine (Cys) constitutes just 2 % of our proteome. Due to this limited presence, coupled with the enhanced nucleophilicity of the thiol sidechain and ease of incorporation of non‐native Cys via site‐directed mutagenesis, reactions that target this residue have been widely adopted throughout industry and academia.[^6^, ^7^]

**Figure 1:**
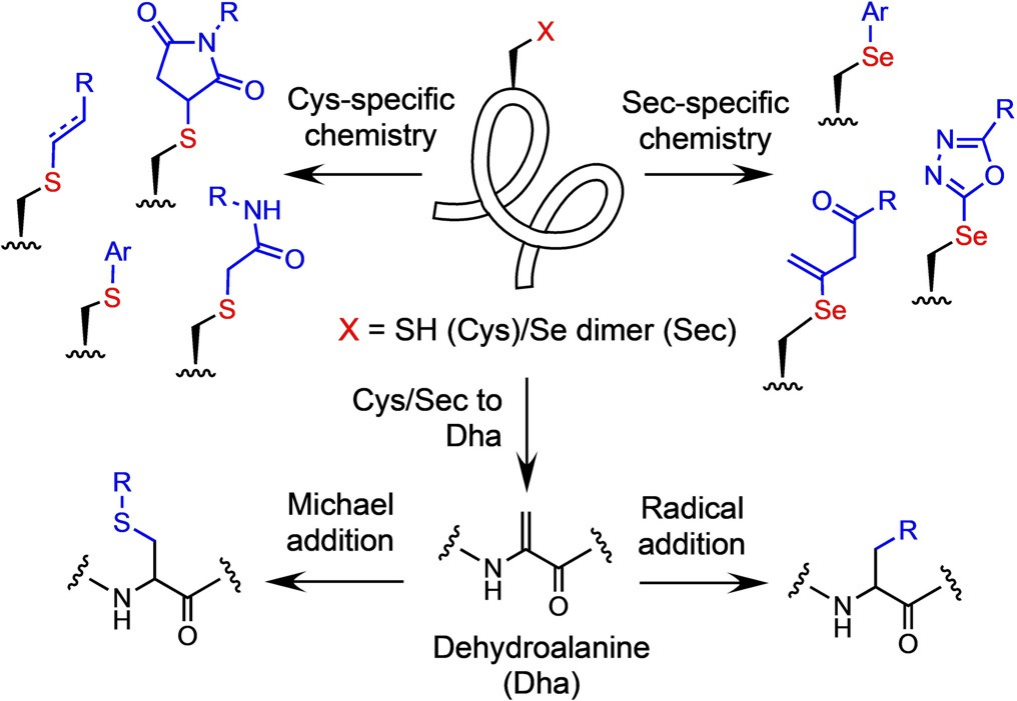
Current approaches to selective peptide and protein modification at selenocysteine (Sec) and cysteine (Cys) residues.

The “standard” Cys‐specific conjugation methods employ electrophilic moieties such as α‐halocarbonyl[^7^, ^8^] and maleimide[^7^, ^9^] groups (Figure 1). However, such components present chemoselectivity and stability challenges, respectively. More recently, bromomaleimides,^[10]^ perfluoroaromatics,^[11]^ phosphonamidates,^[12]^ allenamides,^[13]^ vinyl/alkynyl,^[14]^ and acrylate groups^[15]^ have been investigated to selectively label Cys residues. Methods beyond simple addition chemistry, such as thiol‐ene/yne click reactions,^[16]^ metal‐catalyzed^[17]^ and metal‐free arylation,^[18]^ and oxidative elimination to dehydroalanine (Dha) with subsequent Michael^[19]^ or radical addition,[^20^, ^21^, ^22^] have also been explored. Despite the broad range of chemistry developed to target this residue, challenges persist regarding reaction efficiency, chemoselectivity, tolerance, operational simplicity, and conjugate stability. Thus, novel methods are in high demand.

Selenocysteine (Sec) represents an alternative conjugation handle to Cys. Known as the 21^st^ proteinogenic amino acid, biological expression of Sec is rare; only 25 selenoproteins have been identified within the human proteome.^[23]^ Incorporation of this amino acid is facilitated by a dedicated suite of proteins and a Sec Insertion Sequence Element (SECIS), a stem‐loop RNA structure that repurposes the opal codon (UGA) for Sec installation. Thus, selenoproteins can be prepared using modified expression techniques, albeit in lower yield than can be achieved with standard recombinant expression.[^24^, ^25^, ^26^, ^27^] The selenol moiety of the Sec residue exhibits enhanced nucleophilicity over the thiol group of Cys due to the increased polarizability of the selenium atom.^[28]^ With a low redox potential (−381 mV), Sec is readily oxidized, thus its predominant role in nature is as a scavenger for damaging oxidizing agents. Strategies to site‐selectively functionalize Sec‐containing peptides and proteins include metal‐catalyzed and metal‐free arylation,^[29]^ heteroarylation, alkylation and allenamidation.^[30]^

Beyond applications in bioconjugation, both Cys and Sec residues have been utilized to facilitate the chemical ligation of peptide sequences. The technique of native chemical ligation (NCL)[^31^, ^32^] enables peptides bearing an N‐terminal Cys residue to be chemoselectively linked to sequences containing a C‐terminal thioester via a native amide bond. Further developments to this powerful method^[33]^ include the use of Sec‐containing peptides[^34^, ^35^, ^36^] and selenoester peptides to accelerate the ligation reaction.^[37]^ The development of diselenide‐selenoester ligation (DSL) employs both selenium‐containing fragments to enable rapid, additive‐free peptide ligation.[^38^, ^39^, ^40^, ^41^, ^42^, ^43^] Protocols that facilitate the post‐ligation conversion of internal Cys and Sec residues to alanine (Ala), via desulfurization and deselenization, respectively,[^44^, ^45^] permit peptide ligation at sites that contain this more abundant residue. Further developments in this field include the utilization of non‐proteinogenic thiolated and selenolated residues, which, when coupled with dechalcogenation,[^46^, ^47^] grant access to a broad range of ligation junctions, dramatically enhancing the scope of the technique.

**Figure 2:**
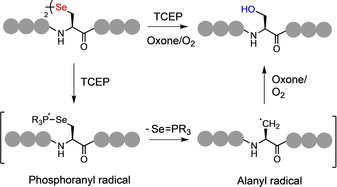
Proposed mechanism of free‐radical‐mediated deselenization with addition of oxone or O_2_ to afford serine (Ser) at the site of deselenization.

**Figure 3:**
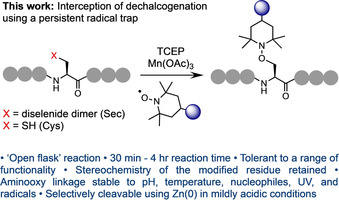
Trapping of the alanyl radical produced from free‐radical‐mediated deselenization/desulfurization at Sec/Cys residues using a TEMPO‐based persistent radical.

It is proposed that the desulfurization and deselenization pathways (mediated by a phosphine) proceed, in both cases, via a free‐radical process (Figure 2). Desulfurization of Cys‐containing peptides requires the initial formation of a thiyl radical (usually via addition of the initiator, VA‐044) which adds into the phosphine (TCEP—tris(2‐carboxyethyl)phosphine) to form a phosphoranyl radical. Sec undergoes this reaction spontaneously under ambient conditions. Subsequent homolysis of the C−S/C−Se bond of the phosphoranyl sulphide/selenide produces an alanyl radical (with release of the phosphine sulfide/selenide). This intermediate radical species will then abstract an H‐atom from a thiol additive to yield an Ala residue at this position. In the case of deselenization, the alanyl radical can be trapped by a peroxide salt (Oxone)[^48^, ^49^] or O_2_ ^[50]^ to install a hydroxyl group at the ligation junction, and thus afford the amino acid serine (Ser; Figure 2). Successful trapping of the alanyl radical suggests potential scope for the development of a novel bioconjugation strategy. Previous research has described the use of thiophosphonium salts to induce the conversion of disulfides to thioethers^[51]^ and also as a method to deuterate Cys‐containing peptides via desulfurization.^[52]^ However, to our knowledge, free‐radical‐mediated dechalcogenation has not been explored as a method to install groups of interest into peptides and proteins. Interception of this pathway, using a suitable functionalized trapping agent, would potentially be a powerful addition to the synthetic methodology within this field (Figure 3).

The persistent radical, TEMPO (2,2,6,6‐tetramethyl‐1‐piperidine‐1‐oxyl), would be an ideal trapping reagent in this context due to the stability of the sterically shielded nitroxyl radical. This reaction is likely to be chemoselective in the presence of native proteinogenic chemical functionality and rapid, assuming it can outcompete H‐atom abstraction. Crucially, the generation and trapping of a radical at the β‐carbon of Sec or Cys, via the described pathway, would maintain the integrity of the α‐stereocenter of the target residue. By applying this approach, utilizing TEMPO derivatives that carry a broad range of desirable moieties, we demonstrate the first example of site‐selective functionalization via trapping of free‐radical‐mediated dechalcogenation, representing an entirely novel method of peptide and protein modification (Figure 3).

## Results and Discussion

For our initial investigations into this concept, we synthesized the small Sec‐containing model peptide **1 a** (H‐UAF‐OMe) and subjected it to standard deselenization conditions on an analytical scale: 125 mM TCEP (50 molar eq.), 62.5 mM TEMPO (25 equiv.) at 2.5 mM *wrt* the selenol monomer (1.25 mM *wrt* to the diselenide dimer), in neutral buffer containing a high concentration of a chaotropic salt (6 M Gdn⋅HCl, 0.1 M Na_2_HPO_4_, pH 6.5).

**Figure 4:**
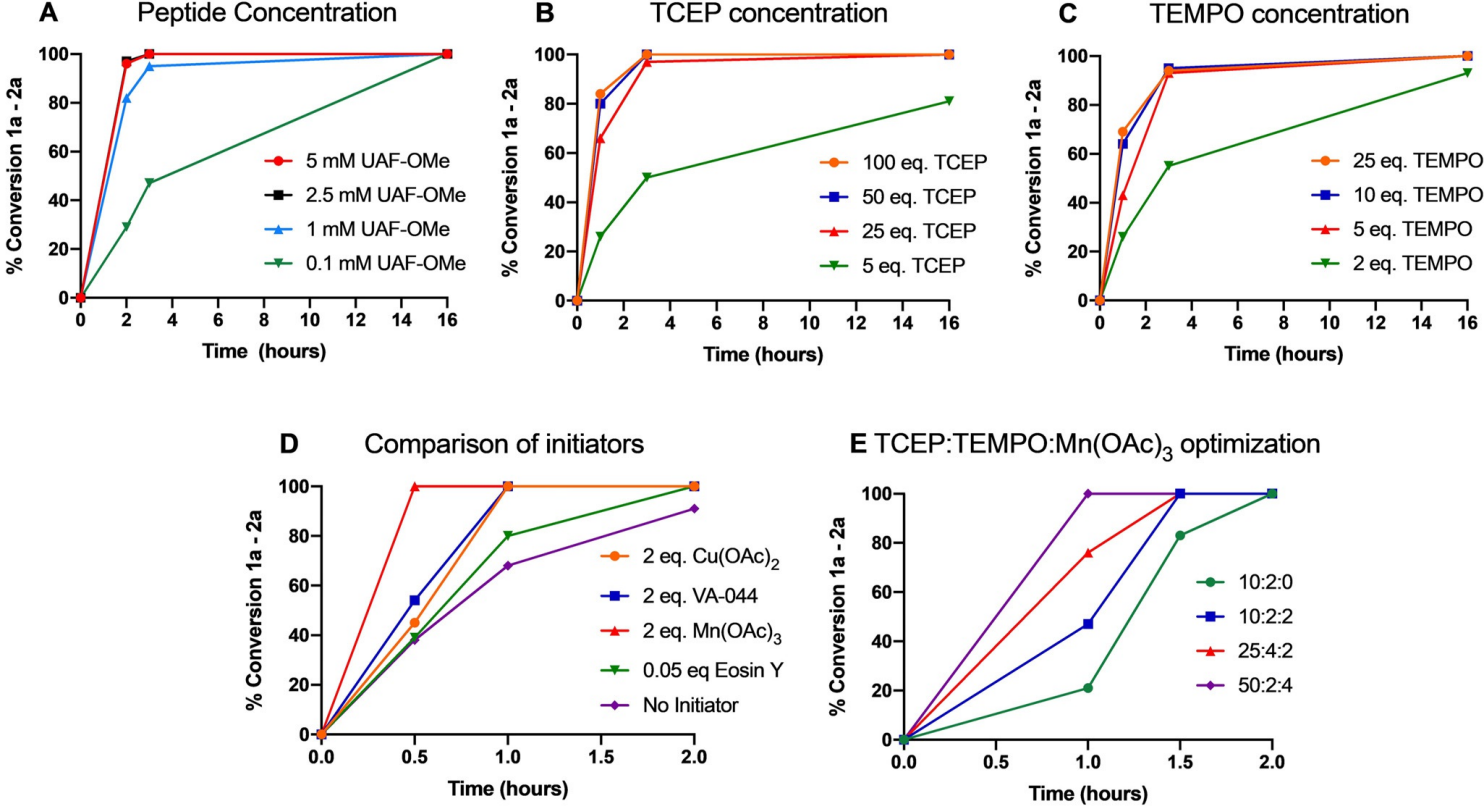
Data plots illustrating the effect of several variables on reaction rate (**1 a**–**2 a**); plots generated from integration of the desired product peak (**2 a**) relative to the starting peptide (**1 a**) by analytical HPLC; see SI Figures S7–S16 for further details regarding reaction conditions.

A co‐solvent (20 % methanol) was included to facilitate dissolution of the reagents and the reaction run at 37 °C. Full conversion to **2 b** was complete within 6 hours (Table 1, entry 1; reaction monitored by analytical HPLC). Gratifyingly, the undesired Ala by‐product, produced via quenching of the alanyl radical by H‐atom abstraction from a suitable donor, was not observed. The persistent TEMPO radical is able to outcompete this process to yield quantitative conversion to the desired conjugate. To enhance the rate of conjugation several variables were then explored, including; temperature, pH, stoichiometry of reagents, co‐solvent, and the use of various additives (see Table 1, Figure 4 and SI for details). The reaction was observed to proceed at a slightly slower rate if the excess of TCEP was dropped to 25 equiv. and failed to reach completion over 16 hrs with 5 equiv. (Figure 4 B). Doping the solution with TCEP and TEMPO twice over the first hour pushed the reaction to completion within 3 hours (entry 2). Doping also enabled us to lower the excess of TEMPO to 5 equiv. without dramatically decreasing the rate of reaction (entry 3). Running the reaction without a co‐solvent did not affect the rate (entry 5). Raising the temperature to 50 °C further accelerated the reaction eliminating the need for doping, allowing us to employ a more acceptable overall stoichiometry of TEMPO (entry 6). Doping under these conditions dropped the conversion rate to 2 hours (entry 7) and allowed us to again reduce the excess of TCEP and TEMPO, to 10 and 2 equiv. respectively (entry 8).

**Table 1:** Reaction exploration and optimization. 
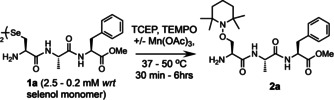

Entry	Peptide [mM]	*T* [°C]	% Co‐solvent^[a]^	TCEP [equiv]	TEMPO [equiv].	Doping^[b]^	Additive [equiv]	Full conversion **1 a**–**2 a** ^[c]^
1	2.5	37	20	50	25	0	–	6 h
2	2.5	37	20	50	25	2	–	3 h
3	2.5	37	20	50	5	2	–	4 h
4	2.5	37	20	25	5	2	–	4 h
5	2.5	37	0^[d]^	25	5	2	–	4 h
6	2.5	50	20	50	5	0	–	4 h
7	2.5	50	20	50	5	2	–	2 h
8	2.5	50	20	10	2	2	–	2 h
9	2.5	50	20	10	2	0	2 (Mn(OAc)_3_)	90 mins
**10^[e]^**	**2.5**	**50**	**20**	**50**	**2**	**0**	**4 (Mn(OAc)_3_)**	**60 mins**
11	2.5	50	20	50	5	2	2 (Mn(OAc)_3_)	30 mins
12	0.2	≈30	20	50	5	2	0.05 (Eosin Y)^[f]^	2 h

[a] 20 % methanol in ligation buffer (6 M Gdn⋅HCl, 0.1 M Na_2_HPO_4_, pH 7.0); degassing of the solution was not required. [b] Addition of TCEP and TEMPO at stated equiv at 15 and 45 mins; entry 11 doped at 5 and 15 mins. [c] Reaction reached 100 % conversion to **2 a** by the stated time, as determined by analytical HPLC. [d] 100 % ligation buffer (6 M Gdn⋅HCl, 0.1 M Na_2_HPO_4_, pH 7.0). [e] Conditions selected as the optimal balance between rate and stoichiometry. [f] Sample irradiated under blue LEDs, temp. measured as approximately 30 °C.

To further accelerate the rate of the reaction, we next explored the introduction of several additives to facilitate production of the alanyl radical. Cu(OAc)_2_, Mn(OAc)_3_, the radical initiator VA‐044, and the photosensitive dye, Eosin Y, were all evaluated (Figure 4 D). Each additive successfully enhanced the rate to afford complete conversion within 2 hrs. Mn(OAc)_3_ gave the fastest conversion; 4 equiv. of Mn(OAc)_3_ enabled us to decrease the excess of TEMPO to 2 equiv. with complete conversion observed in under 60 minutes, without doping (entry 10). However, doping with 2 equiv. of Mn(OAc)_3_ did allow us to push the reaction to completion within 30 minutes (entry 11). Additionally, the presence of an additive accelerated the reaction at lower concentrations of peptide **1 a**; a catalytic amount of the dye Eosin Y (5 mol %), with doping under blue LEDs, afforded full conversion at 0.2 mM over 2 hours (entry 12). The need for a balance between the rate of the reaction and a reasonable stoichiometry of TEMPO, which will carry the desired group for conjugation, led us to conclude that entry 10 represented the optimal set of conditions to take forward (referred to hereafter as protocol A, see Table S1). These conditions were repeated on a larger scale (8 mg of model peptide **1 a**) and the product purified via HPLC to afford the desired conjugate **2 a** in excellent yield (83 %, Table 2, entry 13).

**Table 2:** Yields of isolated peptide conjugates **2 a**, **8**–**12** from model peptide **1 a**. 
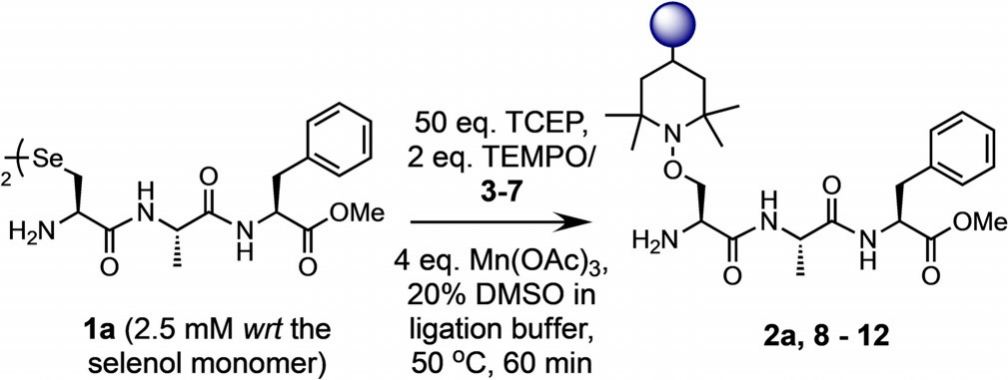

Entry	Product	Trap	Yield [%]
13	**2 a**	TEMPO	83
14	**8**	tetra‐EG (**3**)	72
15	**9**	propargyl (**4**)	80
16	**10**	fluorescein (**5**)	69
17	**11**	biotin (**6**)	78
18	**12**	gemcitabine (**7**)	67

**Figure 5:**
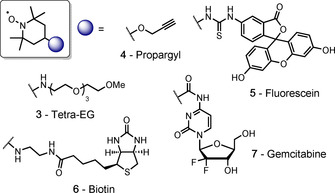
TEMPO‐based persistent radical traps **3**–**7**.

Based on the proposed mechanism of dechalcogenation under the described conditions, it is assumed that epimerization of the α‐carbon at the target residue should not occur. To confirm this, model peptide **1 b** was prepared carrying the d‐isomer of Sec (H‐d‐UAF‐OMe) and subjected to protocol A to yield conjugate **2 b**. Comparison of the ^1^H NMR spectra for epimers **2 a** and **2 b** clearly highlights that the signal shifts observed for **2 b** are not present in the NMR for **2 a** and vice versa (Figure S36). Therefore, it can be concluded that the integrity of the stereochemistry at the target residue remains intact throughout the conjugation process. Confident that we had fully optimized the method, a range of TEMPO traps, carrying potentially desirable functionality, were then prepared from either 4‐amino‐, carboxy‐, oxo‐, or hydroxy‐TEMPO (**3**–**7**, Figure 5), using mild, well‐established chemistry in high yield (see SI for details).

Tetraethylene glycol (tetra‐EG) polymer (**3**) was included to enable the attachment of a group to modulate the stability of a polypeptide; propargyl (**4**) and biotin (**6**) groups were explored to enable the isolation of tagged peptides/proteins; a fluorophore (fluorescein, **5**) was included for imaging, and a cytotoxic drug (gemcitabine, **7**) used to demonstrate protein‐drug conjugation (via a stable, non‐cleavable linker, thus relying on lysosomal degradation of the conjugate for drug release). Each trap was then conjugated to model peptide **1 a** using protocol A to demonstrate the tolerance of the technique. The reactions all proceeded to completion within 1 hour to afford the desired product in good–excellent isolated yields (67–83 %, products **8**–**12**, Table 2).

Although Sec provides a useful bioconjugation handle, especially when employing peptide ligation to access large peptides or small proteins, Cys is the target residue of choice for researchers wishing to modify recombinantly expressed proteins. Therefore, to test the potential of our reaction to label Cys‐containing peptides, the model H‐CAF‐OMe (**13**) was prepared and conjugation reactions with TEMPO trialed to afford conjugate **14** (Table S13). Although it was gratifying to confirm that the procedure could be applied to trap the alanyl radical produced via desulfurization of Cys, it was observed that the standard protocol A did not give an optimal rate for this model. It was also noted that use of Eosin Y as a catalytic initiator resulted in production of an unidentified impurity which co‐eluted with the product. Therefore, the stoichiometry of TCEP, TEMPO and Mn(OAc)_3_ was again explored to determine the ideal conditions. It was observed that 1 mM peptide, 50 equiv. TCEP, 2 equiv. TEMPO, 5 equiv. Mn(OAc)_3_, 50 °C in 20 % co‐solvent in ligation buffer resulted in full conversion to **14** over 2 hours (referred to hereafter as protocol B). The model peptide Ac‐CWHISKEY‐NH_2_ (**15**) was prepared to test the efficiency and chemoselectivity of the conjugation at Cys against more diverse proteinogenic chemical functionality. The isolated yields obtained on model **15** using protocol B with traps **3**–**7** were comparable to those of the Sec model (**16**–**20**, 71–82 % yield, Table 3). No by‐products were observed from side‐reactions with the nucleophilic (Lys) or aromatic (His) residues within this peptide. Quenching of the alanyl radical via H‐atom abstraction from the thiol groups of the Cys‐containing peptides in solution was a concern for this model. However, the Ala by‐product was not observed under the optimized conditions. Furthermore, to ensure that the reaction did not result in the oxidation of methionine (Met) residues, model peptide H‐UWIMKY‐NH_2_ (**21**) was synthesized and subjected to protocol A conditions to afford conjugate **22** in good yield (68 %, Figure S64) with no detectable oxidation of the Met residue.

**Table 3:** Isolated yields of peptide conjugates **16**–**20** from model peptide **15**. 
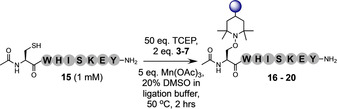

Entry	Product	Trap	Yield [%]
19	**16**	tetra‐EG (**3**)	80
20	**17**	propargyl (**4**)	82
21	**18**	fluorescein (**5**)	71
22	**19**	biotin (**6**)	73
23	**20**	gemcitabine (**7**)	80

**Figure 6:**
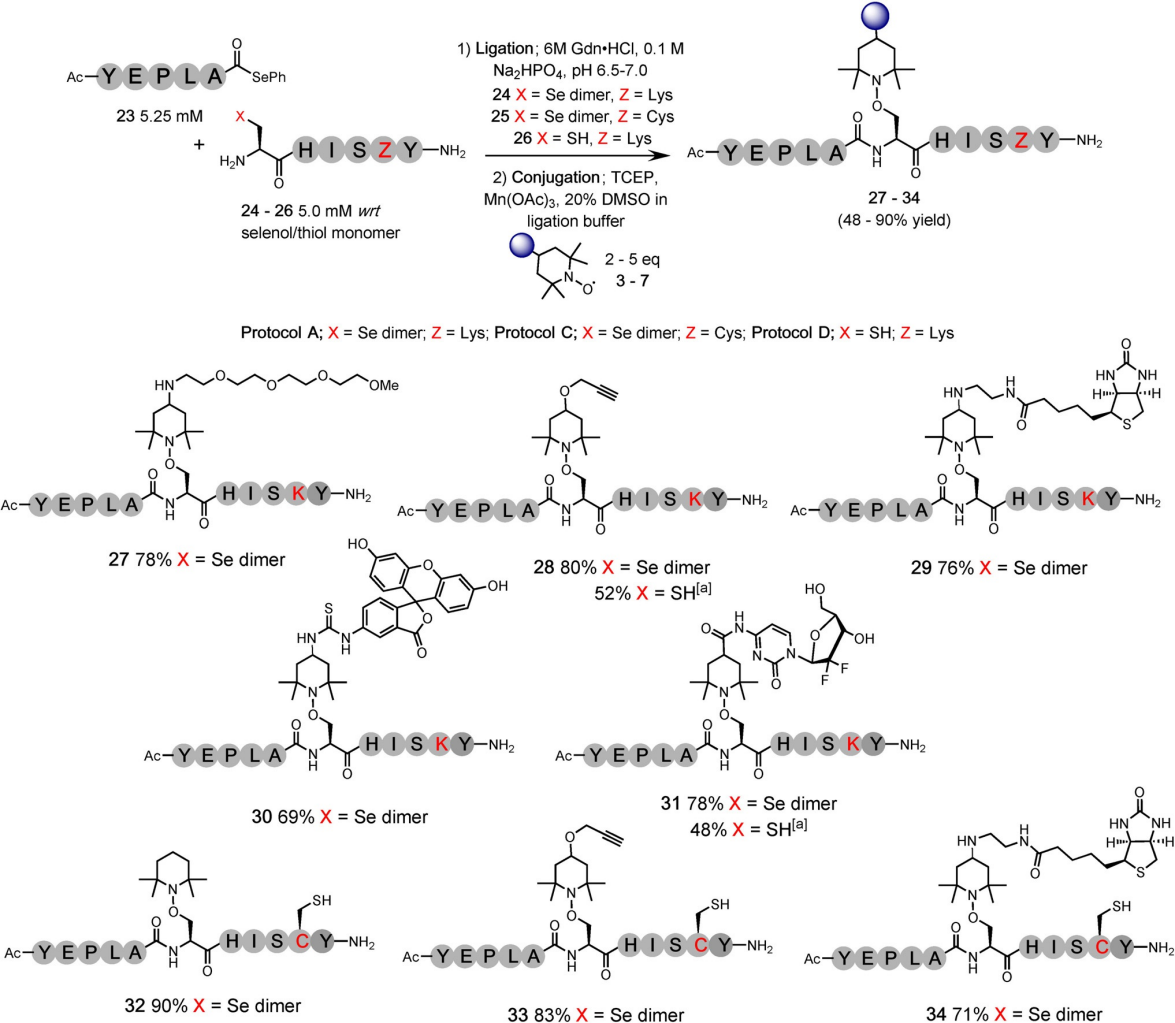
Ligation‐conjugation reactions; Additive‐free ligation conditions: 5 mM *wrt* selenol monomer (2.5 mM *wrt* diselenide dimer), and Cys‐peptide, 1.05 equiv. selenoester; Conjugation protocol A; 2.5 mM peptide (*wrt* selenol monomer), 50 equiv. TCEP, 2 equiv. TEMPO trap, 4 equiv. Mn(OAc)_3_, 50 °C, 1 hour; Protocol C; 2.5 mM peptide (*wrt* selenol monomer), 50 equiv. TCEP, 5 equiv. TEMPO trap, 37 °C, 4 hours; Protocol D; 2.5 mM peptide, 50 equiv. TCEP, 2 equiv. TEMPO trap, 10 equiv. Mn(OAc)_3_, 50 °C, 4 hours; [a] Overall yield over 2 steps.

The chemoselectivity demonstrated using these model peptides was encouraging, however, to ensure selectivity in larger, more complex peptides, and to demonstrate a successful one‐pot ligation‐functionalization protocol, the selenoester peptide Ac‐YEPLA‐SePh (**23**) and selenopeptide H‐UHISKY‐NH_2_ (**24**) were synthesized. Together, these models contain the majority of the chemical functionality present in larger protein systems, including; a nucleophilic amine (Lys), carboxylic acid (glutamic acid, Glu), aromatic groups (tyrosine, Tyr, and His), a primary alcohol (Ser), a primary amide (C‐terminus) and an aliphatic, sterically bulky group (leucine, Leu). Peptide ligation of **24** via DSL^[38]^ proceeded in buffer (6 M Gdn⋅HCl, 0.1 M Na_2_HPO_4_, pH 6.5) at 2.5 mM *wrt* to the diselenide dimer (5 mM *wrt* the selenol monomer) with a slight excess of the selenoester **23** (1.05 equiv.). Precipitation of diphenyl diselenide (DPDS) within 60 seconds indicated that the ligation had reached completion; this precipitate was extracted with hexane and a sample of the solution was removed for analysis. The crude ligation solution was partitioned into five equal volumes and the required equivalents of the reagents added from stock solutions; TCEP (0.625 M stock solution), TEMPO trap (**3**–**7**) (0.1 M stock solution) and Mn(OAc)_3_ (0.1 M stock solution). The reactions were diluted with buffer and DMSO (up to 20 % co‐solvent) to give the final concentrations as described by conjugation protocol A: 2.5 mM peptide *wrt* selenol monomer, 125 mM TCEP (50 equiv.), 5 mM TEMPO (2 equiv.), 10 mM Mn(OAc)_3_ (4 equiv.). The conjugations were heated at 50 °C for 1 hour; once the starting material was shown to be consumed by analytical HPLC, the crude reactions were purified by preparative HPLC to yield the desired products **27**–**31** in high yield (69–80 %) in one‐pot over two steps (Figure 6).

After demonstrating that Cys can be modified under the conjugation conditions containing Mn(OAc)_3_ as an additive (products **16**–**20**, Table 3), we were intrigued to explore the selective modification of Sec in the presence of Cys. To confirm that an internal Cys present in the sequence would not interfere with the modification of Sec, the model peptide H‐UHISCY‐NH_2_ (**25**) was synthesised and the one‐pot ligation‐conjugation attempted with selenoester **23** under conditions similar to protocol A, but with omission of Mn(OAc)_3_, run at 37 °C (50 equiv. TCEP, 5 equiv. TEMPO at a final peptide concentration of 2.5 mM—labelled as protocol C). The reaction was monitored via HPLC and observed to reach completion over 4 hours. The reaction mixture was purified to yield the desired product **32** in an excellent yield (90 %) over the two steps, with no apparent interference from the internal Cys residue (Figure 6). This ligation‐conjugation protocol was successfully repeated with these peptide models using the propargyl‐TEMPO (**4**) and biotin‐TEMPO (**6**) traps to yield products **33** and **34** in 83 % and 71 % respectively. No conjugation or desulfurization at the internal Cys residue was detected under these conditions. Crucially, the presence of the internal thiol (an excellent H‐atom source) within the peptide sequence did not quench the conjugation reaction at the Sec residue.

To demonstrate the application of our method to a ligation‐conjugation protocol using N‐terminal Cys residues with a selenoester peptide, we synthesized the model peptide H‐CHISKY‐NH_2_ (**26**) and submitted it to a ligation‐conjugation protocol with selenoester **23**. However, post‐ligation the internal Cys residue (the intended site of conjugation) reacts with any excess of the selenoester starting peptide to give an undesired pendent selenoester. This by‐product is not observed using an N‐terminal Sec residue for ligation as the low redox potential of this residue leads to rapid oxidation to the diselenide product. Treatment with hydroxylamine results in successful hydrolysis of the selenoester by‐product to yield the desired thiol, however, use of this additive prevents direct conjugation using the crude solution. We therefore switched to a two‐pot protocol for model peptide **26**. Ligation of the two peptides was followed by extraction of DPDS, addition of hydroxylamine, and purification of the ligated product to afford the desired peptide **35** in 77 % yield (Figure S85). When applying protocol B (optimized for model **15**; Ac‐CWHISKEY‐NH_2_) to the purified ligation product using propargyl‐TEMPO **4**, it was found that a slightly higher excess of Mn(OAc)_3_ (10 equiv.) at 2.5 mM *wrt* the starting peptide was required to successfully modify an internal Cys residue (labelled protocol D: Table S1). These conditions afforded the desired conjugate **28** in 81 % conversion (by HPLC) over 4 hours. The conjugation was repeated on an isolatable scale using peptide **35** with propargyl‐TEMPO **4** and gemcitabine‐TEMPO **7** to yield the desired conjugates (**28** and **31**) over the same time course in 67 % and 62 % isolated yield, respectively (52 % and 48 % over two steps).

Our investigation of one‐pot ligation‐conjugation using model peptide **25** (H‐UHISCY‐NH_2_) demonstrates that we can selectively modify a Sec residue in the presence of Cys. To further explore this selectivity, peptide **25** was subjected to the mild conjugation protocol C (i.e. no Mn(OAc)_3_) using the propargyl‐TEMPO trap **4** and purified to give mono‐modified peptide **36** in 68 % yield (Figure S90). This peptide was then submitted to the standard protocol A with the tetra‐EG trap **3** and the di‐modified peptide **37** was successfully isolated in 64 % yield (Figure S92), thus demonstrating that alternative moieties can be selectively installed at Sec and Cys residues by tuning the reaction conditions.

**Figure 7:**
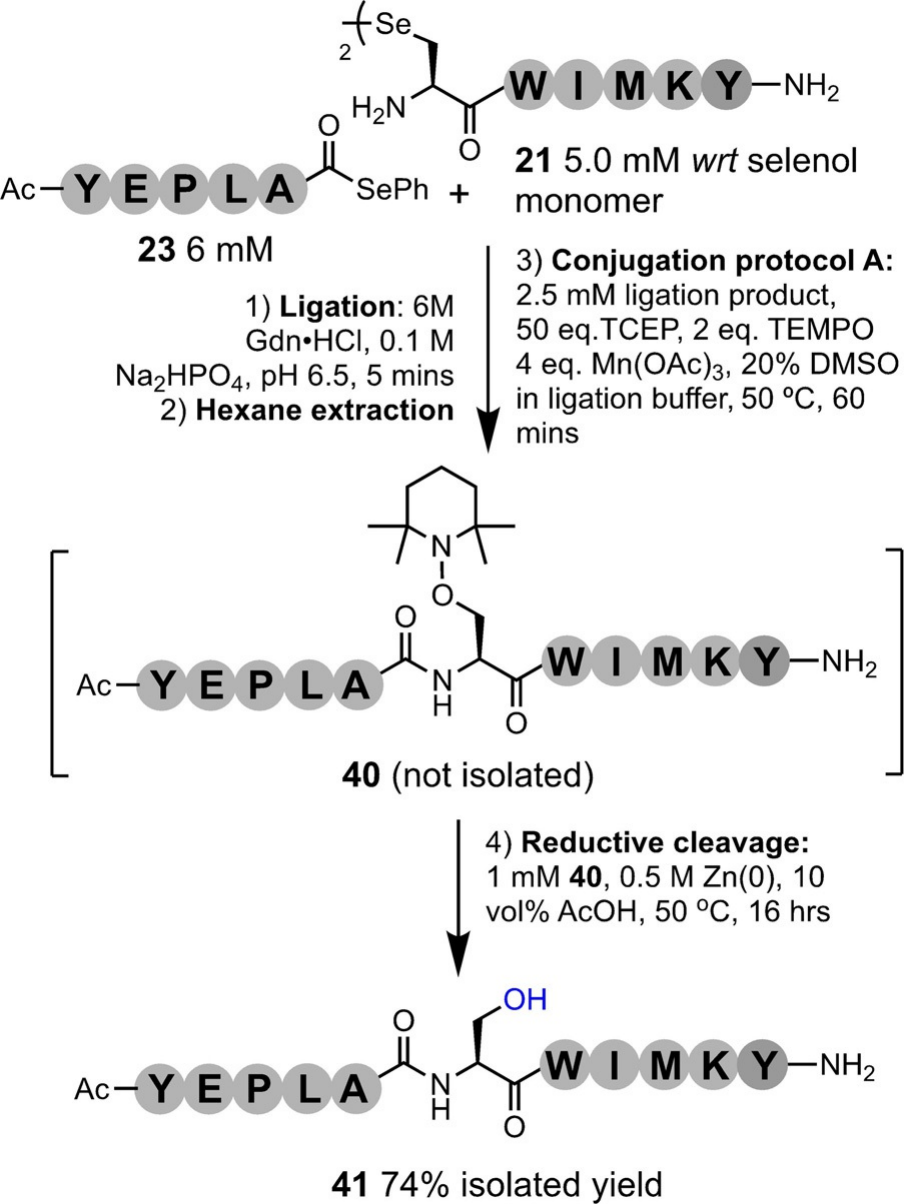
One‐pot ligation at Ser; DSL of model fragments **23** and **21**, followed by conjugation of TEMPO and reductive cleavage, affords the desired peptide bearing Ser at the ligation junction.

The stability of polypeptide conjugates is a vital aspect of any bioconjugation method. It is crucial, for instance, that protein‐drug conjugates do not prematurely release their cytotoxic cargo before reaching their target site. Conversely, applications within proteomics and protein profiling require selectively cleavable linkers to release isolated protein material for analysis. To investigate the stability of our aminooxy linker, TEMPO‐peptide conjugates **2 a** and **38**, prepared from starting peptides **1 a** (H‐UAF‐OMe) and **24** (H‐UHISKY‐NH_2_), were explored over 16 hrs at varying pH (2–9), elevated temperature, exposure to UV radiation, and in the presence of additives, including; VA‐044 (a radical initiator) and glutathione (see Tables S15 and S16 for details). The conjugate was found to be remarkably stable under the conditions explored. However, the aminooxy linker is labile in the presence of a low oxidation state transition metal in mildly acidic conditions. Introduction of 10 equiv. of Zn^0^ in 10 % acetic acid resulted in quantitative and clean degradation of the linker to leave a Ser residue at the site of conjugation. This protocol was applied to purified conjugate **28** with successful and exceptionally clean release of the propargyl trap to afford **39** in high yield (92 %) (Figure S103). To evaluate this method as a potential technique to achieve peptide ligation at a Ser residue, we investigated a one‐pot DSL‐TEMPO conjugation‐reductive cleavage protocol (Figure 7). Selenoester model **23** and diselenide model **21** were ligated via standard DSL conditions. Following hexane extraction of the precipitated DPDS, conjugation protocol A was carried out using TEMPO to afford conjugate **40**. To effect reductive cleavage of the aminooxy linkage of the conjugate in the crude mixture at 1 mM *wrt* **40**, 0.5 M Zn^0^ was required in 10 vol. % AcOH. The cleavage was complete after 16 h and the desired peptide (**41**), bearing a Ser residue at the ligation site, was isolated in good yield (74 %). This protocol is thus comparable to the previously reported Oxone‐TCEP method for programmable ligation at Ser (Figure S111).[^48^, ^49^]

**Figure 8:**
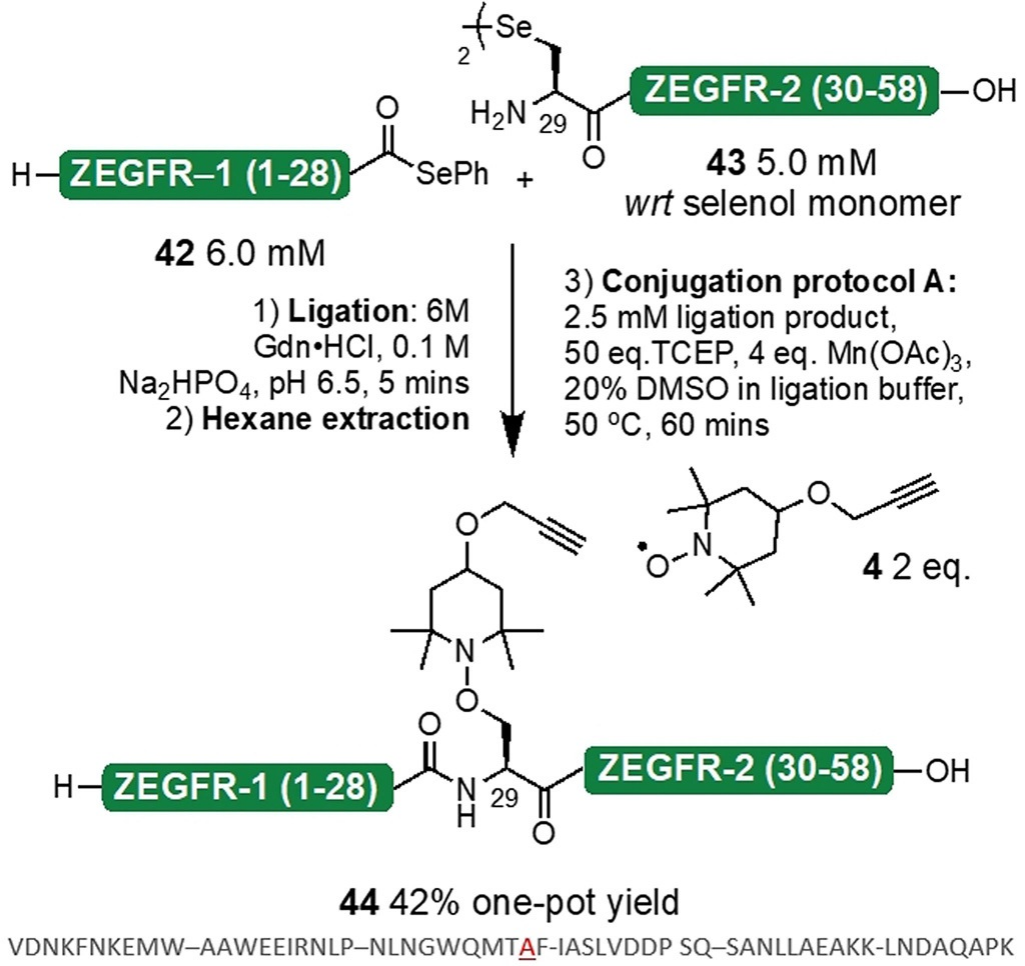
One‐pot ligation‐conjugation of Z_EGFR:1907_ affibody (native sequence shown): DSL of fragments **42** and **43** followed by conjugation of the propargyl **4** trap using protocol A afforded peptide conjugate **44**.

Finally, we applied our conjugation reaction to two larger, more complex polypeptide systems. To fully demonstrate the scope of the one‐pot ligation‐conjugation protocol, the affibody, Z_EGFR:1907_ (a 58 mer peptide derived from immunoglobin‐binding protein A)^[53]^ was synthesized as two fragments; selenoester **42** (amino acids 1–28) and diselenide **43** (amino acids 29–58). These two peptides were ligated under standard DSL conditions and, following hexane extraction of the precipitated DPDS, the propargyl‐TEMPO trap **4** was conjugated using protocol A. The desired conjugate **44** was isolated in a 42 % one‐pot yield (Figure 8).

**Figure 9:**
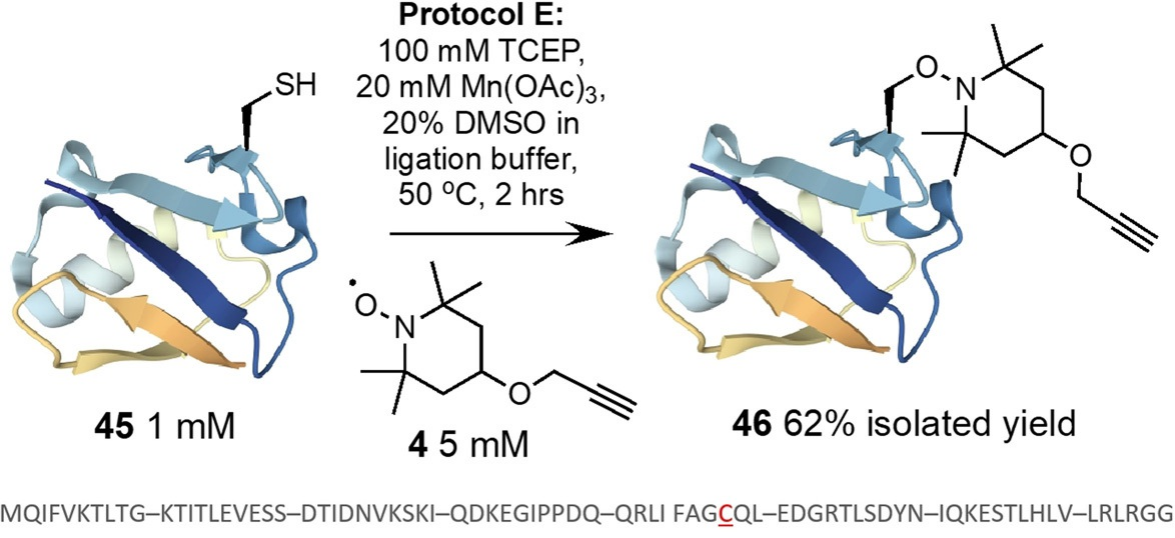
Conjugation of propargyl‐TEMPO trap **4** to recombinantly expressed Ub **45** (K48C; sequence shown)^[54]^ to yield conjugate **46** (Ub representation from PDB ID: 2L00).

To demonstrate our conjugation method on a biologically expressed protein sample, a K48C mutant of ubiquitin (**45**) was prepared via recombinant expression in *E. coli*.^[54]^ It was observed that modification of Cys within a protein required slightly higher equiv. than present in protocol D. Therefore, at 1 mM protein concentration, the propargyl‐TEMPO trap **4** was conjugated using 100 mM TCEP, 5 mM **4**, and 20 mM Mn(OAc)_3_ at 50 °C for 2 hours (protocol E). The product was purified by preparative HPLC and desired conjugate **46** isolated in 62 % yield (Figure 9). Purified Ub conjugate **46** and the K48C mutant **45** were re‐dissolved in ligation buffer and folded via dialysis into 25 mM Na_2_HPO_4_, 100 mM NaCl, pH 6.9. The samples were analysed via ^1^H NMR and assigned by comparison to the published data.^[55]^ Both conjugate **46** and K48C mutant **45** showed extended NH regions (6.5–9.5 ppm) and upshifted methyl groups indicating formation of the native tertiary structure (figs S121–S123). A comparison of the chemical shift values of the NH signals for **45** and **46** shows very little deviation across the majority of the sequence (Figure S120). As expected, those residues flanking modified position 48 do experience minor perturbations in chemical shift due to the installed moiety.

## Conclusion

In summary, we have developed a novel approach to site‐selective polypeptide modification via trapping of free‐radical‐mediated dechalcogenation. The reaction is operationally very simple (carried out on the bench in an open vessel, with no requirement to degas the solutions), and tolerant to a diverse range of moieties appended to a persistent radical trap. Crucially, we demonstrate that the stereochemistry of the α‐center of the modified Sec or Cys residue is retained during conjugation. Reagent stoichiometry has been optimized throughout (protocols A–E, see table S1) to demonstrate rapid labelling within 30–60 minutes for Sec residues and slightly longer for Cys (2–4 hrs). The reaction is effective down to a concentration of 100 μM (over 16 hrs, Figure 4 A), and selective for Sec in the presence of Cys. The aminooxy linker of the conjugate is stable under the conditions explored and can be controllably degraded in mild acid with the addition of Zn^0^ with exceptionally clean release. The method affords good–excellent yields on simple model systems and this efficiency translates well onto larger and more complex peptides and proteins carrying a wealth of chemical diversity. While conjugation to an internal Cys residue within a recombinantly expressed protein (ubiquitin) required a higher excess of the reagents relative to the peptide models, this increase does not translate into a significant quantity of material at the scale appropriate for protein chemistry. In addition, the functionalised TEMPO‐based traps can be synthesised on the gram‐scale in high yield, and the TCEP and Mn(OAc)_3_ reagents are relatively inexpensive. Beyond direct modification of polypeptides, we have applied our approach to advance the toolkit of ligation chemistry. We report a DSL‐conjugation protocol that enables the one‐pot chemical synthesis and modification of large peptides and small proteins. Furthermore, by exploiting the lability of the aminooxy linker to low oxidation state transition metals, we have developed a one‐pot ligation‐conjugation‐reductive cleavage technique that allows peptide ligation at Ser residues.

The tolerance, selectivity, and operational simplicity of this novel method, coupled with the versatility of the aminooxy linker, will enable researchers to employ the described protocols to prepare polypeptide‐small molecule conjugates suitable for a diverse range of applications.

## Conflict of interest

The authors declare no conflict of interest.

## Supporting information

As a service to our authors and readers, this journal provides supporting information supplied by the authors. Such materials are peer reviewed and may be re‐organized for online delivery, but are not copy‐edited or typeset. Technical support issues arising from supporting information (other than missing files) should be addressed to the authors.

Supplementary
