# Supplementary material for: Site‐Selective Modification of Peptides and Proteins via Interception of Free‐Radical‐Mediated Dechalcogenation
Source: Angew Chem Int Ed Engl. 2020 Oct 19;59(52):23659–67. doi: 10.1002/anie.202006260 (PMC7756370; doi:10.1002/anie.202006260)
Supplement: Supplementary file 1 — Supplementary [file ANIE-59-23659-s001.pdf]

Angewandte Chemie
Eine Zeitschrift der Gesellschaft Deutscher Chemiker

Supporting Information

Site-Selective Modification of Peptides and Proteins via Interception of Free-Radical-Mediated Dechalcogenation

*Rhys C. Griffiths, Frances R. Smith, Jed E. Long, Huw E. L. Williams, Robert Layfield, and Nicholas J. Mitchell**

anie_202006260_sm_miscellaneous_information.pdf

Contents

General methods S2

General conjugation protocols S7

Compound synthesis S10

Initial optimization of the conjugation protocol S25

Fmoc-SPPS and analytical data for model peptides S36

Conjugation reactions S49

Ligation-conjugation protocols S85

Conjugate stability studies S112

One-pot ligation-conjugation-reductive cleavage – ligation at serine S123

Synthesis and modification of ZEGFR:1907 via one-pot ligation-conjugation S128

Site-selective modification of ubiquitin K48C S134

References S140

Appendix S141

General methods

NMR samples were analysed on a Bruker AVIII 400 NMR system (^{1}H-NMR frequency 400 MHz; ^{13}C-NMR frequency 100 MHz). Folded Ub protein was analysed on a Bruker Avance III 800 MHz system. Chemical shifts are reported in parts per million (ppm) and are referenced to solvent residual signals: $CDCl_3$ (δ 7.26 [^{1}H]), DMSO (δ 2.50 [^{1}H]), MeOD (δ 3.31 [^{1}H]). ^{1}H NMR data is reported as chemical shift (δ), multiplicity (s = singlet, d = doublet, t = triplet, q = quartet, dd = doublet of doublets, ddd = doublet of doublet of doublets or combinations of these multiples; m = unassigned multiplet), relative integral, coupling constant (J Hz) and assignment where possible. To enable NMR analysis of the TEMPO traps, the persistent radical was first quenched with either direct addition of phenyl hydrazine to the NMR tube prior to analysis or injection into a RP-HPLC column using 0.1% TFA in water/acetonitrile, product collection and lyophilisation. This latter method proved useful for quenching the TEMPO radical without masking the aromatic region of the NMR with phenyl hydrazine peaks.

EPR spectra were recorded on a Bruker EMX spectrometer fitted with an X-band microwave bridge and running Xenon software. Samples were recorded as fluid solutions at ambient temperature using a microwave power of ca. 20 mW and a microwave frequency of 100 kHz. Data are reported at a modulation amplitude of 0.1 G (except FITS where the modulation amplitude was 1 G). Spectra were simulated using WINEPR SimFonia (Shareware version 1.25, Brüker Analytische Messtechnik GmbH).

High-resolution mass spectra were recorded on a Bruker MicroTOF Focus II MS (ESI) operating in positive or negative ionisation mode.

Analytical HPLC was performed on a Thermo Ultimate 3000 uHPLC system equipped with PDA eλ detector (λ = 210 – 400 nm). Peptides were analyzed using a Waters Sunfire 5 µm, 2.1 x 150 mm column (C-18) at a flow rate of 0.6 mL min^{-1}. The mobile phase composed of 0.1% trifluoroacetic acid in H_2O

(Solvent A) and 0.1% trifluoroacetic acid in acetonitrile (Solvent B). The analysis of the chromatograms was conducted using Chromeleon 7 software.

Preparative reverse-phase HPLC was performed using a Waters 1525 binary pump HPLC equipped with a dual wavelength UV detector set to 210 nm and 280 nm. Peptides were purified on a Waters Sunfire 5 µm (C-18) preparative column with 5-µm particle size, 19 x 150 mm, operating at a flow rate of 6 mL min^{-1} using a mobile phase of 0.1% trifluoroacetic acid in water (Solvent A) and 0.1% trifluoroacetic acid in acetonitrile (Solvent B) using the gradient specified in the experimental section. Semi-preparative reverse-phase HPLC was performed using the same HPLC and solvent system. The column used was a Waters Sunfire 5 µm (C-18) preparative column, 10 x 250 mm, operating at a flow rate of 5 mL min^{-1} using the gradient specified in the experimental section.

Materials

Commercial materials were used as received unless otherwise noted. Amino acids, coupling reagents and resins were obtained from Novabiochem, Fluorochem or GL Biochem. Reagents that were not commercially available were synthesized as outlined in the experimental section. Solvents were obtained as reagent grade from Merck or Fisher. $(Boc\text{-}Sec\text{-}OH)_2$ was synthesized from commercially available $(H_2N\text{-}Sec\text{-}OH)_2$ (Acros organic).

Solid Phase Peptide Synthesis (SPPS)

Preloading Rink Amide resin: Rink amide resin was initially washed with DCM (5 × 3 mL) followed by removal of the Fmoc group by treatment with 20% piperidine/DMF (2 × 5 min). The resin was washed with DMF (5 × 3 mL), DCM (5 × 3 mL) and DMF (5 × 3 mL). Oxyma pure (4 eq.) and DIC (4 eq.) were added to a solution of Fmoc-AA-OH (4 eq.) in DMF. After 5 min of pre-activation, the mixture was added to the resin. After 2 h the resin was washed with DMF (5 × 3 mL), DCM (5 × 3 mL) and DMF (5 × 3 mL), capped with acetic anhydride/pyridine (1:9 v/v) (2 × 3 min) and washed with DMF (5 × 3 mL), DCM (5 × 3 mL) and DMF (5 × 3 mL).

Preloading 2-chlorotrityl chloride resin: 2-Chlorotrityl chloride resin was swollen

in DCM for 30 min then washed with DCM (2 × 3 mL). A solution of Fmoc-AA-OH (0.5 equiv. relative to resin functionalization) and *i*Pr_2NEt (2.0 eq. relative to resin functionalization) in DCM (final concentration 0.1 M of amino acid) was added and the resin shaken at rt for 16 h. The resin was washed with DMF (5 × 3 mL) and DCM (5 × 3 mL). The resin was treated with a solution of DCM/CH_3OH/*i*Pr_2NEt (17:2:1 v/v/v, 3 mL) for 1 h and washed with DMF (5 × 3 mL), DCM (5 × 3 mL), and DMF (5 × 3 mL). The resin was subsequently submitted to iterative peptide assembly (Fmoc-SPPS).

Estimation of amino acid loading: The resin was treated with 20% piperidine/DMF (2 x 3 mL, 3 min) and 20 µL of the combined deprotection solution was diluted to 10 mL using 20% piperidine/DMF in a volumetric flask. The UV absorbance of the resulting piperidine-fulvene adduct was measured (λ = 301 nm, ε = 7800 M^{-1} cm^{-1}) to determine the amount of amino acid loaded onto the resin.

Manual Iterative peptide assembly (Fmoc-SPPS)

General amino acid coupling: A solution of protected amino acid (4 eq.), DIC (4 eq.) and oxyma pure (4 eq.) in DMF (final concentration 0.1 M) was added to the resin. After 1 h, the resin was washed with DMF (5 × 3 mL), DCM (5 × 3 mL) and DMF (5 × 3 mL).

Deprotection: The resin was treated with 20% piperidine/DMF (2 x 3 mL, 3 min) and washed with DMF (5 × 3 mL), DCM (5 × 3 mL) and DMF (5 × 3 mL).

Capping: Acetic anhydride/pyridine (1:9 v/v) was added to the resin (3 mL). After 3 min the resin was washed with DMF (5 × 3 mL), DCM (5 × 3 mL) and DMF (5 × 3 mL).

Coupling conditions for $(Boc\text{-}Sec\text{-}OH)_2$: A solution of compound $(Boc\text{-}Sec\text{-}OH)_2$ (1 eq.), DIC (2 eq.), and Oxyma pure (2 eq.) in DMF was added to the resin (1 eq.) and shaken at rt for 16 h. The resin was then washed with DMF (5 × 3 mL), and DCM (10 × 3 mL).

Cleavage: A mixture of TFA, thioanisole, tri*iso*propylsilane (TIS) and water (90:4:4:2 v/v/v/v) was added to the resin. After 3 h, the resin was washed with

TFA (3 × 2 mL).

Work-up: The combined solutions were concentrated under a stream of nitrogen to < 5 mL. 40 mL of diethyl ether was added to precipitate the peptide and the suspension centrifuged. The pellet was then dissolved in water containing 0.1% TFA, filtered and purified by preparative HPLC and analyzed by HPLC and ESI mass spectrometry.

Automated solid-phase peptide synthesis

Automated Fmoc-SPPS was carried out on a Biotage Initiator^{+} Alstra microwave peptide synthesizer. General synthetic procedures for Fmoc-deprotection and capping were carried out in accordance with the manufacturer's specifications. Standardized amino acid couplings were performed for 15 min at 50 °C under microwave irradiation in the presence of amino acid (0.5 M in DMF, 4 eq.), Oxyma (0.5 M in DMF, 4 eq.) and di*iso*propylcarbodiimide (0.5 M in DMF, 4 eq.). Peptide cleavage and work-up were carried out as described above for manual SPPS.

Selenoester synthesis general procedure

Model peptide selenoesters were prepared on 2-chlorotrityl chloride resin using Fmoc-SPPS as described in the general methods. Cleavage of the peptides from the resin was achieved by treating with 30 vol.% HFIP in DCM (5 ml) for 2 hours before concentrating *in vacuo*. The resulting residue was dissolved in DCM and cooled to 0 °C. Diphenyl diselenide (DPDS) (30 eq. in DCM) was added to the solution followed by Bu_3P (30 eq.). The reaction was allowed to proceed at 0 °C for 3 h, after which time the solvent was removed *in vacuo*. The crude material was put on ice and the protecting groups removed *via* treatment with TFA:TIS:thioanisole:H_2O (90:4:4:2 v/v/v/v). After 3 h at room temperature the cleavage cocktail was removed under a stream of N_2 and the crude residue suspended in diethyl ether and cooled to -50 °C. The precipitate was pelleted by centrifugation at 4000 rpm for 5 min, the supernatant decanted and the pellet dissolved in 0.1% TFA/H_2O with addition of 0.1% TFA/MeCN and purified *via* preparative HPLC.

General additive-free selenocystine-selenoester ligation procedure

Peptide selenoesters (1.05-1.20 eq.) and peptide dimers bearing N-terminal selenocystine (0.5 eq) were dissolved separately in ligation buffer (6 M guanidine hydrochloride, 100 mM Na_2HPO_4, pH 7.2) to a concentration of 10.5-12 mM with respect to the peptide selenoester and 5 mM with respect to the peptide diselenide dimer. The solutions were combined to give an overall concentration of 2.5 mM with respect to the selenopeptide dimer, 5.25-6 mM with respect to the peptide selenoester and a final pH of 6.2-6.5. The reaction was monitored by RP-HPLC and once complete the DPDS was extracted with hexane. Immediately before purification by preparative RP-HPLC a small amount of TCEP was added to the ligation mixture. Fractions containing the desired ligated product were lyophilized.

General additive-free cysteine-selenoester ligation procedure

The peptide selenoester (1.05 eq.) and peptide bearing N-terminal cysteine (1.0 eq) were dissolved separately in ligation buffer (6 M guanidine hydrochloride, 100 mM Na_2HPO_4, pH 7.2) to a concentration of 10.5 mM and 10 mM respectively. The solutions were combined to give an overall concentration of 5 mM with respect to the peptide bearing an N-terminal cysteine and 5.25 mM with respect to the peptide selenoester, and a final pH of the reaction of 6.5-7.0. If the pH was below 6.5 the pH was carefully adjusted with 2 M NaOH, ensuring to not exceed pH 7. The reaction was monitored by RP-HPLC and once complete the DPDS was extracted with hexane. Hydroxylamine (10 eq) was added to the ligation solution and the pendant thioester hydrolysis monitored by RP-HPLC. Once hydrolyzed, TCEP was added and purification by preparative HPLC was performed. Fractions containing the desired ligated product were lyophilized.

General conjugation protocols

General one-pot ligation-conjugation protocol

Peptide selenoesters (1.05-1.20 eq.) and peptide dimers bearing N-terminal selenocystine (0.5 eq) were dissolved separately in ligation buffer (6 M guanidine hydrochloride, 100 mM Na_2HPO_4, pH 7.2) to a concentration of 10.5-12 mM with respect to the peptide selenoester and 10 mM with respect to the selenopeptide monomer. The solutions were combined to give an overall concentration of 5.0 mM with respect to the selenol monomer, 5.25-6 mM with respect to the peptide selenoester and a final pH of the reaction of 6.0-7.0. After 10 minutes hexane (equal volume to current solution) was added to the ligation mixture to extract away the DPDS. The ligation solution was then subjected to general conjugation protocol A: to the ligation mixture was added a solution of TCEP (0.625 M stock solution adjusted to pH 7, 50 eq. (eq. based on moles), 125 mM), TEMPO trap (0.1 M solution in DMSO, 2 eq., 5 mM) and $Mn(OAc)_3$ (0.1 M stock in DMSO, 4 eq., 10 mM). The solution was diluted to a final selenol monomer concentration of 2.5 mM. The final solution composition was 20% DMSO in ligation buffer. The pH was checked to be 7-7.5 and the reaction shaken at 50 ^{o}C for 1 hour. Reaction completion was checked by RP-HPLC and purified by semi-preparative HPLC.

For selenopeptide fragments containing Cys the same ligation protocol was followed, using general protocol C for the conjugation:

General conjugation protocol A – Accelerated modification of Sec

To peptide dissolved in 20% DMSO in ligation buffer to a concentration of 5 mM was added a solution of TCEP (0.625 M stock solution in LB pH adjusted to 7, 50 eq.), TEMPO trap (0.1 M stock solution in DMSO, 2 eq.) and $Mn(OAc)_3$ (0.1 M stock solution in DMSO, 4 eq.). The pH of the reaction mixture was checked to be 7-7.5 then the reaction mixture diluted to the final peptide concentration was 2.5 mM. The reaction was then heated to 50 ^{o}C and shaken for 1 hour. Once the starting material was shown to be fully consumed by HPLC the reaction mixture was purified by semi-preparative HPLC.

General conjugation protocol B – Accelerated modification of Cys

To peptide dissolved in 20% DMSO in ligation buffer to a concentration of 2 mM was added a solution of TCEP (0.625 M stock solution in LB pH adjusted to 7, 50 eq.), TEMPO (0.1 M stock solution in DMSO, 2 eq.) and $Mn(OAc)_3$ (0.1 M stock solution in DMSO, 5 eq.). The pH of the reaction mixture was checked to be 7-7.5 then the reaction mixture diluted to the final peptide concentration was 1.0 mM. The reaction was then heated to 50 ^oC and shaken for 2 hours. Once the starting material was shown to be fully consumed by HPLC the reaction mixture was purified by semi-preparative HPLC.

General conjugation protocol C – Sec-selective TEMPO conjugation in presence of Cys

To peptide dissolved in 20% DMSO in ligation buffer to a concentration of 5 mM was added a solution of TCEP (0.625 M stock solution in LB pH adjusted to 7, 50 eq.) and TEMPO (0.1 M stock solution in DMSO, 5 eq.). The pH of the reaction mixture was checked to be 7-7.5 then the reaction mixture diluted so the final peptide concentration was 2.5 mM. The reaction was then heated to 37 ^oC and shaken for 4 hours. Analytical RP-HPLC was used to show full consumption of starting peptide and the reaction mixture was purified by semi-preparative HPLC.

General conjugation protocol D – Internal **Cys residue conjugation**

To peptide dissolved in 20% DMSO in ligation buffer to a concentration of 5 mM was added a solution of TCEP (0.625 M stock solution in LB pH adjusted to 7, 50 eq.), TEMPO (0.1 M stock solution in DMSO, 2 eq.) and $Mn(OAc)_3$ (0.1 M stock solution, 4 eq.). The pH of the reaction mixture was checked to be 7-7.5 then the reaction mixture diluted to the final peptide concentration was 2.5 mM. The reaction was then heated to 50 ^oC and shaken for 1 hour. Once the starting material was shown to be fully consumed by HPLC the reaction mixture was purified by semi-preparative HPLC.

General conjugation protocol E – Internal Cys residue protein conjugation

To peptide dissolved in 20% DMSO in ligation buffer to a concentration of 2 mM was added a solution of TCEP (0.625 M stock solution in LB, pH adjusted to 7, 100 eq.), TEMPO (0.1 M stock solution in DMSO, 5 eq.) and $Mn(OAc)_3$ (0.25 M stock solution in DMSO, 20 eq.). The pH of the reaction mixture was checked to be 7-7.5 then the reaction mixture diluted to the final peptide concentration was 1.0 mM. The reaction was then heated to 50 ^{o}C and shaken for 2 hours. Once the starting material was shown to be fully consumed by HPLC the reaction mixture was purified by semi-preparative HPLC.

General Conjugation Protocol Summary

Table S1. Summary of the conditions used in the general protocols above.

General Protocol	Peptide/ mM	TCEP eq.	TEMPO trap eq.	$Mn(OAc)_3$ eq.	Temp/ ^{o}C	Time/ hours
A	2.5	50	2	4	50	1
B	1.0	50	2	5	50	2
C	2.5	50	5	0	37	4
D	2.5	50	2	10	50	4
E	1.0	100	5	20	50	2

Compound synthesis

Tetra-EG-TEMPO (3)

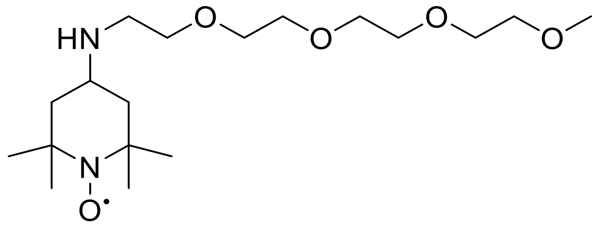

To tetraethylene glycol monomethyl ether (1.0 g, 4.80 mmol) in DCM at 0 °C was added triethylamine (3.35 ml, 24.0 mmol) followed by a mesyl chloride (1.11 ml, 14.4 mmol) dropwise. The reaction mixture was allowed to warm to room temperature and stir for 2 hours. The reaction mixture was washed with 1 M HCl (3 x 10 ml), saturated $NaHCO_3$ (3 x 10 ml), brine (10 ml), dried over $MgSO_4$ and concentrated in vacuo.

To the crude mesylate (1.30 g, 4.54 mmol) in 35% ammonia in water (50 ml) was added ammonium acetate (4.86 g, 90.8 mmol). This was stirred at room temperature for 48 hours. The reaction mixture was saturated with NaCl and extracted with DCM (5 x 20 ml). The combined organic layers were washed with brine (10 ml), dried over $MgSO_4$, filtered and concentrated in vacuo.

The resulting colourless oil (0.85 g, 4.10 mmol) was redissolved in dry MeOH (20 ml) and, under N_2, 4-oxoTEMPO (0.63 g, 3.73 mmol) and glacial acetic acid (0.19 ml, 3.36 mmol) were added. The reaction mixture was heated to 50 °C and stirred for 2 hours. The reaction mixture was cooled to room temperature and sodium cyanoborohydride (0.70 g, 11.2 mmol) was added and the reaction mixture again heated to 50 °C and stirred under N_2 for 16 hours. The reaction mixture was cooled, then poured into saturated $NaHCO_3$ (10 ml) and the aqueous layer extracted with DCM (5 x 20 ml). The organic layers were washed with brine (20 ml), dried over $MgSO_4$, filtered and concentrated in vacuo. The resultant orange oil was purified by column chromatography (DCM: MeOH 9:1) to give the title compound as an orange oil (1.13 g, 3.13 mmol, 84% yield). HRMS Calcd.: 362.2781 $[M+H]^+$. Obs.: 362.2797 $[M+H]^+$. NMR Method A used for NMR analysis. ^1H NMR (400 MHz, MeOD) δ 3.69 – 3.59 (m, 14H), 3.58 – 3.52 (m, 2H), 3.37 (s, 2H), 2.97 – 2.86 (m, 1H), 2.80 (t, J = 5.3 Hz, 2H), 1.88 – 1.80 (m, 2H), 1.35 – 1.27 (m, 3H), 1.17 (d, J = 9.8 Hz, 12H), 1.05 (s, 1H). ^{13}C

NMR (101 MHz, MeOD) δ 71.6, 70.1, 69.5, 58.3, 57.7, 45.5, 44.7, 31.6, 19.2. IR (ATR, cm^{-1}) 3276m, 3125 brw, 2989w, 2967m, 2929m, 2887m, 2854m, 1487s, 1391m, 1378m, 1179w, 1137s, 1126s, 1116m, 1098w.

Propargyl TEMPO (4)

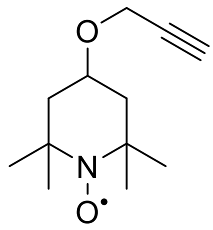

To a solution of NaH (60% in mineral oil, 0.70 g, 17.4 mmol) in anhydrous DMF (5 ml) under N_2 was added 4-hydroxy TEMPO (1.0 g, 5.81 mmol) in anhydrous DMF (5 ml). The solution was stirred for 30 minutes before being cooled to 0 °C and propargyl bromide (80% in toluene, 2.07 g, 17.4 mmol) was added dropwise over 15 minutes. The solution was then left to stir at room temperature under N_2 overnight. The solution was poured into water (25 ml), and the solution extracted with ethyl acetate (3 x 25 ml). The combined organics were washed with brine (25 ml), dried over $MgSO_4$, filtered and concentrated *in vacuo*. The resulting brown oil was purified by column chromatography (95:5 Hexane: Ethyl acetate) to give the title compound as an orange solid (0.81 g, 3.86 mmol, 66% yield). HRMS Calcd.: 211.1572 $[M+H]^+$. Obs.: 211.1574 $[M+H]^+$. NMR Method A used for NMR analysis. ^1H NMR (400 MHz, $CDCl_3$) δ 4.20 (d, J = 2.4 Hz, 2H), 3.88 (tt, J = 11.2, 4.1 Hz, 1H), 2.46 (t, J = 2.4 Hz, 1H), 1.99 (m, 2H), 1.58 – 1.42 (m, 2H), 1.24 (d, J = 18.8 Hz, 12H). ^{13}C NMR (101 MHz, $CDCl_3$) δ 74.2, 69.8, 59.6, 55.3, 44.3, 32.0, 20.7. Melting point – 58-59 °C. FTIR (ATR) $\nu max/cm^{-1}$ 3229m, 2996w, 2973w, 2953w, 2936w, 2870w, 2111w, 1466w, 1445w, 1379w, 1365m, 1348m, 1312m, 1020s, 1000m.

Fluorescein isothiocyanate-TEMPO (5)

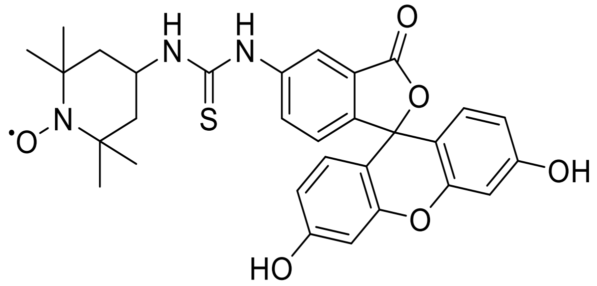

To a solution of 4-amino TEMPO (0.146 g, 0.852 mmol) in THF (6 ml) was added FITC (0.316 g, 0.811 mmol) in one portion. The suspension was left to stir at room temperature overnight. The reaction mixture was poured into water (25 ml), and the aqueous layer extracted with EtOAc (3 x 20 ml). The combined organic layers were washed with 1 M HCl (2 x 10 ml), water (1 x 10 ml) and brine (1 x 10 ml), before being dried over $MgSO_4$, filtered and concentrated *in vacuo*. The resulting orange solid was purified by column chromatography (1:4 Hexane: Ethyl acetate) to give the title compound as an orange solid (0.364 g, 80%, 0.649 mmol). HRMS Calcd.: 561.1934 $[M+H]^+$. Obs: 561.1951 $[M+H]^+$. NMR Method B used for NMR analysis. ^{1}H NMR (400 MHz, MeOD) δ 8.31 (d, *J* = 2.1 Hz, 1H), 7.85 (d, *J* = 2.1 Hz, 1H), 7.21 (d, J=2.1 Hz, 1H), 6.91 (m, 4H), 6.71 (d, *J* = 2.4 Hz, 2H), 5.03 (m, 1H), 2.43 (m, 2H), 1.91 (t, *J* = 13.3 Hz, 2H), 1.53 (d, *J* = 35.9 Hz, 12H). ^{13}C NMR (101 MHz, MeOD) δ 181.3, 168.9, 162.8, 161.1, 154.4, 141.4, 129.9, 128.4, 125.6, 120.2, 117.7, 114.8, 114.1, 111.8, 102.1, 68.5, 44.0, 41.3, 26.8, 19.0. Melting point – decomposition at 190 ^oC. IR (ATR, cm^{-1}) - 3202 brw, 3053w, 2973w, 2934w, 1732w, 1605m, 1530m, 1502m, 1443m, 1381m, 1314m, 1238m, 1206s, 1169s, 1108s, 1047m.

D-Biotin-EDA-TEMPO (6)

D-Biotin-OMe (47)

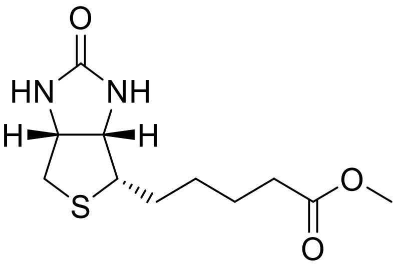

D-Biotin-OMe (**47**) was synthesized following a literature procedure[1]. To methanol (10 ml) at 0 °C was added acetyl chloride (1.46 ml, 20.5 mmol) dropwise. This was stirred for 5 minutes before adding dropwise to a suspension of D-Biotin (1.0 g, 4.09 mmol) in methanol (10 ml) at 0 °C. The resulting solution was allowed to warm to room temperature and stirred for 1 hour. Over this time the solution became clear. The reaction mixture was concentrated *in vacuo*, then redissolved in 2% methanol in DCM (50 ml). This solution was extracted with saturated $NaHCO_3$ (3 x 10 ml), water (10 ml) and brine (10 ml), then dried over $MgSO_4$, filtered and concentrated *in vacuo* to give the title compound as an off-white solid (1.04 g, 4.01 mmol, 98% yield). ^{1}H NMR (400 MHz, DMSO-d6) δ 6.50 – 6.43 (s, 1H), 6.40 – 6.33 (s, 1H), 4.39 – 4.24 (m, 1H), 4.14 (dd, J = 7.7, 4.4 Hz, 1H), 3.59 (s, 3H), 3.10 (ddd, J = 8.6, 6.2, 4.4 Hz, 1H), 2.83 (dd, J = 12.5, 5.1 Hz, 1H), 2.58 (d, J = 12.4 Hz, 1H), 2.51 (p, J = 1.8 Hz, 1H), 2.30 (t, J = 7.4 Hz, 2H), 1.70 – 1.26 (m, 6H).^{13}C NMR (101 MHz, DMSO-d6) δ 173.8, 163.2, 61.5, 61.4, 59.6, 59.5, 55.8, 51.7, 33.6, 28.5, 24.9.

D-Biotin-ethylenediamine (48)

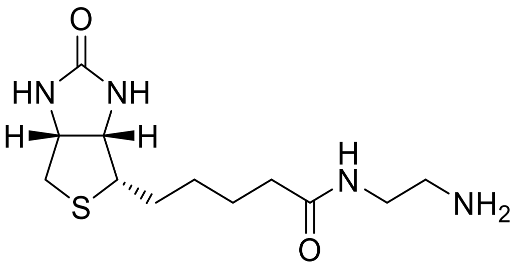

D-Biotin-ethylenediamine (**48**) was synthesized following a literature procedure[1]. To D-Biotin-OMe (**47**, 1.0 g, 3.87 mmol) in methanol (25 ml) was added ethylenediamine (6.5 ml, 96.8 mmol). The resulting solution was stirred at 60 °C for 16 hours. TLC was used to confirm complete consumption of the

starting material at this point. The reaction mixture was concentrated *in vacuo*, with excess ethylenediamine being co-evaporated with toluene (3 x 20 ml). The resulting solid was washed with ethyl acetate (3 x 25 ml) and dried under high vacuum to give the title compound as an off-white solid (1.0 g, 3.49 mmol, 90% yield). HRMS Calcd.: 287.1542 $[M+H]^+$. Obs.: 287.1563 $[M+H]^+$. ^{1}H NMR (400 MHz, DMSO-d6) δ 7.74 (t, J = 5.6 Hz, 1H), 6.44 (s, 1H), 6.37 (s, 1H), 4.35 – 4.28 (m, 1H), 4.13 (ddd, J = 7.8, 4.5, 1.9 Hz, 1H), 3.10 (ddd, J = 8.5, 6.1, 4.4 Hz, 1H), 3.02 (q, J = 6.2 Hz, 2H), 2.83 (dd, J = 12.4, 5.1 Hz, 1H), 2.58 (d, J = 15.4 Hz, 1H), 2.53 (t, J = 6.4 Hz, 2H), 2.06 (t, J = 7.4 Hz, 2H), 1.69 – 1.22 (m, 6H). ^{13}C NMR (101 MHz, DMSO-d6) δ 172.5, 163.2, 61.5, 59.7, 55.9, 42.7, 41.9, 40.6, 35.7, 28.7, 28.5, 25.8.

D-Biotin-EDA-TEMPO (6)

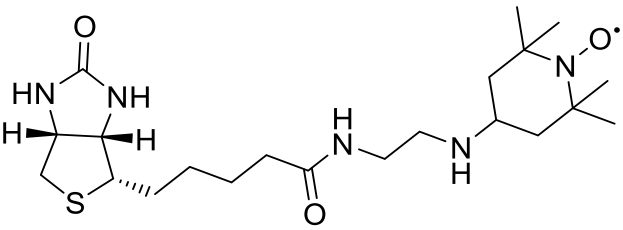

To 4-oxoTEMPO (0.5 g, 2.94 mmol) and D-biotin-EDA (**48**, 1.01 g, 3.52 mmol) in dry methanol (5 ml) under nitrogen was added glacial acetic acid (0.15 ml, 2.65 mmol). The solution was heated to 50 °C and stirred under nitrogen for 3 hours. At this time the solution was allowed to cool to room temperature and sodium cyanoborohydride (0.20 g, 3.23 mmol) was added in a single portion. The reaction mixture was again heated to 50 °C and stirred under nitrogen for 16 hours. The reaction was quenched by addition of saturated sodium bicarbonate (5 ml) and the aqueous layer extracted with DCM (5 x 10 ml). The combined organics were washed with brine (5 ml), dried over $MgSO_4$, filtered and concentrated *in vacuo*. The resultant orange oil was dried under high vacuum to give the title compound as a pale orange crystalline solid (1.11 g, 2.53 mmol, 86% yield). HRMS – Calcd. $[M+H]^+$ - 441.2783. Obs. $[M+H]^+$ - 441.2774. NMR Method B used for NMR analysis. ^{1}H NMR (400 MHz, MeOD) δ 4.53 (dd, *J* = 7.9, 4.4 Hz, 1H), 4.34 (dd, *J* = 7.9, 4.4 Hz, 1H), 3.90 (m, 1H), 3.58 – 3.52 (m, 2H), 3.30 – 3.20 (m, 3H), 2.95 (dd, *J* = 12.8, 5.0 Hz, 1H), 2.73 (d, *J* = 12.8 Hz, 1H), 2.44 (m, 2H), 2.29 (t, *J* = 7.4 Hz, 2H), 2.06 (m, 2H), 1.83

– 1.56 (m, 4H), 1.52 (d, J = 4.5 Hz, 12H). ^{13}C NMR (101 MHz, MeOD) δ 177.6, 166.1, 162.8, 119.4, 116.5, 68.5, 63.3, 61.7, 57.0, 46.2, 41.0, 37.2, 36.4, 29.7, 29.4, 28.2, 26.4, 20.2. Melting Point (˚C) - 54-56. FTIR (ATR) νmax/cm^{-1} 3271brm, 3080w, 2971w, 2929m, 2859w, 1692s, 1648s, 1546m, 1458m, 1376w, 1361w, 1331w, 1308w, 1243w, 1179w, 1120w.

DMTMM

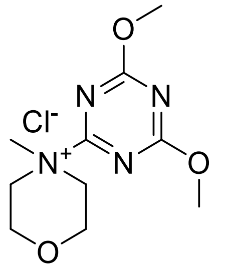

DMTMM was synthesized following a literature procedure[2]. To a solution of CDMT (2.5 g, 14.2 mmol) in THF (25 ml) was added NMM (1.49 ml, 13.5 mmol) dropwise. Almost immediately a white precipitate began to form. The reaction was left to stir for a further 15 minutes before being filtered, and the white crystals washed with THF. The crystals were then dried under high vacuum to give the title compound as free-flowing white crystals (3.51 g, 12.69 mmol, 94%). HRMS calcd.: 241.1301 $[M]^+$ Obs.: 241.1321 $[M]^+$. ^1H NMR (400 MHz, DMSO-d_6) δ 4.36 (d, 2H), 4.11 (s, 6H), 4.05 – 3.89 (m, 4H), 3.82 – 3.71 (m, 2H), 3.50 (s, 3H). ^{13}C NMR (101 MHz, DMSO-d_6) δ 173.8, 170.6, 149.9, 61.8, 59.9, 57.2.

Gemcitabine-TEMPO (7)

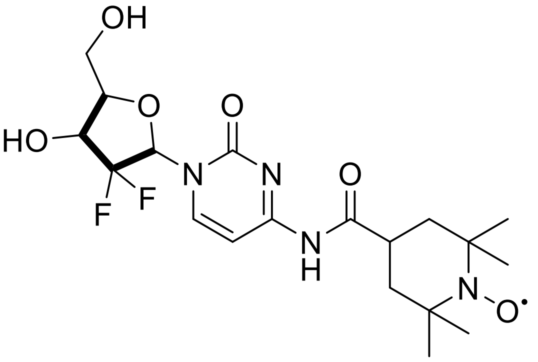

To gemcitabine hydrochloride (0.25 g, 0.834 mmol) and 4-carboxyTEMPO (0.18 g, 0.918 mmol) in DMF: DMSO 3:1 (10 ml) was added NMM (0.10 ml, 0.918 mmol), HOBt (0.12 g, 0.918 mmol) and EDC.HCl (0.21 g, 1.08 mmol).

The reaction mixture was stirred at room temperature for 16 hours before adding brine (10 ml) and extracting with EtOAc (3 x 20 ml). The combined organic layers were washed with 20% LiCl (10 ml), sat. $NaHCO_3$ (10 ml), brine (10 ml), dried over $MgSO_4$, filtered and concentrated under reduced pressure. The resultant oil was purified by column chromatography ($CHCl_3$: MeOH 9.5:0.5) to give the title compound as an orange solid (0.15 g, 0.336 mmol, 40%). HRMS Calcd.: 446.1977 $[M+H]^+$. Obs.: 446.1978 $[M+H]^+$. NMR Method B used for NMR analysis. ^{1}H NMR (400 MHz, MeOD) δ 8.40 (d, J = 8.0 Hz, 1H), 7.46 (d, J = 8.0 Hz, 1H), 6.29 (t, *J* = 8 Hz, 1H), 4.31 (m, 1H), 4.01 (m, 2H), 3.89 – 3.80 (m, 1H), 2.21 (dd, J = 14.8, 3.3 Hz, 2H), 2.04 (t, J = 13.6 Hz, 2H), 1.51 (d, J = 13.6 Hz, 12H). ^{13}C NMR (101 MHz, MeOD) δ 173.8, 163.4, 156.1, 144.8, 125.1, 122.5, 96.9, 81.5, 69.0, 67.6, 58.8, 38.6, 35.1, 26.8, 18.7. ^{19}F NMR (376 MHz, MeOD) δ -119.15 (d, J = 248 Hz, 1F), -119.82 (dd, J = 236 Hz, 1F). Melting point – 212-214 °C. IR (ATR, cm^{-1}) - 3172m, 2975w, 2933m, 1720 m, 1648s, 1617m, 1559s, 1491s, 1467s, 1437m, 1384s, 1314m, 1275s, 1241m, 1100s, 1083s, 1071s.

Synthesis of Boc-D-Sec-OH (52)

Methyl 2-((*tert*-butoxycarbonyl)amino)-3-iodopropanoate (49)

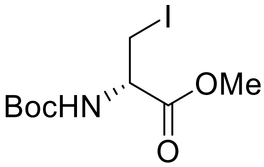

Triphenylphosphine (897 mg, 3.42 mmol) and imidazole (233 mg, 3.42 mmol) were dissolved in anhydrous dichloromethane (12 ml) and cooled to 0 °C. Iodine (866 mg, 3.42 mmol) was added and the reaction mixture was warmed to room temperature and stirred for 10 min, before cooling to 0 °C again. Boc-D-Ser-OH (500 mg, 2.28 mmol) in anhydrous dichloromethane (8 ml) was added and the reaction mixture was stirred at 0 °C for 2 h. The reaction mixture was then warmed to room temperature and passed through a short silica plug, washing with 1:1 diethyl ether:light petroleum, then concentrated *in vacuo* to give the title compound as a yellow oil (700 mg, 2.13 mmol, 93%). HRMS calcd.: $[M+Na]^+$ = 352.0016, Obs.: $[M+Na]^+$ 352.0032; ^{1}H NMR (400 MHz, $CDCl_3$) δ 5.35 (d, *J* = 6.5 Hz, 1H, NH), 4.51 (dt, *J* = 7.4, 3.6 Hz, 1H, CH), 3.79 (s, 3H,

CH_3), 3.60-3.51 (m, 2H, CH_2), 1.45 (s, 9H, Boc); ^{13}C NMR (100 MHz, $CDCl_3$) δ 170.2 (C), 155.0 (C), 80.6 (C), 53.8 (CH), 53.2 (CH_3), 28.4 (Boc), 8.01 (CH_2).

Methyl-2-((*tert*-butoxycarbonyl)amino)-3-selenocyanatopropanoate (50)

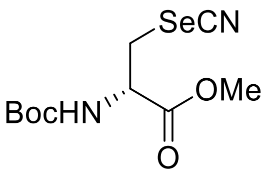

Methyl 2-((*tert*-butoxycarbonyl)amino)-3-iodopropanoate (**49**, 700 mg, 2.13 mmol) was dissolved in acetonitrile (20 ml). Potassium selenocyanate (613 mg, 4.26 mmol) was added and the reaction mixture was heated to 60 °C and stirred for 16 h. The reaction mixture was then cooled to room temperature and concentrated under reduced pressure. The residue was then dissolved in dichloromethane (50 ml) and water (50 ml) was added. The layers were separated, and the aqueous phase was re-extracted using dichloromethane (3 x 50 ml). The combined organics were washed with brine (200 ml), dried using Na_2SO_4 and concentrated *in vacuo*. The crude product was purified by column chromatography eluting with 25% ethyl acetate in light petroleum to give the title compound as a yellow oil (490 mg, 1.59 mmol, 76%). HRMS calcd. $[M+H]^+$ = 309.0348, Obs. $[M+H]^+$ = 309.0344; ^1H NMR (400 MHz, $CDCl_3$) δ 5.50 (br s, 1H, NH), 4.74 (d, J = 4.3, 1H, CH), 3.84 (s, 3H, CH_3), 3.55 (qd, J = 12.6, 4.5 Hz, 2H, CH_2), 1.45 (s, 9H, Boc); ^{13}C NMR (100 MHz, $CDCl_3$) δ 169.8 (C), 155.1 (C), 101.2 (C), 81.2 (C), 53.4 (CH_3), 31.9 (CH_2), 28.4 (Boc), 14.3 (CH).

Methyl-2-((*tert*-butoxycarbonyl)amino)-3-(methylselanyl)propanoate (51)

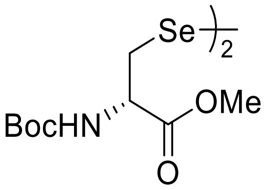

Methyl-2-((*tert*-butoxycarbonyl)amino)-3-selenocyanatopropanoate (**50**, 280 mg, 0.91 mmol) was dissolved in anhydrous THF (2 ml) and cooled to 0 °C. $NaBH_4$ (67 mg, 1.81 mmol) in a 95% ethanol solution (0.5 ml) was added

dropwise. The reaction mixture was stirred at 0 °C for 2 h, then diluted with ethyl acetate (20 ml) and 1M $HCl_{(aq)}$ (20 ml). The layers were separated, and the aqueous phase was extracted using ethyl acetate (3 x 20 ml). The combined organics were dried using Na_2SO_4 and concentrated *in vacuo*. The crude product was purified by column chromatography eluting with 25% ethyl acetate in light petroleum to give the title compound as a yellow solid (110 mg, 0.39 mmol, 43%). HRMS calcd. $[M+Na]^+$ = 587.0487, Obs. $[M+Na]^+$ = 587.0467; ^1H NMR (400 MHz, $CDCl_3$) δ 5.40 (d, J = 7.3 Hz, 1H, NH), 4.61-4.58 (m, 1H, CH), 3.74 (s, 3H, CH_3), 3.37 (qd, J = 13.0, 5.5 Hz, 2H, CH_2), 1.43 (s, 9H, Boc). ^{13}C NMR (100 MHz, $CDCl_3$) δ 169.8, 155.1, 101.2, 81.2, 53.4, 31.9, 28.4, 14.3.

2-((*tert*-Butoxycarbonyl)amino)-3-(methylselanyl)propanoic acid (52)

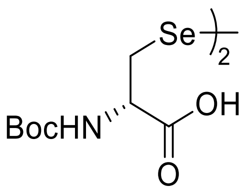

A 1 M NaOH solution (5 ml) was prepared and added to methanol (5 ml) and cooled to 0 °C. Methyl-2-((*tert*-butoxycarbonyl)amino)-3-(methylselanyl) propanoate (**51**, 110 mg, 0.39 mmol) was dissolved in methanol (5 ml) and added dropwise to the NaOH/methanol solution. The reaction mixture was stirred at 0 °C for 20 min, before adding water (50 ml) and diethyl ether (50 ml). The layers were separated, and the aqueous phase was washed using diethyl ether (50 ml). The aqueous phase was then acidified to pH 2 using 1 M $HCl_{(aq)}$ and extracted using ethyl acetate (3 x 50 ml). The combined organics were washed with brine (100 ml), dried using $MgSO_4$ and concentrated *in vacuo* to give the title compound as a pale-yellow solid (105 mg, 0.39 mmol, 99%). The crude product was reacted on without purification. HRMS calcd. $[M-H]^-$ = 536.0125, Obs. $[M-H]^-$ = 536.0117.

H-AF-OMe (53)

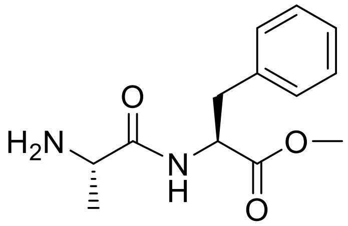

To a solution of Boc-Ala-OH (1.03 g, 5.48 mmol) and H-Phe-OMe.HCl (1.30 g, 6.03 mmol) in DMF (10 ml) was added DMTMM (1.70 g, 6.03 mmol) portion-wise. This was left to stir at room temperature overnight before being diluted with water (50 ml) and extracted with ethyl acetate (3 x 50 ml). The combined organic layers were then washed with 1 M HCl (3 x 20 ml), 1 M $NaHCO_3$ (3 x 20 ml), water (1 x 20 ml) and brine (1 x 20 ml), dried over $MgSO_4$, filtered and concentrated *in vacuo*. The resulting white foam was redissolved in 50% TFA in DCM (100 ml) and stirred for 1 hour. This was concentrated *in vacuo,* with excess TFA co-evaporated with toluene (3 x 25 ml). The resulting white solid was washed with diethyl ether (3 x 50 ml) to give the title compound as a free-flowing white powder (1.26 g, 5.04 mmol, 92% yield). HRMS Calcd.: 251.1396 $[M+H]^+$. Obs.: 251.1387 $[M+H]^+$. ^1H NMR (400 MHz, DMSO-d6) δ 8.86 (d, J = 7.5 Hz, 1H, NH), 8.12 (s, 3H, NH_3), 7.47 – 7.16 (m, 5H, Ar-H), 4.54 (ddd, J = 9.2, 7.5, 5.3 Hz, 1H, CH_α), 3.84 (q, J = 7.0 Hz, 1H, CH_α), 3.63 (s, 3H, OCH_3), 3.10 (dd, J = 14.0, 5.3 Hz, 1H, $CH_{2\beta}$), 2.96 (dd, J = 14.0, 9.3 Hz, 1H, $CH_{2\beta}$), 1.34 (d, J = 6.9 Hz, 3H, CH_3). ^{13}C NMR (101 MHz, DMSO-d6) δ 171.9, 170.3, 137.4, 129.5, 128.8, 127.2, 54.4, 52.5, 48.4, 36.7, 17.5.

H-UAF-OMe (1a)

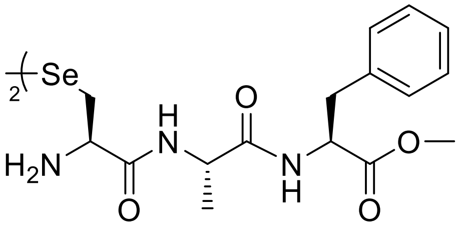

To Boc-Sec-OH (0.1 g, 0.187 mmol) and H-AF-OMe (**53**, 0.094 g, 0.374 mmol) in DMF (5 ml) was added NMM (0.062 ml, 0.561 mmol) and DMTMM (0.103 g, 0.374 mmol). The solution was stirred at room temperature for 16 hours before being diluted with 1 M HCl (5 ml) and extracted with ethyl acetate (3 x 20 ml).

The combined organic layers were washed with 1 M HCl (3 x 5 ml), saturated $NaHCO_3$ (3 x 5 ml), water (5 ml) and brine (5 ml), then dried over $MgSO_4$, filtered and concentrated in vacuo. The resultant residue was dissolved in a 1:1 solution of DCM: TFA (10 ml) and stirred at room temperature for 30 minutes before being concentrated in vacuo. The remaining TFA was co-evaporated using toluene (2 x 5 ml). The resultant solid was washed with diethyl ether (3 x 20 ml), then dried under high vacuum to give the title compound as a pale-yellow solid (0.125 g, 0.157 mmol, 84% yield). HRMS Calcd. $[M+H]^+$ = 801.1650. Obs. $[M+H]^+$ = 801.1670. ^1H NMR (400 MHz, MeOD) δ 8.53 (d, *J* = 7.7 Hz, 1H), 7.35 – 7.11 (m, 5H), 4.64 (m, 1H), 4.58 (m, 1H), 4.19 (dd, *J* = 8.0, 4.0 Hz, 1H), 3.67 (s, 3H), 3.47 (dd, *J* = 13.6, 5.0 Hz, 1H), 3.18 – 3.10 (m, 2H), 3.01 (dd, *J* = 13.9, 8.4 Hz, 1H), 1.37 (d, *J* = 7.1 Hz, 3H). ^{13}C NMR (101 MHz, MeOD) δ 174.6, 173.3, 168.5, 138.2, 130.4, 129.8, 128.1, 55.7, 54.0, 52.9, 50.4, 38.4, 30.3, 18.7.

H-D-UAF-OMe (1b)

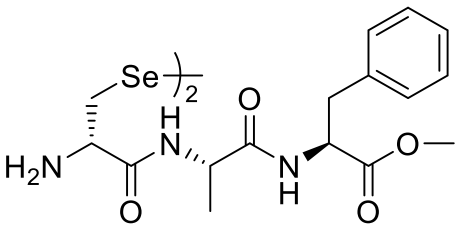

2-((*tert*-Butoxycarbonyl)amino)-3-(methylselanyl)propanoic acid (**52**, 105 mg, 0.39 mmol) was dissolved in anhydrous DMF (2 ml). H_2N-Ala-Phe-OMe (53, 206 mg, 0.82 mmol) was added, followed by *N*-methyl morpholine (172 µl, 1.57 mmol) and DMTMM (227 mg, 0.82 mmol). The reaction mixture was stirred at room temperature for 16 h, before diluting with ethyl acetate (25 ml) and water (25 ml). The organic phase was washed with water (3 x 25 ml), 1 M $HCl_{(aq)}$ (25 ml) and brine (25 ml), dried using $MgSO_4$ and concentrated *in vacuo* to give the protected title compound as a yellow gum (143 mg, 0.28 mmol, 71%). The crude product was reacted on without purification.

Boc-D-H-UAF-OMe (143 mg, 0.28 mmol) was dissolved in dichloromethane (1.5 ml) and trifluoroacetic acid (1 ml) was added. The reaction mixture was stirred at room temperature for 1 h, before concentrating *in vacuo*. Toluene (2 x 10 ml) was added to the residue and then removed *in vacuo* to remove excess

trifluoroacetic acid. The crude product was purified by preparative HPLC (gradient 15-80% over 30 minutes), product eluting at 24 min. The title compound was isolated as a white solid (21 mg, 0.03 mmol, 9%). HRMS Calcd. $[M+H]^+$ = 801.1650. Obs. $[M+H]^+$ = 801.1632. ^{1}H NMR (400 MHz, MeOD) δ 7.31 – 7.18 (m, 5H), 4.65 (dd, J = 8.3, 5.8 Hz, 1H), 4.39 (q, J = 7.2 Hz, 1H), 4.17 (dd, J = 7.4, 5.8 Hz, 1H), 3.68 (s, 3H), 3.51 (dd, J = 13.5, 5.8 Hz, 2H), 3.18 – 2.98 (m, 2H), 1.37 (d, J = 7.2 Hz, 3H). ^{13}C NMR (100 MHz, MeOD) δ 174.7, 173.2, 168.3, 138.0, 130.3, 129.6, 128.0, 55.5, 54.3, 52.7, 50.6, 38.2, 29.6, 18.4.

H-CAF-OMe (13)

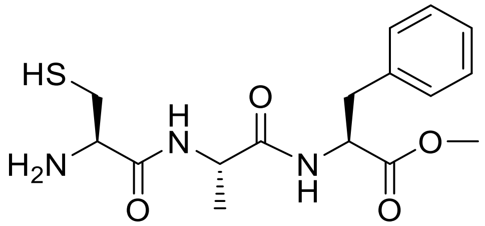

To H-AF-OMe (**53**, 0.15 g, 0.59 mmol) and Boc-Cys(Trt)-OH (0.25 g, 0.54 mmol) in DMF (10 ml) was added NMM (0.18 ml, 1.62 mmol) and DMTMM (0.16 g, 0.59 mmol). The reaction mixture was stirred at room temperature for 16 hours before being diluted with H_2O (10 ml) and extracted with EtOAc (3 x 20 ml). The combined organic layers were washed with 1 M HCl (3 x 10 ml), saturated $NaHCO_3$ (3 x 10 ml) and brine (10 ml), then dried with $MgSO_4$, filtered and concentrated *in vacuo* to yield a colourless residue. The residue was redissolved in a solution of 1:1:0.05 DCM:TFA:TIS (25 ml) and stirred at room temperature for 30 minutes before being concentrated *in vacuo*. The excess TFA was co-evaporated with toluene (3 x 10 ml). The resulting solid was washed with Et_2O (3 x 20 ml) and dried under high vacuum to yield an off white solid. This was purified by reversed-phase preparative HPLC (10-80%B over 30 minutes, 6 ml/min) to give the title compound as a white solid after lyophilisation (0.12 g, 0.34 mmol, 63% yield). HRMS – Calcd. $[M+H]^+$ = 354.1488. Obs. $[M+H]^+$ = 354.1500. ^{1}H NMR (400 MHz, MeOD) δ 7.36 – 7.19 (m, 5H), 4.71 – 4.64 (m, 1H), 4.63 – 4.56 (m, 1H), 4.21 (q, J = 8.0, 4.9 Hz, 1H), 3.69 (s, 3H), 3.49 (dd, J = 13.6, 5.0 Hz, 1H), 3.15 (m, 2H), 3.03 (dd, J = 13.9, 8.4 Hz, 1H), 1.39 (d, J = 7.1 Hz, 3H). ^{13}C NMR (101 MHz, MeOD) δ 173.1,

171.8, 167.0, 136.6, 128.9, 128.2, 126.6, 54.2, 52.5, 51.4, 48.9, 36.9, 28.8, 17.1.

3-azidopropyl-1-amine (54)

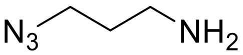

To 3-bromopropyl-1-amine (1.0 g, 4.59 mmol) in H_2O (50 ml) was added sodium azide (0.98 g, 15.1 mmol). The reaction mixture was heated to reflux for 16 hours before being cooled on ice and KOH pellets (2.5 g, 45.2 mmol) slowly added. After stirring for 30 minutes on ice the reaction mixture was extracted with DCM (5 x 20 ml), the combined organic extracts dried over K_2CO_3, filtered and concentrated under reduced pressure to yield a pale yellow oil (0.28 g, 2.85 mmol, 62% yield). ^{1}H NMR (400 MHz, Chloroform-*d*) δ 3.39 (t, J = 6.7 Hz, 2H), 2.82 (t, J = 6.8 Hz, 2H), 1.74 (p, J = 6.8 Hz, 2H). ^{13}C NMR (101 MHz, $CDCl_3$) δ 77.4, 77.1, 76.7, 53.4, 49.2, 39.3, 32.4.

EPR analysis of TEMPO traps (3 - 7)

See General Methods for details of EPR equipment and analysis parameters. Spectra were simulated using WINEPR SimFonia software (black trace – data; red trace – simulation).

Table S2. EPR parameters for analysis of TEMPO and traps **3-7**.

	g_{iso}	Linewidth / G	Lineshape	a_N / G
Tempo	2.0057	2.6	Lorentzian	16.0
Tetra-EG (**3**)	2.0057	2.6	Lorentzian	15.9
Propargyl (**4**)	2.0058	2.8	Lorentzian	15.9
Fluorescein (**5**)	2.0057	2.5	Lorentzian	15.8
Biotin (**6**)	2.0057	2.5	Lorentzian	15.8
Gemcitabine (**7**)	2.0057	2.4	Lorentzian	15.7

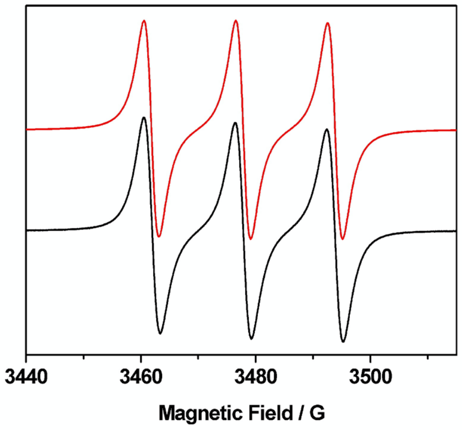

Figure S1. EPR spectra (black trace), and simulation of spectra (red trace), for TEMPO.

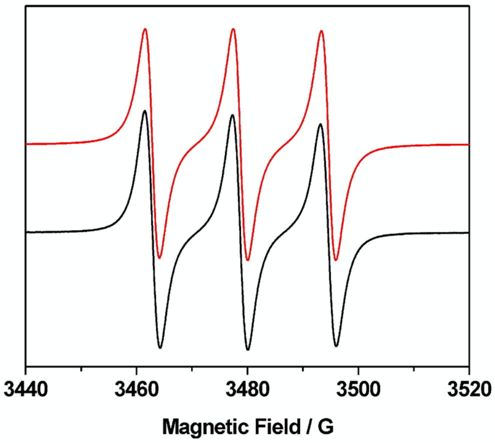

Figure S2. EPR spectra (black trace), and simulation of spectra (red trace), for Tetra-EG trap (**3**).

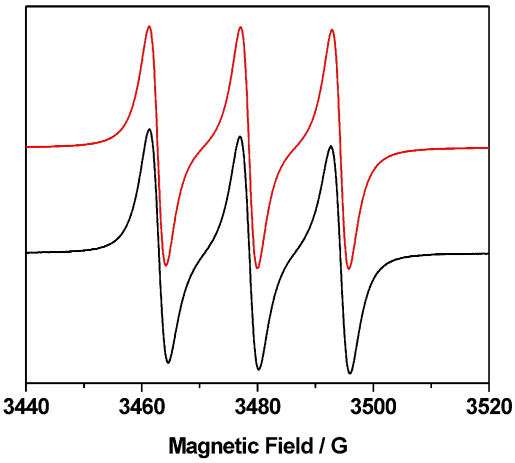

Figure S3. EPR spectra (black trace), and simulation of spectra (red trace), for Propargyl trap (**4**).

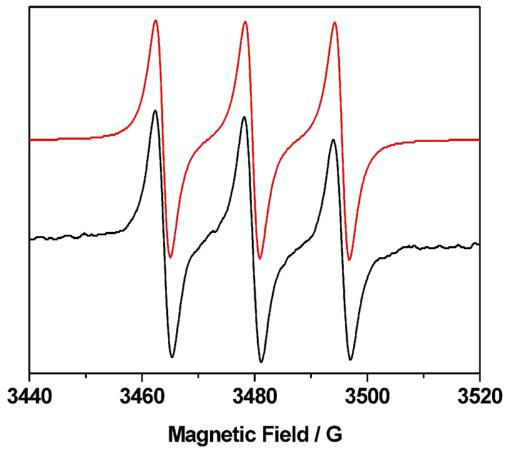

Figure S4. EPR spectra (black trace), and simulation of spectra (red trace), for Fluorescein trap (**5**)

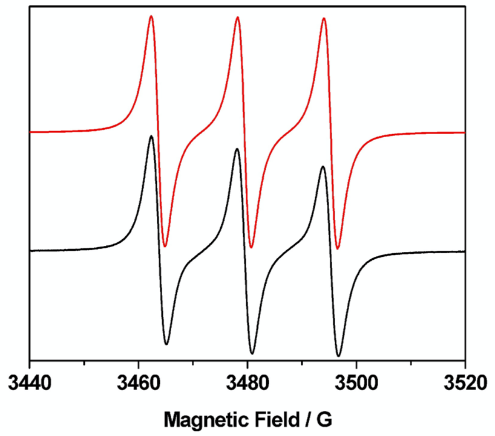

Figure S5. EPR spectra (black trace), and simulation of spectra (red trace), for Biotin trap (**6**)

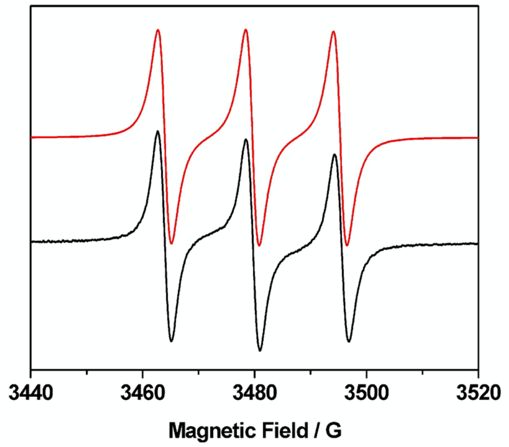

Figure S6. EPR spectra (black trace), and simulation of spectra (red trace), for Gemcitabine trap (**7**).

Initial optimization of the conjugation protocol

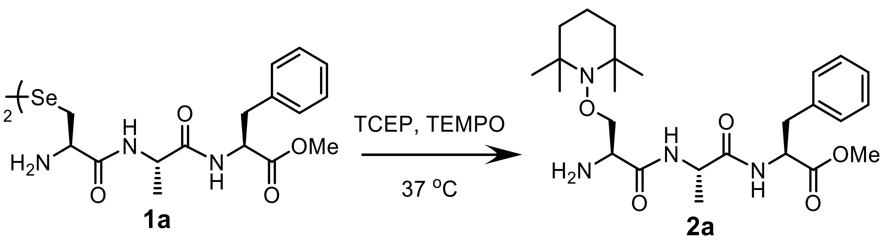

Unless otherwise specified the following method was followed:

To H-UAF-OMe (2.5 mM final concentration) in ligation buffer (LB)/MeOH (20% MeOH) at pH 7-7.5 was added TCEP (0.625 M in LB pH adjusted to 7) followed by TEMPO (0.5 M in MeOH). The reaction was shaken at 37 °C and analyzed by RP-HPLC (10-70% B over 5 minutes) at the specified time points. When doping, the same quantities of TCEP and TEMPO were added at 15, 45 and 60 minutes if doping three times. If doping twice, additional reagents added at 15 and 45 minutes. If doping once, additional reagents were added at 15 minutes. All reactions were performed in a Thermo heater/shaker set to the specified temperature and shaken at 800 rpm. Conversion to product reported was measured based on integration of the product peak from the HPLC trace compared to integration of starting material and by-product peaks.

Initial Optimization – Peptide Concentration

Table S3. TEMPO conjugation optimization by varying peptide concentration.

Trial	Peptide/ mM	TCEP eq.	TEMPO eq.	Doping	2 hrs %conv.	3 hrs %conv.	16 hrs %conv.
1	5	50	25	3	96	100	100
2	2.5	50	25	3	97	100	100
3	1	50	25	3	82	95	100
4	0.1	50	25	3	29	47	100

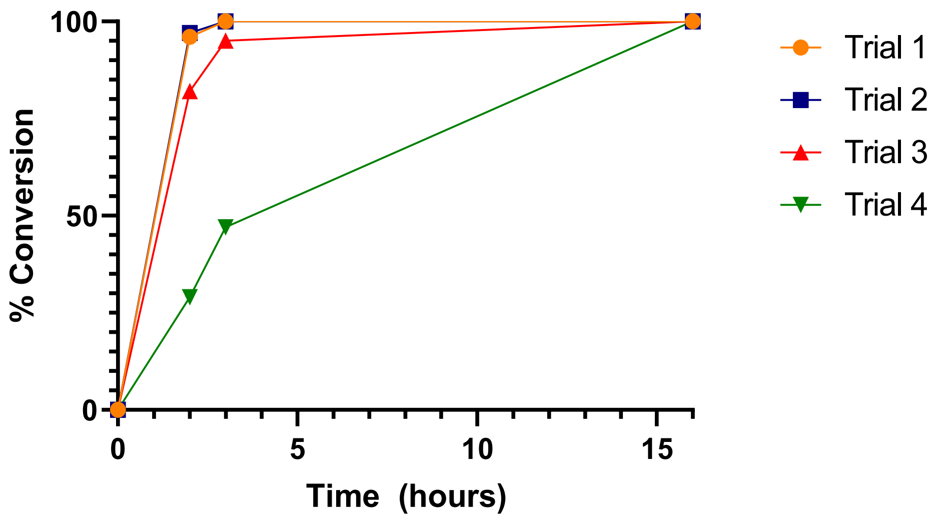

Figure S7. H-UAF-OMe TEMPO trapping optimization – peptide concentration.

Initial Optimization – TCEP Concentration

Table S4. TEMPO conjugation optimization by varying TCEP concentration.

Trial	Peptide/ mM	TCEP eq.	TEMPO eq.	Doping	1 hr %conv.	3 hrs %conv.	16 hrs %conv.
5	2.5	100	25	3	84	100	100
6	2.5	50	25	3	80	100	100
7	2.5	25	25	3	66	97	100
8	2.5	5	25	3	26	50	81

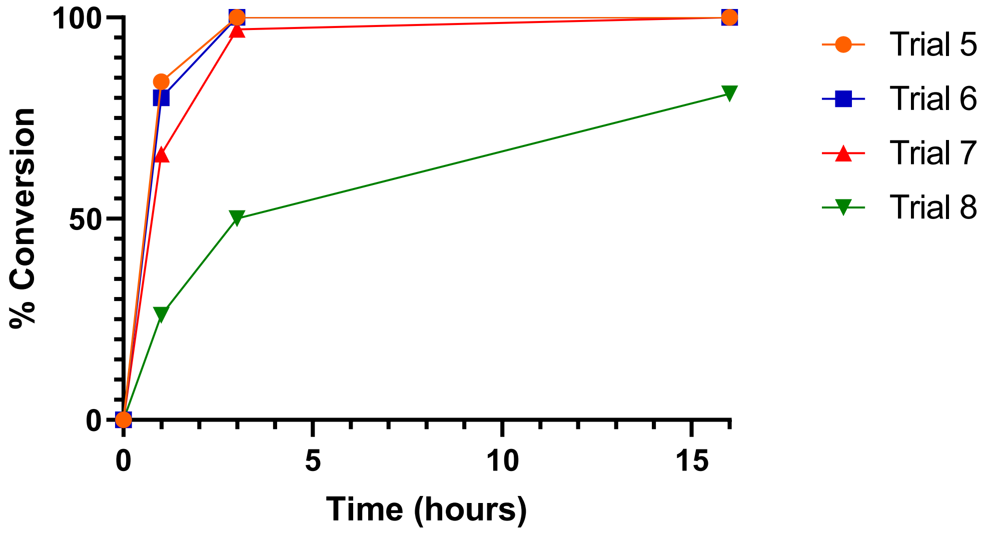

Figure S8. H-UAF-OMe TEMPO trapping optimization – TCEP concentration.

Doping Optimization

Table S5. TEMPO conjugation optimization by varying the number of dopes.

Trial	Peptide/ mM	TCEP eq.	TEMPO eq.	Doping	1 hr %conv.	3 hrs %conv.	16 hrs %conv.
9	2.5	50	25	3	97	97	100
10	2.5	50	25	2	94	98	100
11	2.5	50	25	1	86	92	100
12	2.5	50	25	0	82	80	100

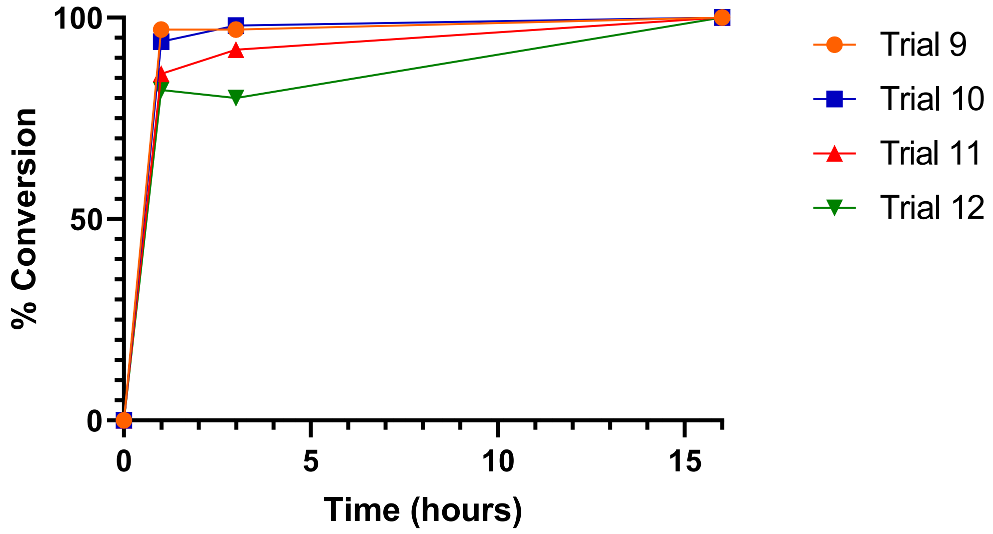

Figure S9. H-UAF-OMe TEMPO trapping optimization – Reagent doping; entry 12 complete within 6 hrs.

TEMPO Optimization

Table S6. TEMPO conjugation optimization by varying TEMPO concentration.

Trial	Peptide/ mM	TCEP eq.	TEMPO eq.	Doping	1 hr %conv.	3 hrs % conv.	16 hrs %conv.
13	2.5	50	25	2	69	94	100
14	2.5	50	10	2	64	95	100
15	2.5	50	5	2	43	93	100
16	2.5	50	2	2	26	55	93

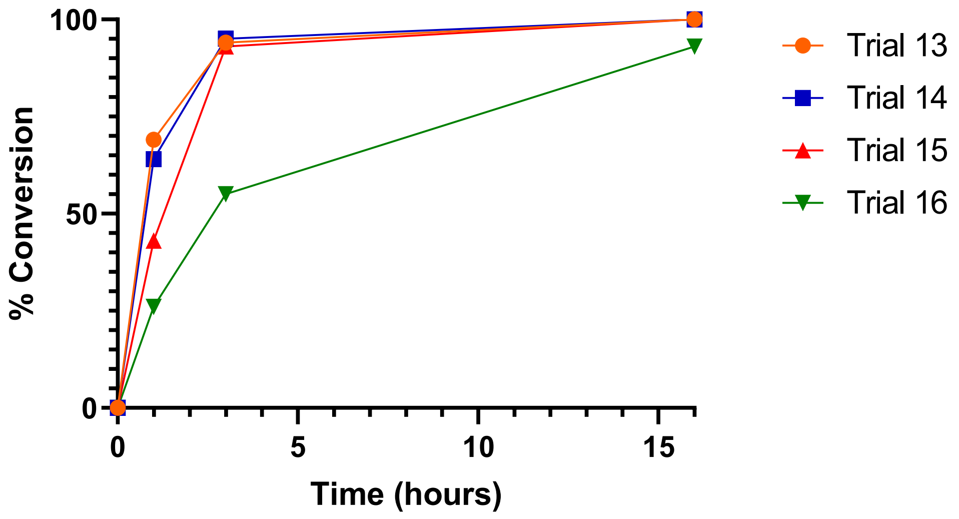

Figure S10. H-UAF-OMe TEMPO trapping optimization – TEMPO concentration.

pH Optimization

Table S7. TEMPO conjugation optimization by varying the reaction pH.

Trial	Peptide/ mM	TCEP eq.	TEMPO eq.	Doping	pH	1 hr %conv.	3 hrs %conv.	16 hrs %conv.
17	2.5	25	5	2	4	26	67	80*
18	2.5	25	5	2	5	23	66	86*
19	2.5	25	5	2	6	27	83	100
20	2.5	25	5	2	7	39	100	100
21	2.5	25	5	2	8	22	76	100

* Remainder is Ala byproduct

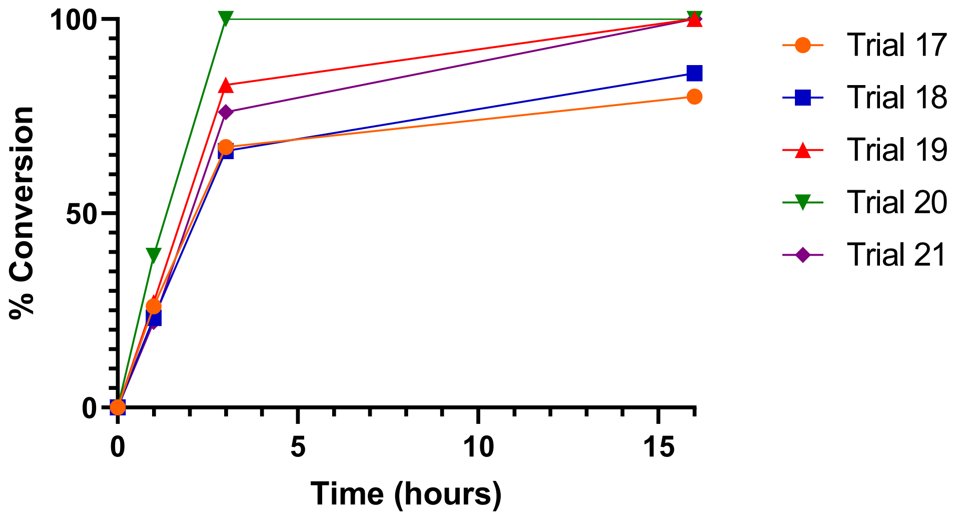

Figure S11. H-UAF-OMe TEMPO trapping optimization – pH.

Organic co-solvent composition

Table S8. TEMPO conjugation optimization by varying the organic co-solvent composition.

Trial	Peptide/ mM	TCEP eq.	TEMPO eq.	Doping	Organic / %	1 hr %conv.	3 hrs %conv.	16 hrs %conv.
22	2.5	25	5	2	0	45	79	100
23	2.5	25	5	2	20	43	79	100
24	2.5	25	5	2	70	72	86	89*
25	2.5	25	5	2	90	80	80	86*

* Remainder is Ala byproduct

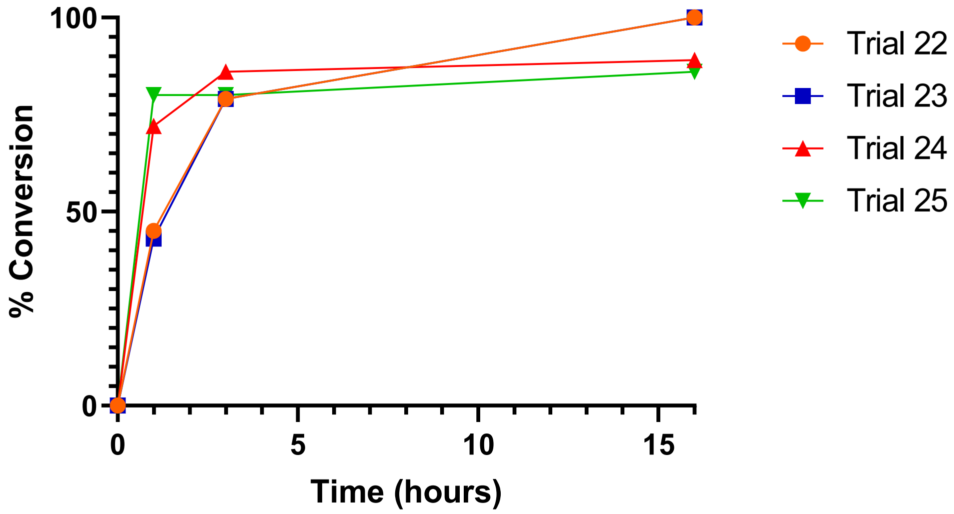

Figure S12. H-UAF-OMe TEMPO trapping optimization – composition of organic co-solvent.

Organic Solvent Compatibility

Table S9. Assessing organic co-solvent compatibility for the TEMPO conjugation reaction.

Trial	Peptide / mM	TCEP eq.	TEMPO eq.	Doping	Organic / 50%	2 hrs %conv.	3 hrs %conv.	16 hrs %conv.
26	2.5	25	5	2	DMSO	94	95	100
27	2.5	25	5	2	DMF	93	94	100
28	2.5	25	5	2	DME	90	91	100
29	2.5	25	5	2	Acetonitrile	96	96	100

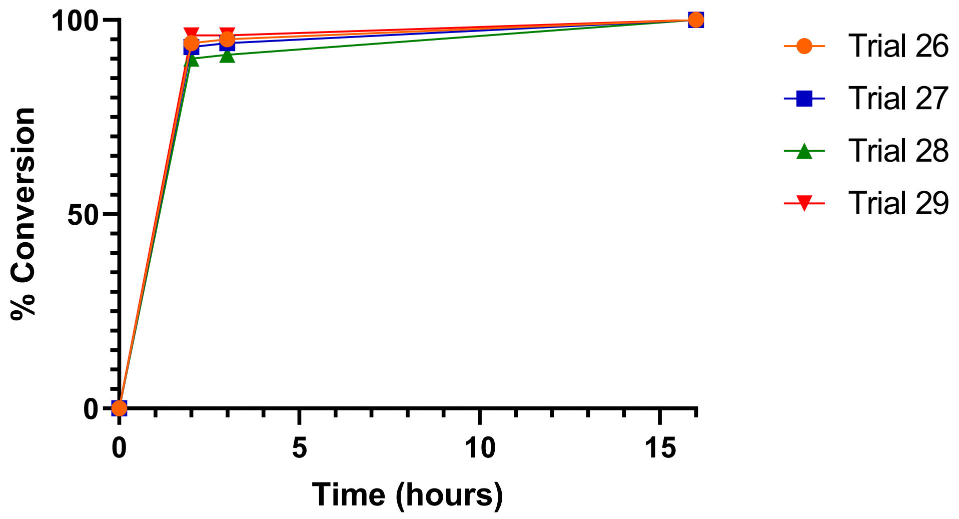

Figure S13. H-UAF-OMe TEMPO trapping optimization – compatibility of a range of organic solvents as the co-solvent in the reaction.

Temperature – Attempts to increase reaction rate

Trial 34 did not require doping to reach completion in a similar time-frame relative to the 'doped' comparison (trial 32) – using a third of the amount of TCEP and TEMPO.

Table S10. TEMPO conjugation optimization by varying the reaction temperature.

Trial	Peptide/ mM	TCEP eq.	TEMPO eq.	Doping	Temp/ °C	1 hr %conv.	2 hrs %conv.	4 hrs %conv.
30	2.5	50	5	2	21	31	29	60
31	2.5	50	5	2	37	53	83	100
32	2.5	50	5	2	50	87	100	100
33	2.5	50	5	2	75	82	81	82
34	2.5	50	5	0	50	56	91	100

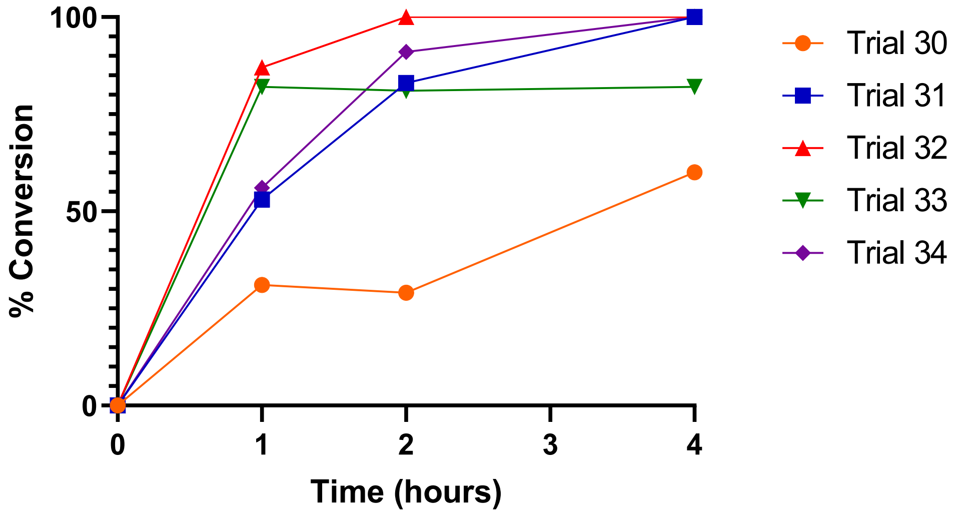

Figure S14. H-UAF-OMe TEMPO trapping optimization – temperature alteration to increase reaction rate.

Initiator Comparison – No doping, 50 °C

Table S11. TEMPO conjugation optimization by trialing a range of initiators.

Trial	Peptide/ mM	TCEP eq.	TEMPO eq.	Initiator (eq.)	30 min %conv.	1 hr %conv.	2 hrs %conv.
35	2.5	50	5	$Cu(OAc)_2$ (2)	45	100	100
36	2.5	50	5	VA-044 (2)	54	100	100
37	2.5	50	5	$Mn(OAc)_3$ (2)	100	100	100
38	2.5	50	5	Eosin Y (0.05)	39	80	100
39	2.5	50	5	None	38	68	91

$Mn(OAc)_3$ showed most promising result – completion in 30 minutes

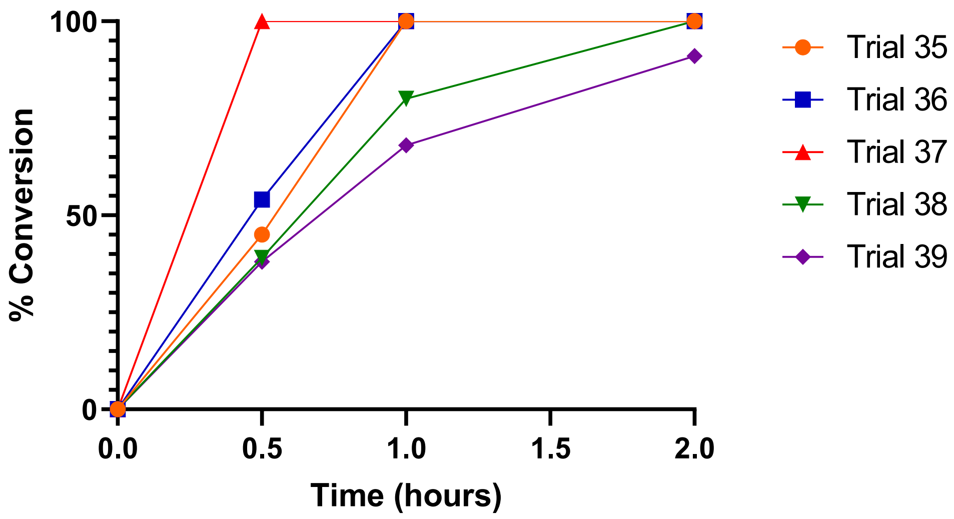

Figure S15. H-UAF-OMe TEMPO trapping optimization – inclusion of a range of potential initiators in an attempt to further improve reaction rate.

Mn trials – Attempts for highest reaction rates and lowest equivalences of reagents – No doping, 50 °C

Table S12. Mn accelerated conjugation optimization.

Trial	Peptide/ mM	TCEP eq.	TEMPO eq.	$Mn(OAc)_3$ eq.	Temp °C	1 hr %conv.	1.5 hrs %conv.	2 hrs %conv.
40	2.5	10	2	0	50	21	83	100
41	2.5	10	2	2	50	47	100	100
42	2.5	25	4	2	50	76	100	100
43	2.5	25	4	2	37	73	93	100
44	2.5	50	2	4	50	100	100	100

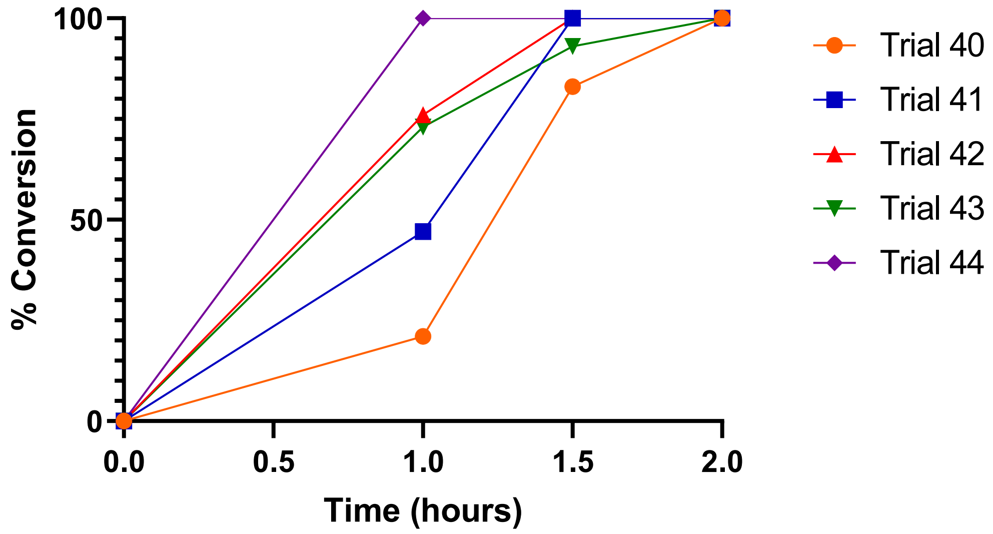

Figure S16. H-UAF-OMe TEMPO trapping optimization – a range of reactions with Mn initiation in attempt to lower the equivalents of TEMPO required.

Fmoc-SPPS and analytical data for model peptides

Ac-CWHISKEY-NH$_2$ (15)

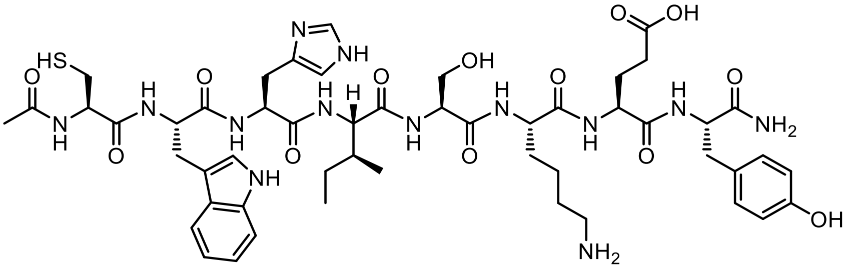

Synthesized using general automated synthesizer procedure with microwave assistance on rink amide resin (0.2 mmol). The crude peptide was purified by preparative RP-HPLC (15-80% B over 30 minutes) and lyophilized to produce the desired peptide (89 mg, 0.08 mmol, 40% yield).

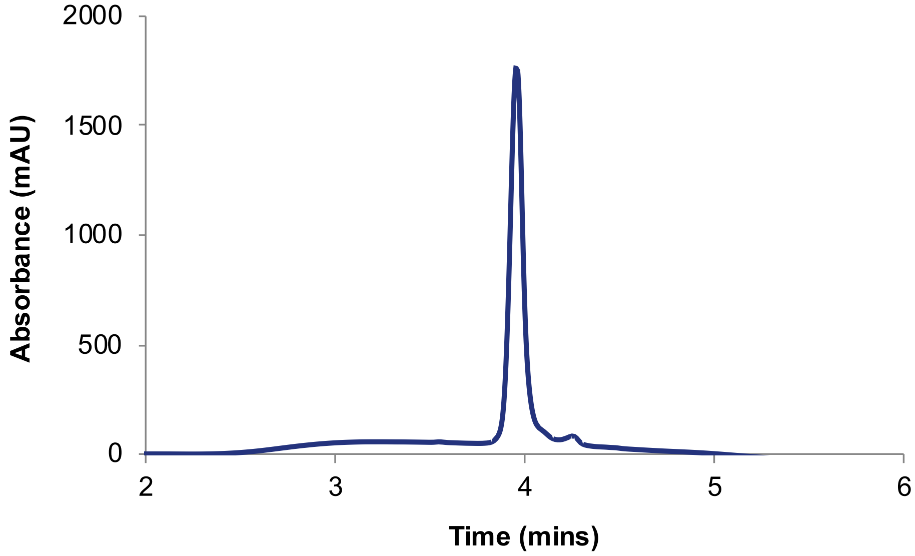

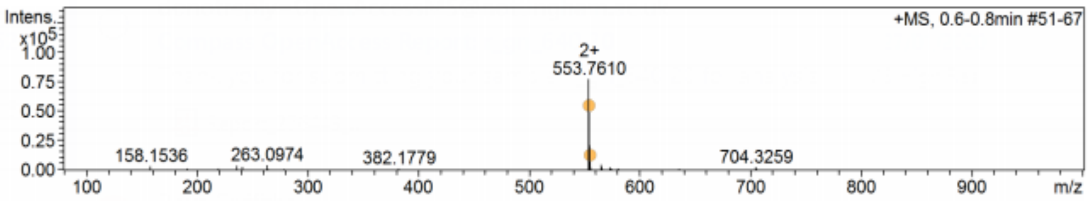

Figure S17. Analytical HPLC trace of purified Ac-CWHISKEY-NH$_2$ (**15**). Analytical gradient 10-100% B over 5 min, 210 nm. Calculated Mass $[M+2H]^{2+}$: 553.7586. Observed Mass $[M+2H]^{2+}$: 553.7610.

H-UWIMKY-NH₂ (21)

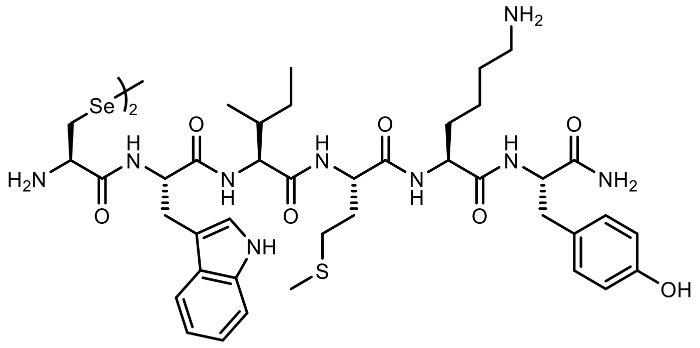

Synthesized using general automated synthesizer procedure with microwave assistance on Rink amide resin (0.313 g, 0.20 mmol). $(Boc\text{-}Sec\text{-}OH)_2$ was coupled using the method outlined in the general methods section. The crude peptide was purified using preparative RP-HPLC (15 to 80%B over 30 minutes) and lyophilized to produce the desired peptide (31 mg, 0.03 mmol, 17% yield).

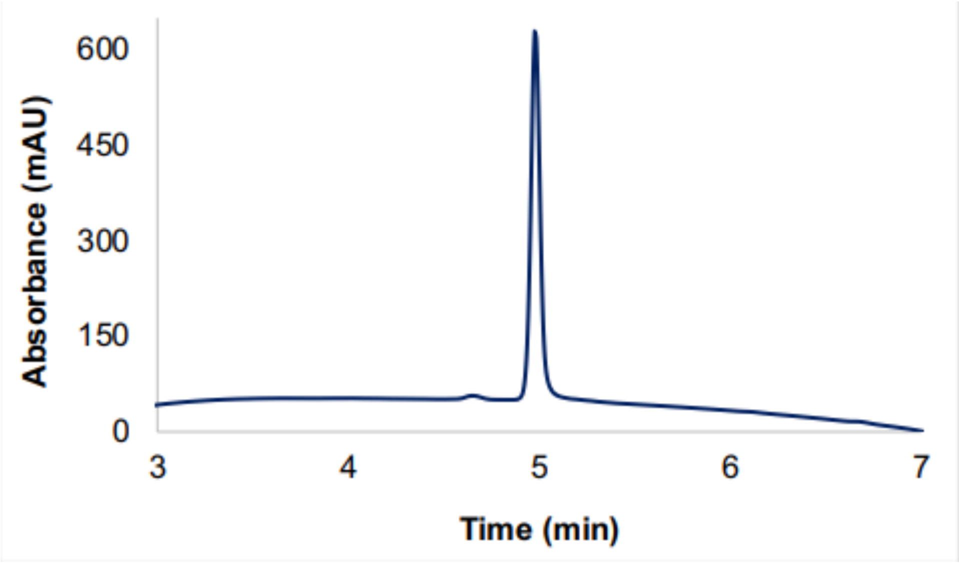

+MS, 0.6-0.8min #51-67

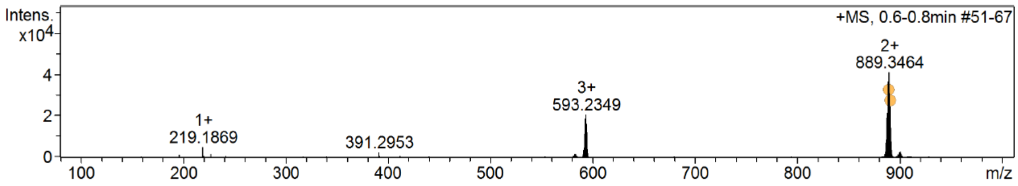

Figure S18. Analytical HPLC trace of pure H-UWIMKY peptide (**21**). Analytical gradient 10-70% B over 10 min, 210 nm. HRMS Calculated $[M+2H]^{2+}$ = 889.3423, $[M+3H]^{3+}$: 593.2308. Observed $[M+2H]^{2+}$ = 889.3464, $[M+3H]^{3+}$: 593.2349.

Ac-YEPLA-SePh (23)

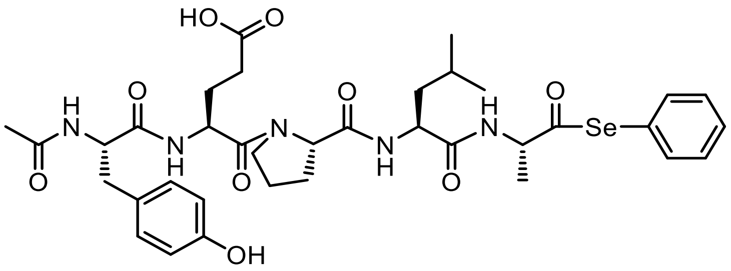

Synthesized using general automated synthesizer procedure with microwave assistance on 2-chlorotrityl chloride resin (0.2 mmol). The selenoester was formed following the method in the general methods section. The crude selenoester was purified by preparative RP-HPLC (30-100% B over 30 minutes) and lyophilized to produce the desired peptide (25 mg, 0.032 mmol, 18% yield).

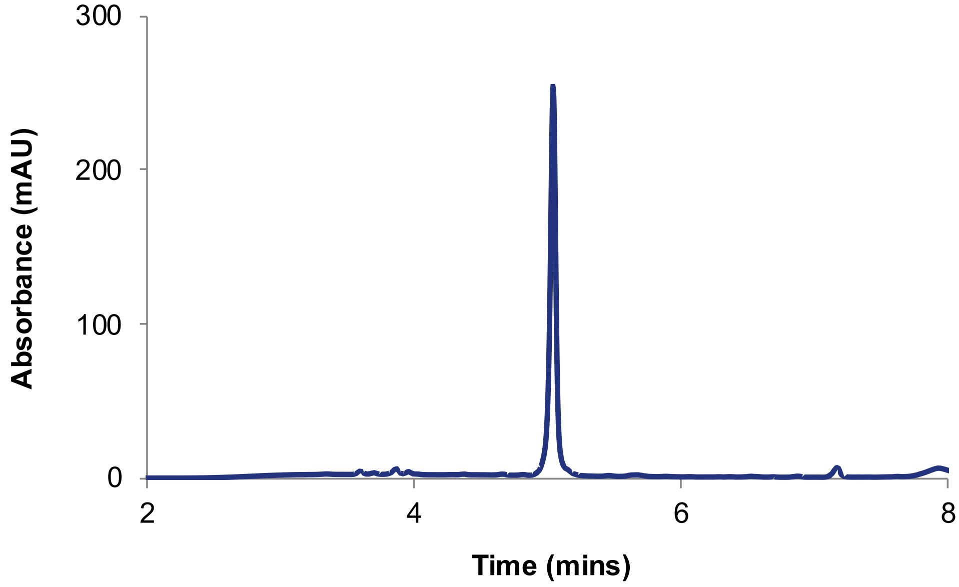

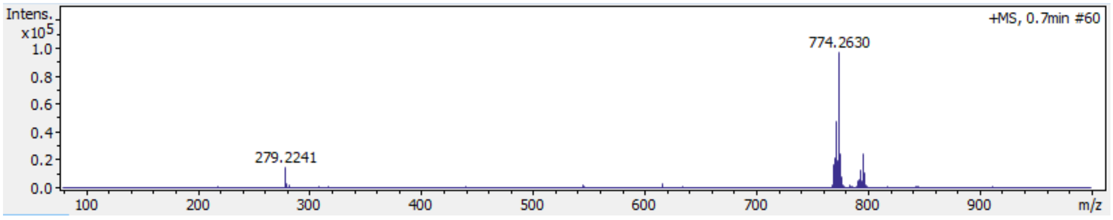

Figure S19. Analytical HPLC trace of purified Ac-YEPLA-SePh (**23**). Analytical gradient 10-100% B over 5 min, 280 nm. Calculated Mass $[M+H]^+$: 774.2617. Observed Mass $[M+H]^+$: 774.2634.

H-UHISKY-NH$_2$ (24)

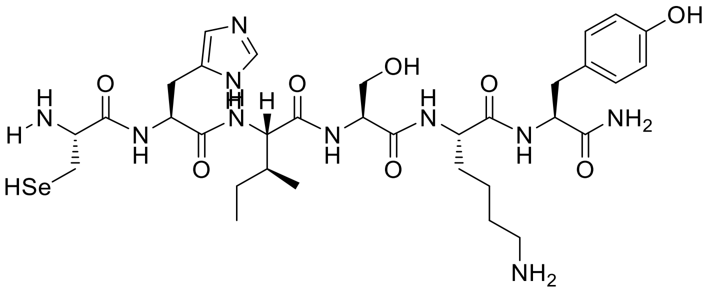

Synthesized using the general automated synthesizer procedure with microwave assistance on Rink amide resin (0.313 g, 0.2 mmol) was used and automatically synthesized up to Sec. (Boc-Sec-OH)$_2$ was coupled using the method outlined in the general methods section. The crude peptide was purified using preparative RP-HPLC (5 to 40%B over 30 minutes) and lyophilized to produce the desired peptide (26 mg, 0.033 mmol, 17% yield).

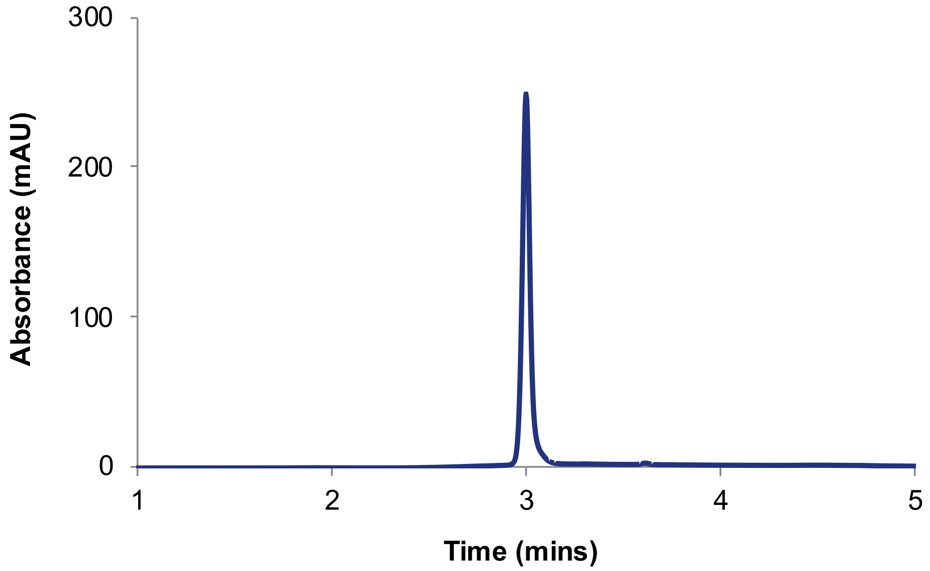

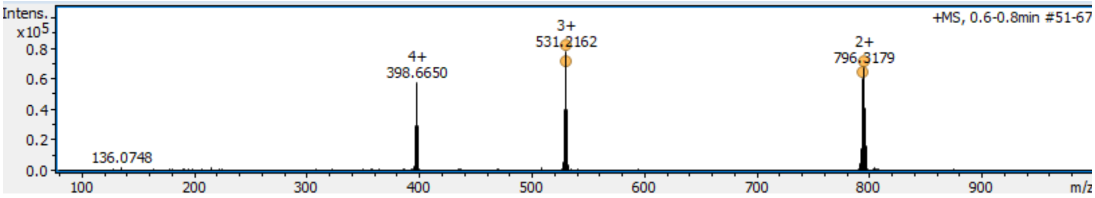

Figure S20. Analytical HPLC trace of purified H-UHISKY-NH$_2$ (**24**). Analytical gradient 10-70% B over 5 min, 210 nm. Calculated Mass $[M+2H]^{2+}$: 796.3135, $[M+3H]^{3+}$: 531.2137, $[M+4H]^{4+}$: 398.6622. Observed Mass $[M+2H]^{2+}$: 796.3166, $[M+3H]^{3+}$: 531.2162, $[M+4H]^{4+}$: 398.6650.

H-UHISCY-NH₂ (25)

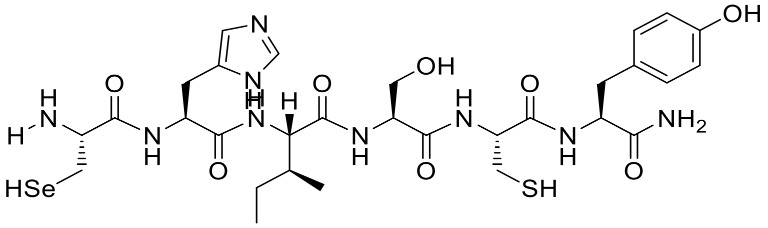

Synthesized using general automated synthesizer procedure with microwave assistance on Rink amide resin (0.313 g, 0.20 mmol). $(Boc\text{-}Sec\text{-}OH)_2$ was coupled using the method outlined in the general methods section. The crude peptide was purified using preparative RP-HPLC (5 to 40%B over 30 minutes) and lyophilized to produce the desired peptide (26 mg, 0.034 mmol, 17% yield).

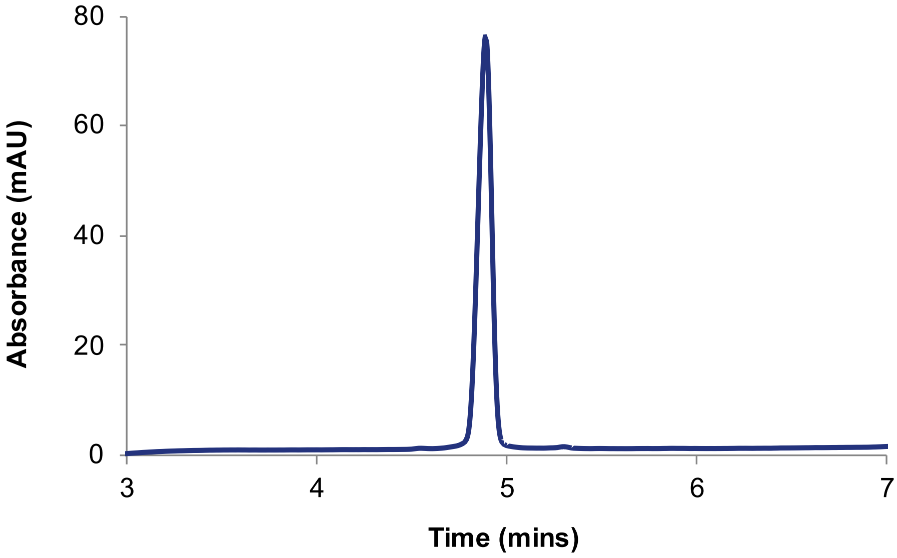

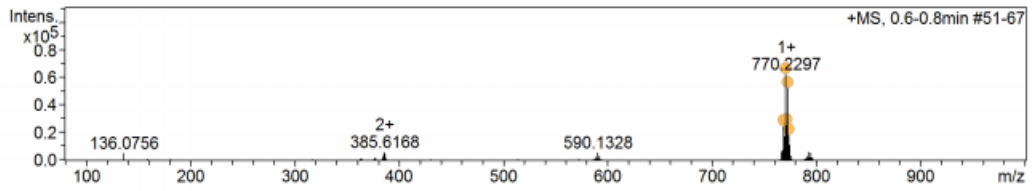

Figure S21. Analytical HPLC trace of purified H-UHISCY-NH_2 (**25**). Analytical gradient 2-40% B over 5 min, 210 nm. Calculated Mass $[M+H]^+$: 770.2199. Observed Mass $[M+H]^+$: 770.2297.

H-CHISKY-NH_2 (26)

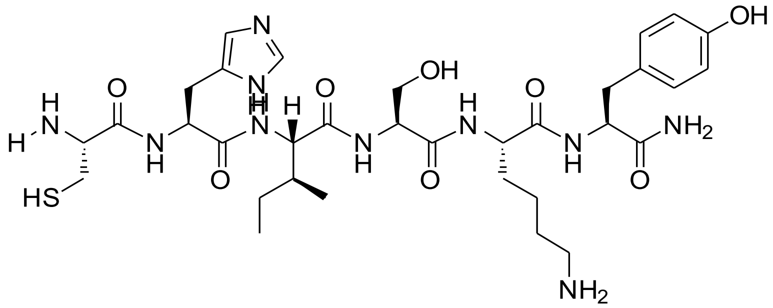

Synthesized using general automated synthesizer procedure with microwave assistance on Rink amide resin (0.313 g, 0.20 mmol). The crude peptide was purified using preparative RP-HPLC (5 to 40%B over 30 minutes) and lyophilized to produce the desired peptide (45 mg, 0.060 mmol, 30% yield).

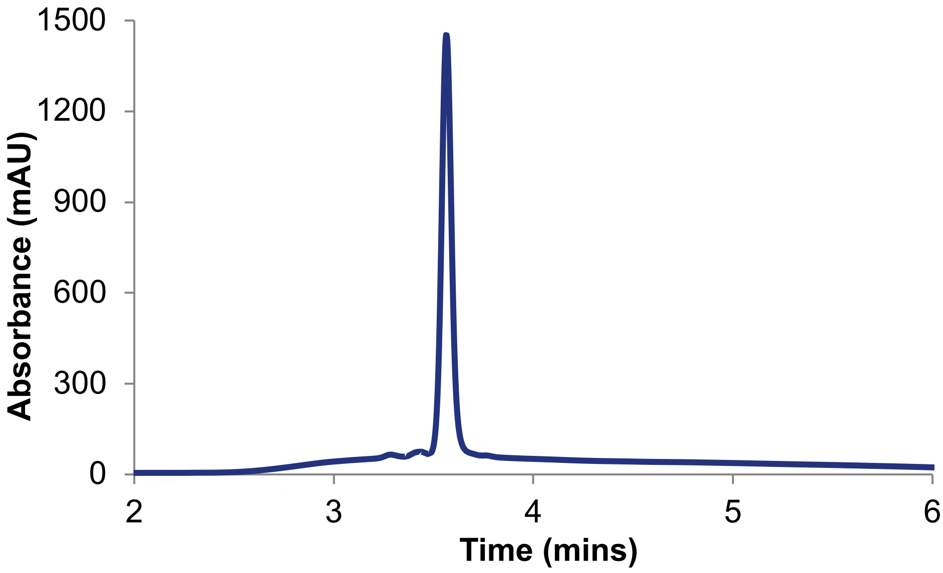

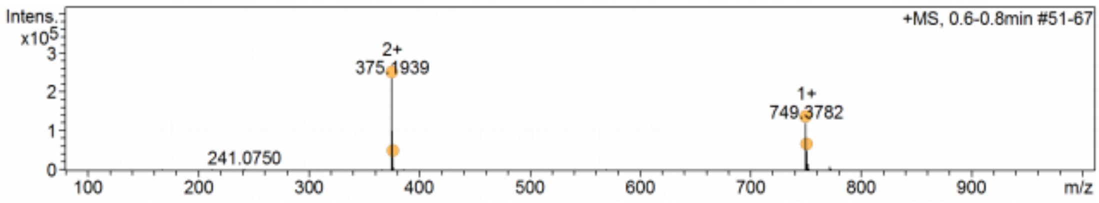

Figure S22. Analytical HPLC trace of purified H-CHISKY-NH_2 (**26**). Analytical gradient 5-60% B over 5 min, 210 nm. Calculated Mass $[M+H]^+$: 749.3769, $[M+2H]^{2+}$: 375.1924. Observed Mass $[M+H]^+$: 749.3782, $[M+2H]^{2+}$: 375.1939.

ZEGFR-1 (42)

H-VDNKFNKEMWAAWEEIRNLPNLNGWQMT-SePh

Synthesized using general automated synthesizer procedure with microwave assistance on 2-chlorotrityl chloride resin (0.2 mmol calculated loading). The selenoester was formed using the general procedure in the general methods section. The crude peptide was purified by preparative RP-HPLC (20-80% B over 30 minutes) and lyophilized to produce the desired peptide (40 mg, 0.011 mmol, 6% yield).

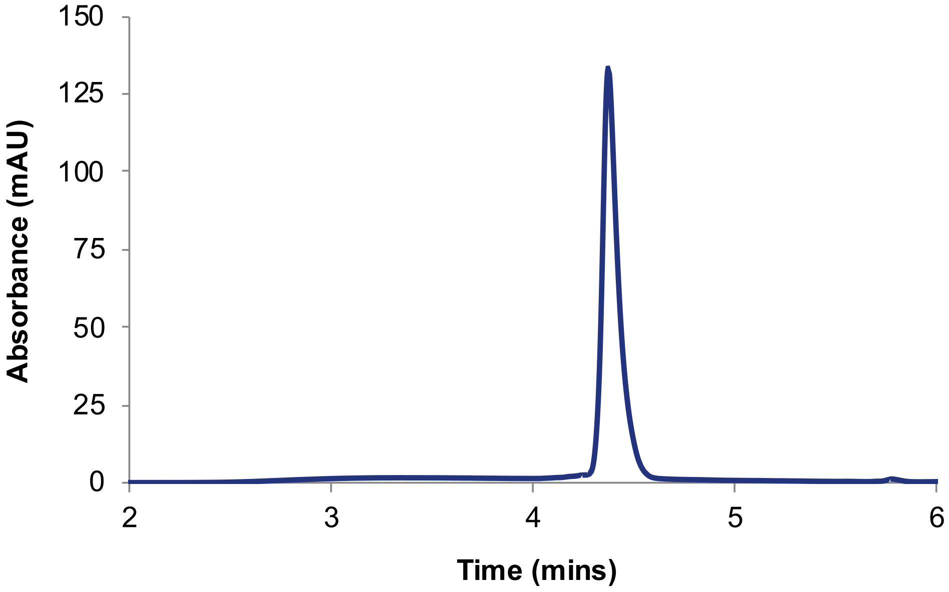

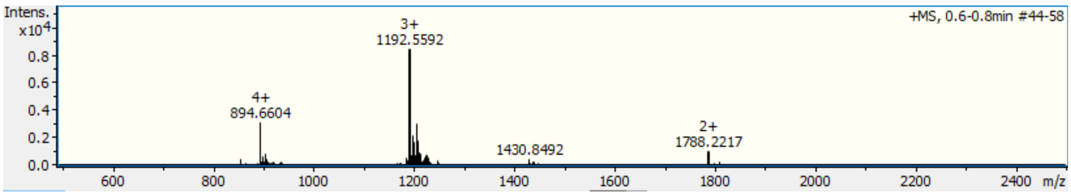

Figure S23. Analytical HPLC trace of ZEGFR-1 selenoester (**42**). Analytical gradient 10-100% B over 5 min, 280 nm. Calculated Mass $[M+2H]^{2+}$: 1787.8010, $[M+3H]^{3+}$: 1192.2033, $[M+4H]^{4+}$: 894.4044. Observed Mass $[M+2H]^{2+}$: 1788.2217, $[M+3H]^{3+}$: 1192.5592, $[M+4H]^{4+}$: 894.6604.

ZEGFR-2 (43)

H-UFIASLVDDPSQSANLLAEAKKLNDAQAPK-OH

Synthesized using general automated synthesizer procedure with microwave assistance on 2-chlorotrityl chloride resin (0.2 mmol calculated loading). (Boc-Sec-OH)$_2$ was coupled using the method outlined in the general methods section. The crude peptide was purified by preparative RP-HPLC (20-70% B over 30 minutes) and lyophilized to produce the desired peptide (78 mg, 0.024 mmol, 12% yield).

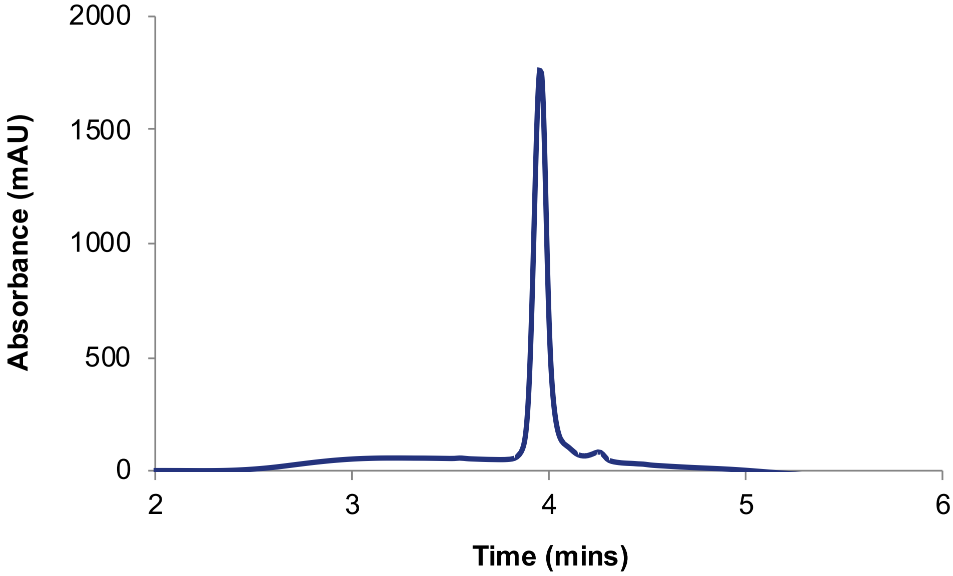

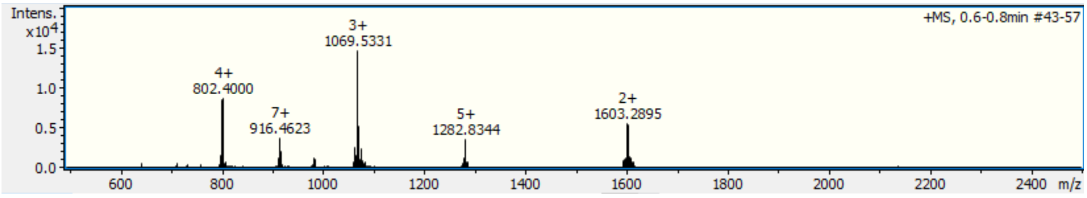

Figure S24. Analytical HPLC trace of ZEGFR-2 (**43**). Analytical gradient 10-100% B over 5 min, 210 nm. MS shows mixture of selenol and diselenide. Calculated Mass $[M+2H]^{2+}$: 1603.2863, $[M+3H]^{3+}$: 1069.1935, $[M+4H]^{4+}$: 802.1471. Observed Mass $[M+2H]^{2+}$: 1603.2895, $[M+3H]^{3+}$: 1069.5331, $[M+4H]^{4+}$: 802.4000.

Ac-AHISKY-OH (55)

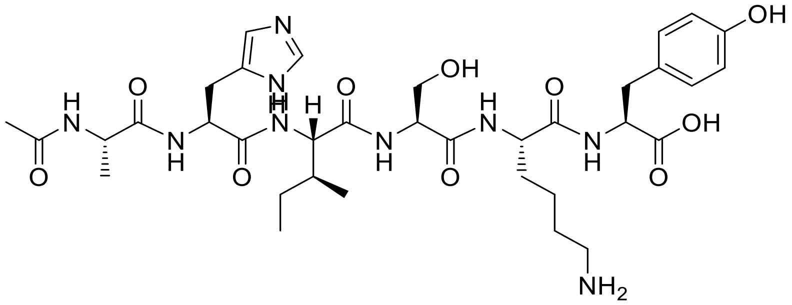

Synthesized using the general manual peptide synthesis procedure on 2-chlorotrityl chloride resin (0.07 mmol, 0.2 g). The crude peptide was purified by preparative RP-HPLC (5-40% B over 30 minutes 6 ml/min) and lyophilized to produce the desired peptide (29 mg, 0.038 mmol, 54% yield).

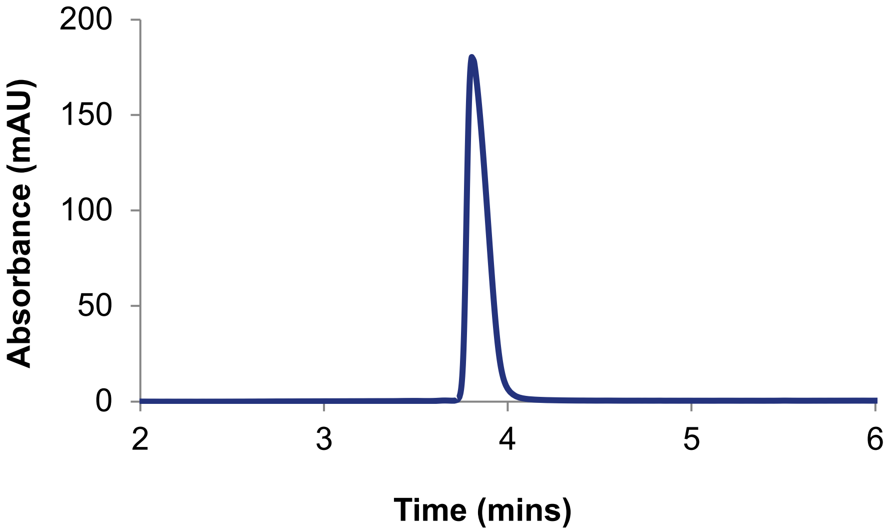

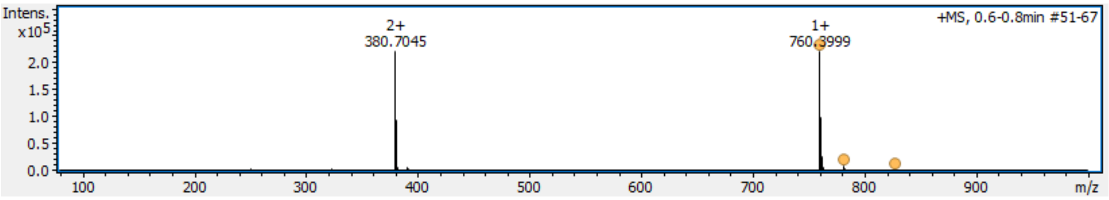

Figure S25. Analytical HPLC trace of purified Ac-AHISKY-OH (**55**). Analytical gradient 10-25% B over 5 min, 210 nm. Calculated Mass $[M+H]^+$: 760.3994, $[M+2H]^{2+}$: 380.7036. Observed Mass $[M+H]^+$: 760.3999, $[M+2H]^{2+}$: 380.7045.

Ac-UHISKY-OH (56)

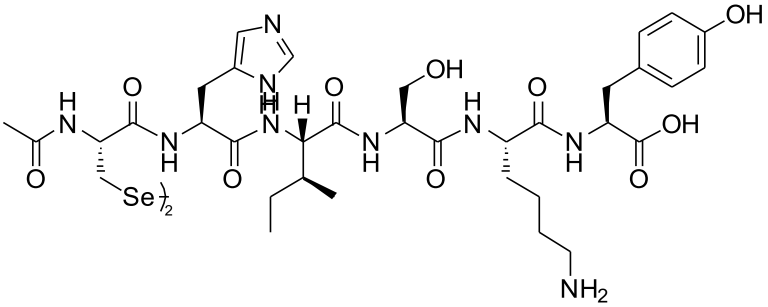

Synthesized using the general manual peptide synthesis procedure on 2-chlorotrityl chloride resin (0.106 mmol, 0.3 g). $(Boc\text{-}Sec\text{-}OH)_2$ was coupled using the method outlined in the general methods section. The crude peptide was purified by preparative RP-HPLC (5-40% B over 30 minutes) and lyophilized to produce the desired peptide (41 mg, 0.049 mmol, 46% yield).

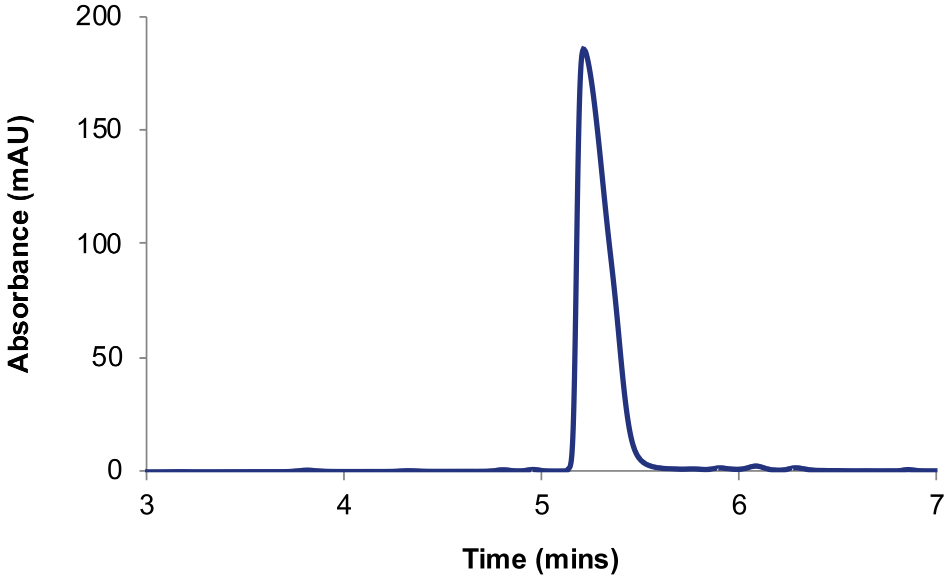

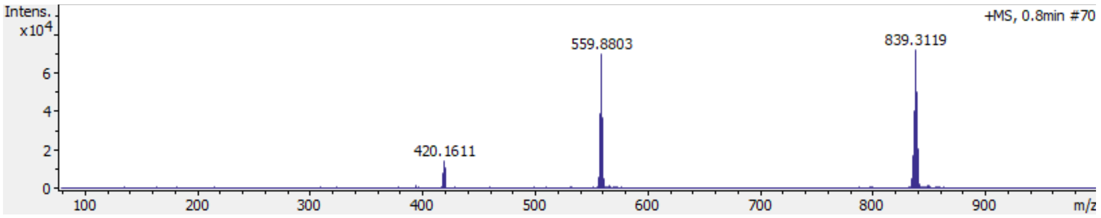

Figure S26. Analytical HPLC trace of purified Ac-UHISKY-OH (**56**). Analytical gradient 10-25% B over 5 min, 210 nm. Calculated Mass $[M+2H]^{2+}$: 839.3081, $[M+3H]^{3+}$: 559.8746. Observed Mass $[M+2H]^{2+}$: 839.3119, $[M+3H]^{3+}$: 559.8803.

Ac-CHISKY-OH (57)

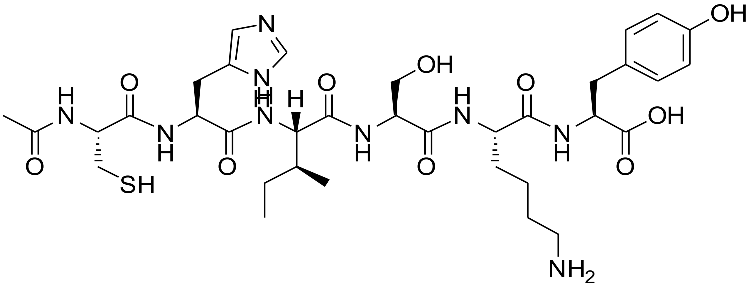

Synthesized using the general manual peptide synthesis procedure on 2-chlorotrityl chloride resin (0.106 mmol, 0.3 g). The crude peptide was purified by preparative RP-HPLC (5-40% B over 30 minutes) and lyophilized to produce the desired peptide (48 mg, 0.061 mmol, 58% yield).

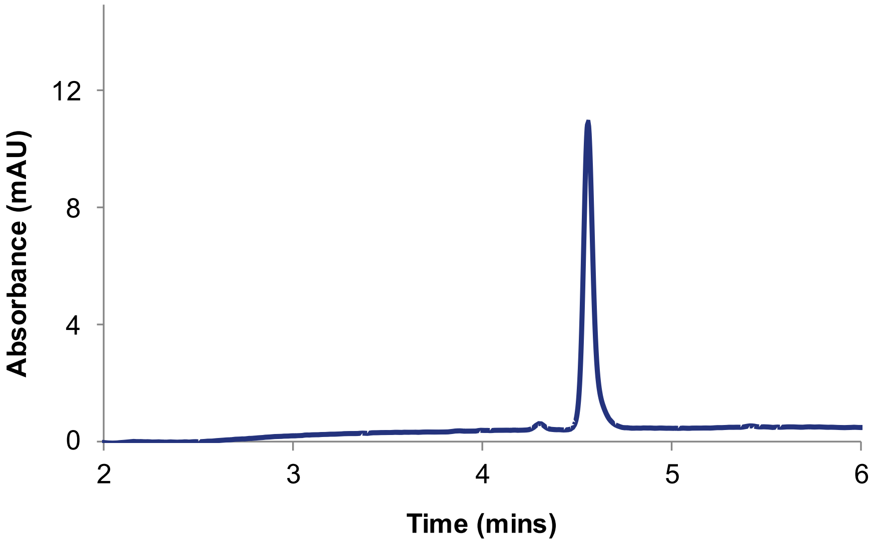

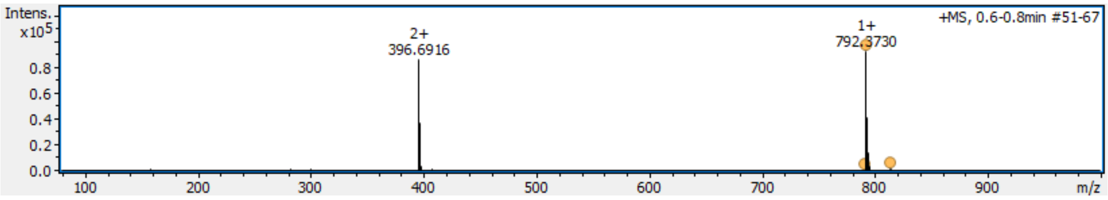

Figure S27. Analytical HPLC trace of purified Ac-CHISKY-OH (**57**). Analytical gradient 10-25% B over 5 min, 210 nm. Calculated Mass $[M+H]^{+}$: 792.3714, $[M+2H]^{2+}$: 396.6896. Observed Mass $[M+H]^{+}$: 792.3730, $[M+2H]^{2+}$: 386.6916.

Ac-YEPLAUHISKY-NH2 (58)

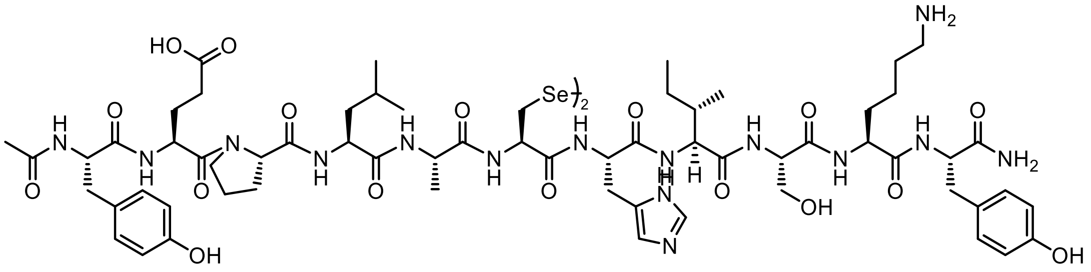

Ligation of Ac-YEPLA-SePh **23** (2.15 mg, 2.78 µmol, 1.05 eq.) and H-UHISKY-NH_2 **24** (2.1 mg, 2.65 µmol (*wrt* selenol), 1 eq.) was performed following the general additive-free ligation procedure in the general methods section. The crude ligation mixture was purified by semi-preparative RP-HPLC (10 to 80%B over 30 minutes) and lyophilized to produce the desired product (3.1 mg, 2.22 µmol, 84% yield).

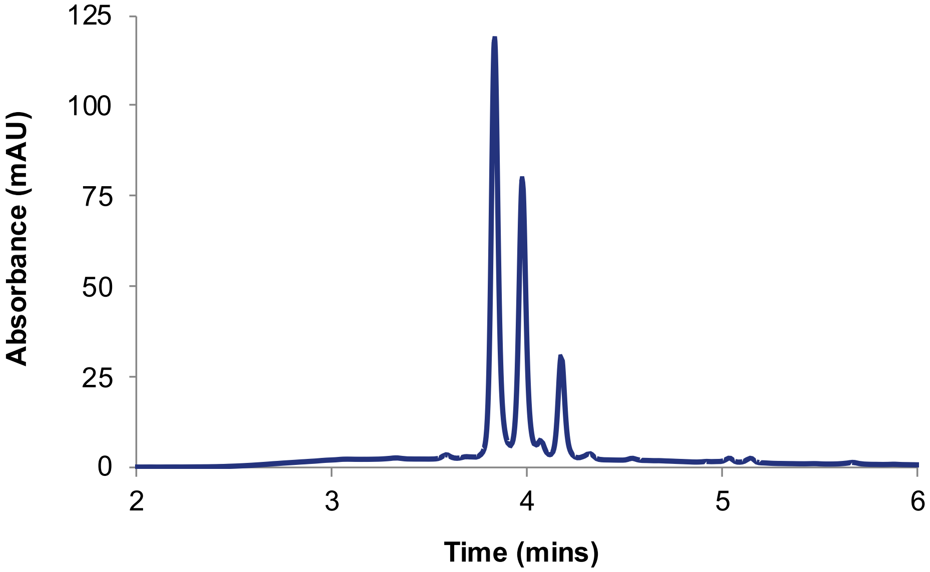

Figure S28. Analytical HPLC trace of crude ligation reaction mixture of (**23**) and (**24**). Analytical gradient 10-100% B over 5 min, 280 nm.

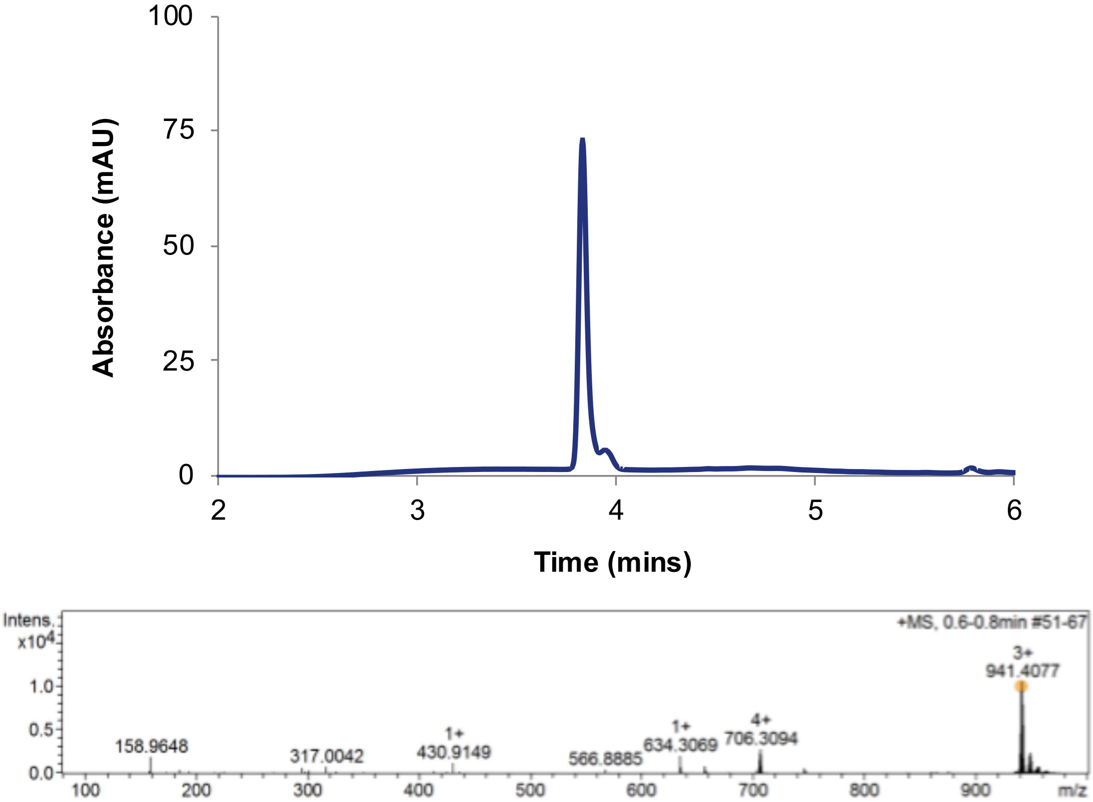

Figure S29. Analytical HPLC trace of pure Ac-YEPLAUHISKY-NH_2 (**58**). Analytical gradient 10-100% B over 5 min, 280 nm. Calculated Mass $[M+3H]^{3+}$: 941.4052, $[M+4H]^{4+}$: 706.3059. Observed Mass $[M+3H]^{3+}$: 941.4077, $[M+4H]^{4+}$: 706.3094.

Conjugation reactions

General protocol A without TEMPO

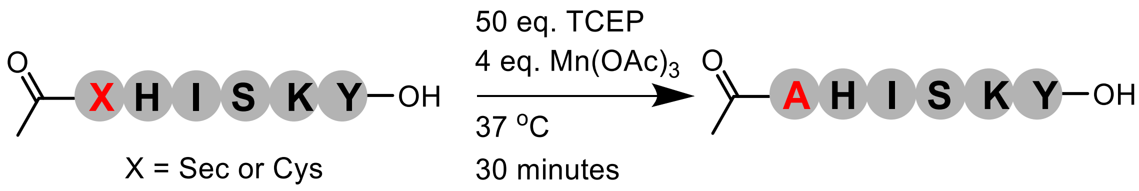

Deselenization of Ac-UHISKY-OH (56) to give Ac-AHISKY-OH (55)

Analytical scale reaction performed using Ac-UHISKY-OH following general conditions A without the addition of TEMPO. Reactions were monitored after being at 37 °C for 30 minutes.

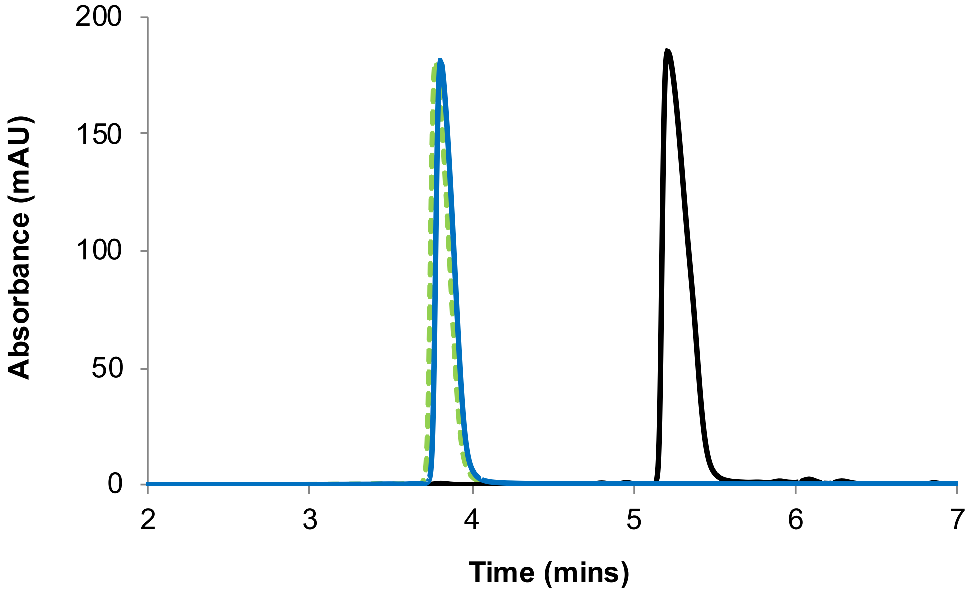

Figure S30. Analytical HPLC trace of Ac-UHISKY-OH deselenization. Analytical gradient 10-25% B over 5 min, 210 nm. Blue – Reaction mixture, Black – Starting material, Green dash – Ac-AHISKY-OH standard.

Desulfurization of Ac-CHISKY-OH (57) to give Ac-AHISKY-OH (55)

Analytical scale reaction performed using Ac-CHISKY-OH following general conditions A without the addition of TEMPO. Reactions were monitored after being at 37 ^{o}C for 30 minutes.

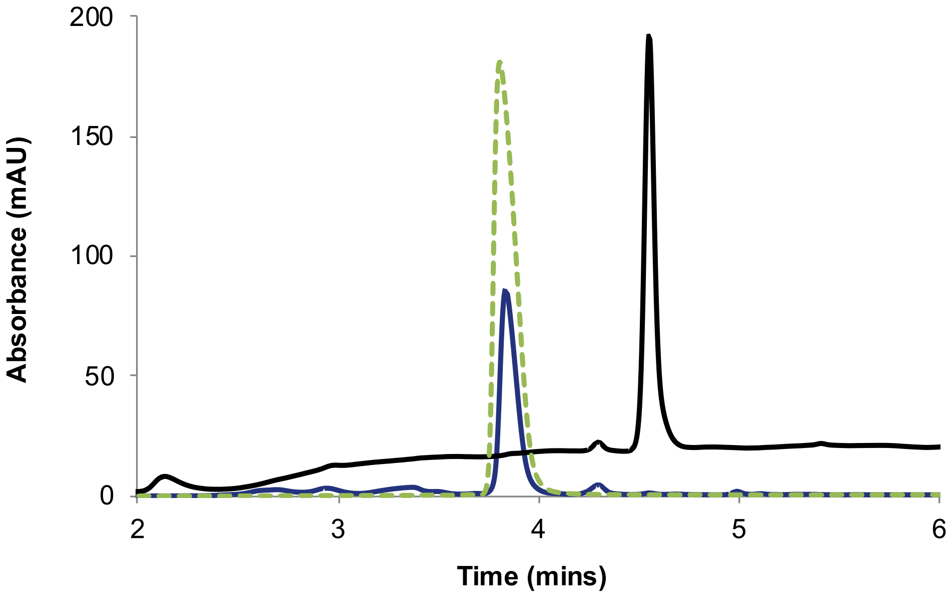

Figure S31. Analytical HPLC trace of Ac-CHISKY-OH desulfurization (**55**). Analytical gradient 10-25% B over 5 min, 210 nm. Blue – Reaction mixture, Black – Starting material, Green dash – Ac-AHISKY-OH standard.

H-UAF-OMe (1a) Bioconjugation (2a, 8 – 12)

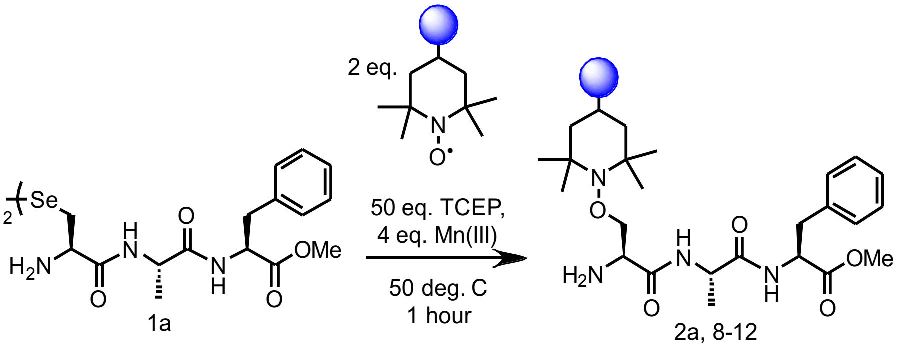

H-UAF-OMe modified with TEMPO (2a)

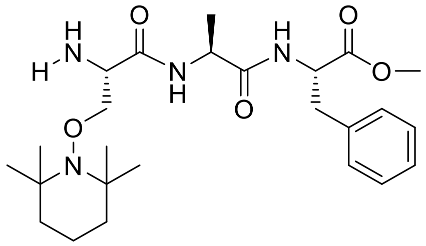

Synthesized using general protocol A using H-UAF-OMe (**1a**) (8 mg, 0.02 mmol). Purified by preparative RP-HPLC (15 to 80% B over 30 minutes) and lyophilized to produce the desired modified peptide (7.9 mg, 0.0166 mmol, 83% yield). ^{1}H NMR (400 MHz, MeOD) δ 7.34 – 7.18 (m, 5H, ArH), 4.64 (dd, J = 8.8, 5.4 Hz, 1H, CHα), 4.49 (q, J = 7.0 Hz, 1H, CHα), 4.16 – 4.04 (m, 3H, CHα and $CH_{2β}$), 3.71 (s, 3H, OCH_3), 3.08 (qd, J = 56, 13.9, 8 Hz, 2H, $CH_β$), 1.73 – 1.52 (m, 2H, CH_2), 1.54 – 1.39 (m, 4H, CH_2), 1.39 (d, J = 7.0 Hz, 3H), 1.27 – 0.96 (m, 12H, TEMPO CH_3).^{13}C NMR (101 MHz, MeOD) δ 172.6, 171.9, 165.7, 136.7, 128.8, 128.2, 126.6, 73.6, 60.2, 60.1, 54.1, 52.3, 51.3, 48.8, 39.4, 36.8, 31.9, 19.2, 17.6, 16.5.

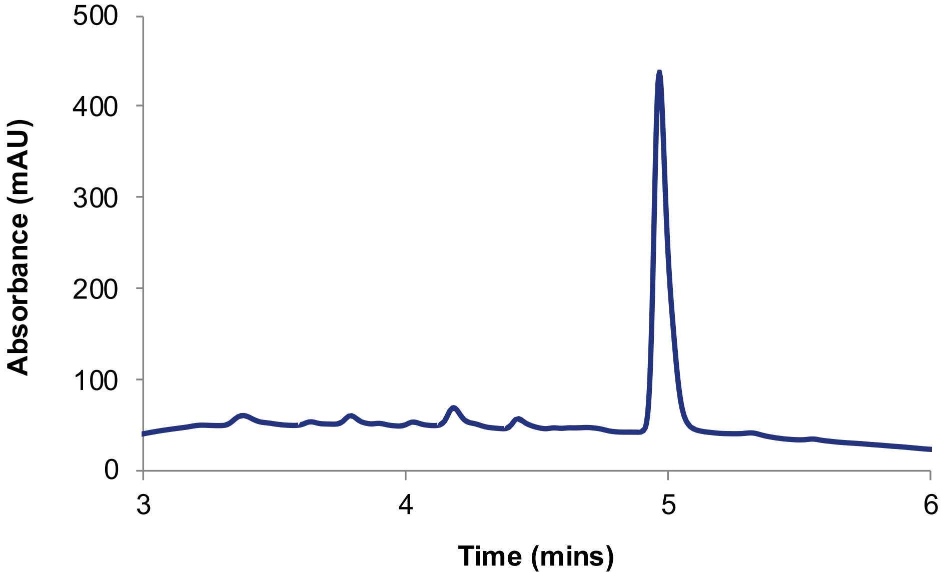

Figure S32. Analytical HPLC trace of 30 minutes crude reaction mixture of (**2a**). Analytical gradient 10-70% B over 5 min, 210 nm.

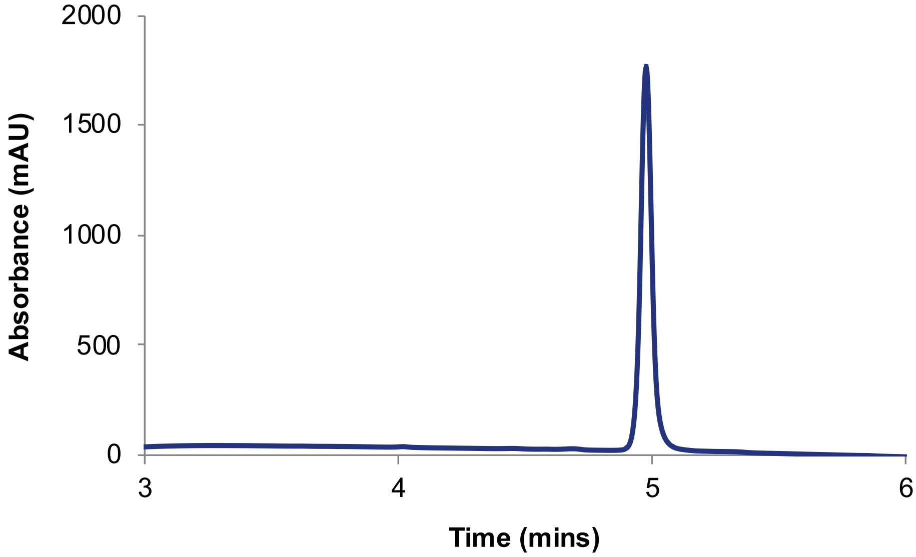

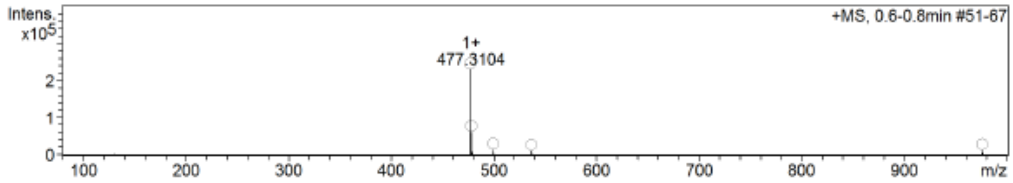

Figure S33. Analytical HPLC trace of pure TEMPO modified H-UAF-OMe (**2a**). Analytical gradient 10-70% B over 5 min, 210 nm. Calculated Mass $[M+H]^+$: 477.3104. Observed Mass $[M+H]^+$: 477.3077.

TEMPO with H-D-UAF (2b)

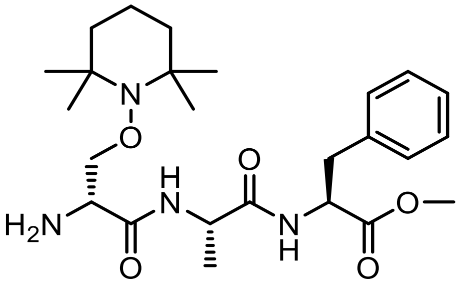

Synthesized using general protocol A using H-D-UAF-OMe (**1b**) (4 mg, 0.01 mmol). Purified by preparative RP-HPLC (15 to 80% B over 30 minutes) and lyophilized to produce the desired modified peptide (2.1 mg, 0.005 mmol, 42%). ^{1}H NMR (400 MHz, $CDCl_3$) δ 7.30-7.19 (m, 5H, ArH), 4.67-4.62 (m, 1H, CH), 4.40 (q, J = 7.2 Hz, 1H, CH), 4.16-4.08 (m, 2H, CH_2), 3.99 (t, J = 5.0 Hz, 1H, CH), 3.68 (s, 3H, CH_3), 3.08 (qd, J = 20.0, 13.9, 8.0 Hz, 2H, CH_2), 1.62 (br s, 2H, CH_2), 1.49-1.47 (m, 4H, CH_2), 1.36 (d, J = 7.0 Hz, 3H, CH_3), 1.17-1.14 (m, 12H, CH_3); ^{13}C NMR (101 MHz, MeOD) δ 173.4, 171.8, 166.1, 136.6, 128.9, 128.1, 126.5, 73.4, 60.3, 60.1, 54.1, 52.1, 51.3, 49.0, 39.4, 36.8, 32.0, 19.2, 16.9, 16.5.

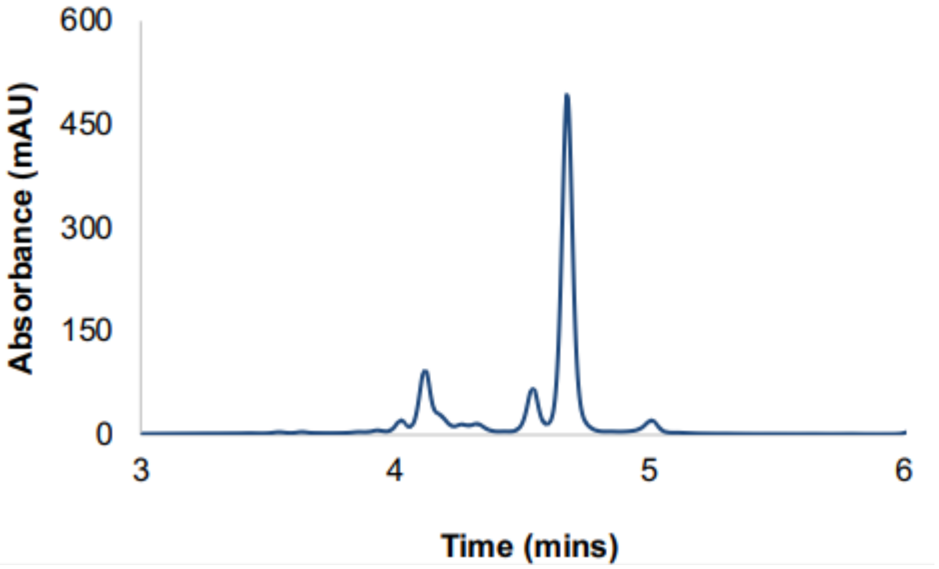

Figure S34. Analytical HPLC trace of 30 minutes crude TEMPO modified H-D-UAF-OMe (**2b**). Analytical gradient 10-70% B over 5 min, 210 nm.

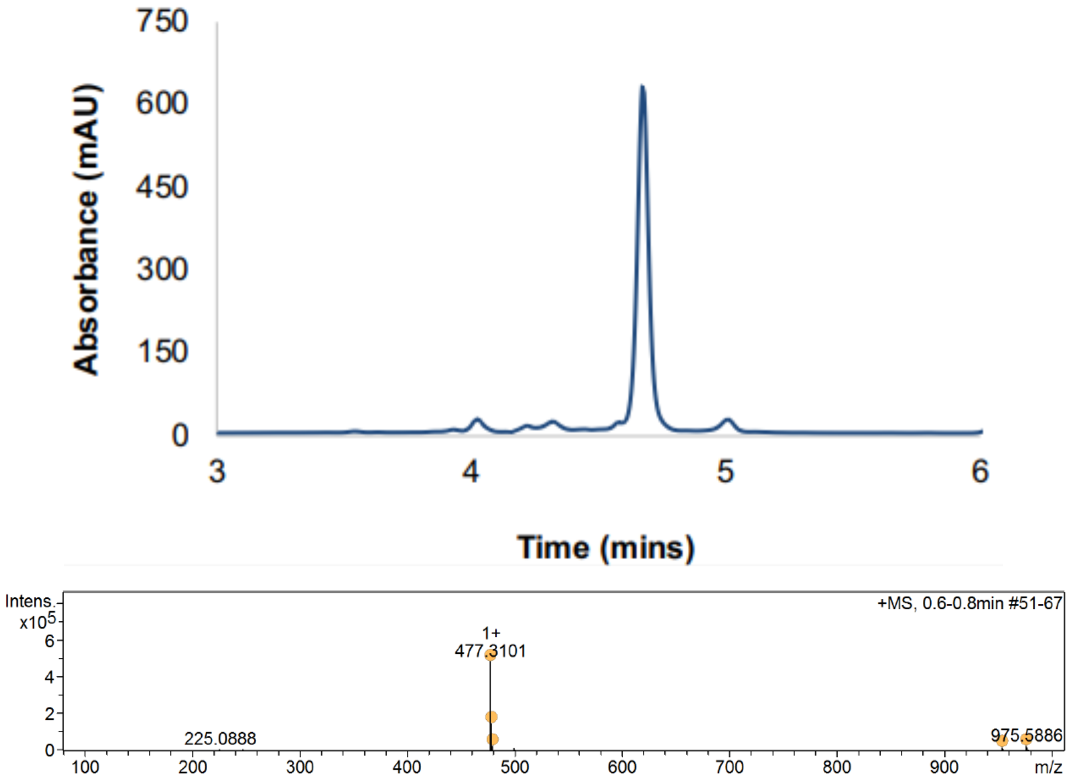

Figure S35. Analytical HPLC trace of pure TEMPO modified H-D-UAF-OMe (**2b**). Analytical gradient 10-70% B over 5 min, 210 nm. HRMS Calculated Mass $[M+H]^+$: 477.2999. Observed Mass $[M+H]^+$: 477.3101.

NMR comparison of product from L and D Sec TEMPO conjugation

Both the L-TEMPO conjugate (**2a**) and D-TEMPO conjugate (**2b**) were synthesized following general protocol A. Briefly, H-UAF-OMe (D or L) dissolved in LB/DMSO (20%) at pH 7 to 7.5 was added TCEP (50 eq.) and TEMPO (2 eq.) followed by $Mn(OAc)_3$ (4 eq.). The pH was checked to be 7 to 7.5 and reaction mixture shaken at 50 °C for 30 minutes, then purified by preparative RP-HPLC.

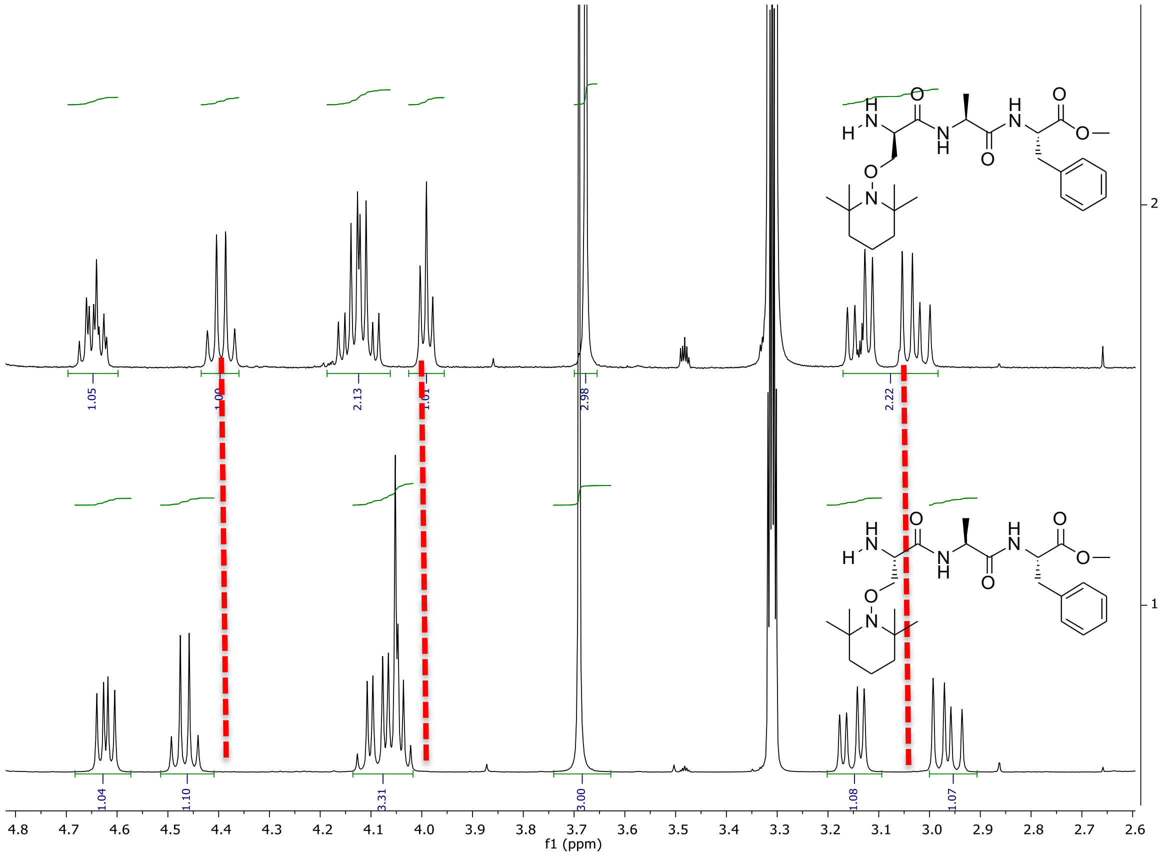

Figure S36. NMR comparison of D-Sec (**2b**, top) and L-Sec (**2a**, bottom) TEMPO conjugated products showing key differences in the NMRs of each product. The absence of highlighted peaks in the L product compared to the D shows racemization does not occur during conjugation.

Tetra-EG-TEMPO (8)

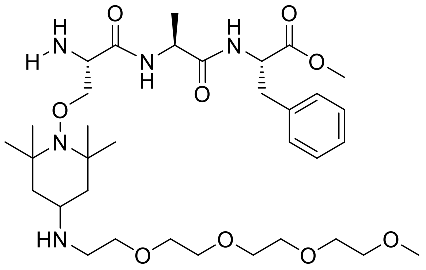

Synthesized using general protocol A using H-UAF-OMe (**1a**) (5 mg, 0.0125 mmol). Purified by preparative RP-HPLC (15 to 80% B over 30 minutes) and lyophilised to produce the desired modified peptide (6.1 mg, 8.95 mmol, 72% yield). ^{1}H NMR (400 MHz, MeOD) δ 7.35 – 7.19 (m, 5H), 4.63 (dd, *J* = 8.7, 5.4 Hz, 1H), 4.50 (q, *J* = 7.0 Hz, 1H), 4.17 – 4.05 (m, 3H), 3.77 (m, 2H), 3.70 (m, 7H), 3.68 – 3.62 (m, 6H), 3.58 – 3.54 (m, 2H), 3.53 – 3.44 (m, 1H), 3.37 (s, 3H), 3.27 (m, 2H), 3.17 (dd, *J* = 14.0, 5.4 Hz, 1H), 2.98 (dd, *J* = 14.0, 4.0 Hz, 1H), 1.96 (m, 2H), 1.56 (q, *J* = 12.0 Hz, 2H), 1.39 (d, *J* = 7.0 Hz, 3H), 1.30 – 1.08 (m, 12H). ^{13}C NMR (100 MHz, MeOD) δ 172.7, 171.8, 165.5, 136.8, 128.9, 128.2, 126.6, 73.9, 71.5, 70.0, 65.7, 59.8, 57.7, 54.1, 52.2, 51.3, 49.8, 44.6, 41.1, 36.8, 31.8, 19.5, 17.6.

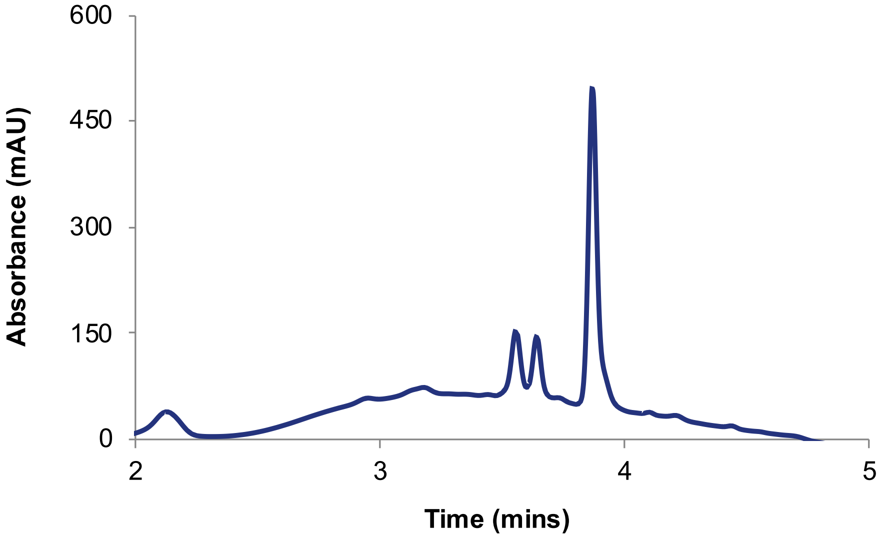

Figure S37. Analytical HPLC trace of 1 hour crude tetra-EG-TEMPO modified H-UAF-OMe (**8**). Analytical gradient 10-100% B over 5 min, 210 nm.

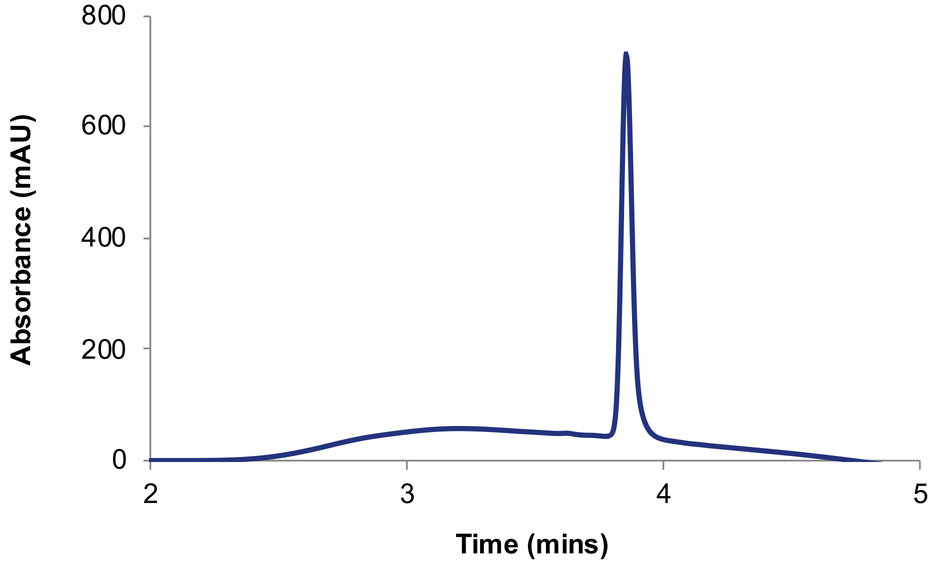

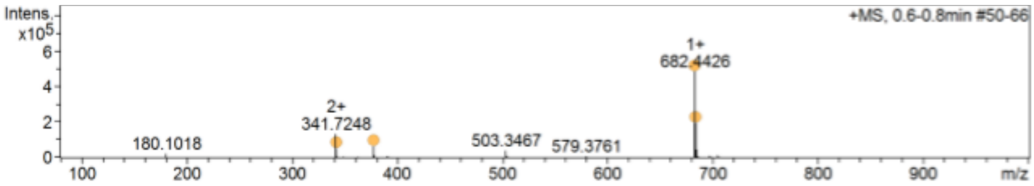

Figure S38. Analytical HPLC trace of pure tetra-EG-TEMPO modified H-UAF-OMe (**8**). Analytical gradient 10-100% B over 5 min, 210 nm. Calculated Mass $[M+H]^+$: 682.4391. Observed Mass $[M+H]^+$: 682.4426.

Propargyl TEMPO Conjugate (9)

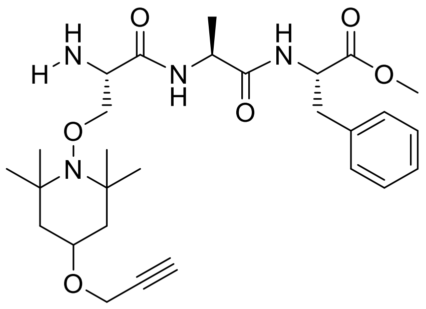

Synthesized using general protocol A using H-UAF-OMe (**1a**) (5 mg, 0.0125 mmol). Purified by preparative RP-HPLC (15 to 80% B over 30 minutes) and lyophilised to produce the desired modified peptide (5.4 mg, 0.01 mmol, 80% yield). ^{1}H NMR (400 MHz, MeOD) δ 7.35 – 7.22 (m, 5H, Ar-H), 4.64 (dd, *J* = 9.0, 5.2 Hz, 1H, CHα), 4.49 (q, *J* = 7.0 Hz, 1H, CHα), 4.20 (d, *J* = 2.4 Hz, 2H, OCH_2), 4.14 – 4.03 (m, 3H, CHα and $CH_{2\beta}$), 3.86 (m, 1H, TEMPO CH), 3.72 (s, 3H, OCH_3), 3.18 (dd, *J* = 14.0, 5.2 Hz, 1H, CH_β), 2.97 (dd, *J* = 14.0, 9.0 Hz, 1H, CH_β), 2.88 (t, *J* = 2.4 Hz, 1H, alkyne CH), 1.87 (m, 2H, CH_2), 1.39 (d, *J* = 7.0 Hz, 3H, Ala CH_3), 1.37 – 1.29 (m, 2H, CH_2), 1.13 (m, 12H, TEMPO CH_3). ^{13}C NMR (101 MHz, MeOD) δ 172.6, 171.9, 165.6, 136.8, 128.9, 128.2, 126.6, 79.6, 74.2, 73.6, 69.0, 60.3, 54.7, 54.1, 52.2, 51.3, 48.8, 44.4, 36.8, 32.1, 20.0, 17.7.

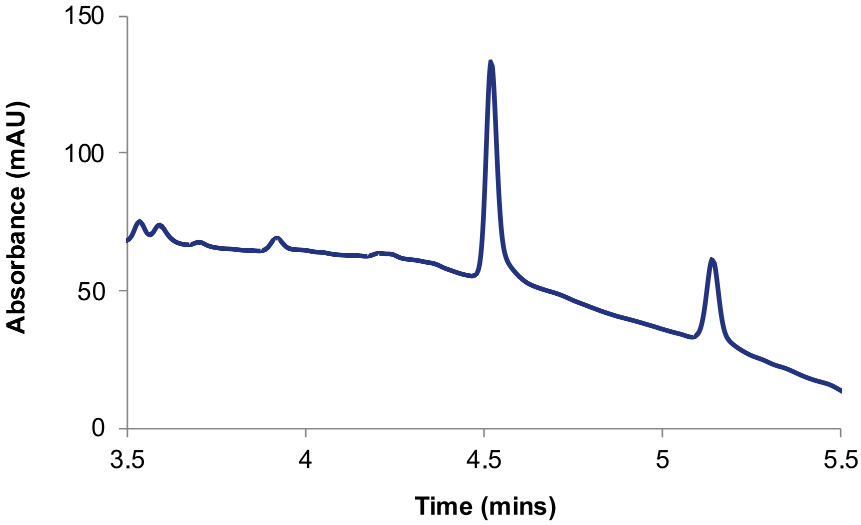

Figure S39. Analytical HPLC trace of 1 hour crude propargyl-TEMPO modified H-UAF-OMe (**9**). Analytical gradient 10-100% B over 5 min, 210 nm.

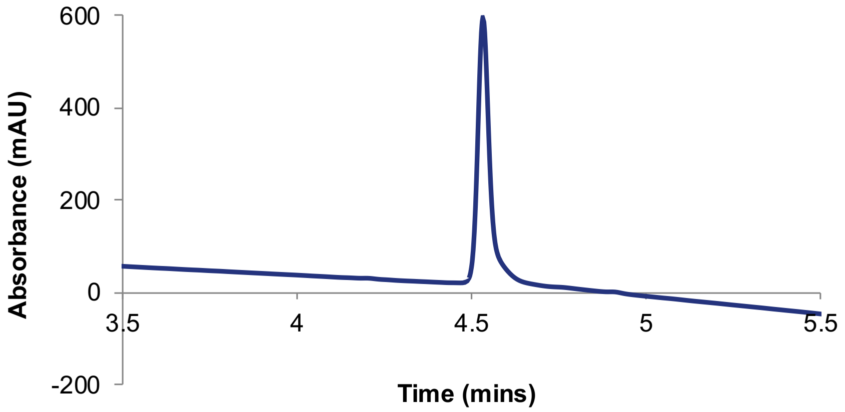

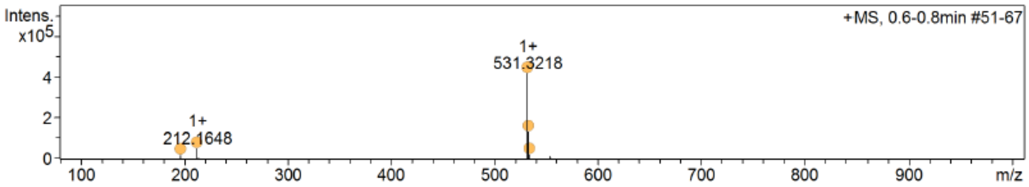

Figure S40. Analytical HPLC trace of pure propargyl-TEMPO modified H-UAF-OMe (**9**). Analytical gradient 10-100% B over 5 min, 210 nm. Calculated Mass $[M+H]^+$: 531.3183. Observed Mass $[M+H]^+$: 531.3218.

FITC-TEMPO Conjugate (10)

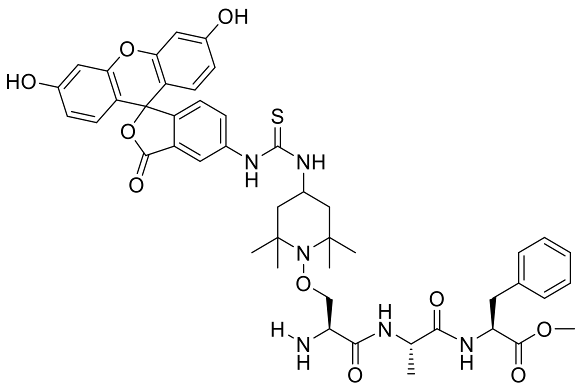

Synthesized using general protocol A using H-UAF-OMe (**1a**) (5 mg, 0.0125 mmol). Purified by preparative RP-HPLC (15 to 80% B over 30 minutes) and lyophilized to produce the desired modified peptide (7.6 mg, 69% yield, 8.62 mmol). ^{1}H NMR (400 MHz, MeOD) δ 8.27 (d, J = 1.6 Hz, 1H), 7.84 (d, J = 4.0 Hz, 1H), 7.38 – 7.17 (m, 6H), 6.88 (m, 4H), 6.70 (m, 2H), 4.65 (m, 1H), 4.51 (q, J = 12.0, 4.0 Hz, 1H), 4.09 (d, J = 5.4 Hz, 3H), 3.72 (s, 3H), 3.19 (dd, J = 14.0, 5.2 Hz, 1H), 2.96 (dd, J = 14.0, 5.1 Hz 1H), 2.68 (s, 1H), 2.01 – 1.90 (m, 2H), 1.66 – 1.44 (m, 2H), 1.40 (d, J = 7.0 Hz, 3H), 1.26 – 1.03 (m, 12H). ^{13}C NMR (101 MHz, MeOD) δ 180.7, 172.7, 171.9, 165.6, 154.1, 141.5, 136.8, 129.7, 128.8, 128.3, 126.8, 125.3, 113.6, 102.1, 73.7, 60.5, 54.2, 52.3, 51.4, 48.8, 45.6, 44.3, 36.8, 31.8, 19.8, 17.7, 15.9.

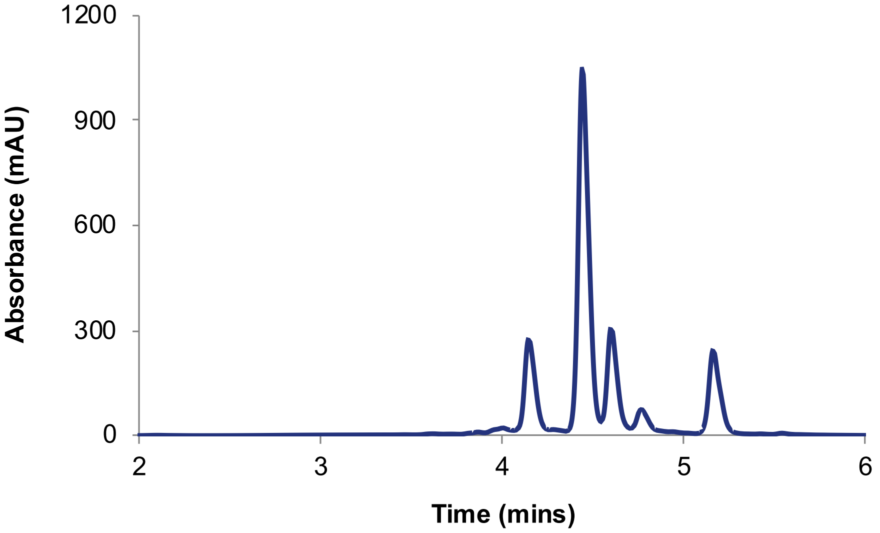

Figure S41. Analytical HPLC trace of 1 hour crude FITC-TEMPO modified H-UAF-OMe (**10**). Analytical gradient 10-100% B over 5 min, 210 nm.

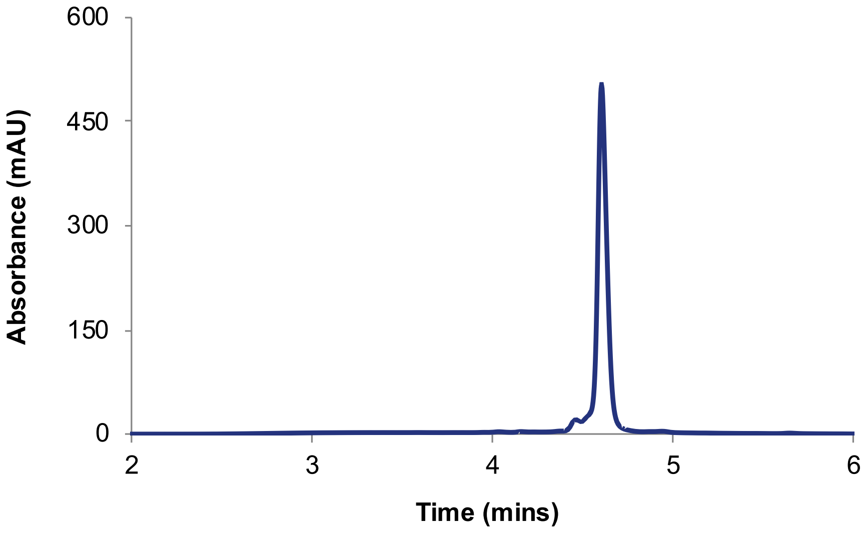

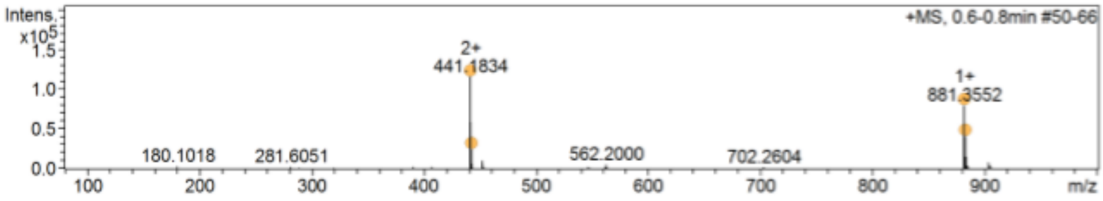

Figure S42. Analytical HPLC trace of pure FITC-TEMPO modified H-UAF-OMe (**10**). Analytical gradient 10-100% B over 5 min, 210 nm. Calculated Mass $[M+H]^+$: 881.3544, $[M+2H]^{2+}$: 441.1811. Observed Mass $[M+H]^+$: 881.3552, $[M+2H]^{2+}$: 441.1834.

Biotin-EDA-TEMPO Conjugate (11)

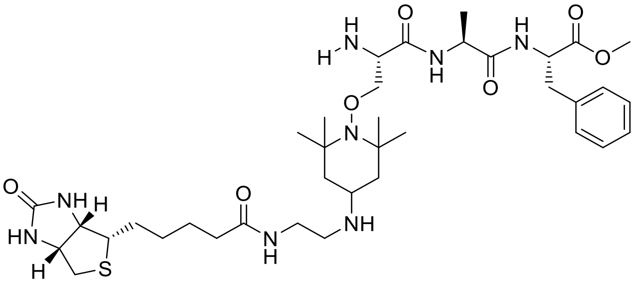

Synthesized using general protocol A using H-UAF-OMe (**1a**) (5 mg, 0.0125 mmol). Purified by preparative HPLC (15 to 80% B over 30 minutes) and lyophilized to produce the desired modified peptide (7.4 mg, 0.0097 mmol, 78% yield).^{1}H NMR (400 MHz, MeOD) δ 8.53 (d, *J* = 7.7 Hz, 1H), 7.39 – 7.18 (m, 5H), 4.63 (m, 1H), 4.52 (m, 2H), 4.32 (m, 1H), 4.19 – 4.02 (m, 3H), 3.71 (s, 3H), 3.55 – 3.44 (m, 3H), 3.28 – 3.13 (m, 4H), 3.03 – 2.90 (m, 2H), 2.78 – 2.65 (m, 1H), 2.30 (t, *J* = 7.3 Hz, 2H), 1.93 (m, 2H), 1.85 – 1.43 (m, 8H), 1.39 (d, *J* = 7.0 Hz, 3H), 1.32 – 1.05 (m, 12H). ^{13}C NMR (101 MHz, MeOD) δ 176.4, 172.7, 171.8, 165.5, 164.7, 136.8, 128.9, 128.2, 126.6, 73.9, 62.0, 60.3, 59.7, 55.6, 54.1, 52.2, 51.3, 49.6, 44.7, 41.1, 39.6, 36.8, 36.1, 35.0, 31.8, 28.4, 28.1, 25.1, 19.4, 17.6.

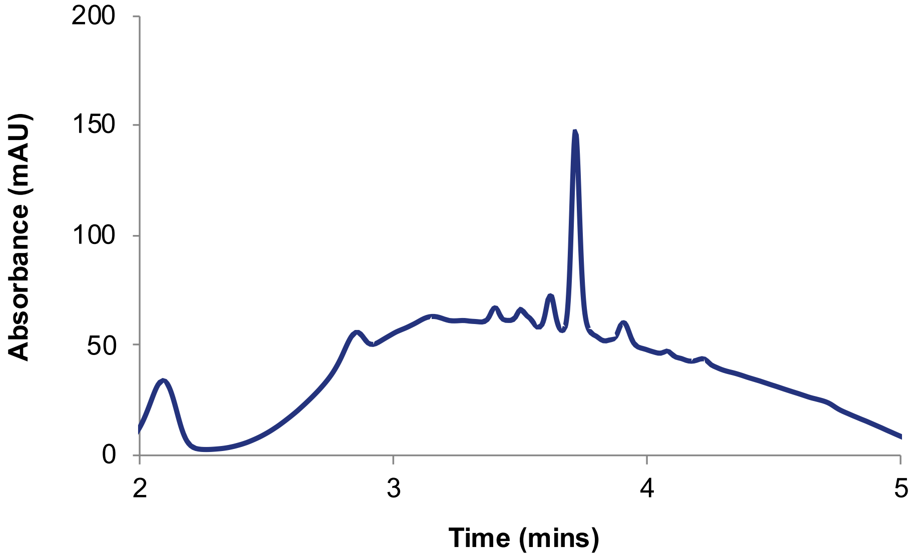

Figure S43. Analytical HPLC trace of 1 hour crude biotin-EDA-TEMPO modified H-UAF-OMe (**11**). Analytical gradient 10-100% B over 5 min, 210 nm.

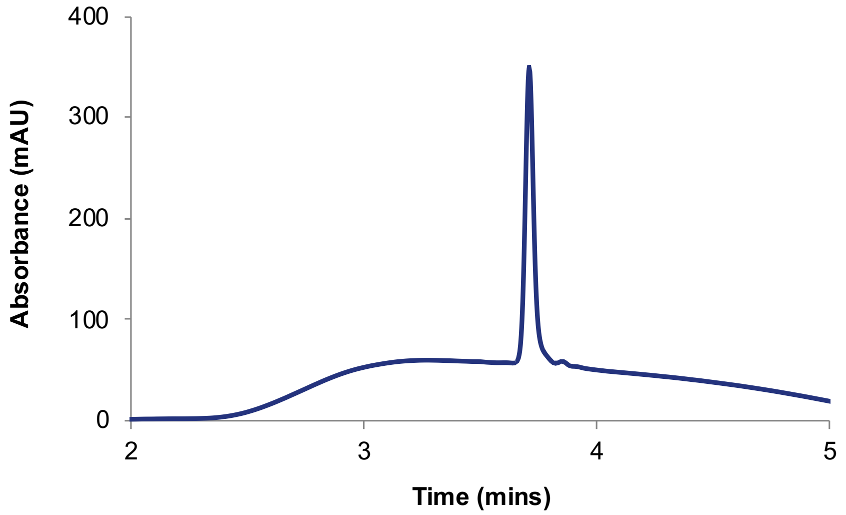

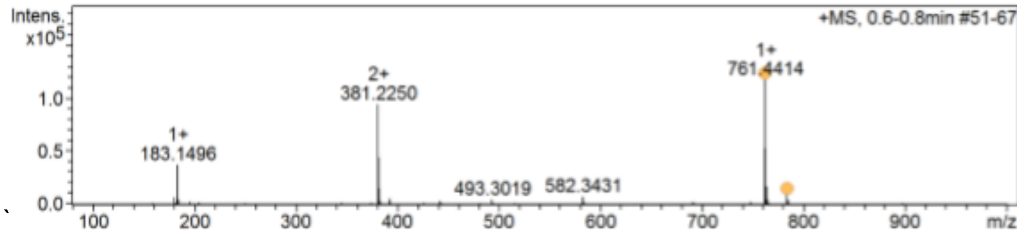

Figure S44. Analytical HPLC trace of pure biotin-EDA-TEMPO modified H-UAF-OMe (**11**). Analytical gradient 10-100% B over 5 min, 210 nm. Calculated Mass $[M+H]^+$: 761.4384, $[M+2H]^{2+}$: 381.2231. Observed Mass $[M+H]^+$: 761.4414, $[M+2H]^{2+}$: 381.2250.

Gemcitabine-TEMPO Conjugate (12)

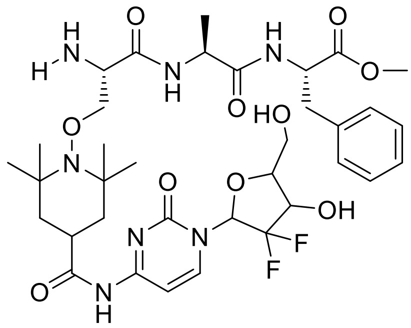

Synthesized using general protocol A using H-UAF-OMe (**1a**) (5 mg, 0.0125 mmol). Purified by preparative RP-HPLC (15 to 80% B over 30 minutes) and lyophilized to produce the desired modified peptide (6.4 mg, 67% yield, 8.36 mmol). ^{1}H NMR (400 MHz, MeOD) δ 8.48 (d, J = 7.7 Hz, 0.5H), 8.37 (d, J = 7.6 Hz, 1H), 8.28 (d, J = 7.7 Hz, 0.5H), 7.50 (d, J = 7.6 Hz, 1H), 7.37 – 7.16 (m, 5H), 6.29-6.15 (m, 1H), 4.64 (m, 1H), 4.49 (m, 1H), 4.32 (m, 1H), 4.18 – 4.06 (m, 3H), 4.04 – 3.94 (m, 2H), 3.83 (m, 1H), 3.71 (s, 3H), 3.18 (ddd, J = 13.9, 5.4, 2.9 Hz, 1H), 3.06 – 2.94 (m, 1H), 1.78 – 1.59 (m, 3H), 1.57 – 1.46 (m, 1H), 1.39 (d, J = 7.0, 1.0 Hz, 3H), 1.28 – 1.05 (m, 12H). ^{13}C NMR (101 MHz, MeOD) δ 185.1, 183.2, 171.9, 165.6, 162.8, 154.4, 150.5, 136.7, 128.2, 126.6, 118.1, 73.73, 71.2, 59.7, 59.6, 58.8, 54.1, 52.2, 51.3, 48.8, 44.4, 41.7, 36.8, 31.8, 19.6, 17.6, 16.1. ^{19}F NMR (376 MHz, MeOD) δ -119.22 (d, J = 248 Hz, 1F), -120.14 (dd, J = 256 Hz, 1F).

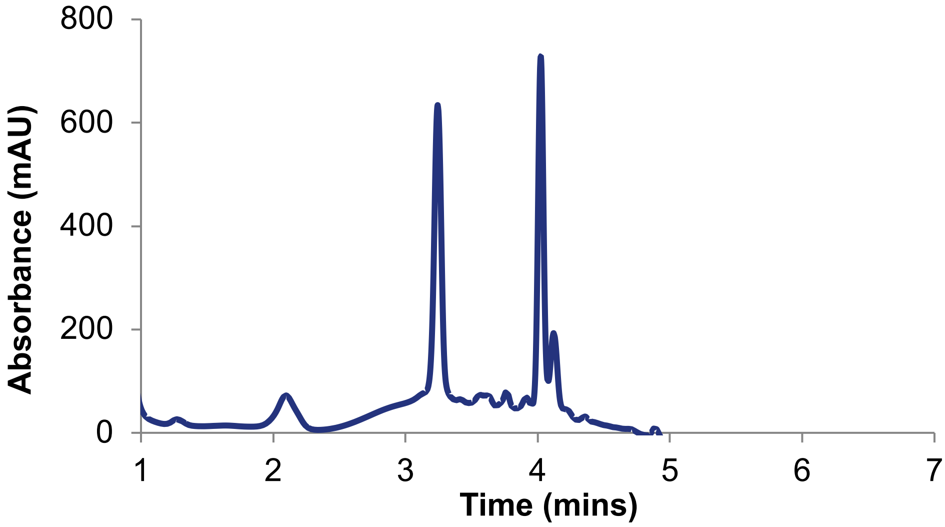

Figure S45. Analytical HPLC trace of 1 hour crude gemcitabine-TEMPO modified H-UAF-OMe (**12**). Analytical gradient 10-100% B over 5 min, 210 nm.

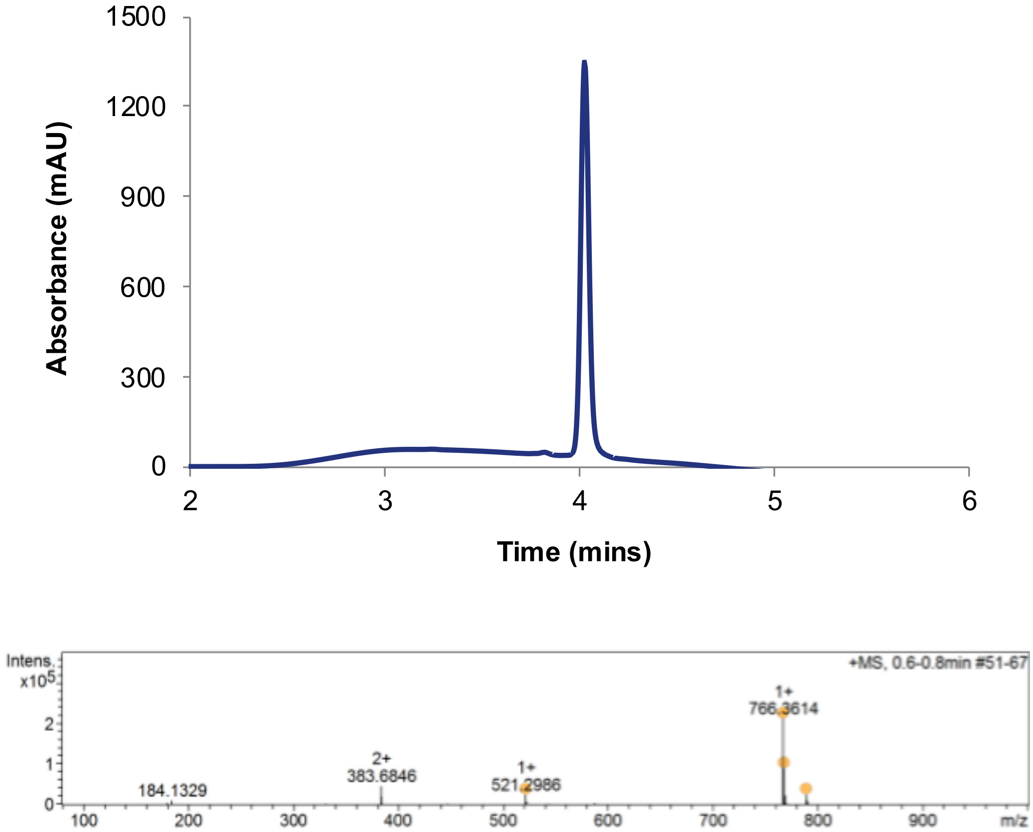

Figure S46. Analytical HPLC trace of pure gemcitabine-TEMPO modified H-UAF-OMe (**12**). Analytical gradient 10-100% B over 5 min, 210 nm. Calculated Mass $[M+H]^+$: 766.3587. Observed Mass $[M+H]^+$: 766.3614.

H-CAF-OMe (13) TEMPO Conjugation (14) Optimization Reactions

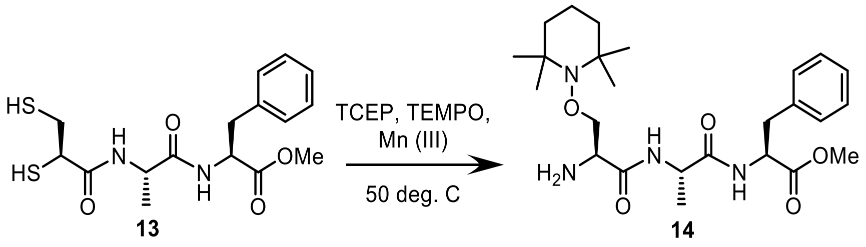

General protocol – Reactions performed on analytical scale (0.5 ml reaction volume) in ligation buffer (pH 7 to 7.5) with 20% DMSO at 50 °C using the specified peptide concentration and reagent equivalences. The reaction was analyzed at 1 and 2 hours. All resulted in complete conversion to TEMPO trapped product, with trial 48 using the lowest equivalences of each reagent.

Table S13. Optimization of TEMPO conjugation with H-CAF-OMe.

Trial	Peptide/ mM	TCEP eq.	TEMPO eq.	$Mn(OAc)_3$ Eq.	1 hr %conv.	2 hrs %conv.
45	2.5	100	2	4	57	>95
46	2.5	50	2	10	66	>95
47	1	100	5	10	68	>95
48	1	50	2	5	75	>95

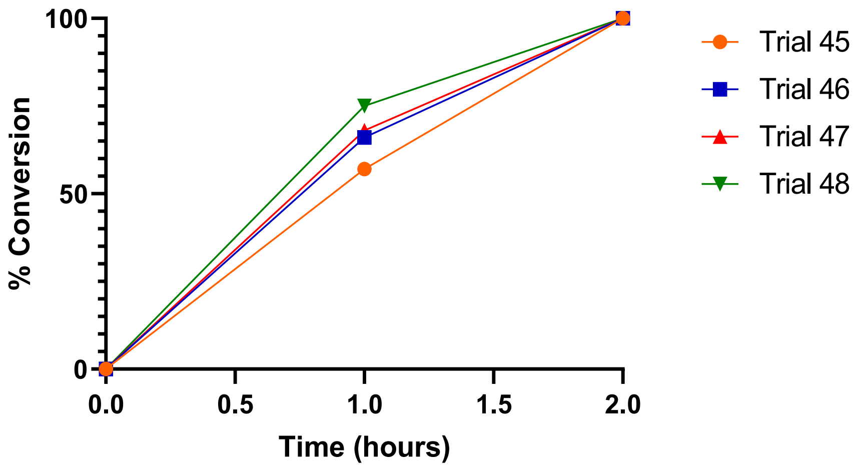

Figure S47. H-CAF-OMe conjugation with TEMPO optimization data.

H-CAF-OMe conjugation with TEMPO (14)

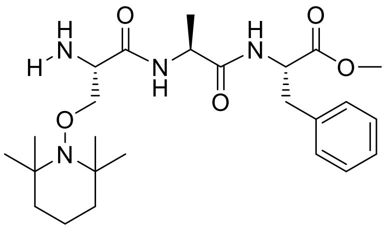

Synthesized following general protocol B using H-CAF-OMe (**13**) (4 mg, 11.3 µmol) followed by purification using preparative RP-HPLC (15-80% B over 30 minutes) and lyophilisation to produce the desired conjugated peptide (3.8 mg, 7.97 µmol, 71% yield). ^{1}H NMR (400 MHz, MeOD) δ 7.33 – 7.20 (m, 5H), 4.64 (m, 1H), 4.48 (q, J = 7.0 Hz, 1H), 4.14 – 4.01 (m, 3H), 3.70 (s, 3H), 3.19 – 3.11 (dd, J = 14.0, 8.8 Hz, 1H), 2.98 (dd, J = 14.0, 8.8 Hz, 1H), 1.63 (m, 2H), 1.51 – 1.42 (m, 4H), 1.38 (d, J = 7.0 Hz, 3H), 1.19 – 1.00 (m, 12H).^{13}C NMR (101 MHz, MeOD) δ 172.6, 171.9, 165.6, 136.7, 128.8, 128.2, 126.6, 73.6, 60.2, 60.1, 54.1, 52.3, 51.3, 48.8, 39.4, 36.8, 32.0, 19.2, 17.6, 16.5.

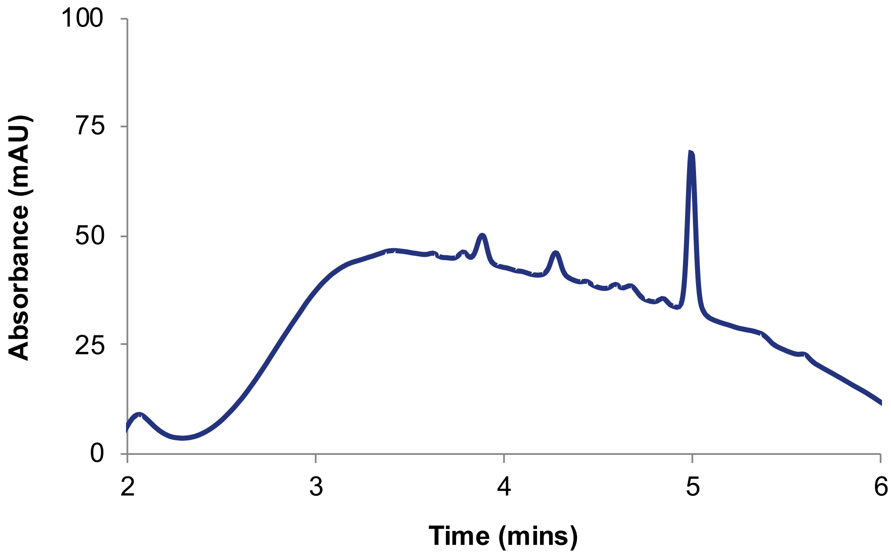

Figure S48. Analytical HPLC trace of 2 hours crude conjugation of H-CAF-OMe using TEMPO (**14**). Analytical gradient 10-70% B over 5 min, 210 nm.

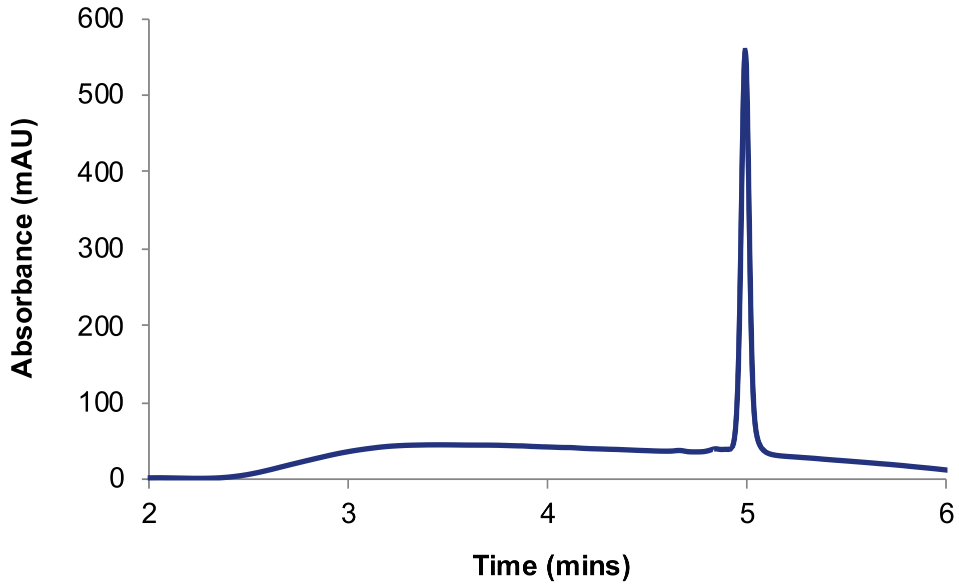

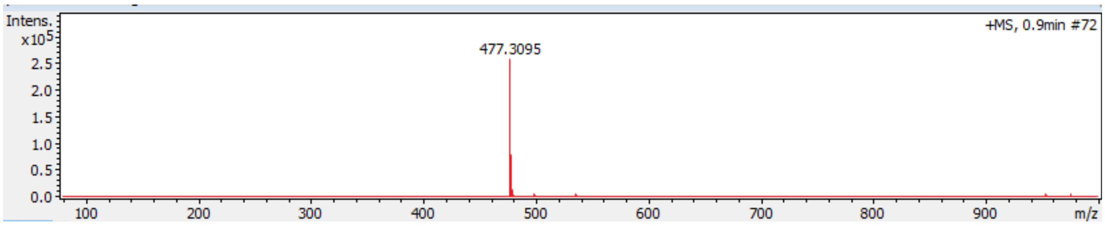

Figure S49. Analytical HPLC trace of pure conjugated H-CAF-OMe using TEMPO (**14**). Analytical gradient 10-70% B over 5 min, 210 nm. Calculated Mass $[M+H]^+$: 477.3077. Observed Mass $[M+H]^+$: 477.3095.

Cys Conjugation – Optimization

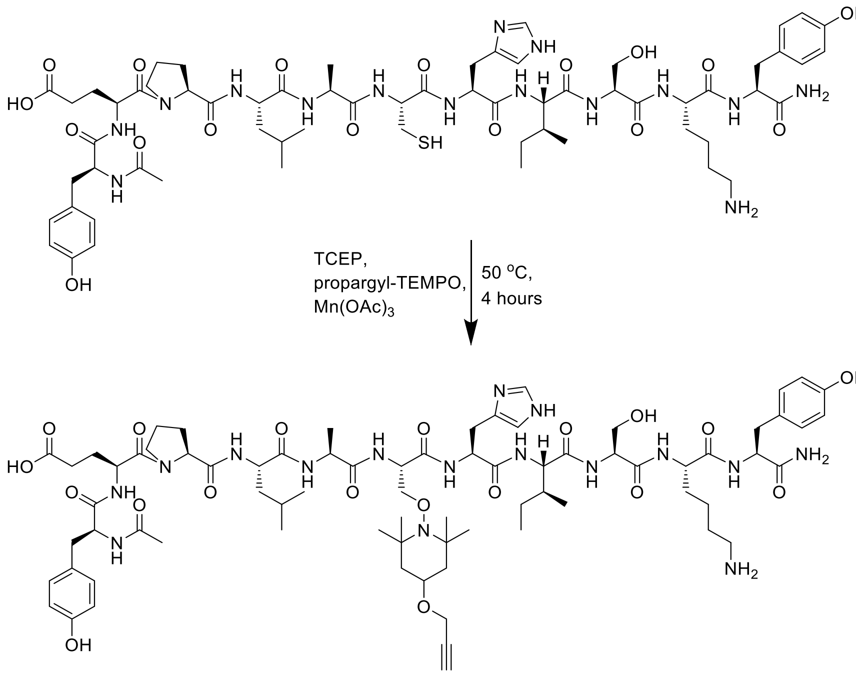

All reactions performed at 50 °C for 4 hours. Reaction mixture analyzed by RP-HPLC (10-100% B over 5 min) at 2 and 4 hours.

Table S14. Optimization of bioconjugation reaction of Ac-YEPLACHISKY-NH_2 with propargyl-TEMPO.

Trial	Peptide/ mM	TCEP eq.	Propargyl TEMPO eq.	$Mn(OAc)_3$ eq.	2 hrs %conv.	4 hrs %conv.
49	1	50	2	5	39	45
50	1	50	2	20	55	73
51	1	100	2	20	52	82
52	1	100	5	20	53	83
53	2.5	50	5	10	50	62
54	2.5	50	2	10	71	81
55	2.5	100	2	5	42	52
56	2.5	100	5	10	64	73

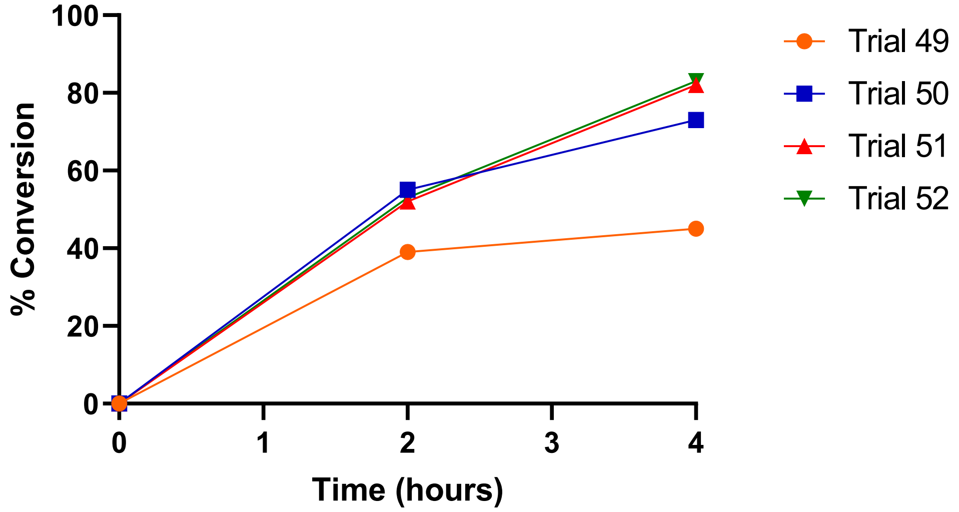

Figure S50. Optimization of the conjugation of Ac-YEPLACHISKY-NH_2 with propargyl-TEMPO at 1 mM peptide concentration (**28**).

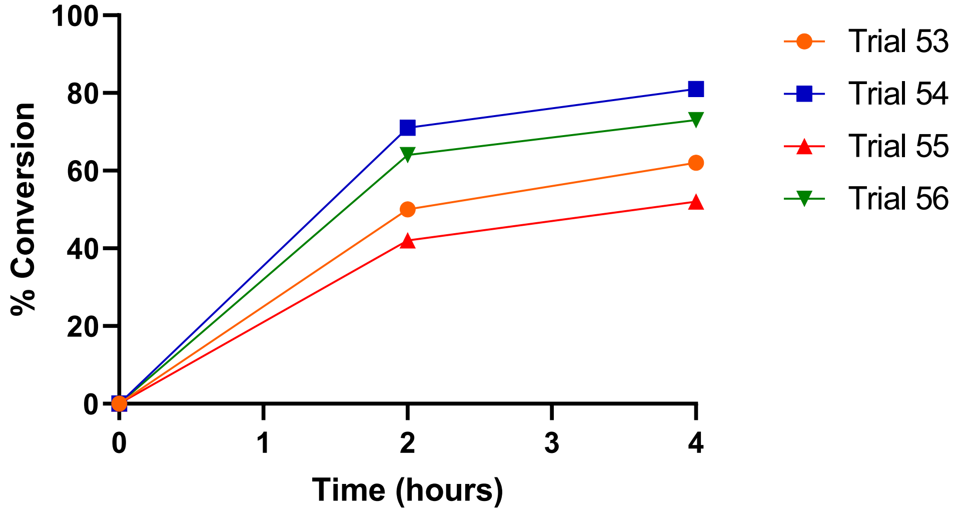

Figure S51. Optimization of the conjugation of Ac-YEPLACHISKY-NH_2 with propargyl-TEMPO at 2.5 mM peptide concentration (**28**).

Ac-CWHISKEY-NH_2 Conjugation Reactions (16-20)

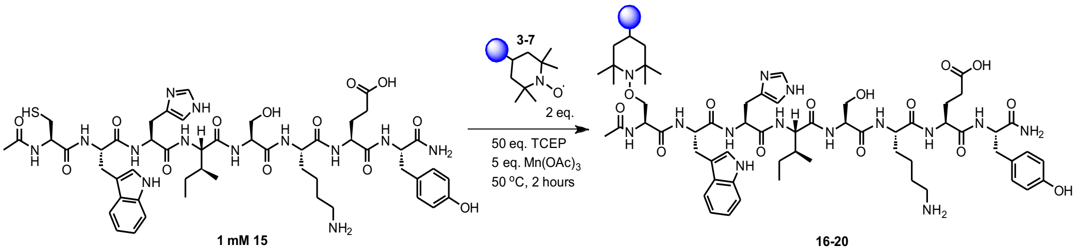

Ac-CWHISKEY-NH_2 with tetra-EG-TEMPO (16)

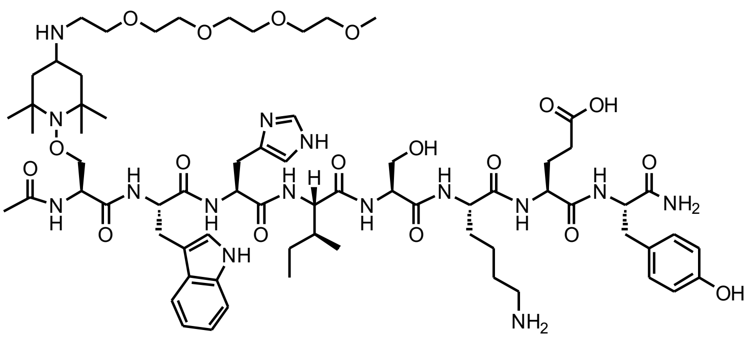

Synthesized following general protocol B using Ac-CWHISKEY-NH_2 (**15**) (2 mg, 1.81 µmol) followed by purification using semi-preparative RP-HPLC (15-80% B over 30 minutes) and lyophilisation to produce the desired conjugated peptide (2.7 mg, 3.47 µmol, 80% yield).

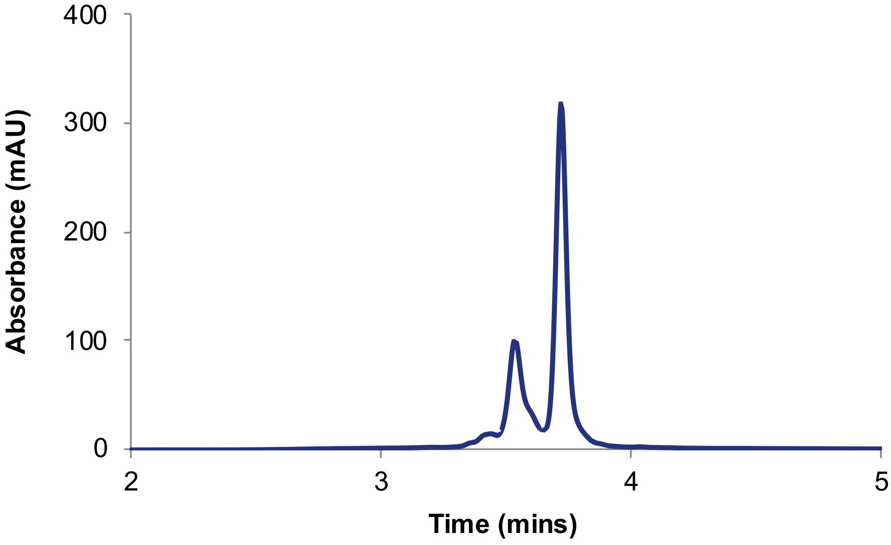

Figure S52. Analytical HPLC trace of 2 hours crude conjugation of Ac-CWHISKEY-NH_2 using tetra-EG-TEMPO (**16**). Analytical gradient 10-100% B over 5 min, 210 nm.

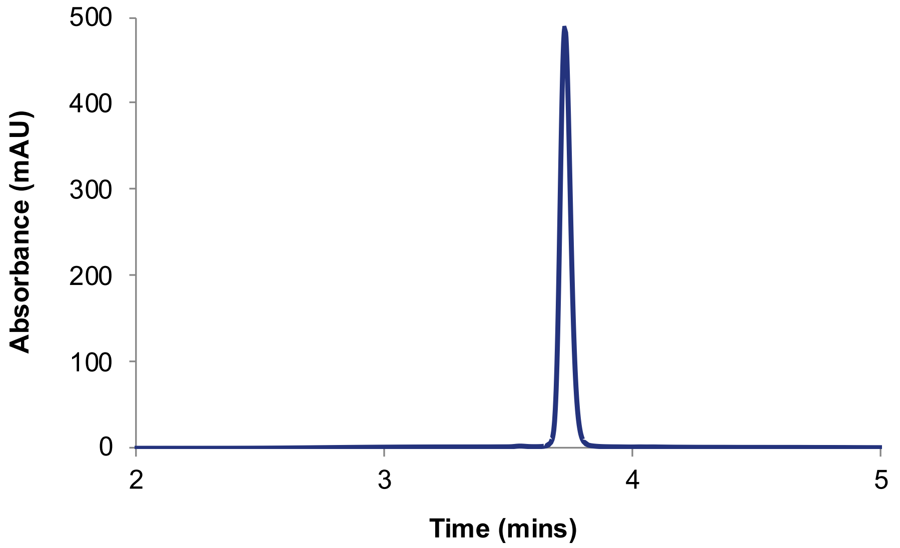

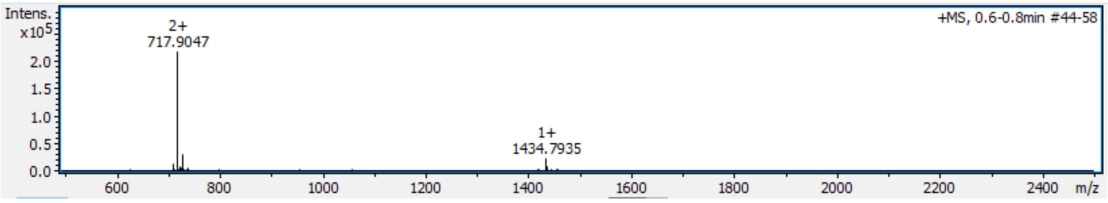

Figure S53. Analytical HPLC trace of pure conjugated Ac-CWHISKEY-NH_2 using tetra-EG-TEMPO (**16**). Analytical gradient 10-100% B over 5 min, 210 nm. Calculated Mass $[M+H]^+$: 1434.7997, $[M+2H]^{2+}$: 717.9038. Observed Mass $[M+H]^+$: 1434.7935, $[M+2H]^{2+}$: 717.9047.

Ac-CWHISKEY-NH2 with Propargyl-TEMPO (17)

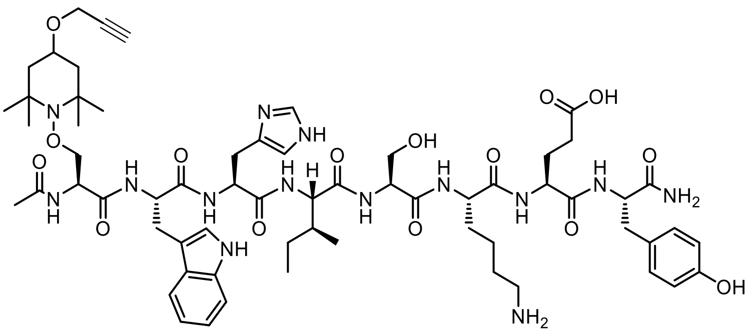

Synthesized following general protocol B using Ac-CWHISKEY-NH2 (**15**) (2 mg, 1.81 μmol) followed by purification using semi-preparative RP-HPLC (15-80% B over 30 minutes) and lyophilisation to produce the desired conjugated peptide (1.9 mg, 1.48 μmol, 82% yield).

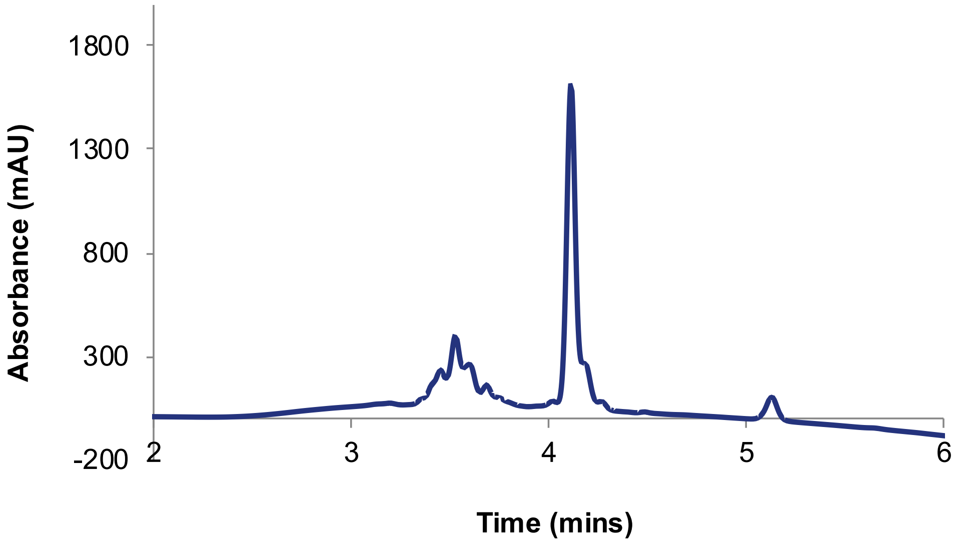

Figure S54. Analytical HPLC trace of 2 hours crude conjugation of Ac-CWHISKEY-NH2 using propargyl-TEMPO (**17**). Analytical gradient 10-100% B over 5 min, 210 nm.

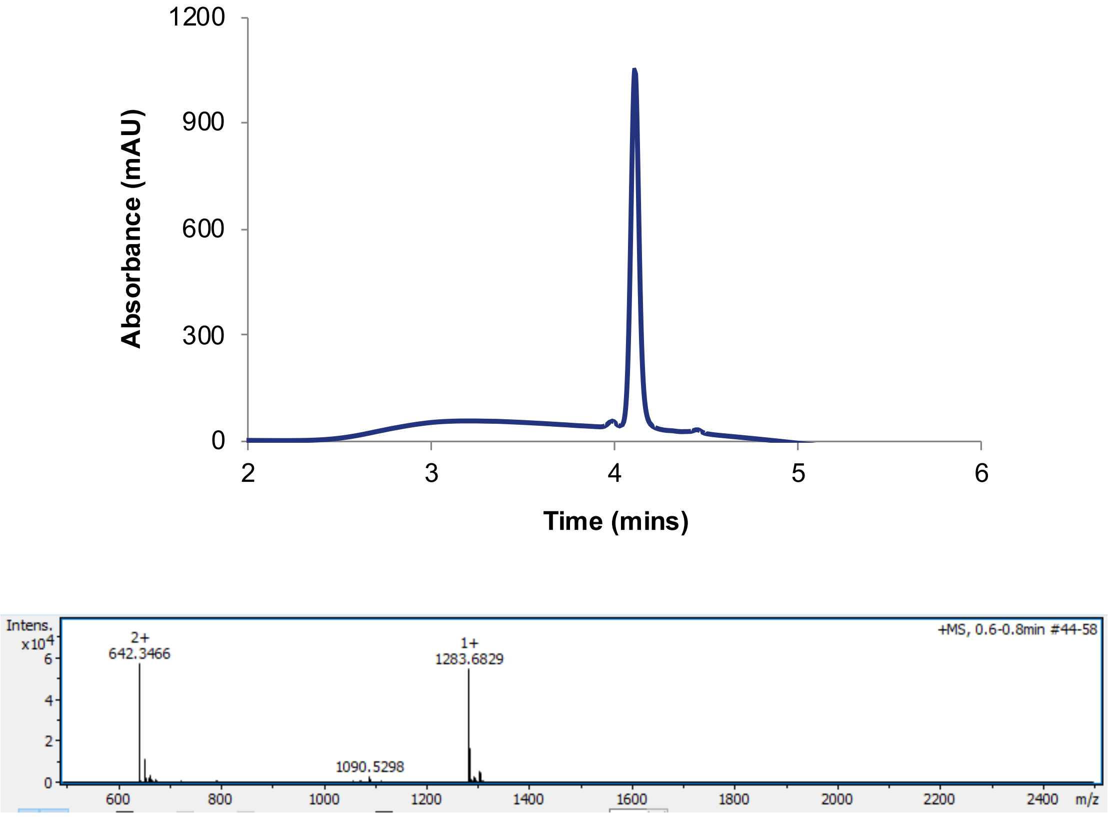

Figure S55. Analytical HPLC trace of pure conjugated Ac-CWHISKEY-NH_2 using propargyl-TEMPO (**17**). Analytical gradient 10-100% B over 5 min, 210 nm. Calculated Mass $[M+H]^+$: 1283.6788, $[M+2H]^{2+}$: 642.3433. Observed Mass $[M+H]^+$: 1283.6829, $[M+2H]^{2+}$: 642.3466.

Analytical-scale CuAAC (Copper(I)-Catalyzed Alkyne-Azide Cycloaddition) between model 17 and 3-azidopropyl-1-amine (59)

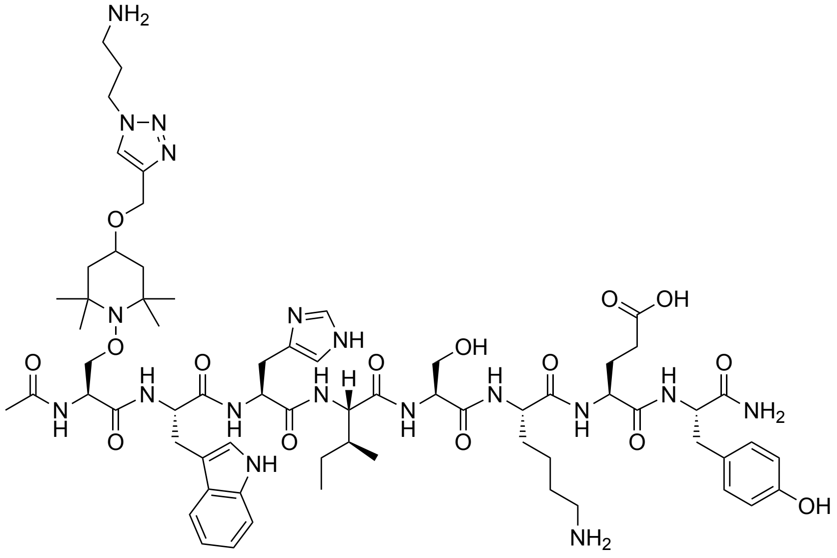

A 2.5 mM solution of starting peptide **17** material was prepared in ligation buffer at pH 7. To this solution was added 3-azidopropyl-1-amine (5 eq.), $CuSO_4$ (0.5 eq.) and sodium ascorbate (5 eq.). The solution was diluted to a final peptide concentration of 1 mM and agitated at 37 °C for 2 hours before analysis using analytical HPLC.

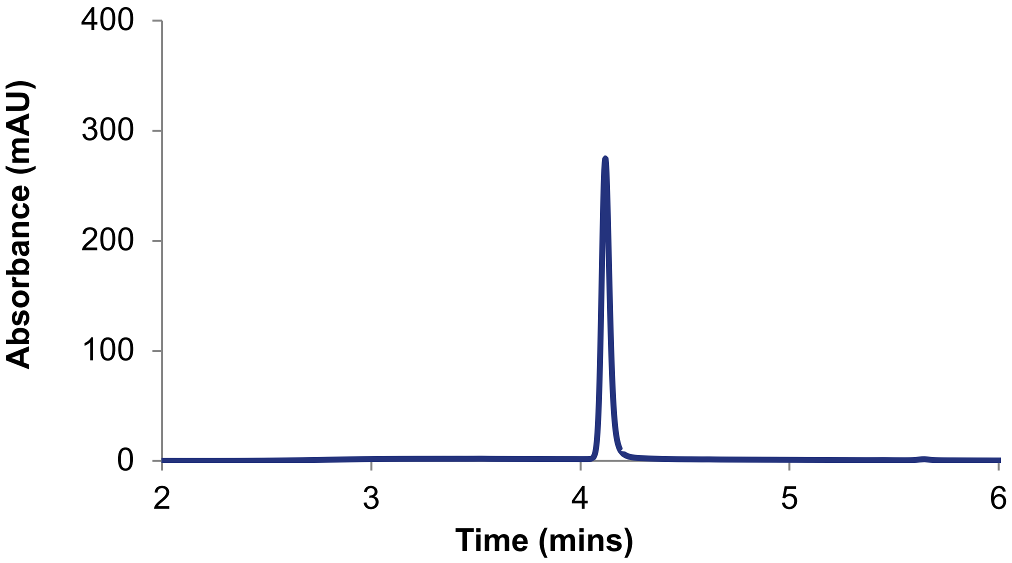

Figure S56. Pure A(Propargyl-TEMPO)WHISKEY-NH_2 starting material. Analytical gradient 10-100% B over 5 minutes, 280 nm.

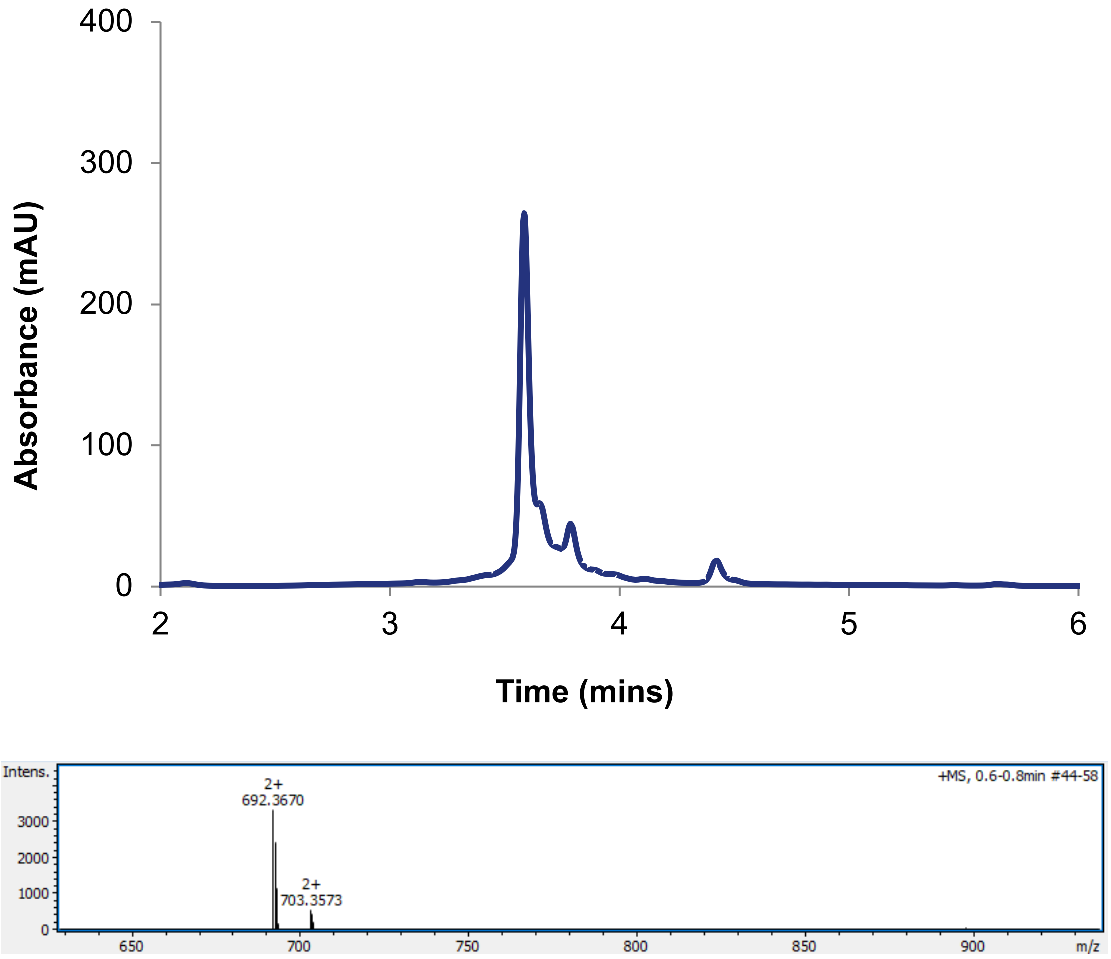

Figure S57. Crude trace from the CuAAC reaction trial showing no starting material remaining after 2 hours. Analytical gradient 10-100% B over 5 minutes, 280 nm. MS analysis of main product **59** peak showing a successful reaction. Calculated Mass $[M+2H]^{2+}$: 692.3808. Observed Mass $[M+2H]^{2+}$: 692.3670. *Note: the amino-oxy linker begins to degrade in the presence of Cu(I) over a 16 hr period.*

Ac-CWHISKEY-NH2 with FITC-TEMPO (18)

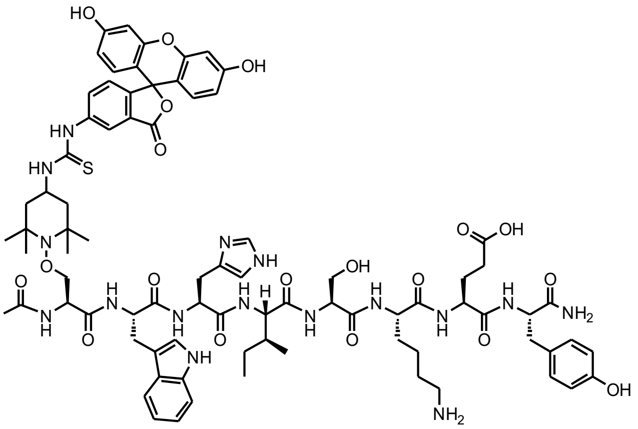

Synthesized following general protocol B using Ac-CWHISKEY-NH2 (**15**) (2 mg, 1.81 μmol) followed by purification using semi-preparative RP-HPLC (15-80% B over 30 minutes) and lyophilisation to produce the desired conjugated peptide (2.1 mg, 1.29 μmol, 71% yield).

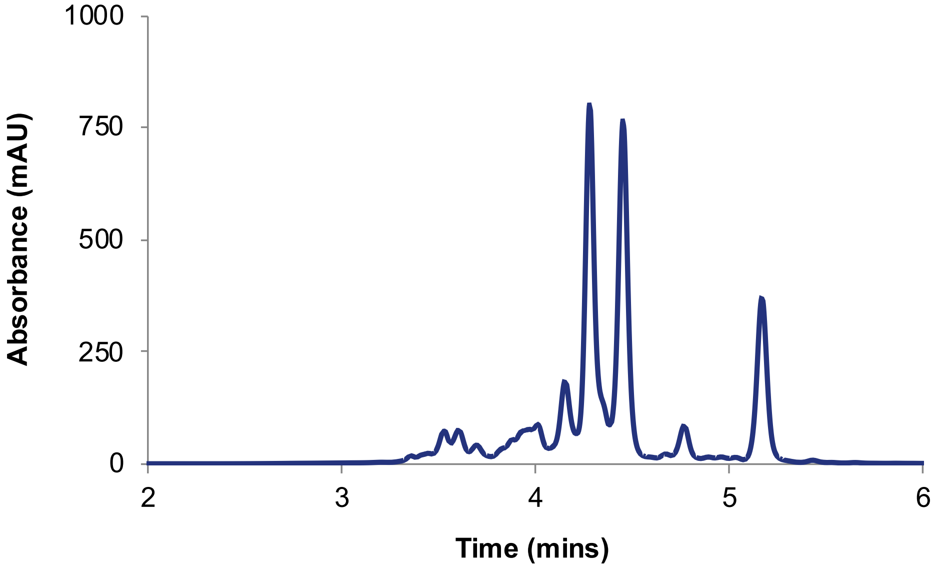

Figure S58. Analytical HPLC trace of 2 hours crude conjugation of Ac-CWHISKEY-NH2 using FITC-TEMPO (**18**). Analytical gradient 10-100% B over 5 min, 280 nm.

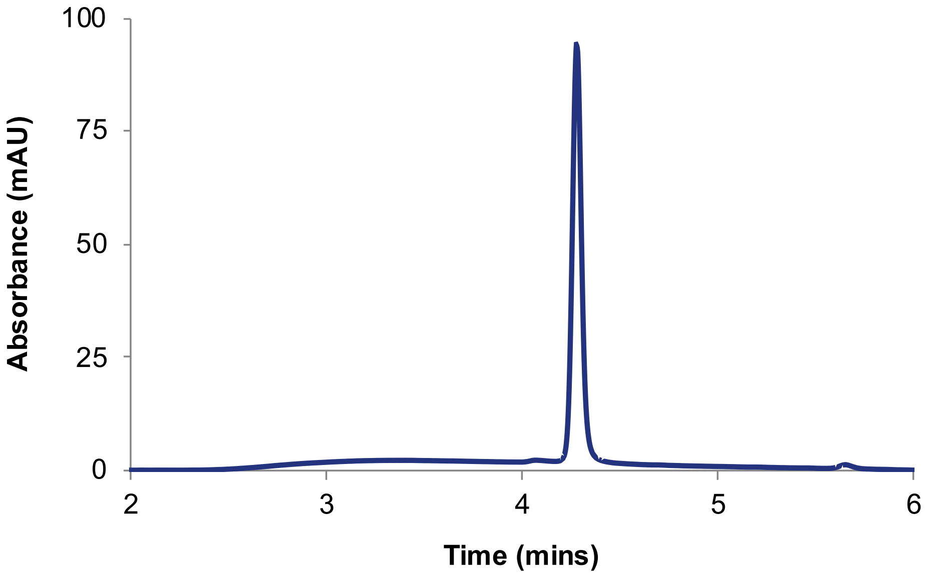

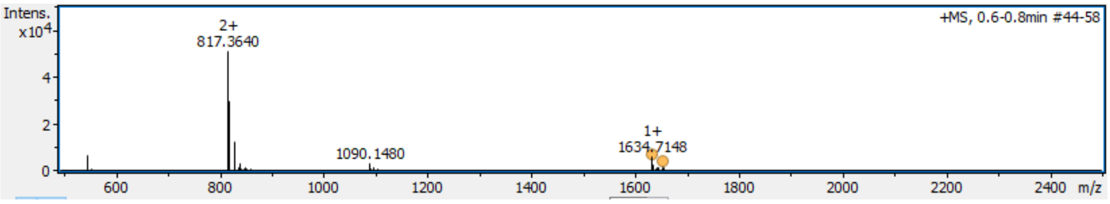

Figure S59. Analytical HPLC trace of pure conjugated of Ac-CWHISKEY-NH_2 using FITC-TEMPO (**18**). Analytical gradient 10-100% B over 5 min, 280 nm. Calculated Mass $[M+H]^+$: 1634.7228, $[M+2H]^{2+}$: 817.3614. Observed Mass $[M+H]^+$: 1634.7148, $[M+2H]^{2+}$: 817.3640.

Ac-CWHISKEY-NH$_2$ with Biotin-EDA-TEMPO (19)

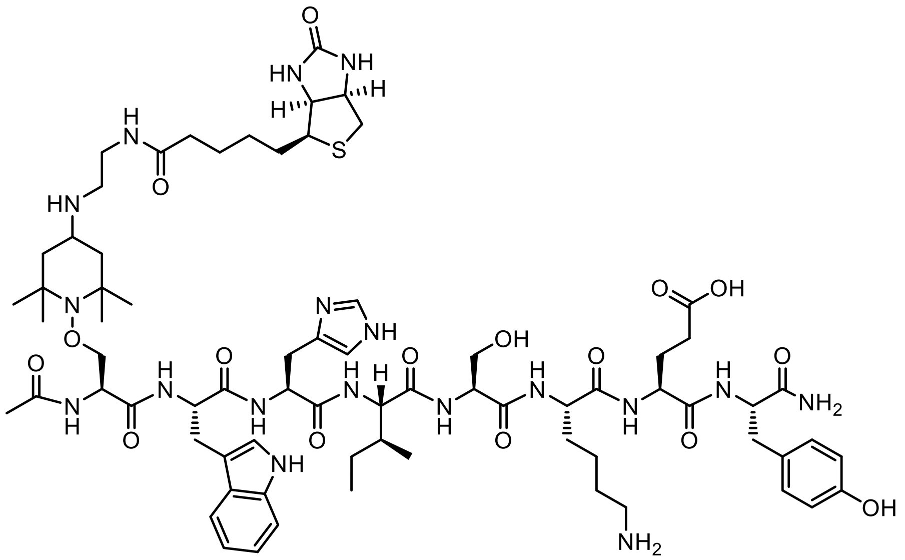

Synthesized following general protocol B using Ac-CWHISKEY-NH$_2$ (**15**) (2 mg, 1.81 μmol) followed by purification using semi-preparative RP-HPLC (15-80% B over 30 minutes) and lyophilisation to produce the desired conjugated peptide (2.0 mg, 1.32 μmol, 73% yield).

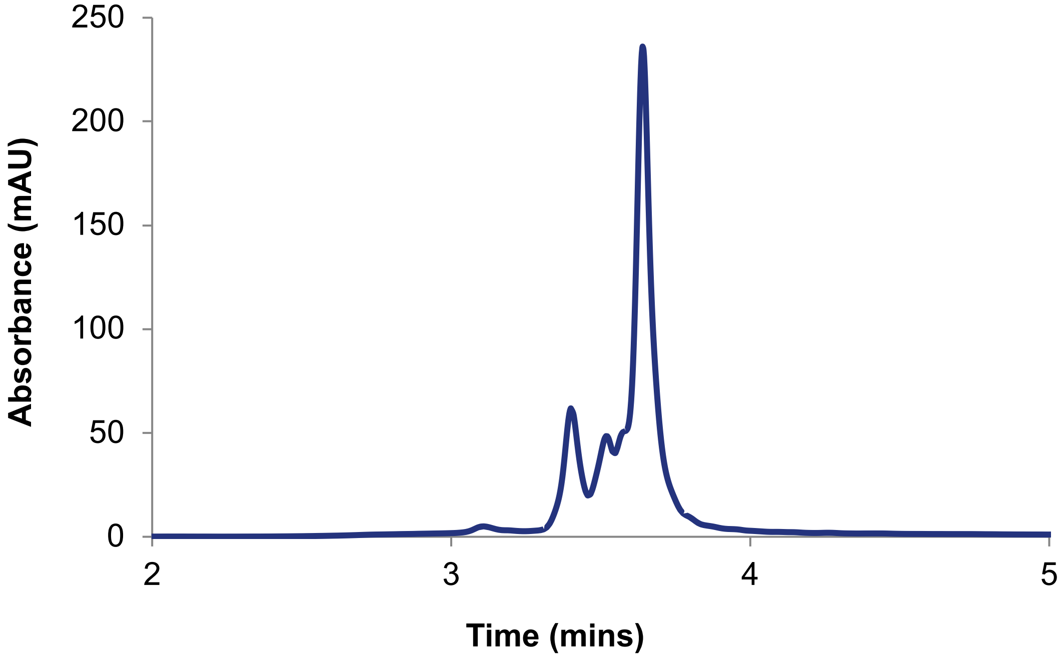

Figure S60. Analytical HPLC trace of 2 hours crude conjugation of Ac-CWHISKEY-NH$_2$ using Biotin-EDA-TEMPO (**19**). Analytical gradient 10-100% B over 5 min, 280 nm.

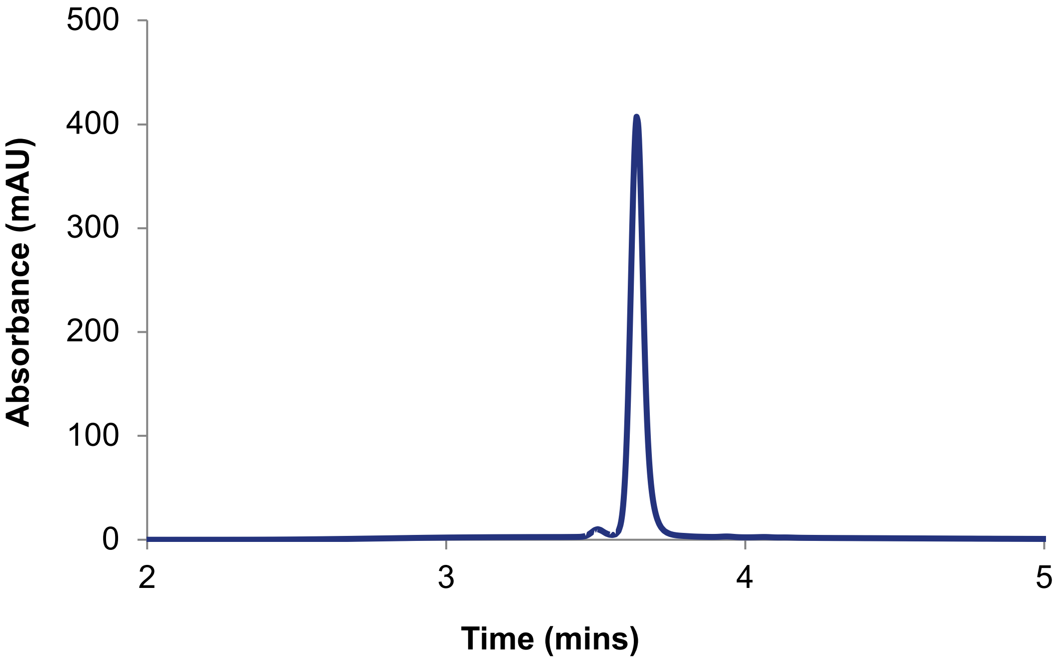

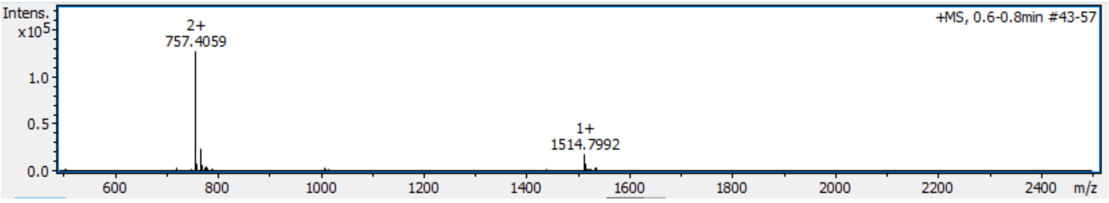

Figure S61. Analytical HPLC trace of pure conjugated of Ac-CWHISKEY-NH_2 using Biotin-EDA-TEMPO (**19**). Analytical gradient 10-100% B over 5 min, 280 nm. Calculated Mass $[M+H]^+$: 1513.7990, $[M+2H]^{2+}$: 757.3995. Observed Mass $[M+H]^+$: 1514.7992, $[M+2H]^{2+}$: 757.4059.

Ac-CWHISKEY-NH$_2$ with Gemcitabine-TEMPO (20)

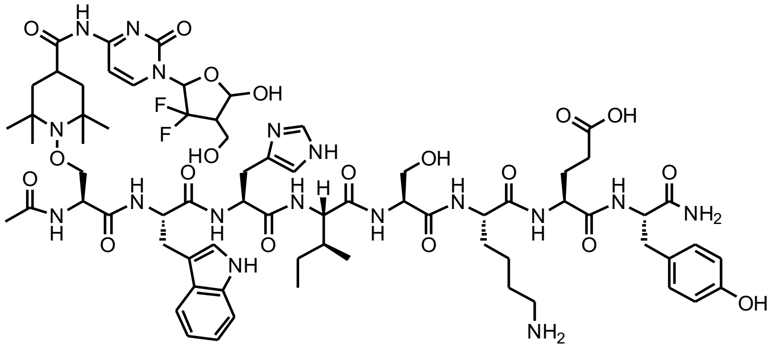

Synthesized following general protocol B using Ac-CWHISKEY-NH$_2$ (**15**) (5 mg, 4.52 µmol) followed by purification using semi-preparative RP-HPLC (15-80% B over 30 minutes) and lyophilisation to produce the desired conjugated peptide (5.2 mg, 3.47 µmol, 80% yield).

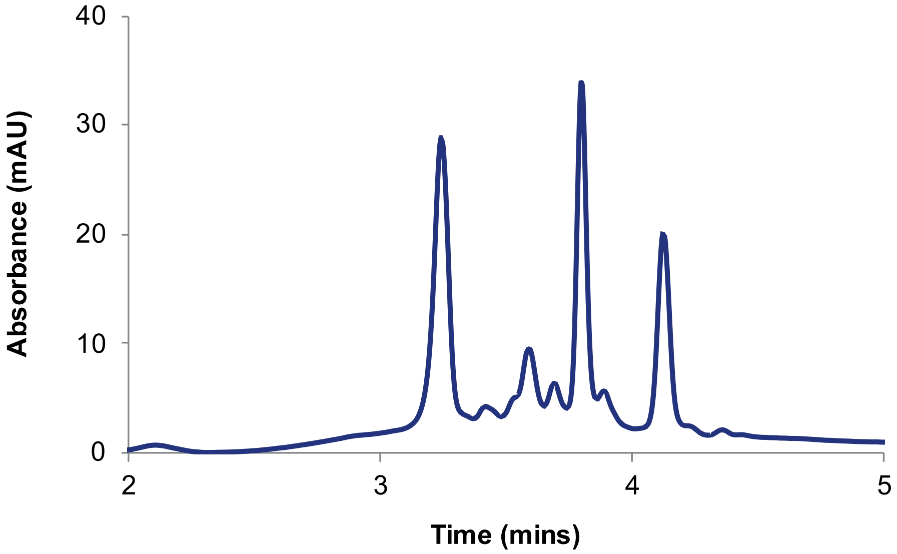

Figure S62. Analytical HPLC trace of 2 hours crude conjugation of Ac-CWHISKEY-NH$_2$ using gemcitabine-TEMPO (**20**). Analytical gradient 10-100% B over 5 min, 280 nm.

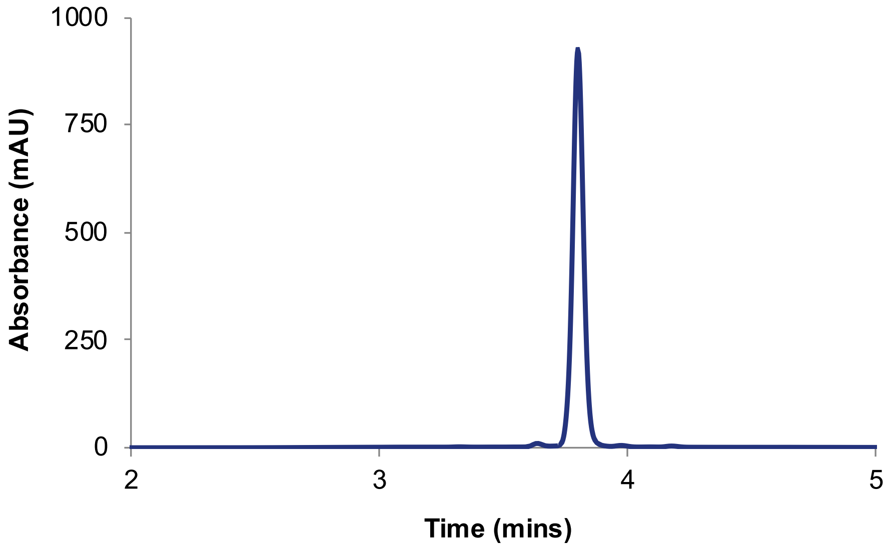

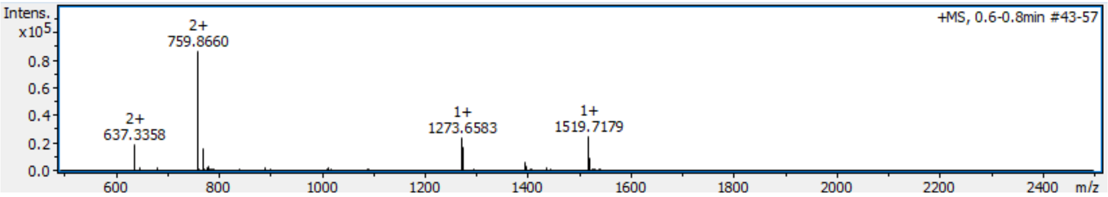

Figure S63. Analytical HPLC trace of pure conjugated Ac-CWHISKEY-NH_2 using gemcitabine-TEMPO (**20**). Analytical gradient 10-100% B over 5 min, 280 nm. Calculated Mass $[M+H]^+$: 1519.7272, $[M+2H]^{2+}$: 759.8636. Observed Mass $[M+H]^+$: 1519.7179, $[M+2H]^{2+}$: 759.8660.

H-A(TEMPO)WIMKY-NH$_2$ (22) – Met stability to conditions

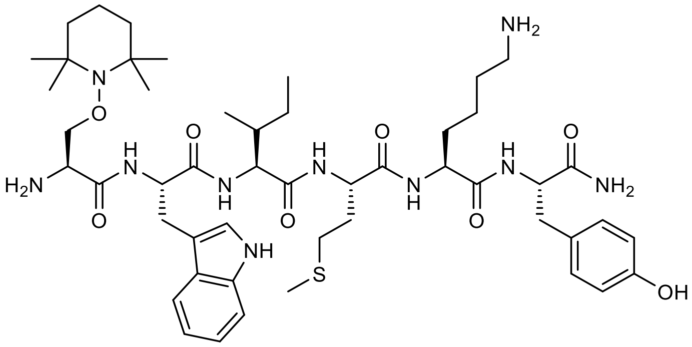

Synthesized using general protocol A using H-UWIMKY-NH$_2$ (**21**) (4 mg, 0.0044 mmol). Purified by preparative RP-HPLC (15 to 80% B over 30 minutes) and lyophilized to produce the desired modified peptide (2.8 mg, 0.003 mmol, 68%).

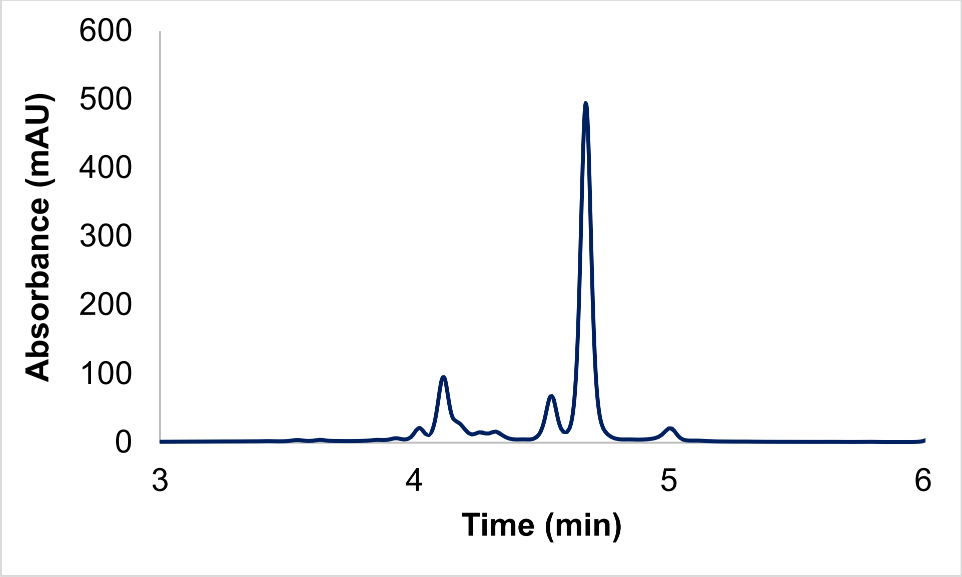

Figure S64. Analytical HPLC trace of 30 min crude TEMPO-WIMKY. Analytical gradient 10-75% B over 10 min, 210 nm.

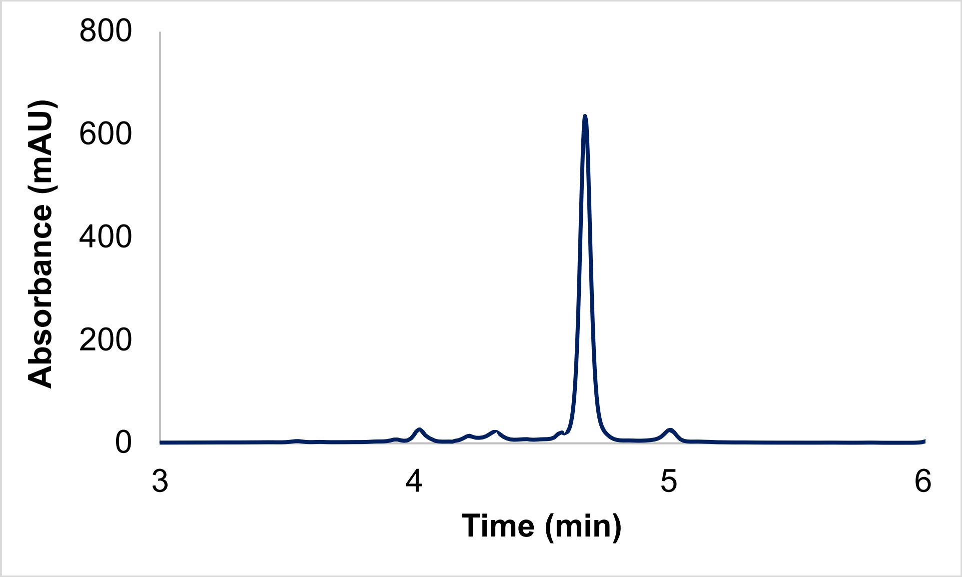

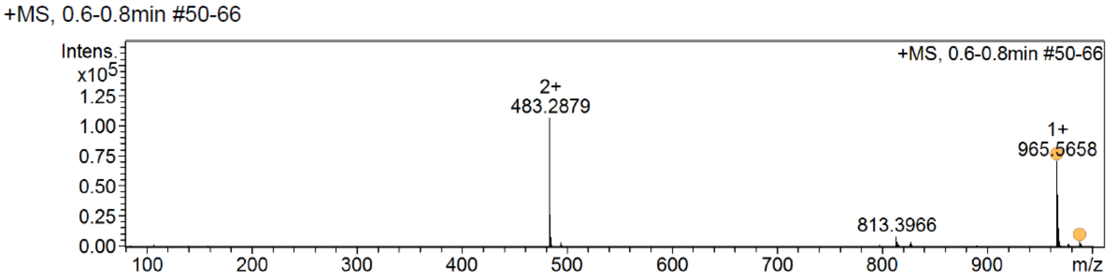

Figure S65. Analytical HPLC trace of pure TEMPO-WIMKY-NH_2 (**22**). Analytical gradient 10-75% B over 10 min, 210 nm. HRMS Calculated Mas: $[M+H]^+$ = 965.5641, $[M+2H]^{2+}$: 483.2860. Observed Mass: $[M+H]^+$ = 965.5658, $[M+2H]^{2+}$: 483.2879.

Ligation-conjugation protocols

Ac-YEPLA-SePh (23) and H-UHISKY-NH2 (24) One-pot Ligation and Conjugation (27 - 31)

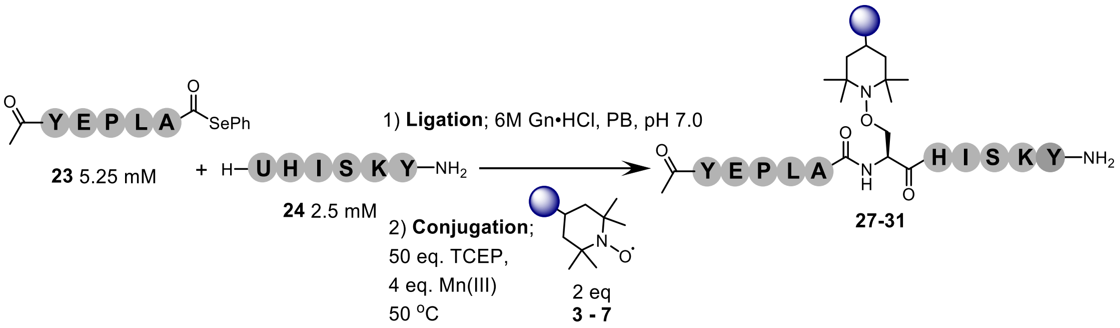

Tetra-EG-TEMPO Conjugate (27)

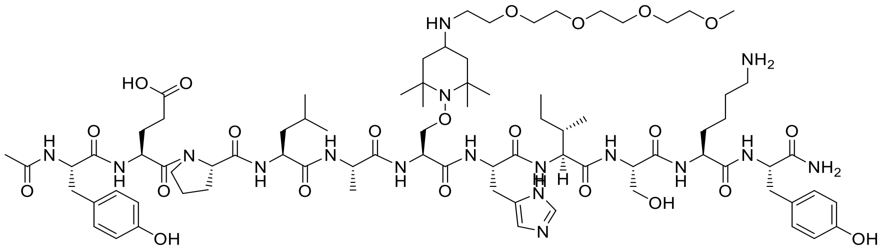

Synthesized following general one-pot ligation-conjugation protocol using H-UHISKY-NH2 (**24**) (2 mg, 2.51 μmol) and Ac-YEPLA-SePh (**23**) (2.04 mg, 2.64 μmol). Purified by semi-preparative RP-HPLC (15-80% B over 30 minutes) and lyophilised to yield the desired modified peptide (3.3 mg, 1.95 μmol, 78% yield).

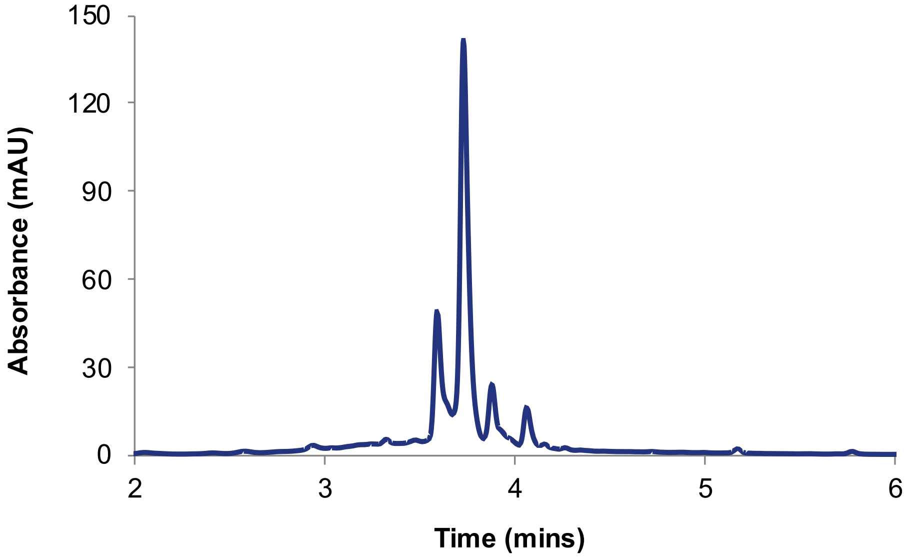

Figure S66. Analytical HPLC trace of 1 hour crude tetra-EG-TEMPO modified Ac-YEPLAUHISKY-NH_2 from one-pot ligation-conjugation (**27**). Analytical gradient 10-100% B over 5 min, 210 nm.

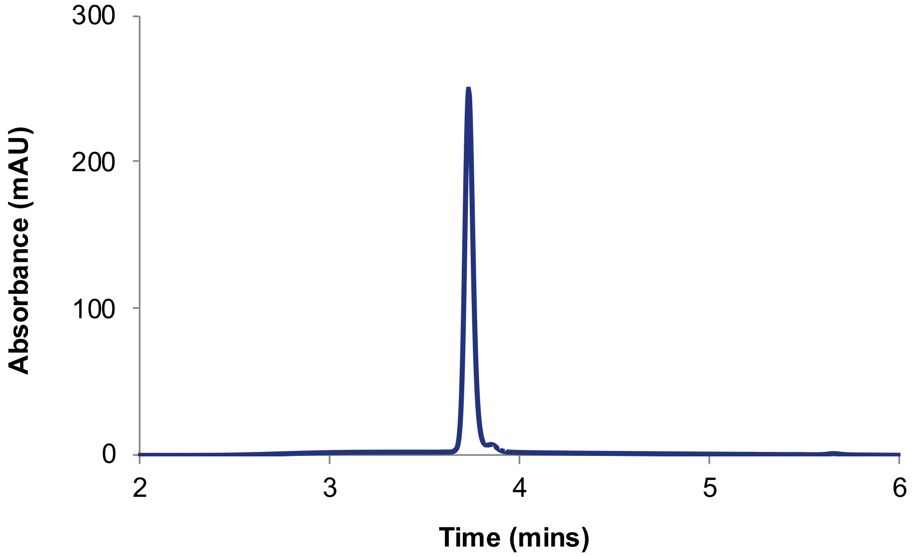

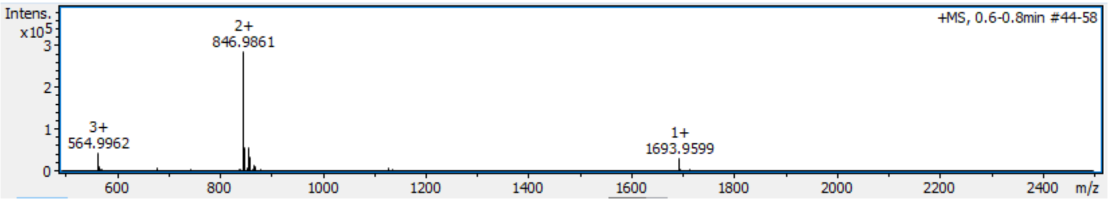

Figure S67. Analytical HPLC trace of pure tetra-EG-TEMPO modified Ac-YEPLAUHISKY-NH_2 from one-pot ligation-conjugation (**27**). Analytical gradient 10-100% B over 5 min, 210 nm. Calculated Mass $[M+H]^+$: 1692.9654, $[M+2H]^{2+}$: 846.9827. Observed Mass $[M+H]^+$: 1693.9599, $[M+2H]^{2+}$: 846.9861.

Propargyl-TEMPO Conjugate (28)

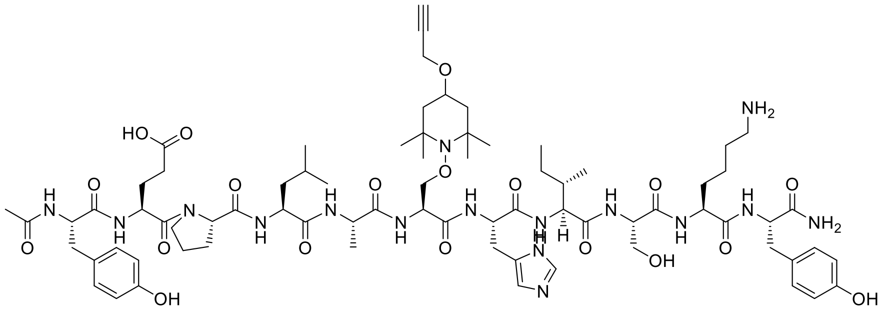

Synthesized following general one-pot ligation-conjugation protocol using H-UHISKY-NH_2 (**24**) (2 mg, 2.51 μmol) and Ac-YEPLA-SePh (**23**) (2.04 mg, 2.64 μmol). Purified by semi-preparative RP-HPLC (15-80% B over 30 minutes) and lyophilised to yield the desired modified peptide (3.1 mg, 2.01 μmol, 80% yield).

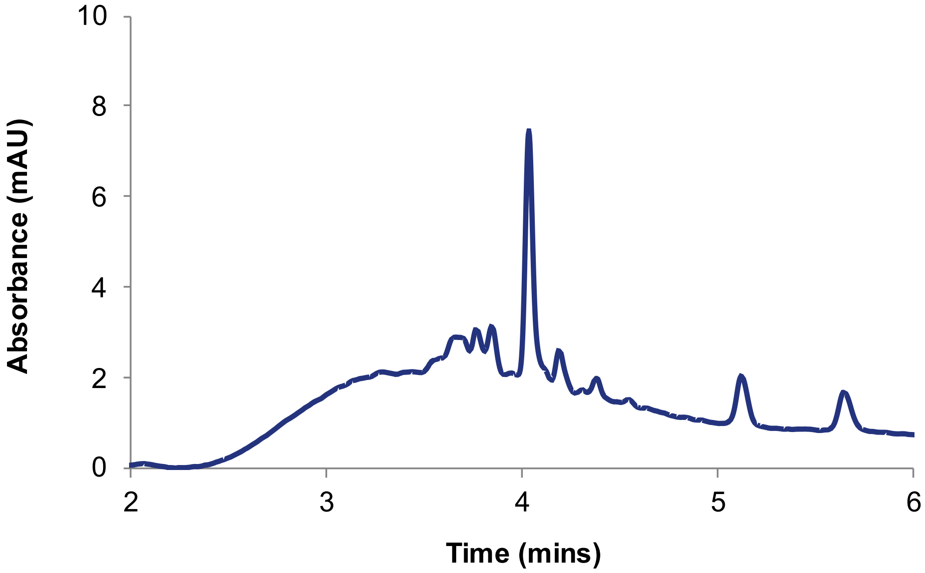

Figure S68. Analytical HPLC trace of 1 hour crude propargyl-TEMPO modified Ac-YEPLAUHISKY-NH_2 from one-pot ligation-conjugation (**28**). Analytical gradient 10-100% B over 5 min, 210 nm.

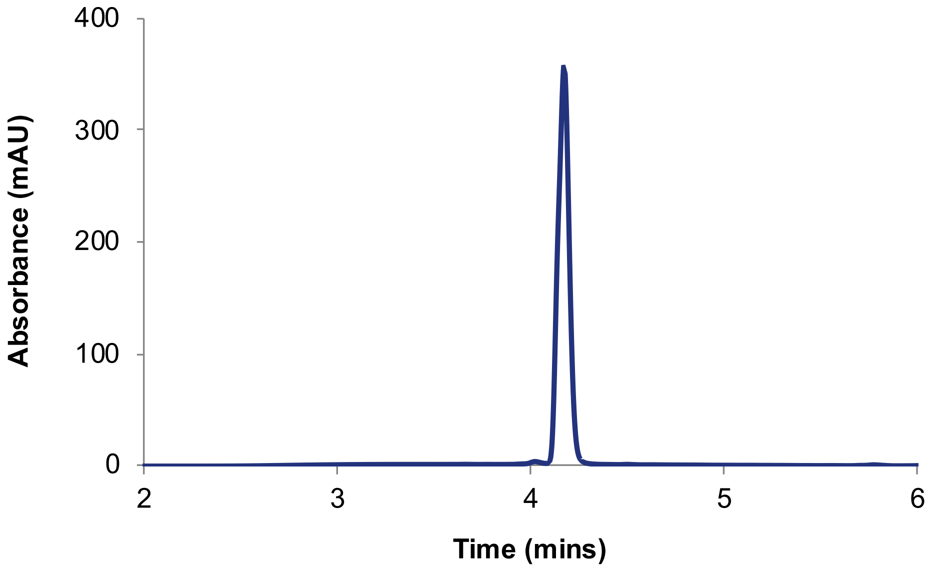

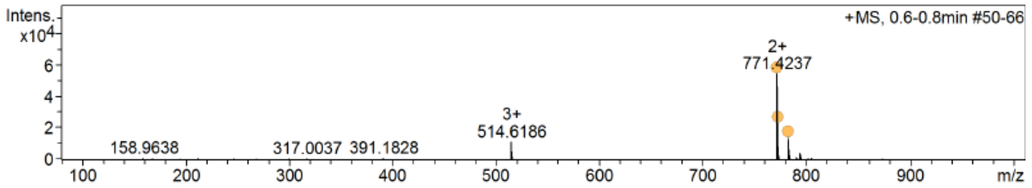

Figure S69. Analytical HPLC trace of pure propargyl-TEMPO modified Ac-YEPLAUHISKY-NH_2 from one-pot ligation-conjugation (**28**). Analytical gradient 10-100% B over 5 min, 210 nm. Calculated Mass $[M+2H]^{2+}$: 771.4223. Observed Mass $[M+2H]^{2+}$: 771.4237.

Biotin-TEMPO Conjugate (29)

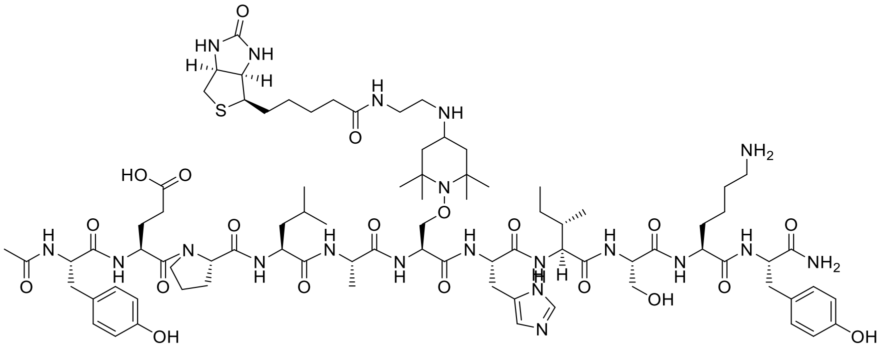

Synthesized following general one-pot ligation-conjugation protocol using H-UHISKY-NH_2 (**24**) (2 mg, 2.51 µmol) and Ac-YEPLA-SePh (**23**) (2.04 mg, 2.64 µmol). Purified by semi-preparative RP-HPLC (15-80% B over 30 minutes) and lyophilised to yield the desired modified peptide (3.4 mg, 1.92 µmol, 76% yield).

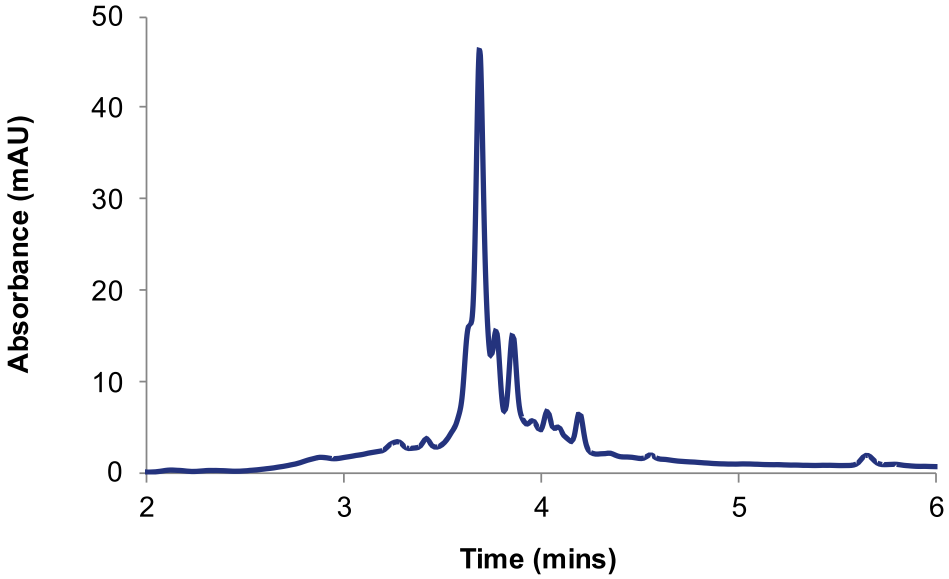

Figure S70. Analytical HPLC trace of 1 hour crude biotin-EDA-TEMPO modified Ac-YEPLAUHISKY-NH_2 from one-pot ligation-conjugation (**29**). Analytical gradient 10-100% B over 5 min, 210 nm.

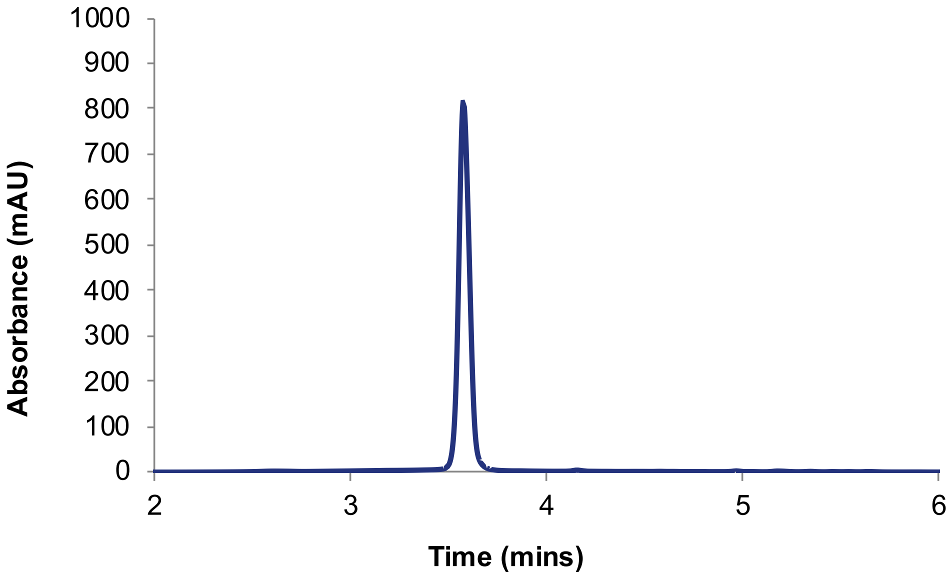

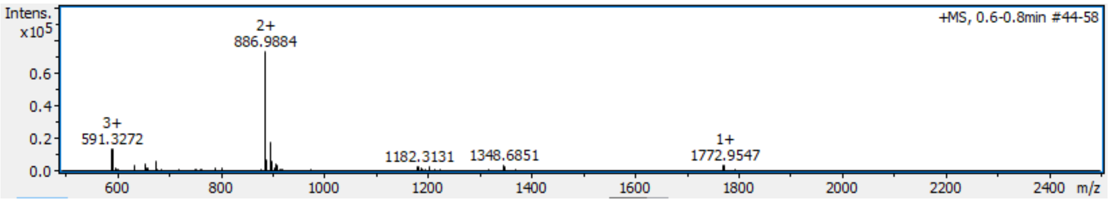

Figure S71. Analytical HPLC trace of pure biotin-EDA-TEMPO modified Ac-YEPLAUHISKY-NH_2 from one-pot ligation-conjugation (**29**). Analytical gradient 10-100% B over 5 min, 210 nm. Calculated Mass $[M+2H]^{2+}$: 886.4824. Observed Mass $[M+2H]^{2+}$: 886.9884.

FITC-TEMPO Conjugate (30)

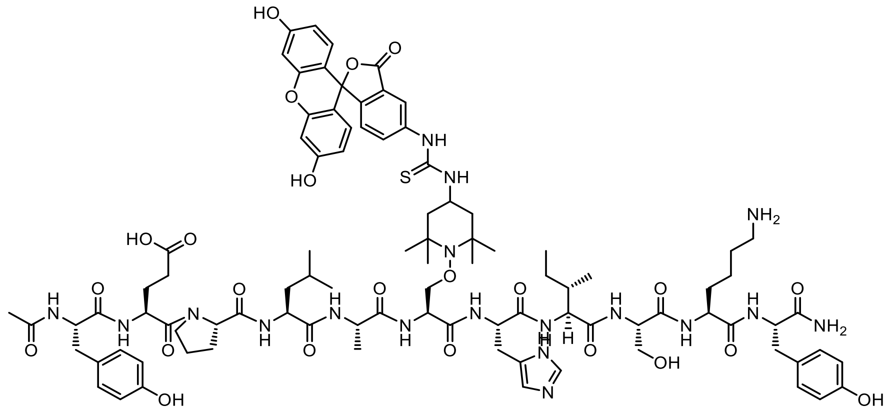

Synthesized following general one-pot ligation-conjugation protocol using H-UHISKY-NH_2 (**24**) (2 mg, 2.51 μmol) and Ac-YEPLA-SePh (**23**) (2.04 mg, 2.64 μmol). Purified by semi-preparative RP-HPLC (15-80% B over 30 minutes) and lyophilised to yield the desired modified peptide (3.3 mg, 1.74 μmol, 69% yield).

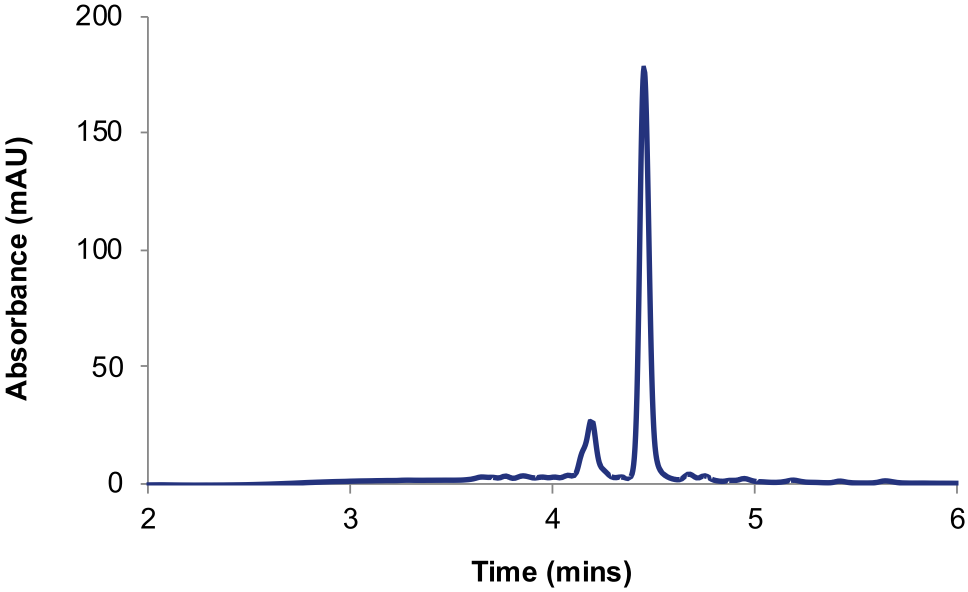

Figure S72. Analytical HPLC trace of 1 hour crude FITC-TEMPO modified Ac-YEPLAUHISKY-NH_2 from one-pot ligation-conjugation (**30**). Analytical gradient 10-100% B over 5 min, 280 nm.

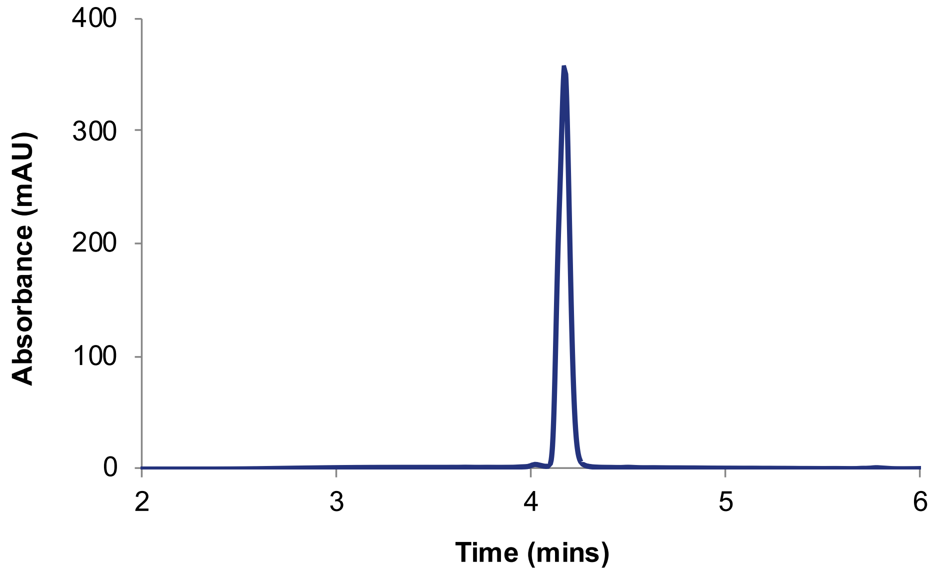

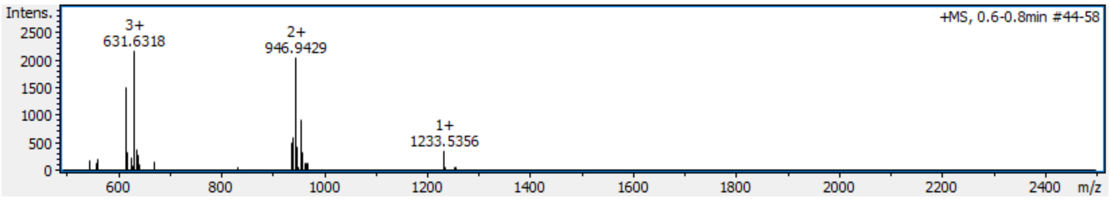

Figure S73. Analytical HPLC trace of pure FITC-TEMPO modified Ac-YEPLAUHISKY-NH_2 from one-pot ligation-conjugation (**30**). Analytical gradient 10-100% B over 5 min, 280 nm. Calculated Mass $[M+2H]^{2+}$: 946.4404, $[M+3H]^{3+}$: 631.2962. Observed Mass $[M+2H]^{2+}$: 946.9429, $[M+3H]^{3+}$: 631.6318.

Gemcitabine-TEMPO Conjugate (31)

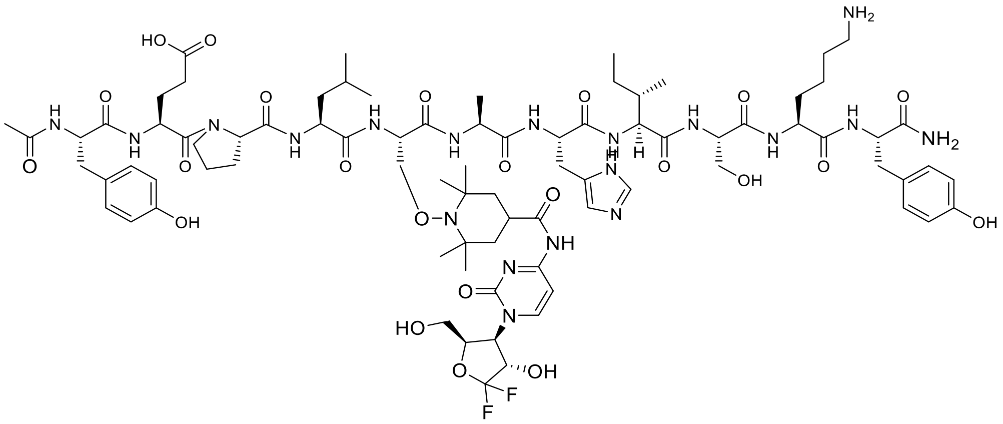

Synthesized following general one-pot ligation-conjugation protocol using H-UHISKY-NH_2 (**24**) (2 mg, 2.51 μmol) and Ac-YEPLA-SePh (**23**) (2.04 mg, 2.64 μmol). Purified by semi-preparative RP-HPLC (15-80% B over 30 minutes) and lyophilised to yield the desired modified peptide (3.5 mg, 1.97 μmol, 78% yield).

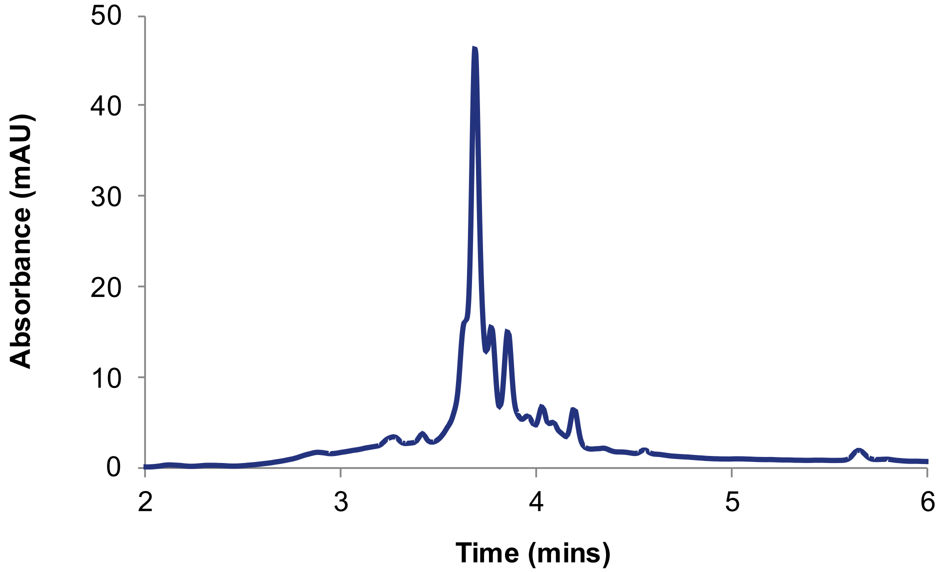

Figure S74. Analytical HPLC trace of 1 hour crude gemcitabine-TEMPO modified Ac-YEPLAUHISKY-NH_2 from one-pot ligation-conjugation (**31**). Analytical gradient 10-100% B over 5 min, 210 nm.

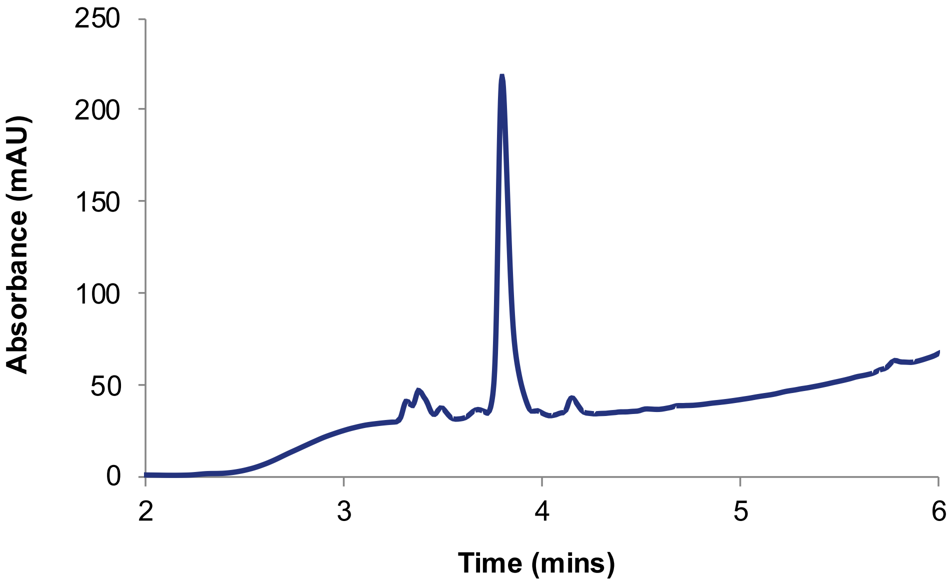

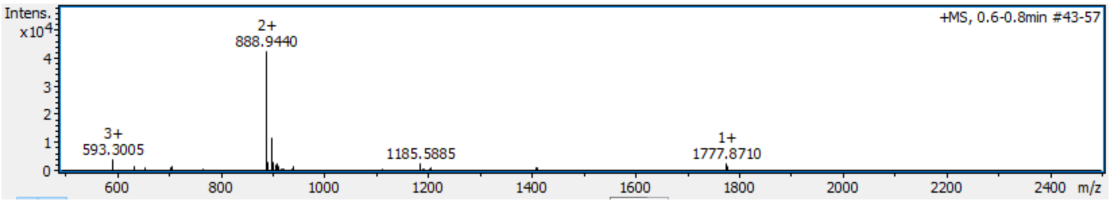

Figure S75. Analytical HPLC trace of pure gemcitabine-TEMPO modified Ac-YEPLAUHISKY-NH_2 from one-pot ligation-conjugation (**31**). Analytical gradient 10-100% B over 5 min, 210 nm. Calculated Mass $[M+2H]^{2+}$: 888.9425. Observed Mass $[M+2H]^{2+}$: 888.9440.

Ac-YEPLA-SePh (23) and H-UHISCY-NH2 (25) One-pot Ligation and Conjugation (32 - 34)

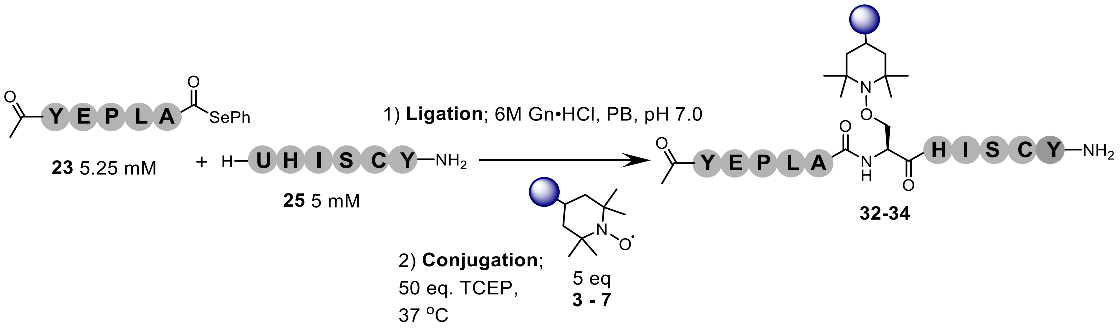

Crude ligation product (59) of Ac-YEPLA-SePh (23) and H-UHISCY-NH2 (25)

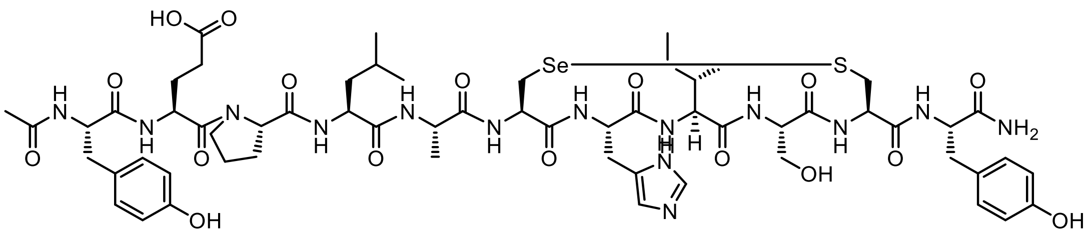

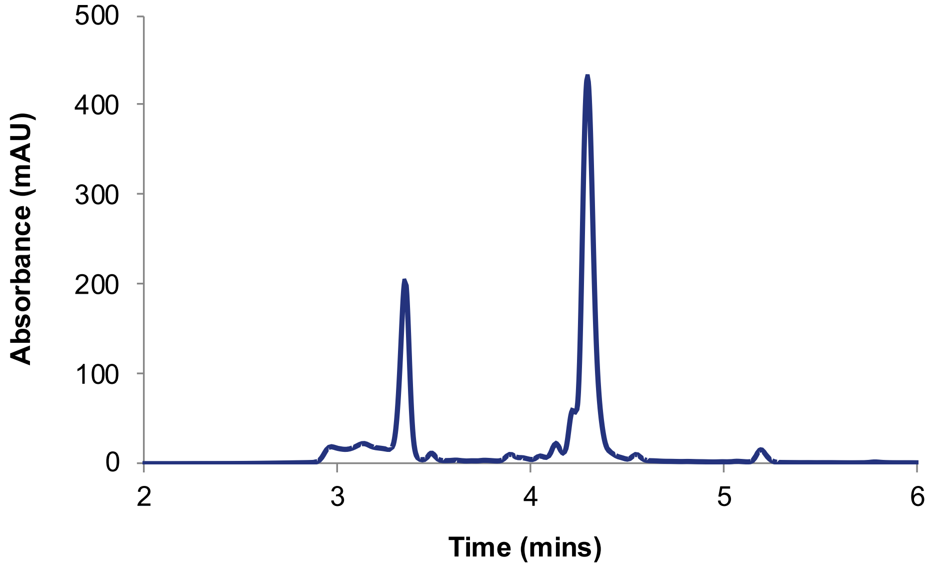

Figure S76. Analytical HPLC trace of crude Ac-YEPLA-SePh and H-UHISCY-NH2 ligation. Analytical gradient 10-100% B over 5 min, 210 nm.

TEMPO Conjugate (32)

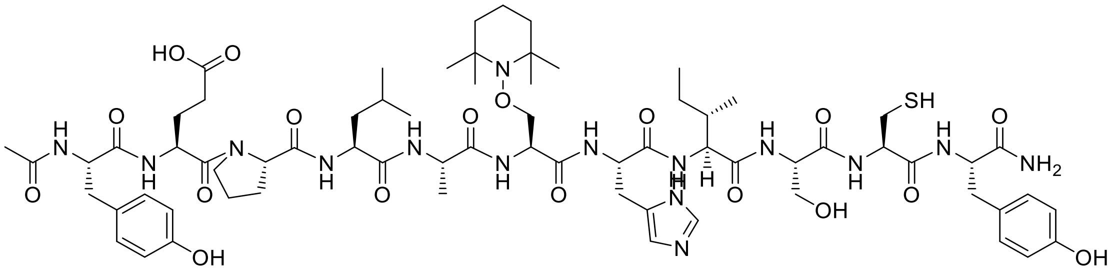

Synthesized following general one-pot ligation-conjugation protocol using H-UHISCY-NH_2 (**25**) (2.05 mg, 2.66 μmol) and Ac-YEPLA-SePh (**23**) (2.16 mg, 2.79 μmol). Purified by semi-preparative RP-HPLC (10-80% B over 30 minutes) and lyophilised to yield the desired modified peptide (3.5 mg, 2.39 μmol, 90% yield).

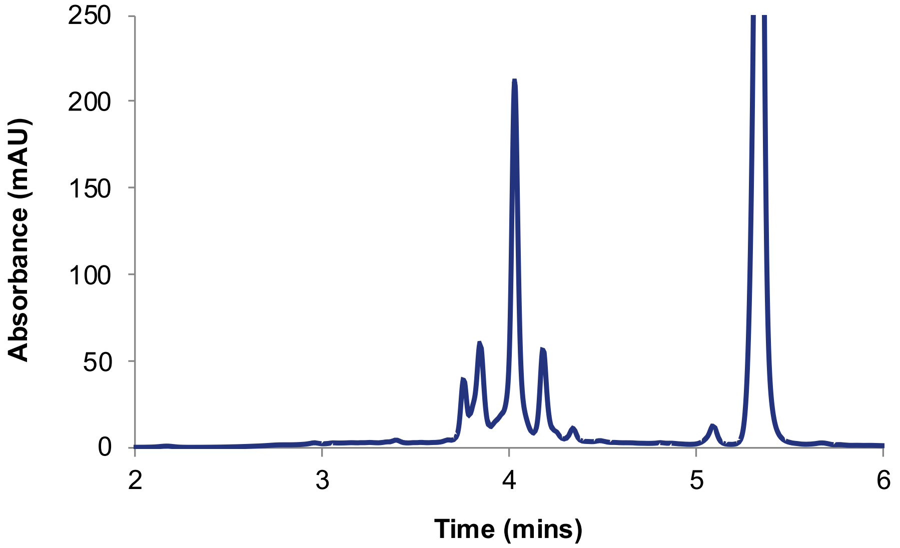

Figure S77. Analytical HPLC trace of 4 hours crude TEMPO modified Ac-YEPLAUHISCY-NH_2 from one-pot ligation-conjugation (**32**). Analytical gradient 10-100% B over 5 min, 210 nm.

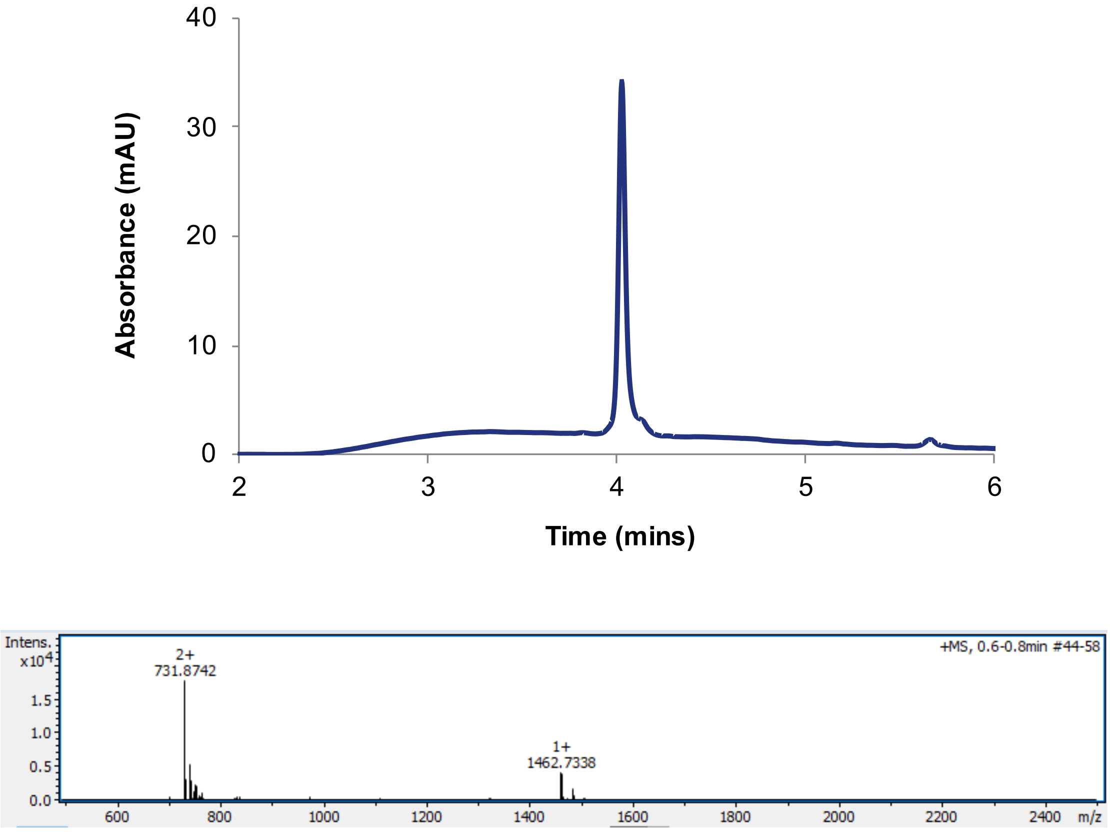

Figure S78. Analytical HPLC trace of pure TEMPO modified Ac-YEPLAUHISCY-NH_2 from one-pot ligation-conjugation (**32**). Analytical gradient 10-100% B over 5 min, 210 nm. Calculated Mass $[M+H]^+$: 1462.7405, $[M+2H]^{2+}$: 731.8740. Observed Mass $[M+H]^+$: 1462.7338, $[M+2H]^{2+}$: 731.8742.

Propargyl-TEMPO Conjugate (33)

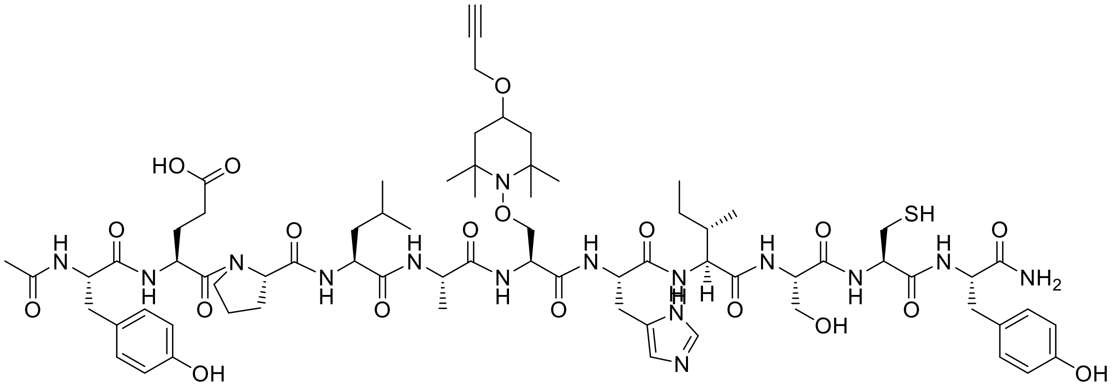

Synthesized following general one-pot ligation-conjugation protocol using H-UHISCY-NH_2 (**25**) (2.05 mg, 2.66 µmol) and Ac-YEPLA-SePh (**23**) (2.16 mg, 2.79 µmol). Purified by semi-preparative RP-HPLC (10-80% B over 30 minutes) and lyophilised to yield the desired modified peptide (3.3 mg, 2.20 µmol, 83% yield).

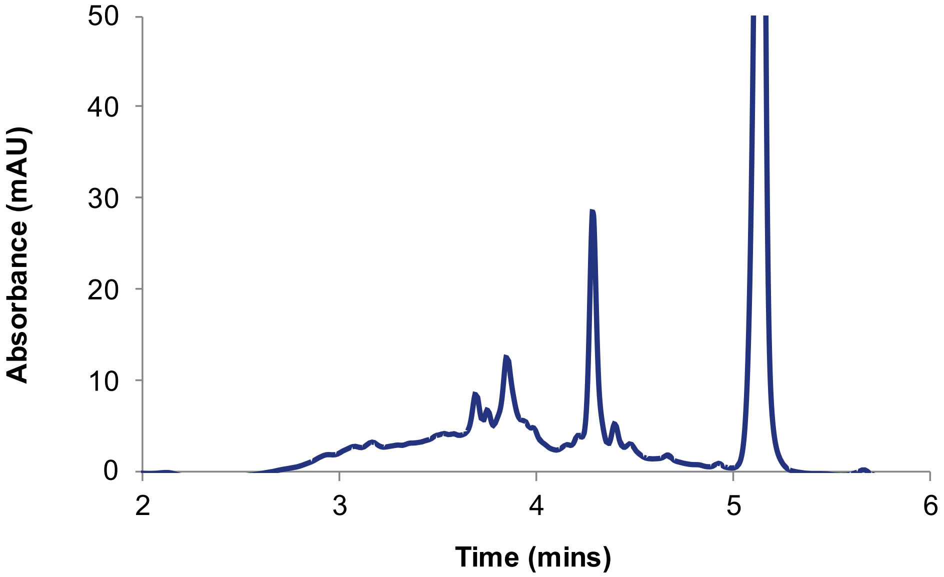

Figure S79. Analytical HPLC trace of 4 hours crude propargyl-TEMPO modified Ac-YEPLAUHISCY-NH_2 from one-pot ligation-conjugation (**33**). Analytical gradient 10-100% B over 5 min, 210 nm.

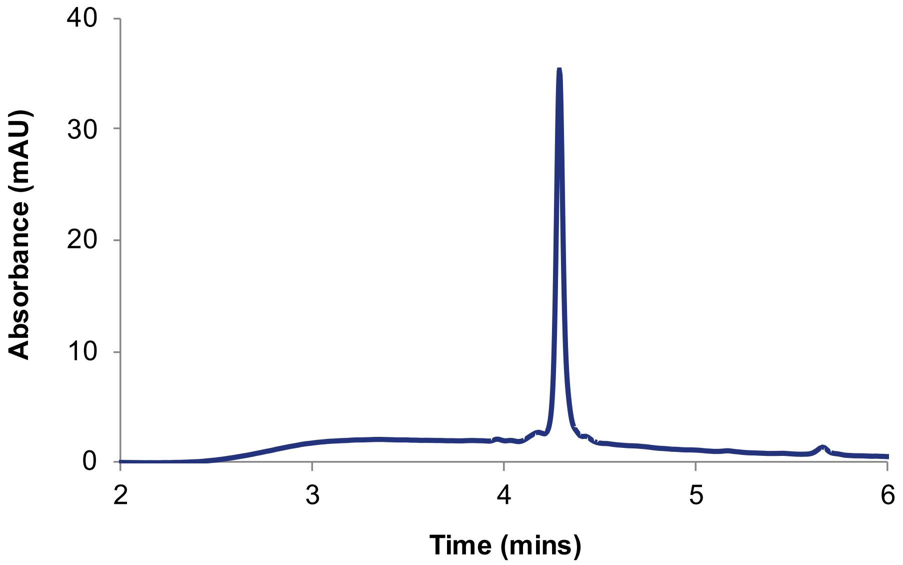

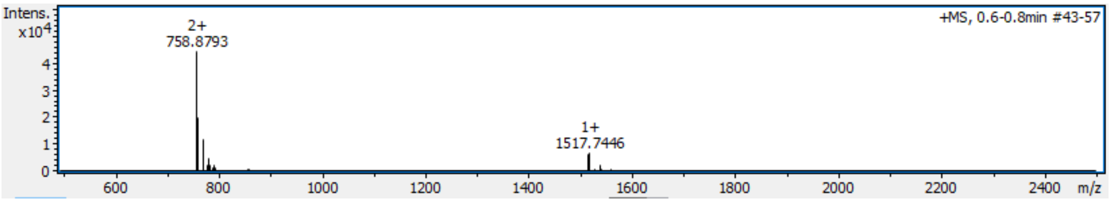

Figure S80. Analytical HPLC trace of pure propargyl-TEMPO modified Ac-YEPLAUHISCY-NH_2 from one-pot ligation-conjugation (**33**). Analytical gradient 10-100% B over 5 min, 210 nm. Calculated Mass $[M+H]^+$: 1517.7588, $[M+2H]^{2+}$: 759.3833. Observed Mass $[M+H]^+$: 1517.7446, $[M+2H]^{2+}$: 758.8793.

Biotin-EDA-TEMPO (34)

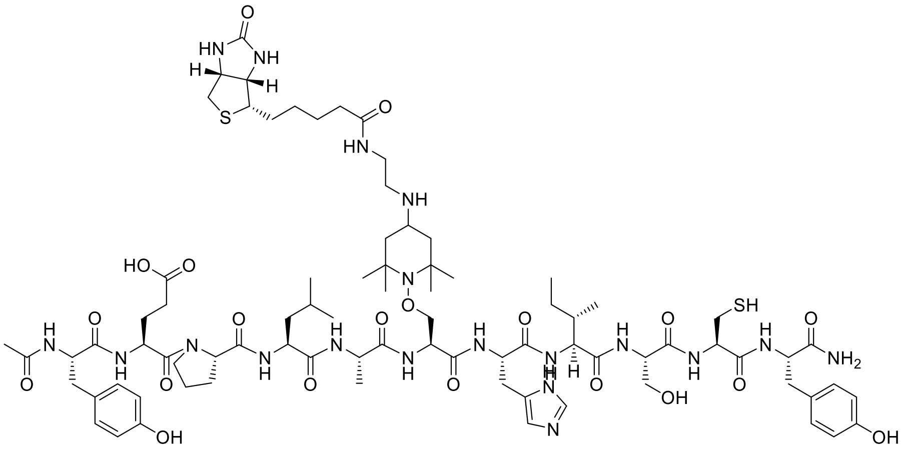

Synthesized following general one-pot ligation-conjugation protocol using H-UHISCY-NH_2 (**25**) (2.05 mg, 2.66 μmol) and Ac-YEPLA-SePh (**23**) (2.16 mg, 2.79 μmol). Purified by semi-preparative RP-HPLC (2-70% B over 30 minutes) and lyophilised to yield the desired modified peptide (3.3 mg, 1.89 μmol, 71% yield).

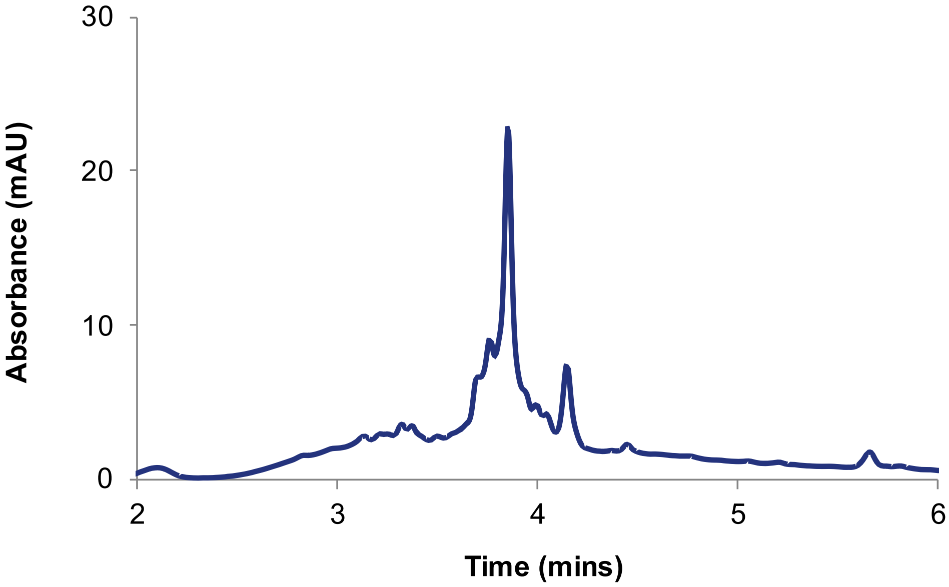

Figure S81. Analytical HPLC trace of 4 hours crude biotin-EDA-TEMPO modified Ac-YEPLAUHISCY-NH_2 from one-pot ligation-conjugation (**34**). Analytical gradient 10-100% B over 5 min, 210 nm.

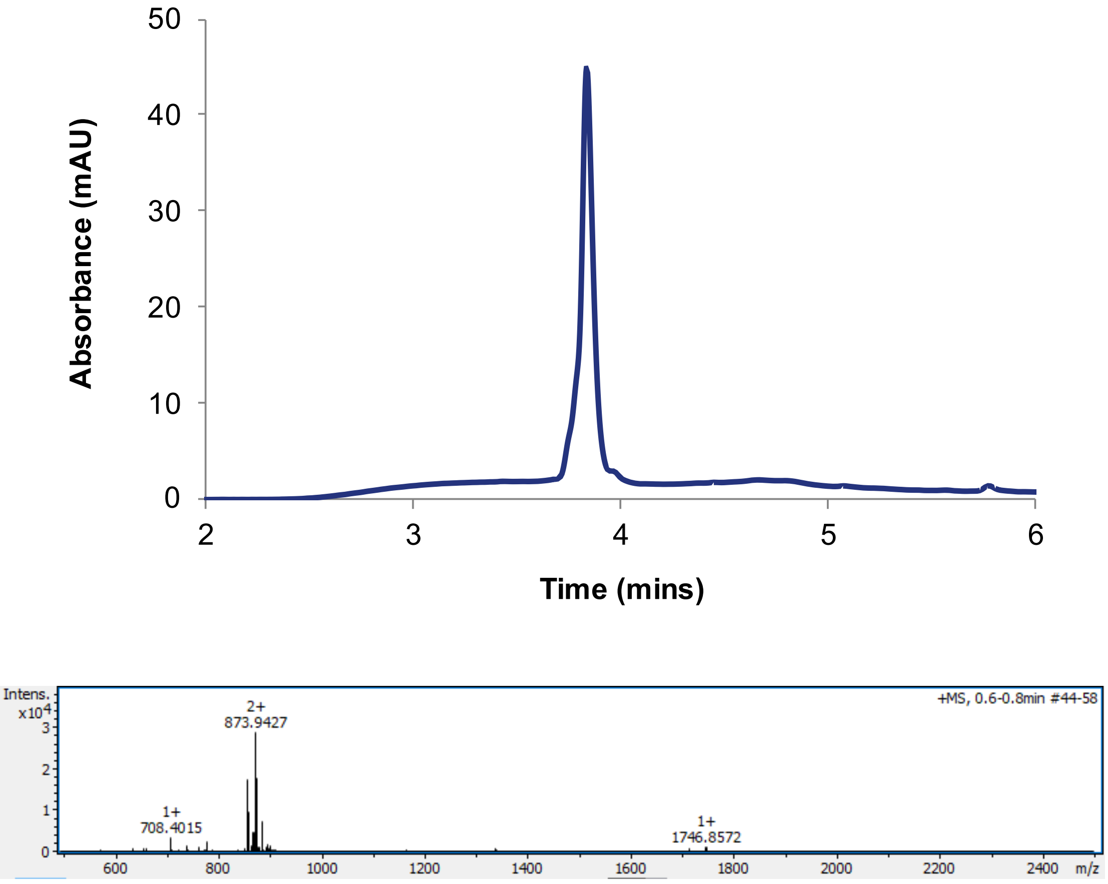

Figure S82. Analytical HPLC trace of pure biotin-EDA-TEMPO modified Ac-YEPLAUHISCY-NH_2 from one-pot ligation-conjugation (**34**). Analytical gradient 10-100% B over 5 min, 210 nm. Calculated Mass $[M+H]^+$: 1746.8711, $[M+2H]^{2+}$: 873.9395. Observed Mass $[M+H]^+$: 1746.8572, $[M+2H]^{2+}$: 873.9427.

Ac-YEPLA-SePh (23) and H-CHISKY-NH$_2$ (26) Two-step Ligation and Conjugation (28 and 31)

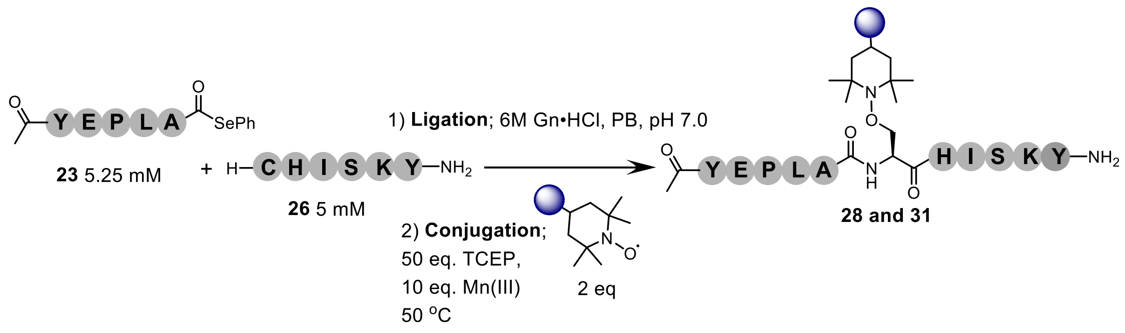

Ligation (35)

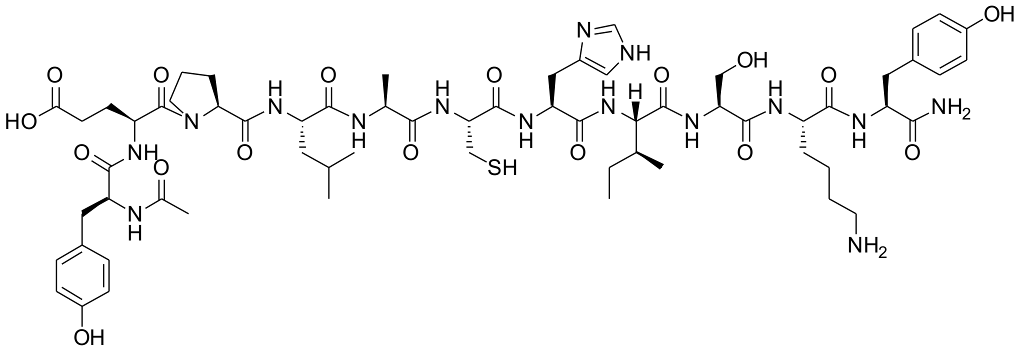

Synthesized following the general ligation protocol for N-terminal Cys peptides using H-CHISKY-NH$_2$ (**26**) (2.3 mg, 3.07 µmol) and Ac-YEPLA-SePh (**23**) (2.49 mg, 3.22 µmol) followed by purification using semi-preparative RP-HPLC (15-80% B over 30 minutes) and lyophilisation to produce the desired ligated peptide (3.2 mg, 2.35 µmol, 77% yield).

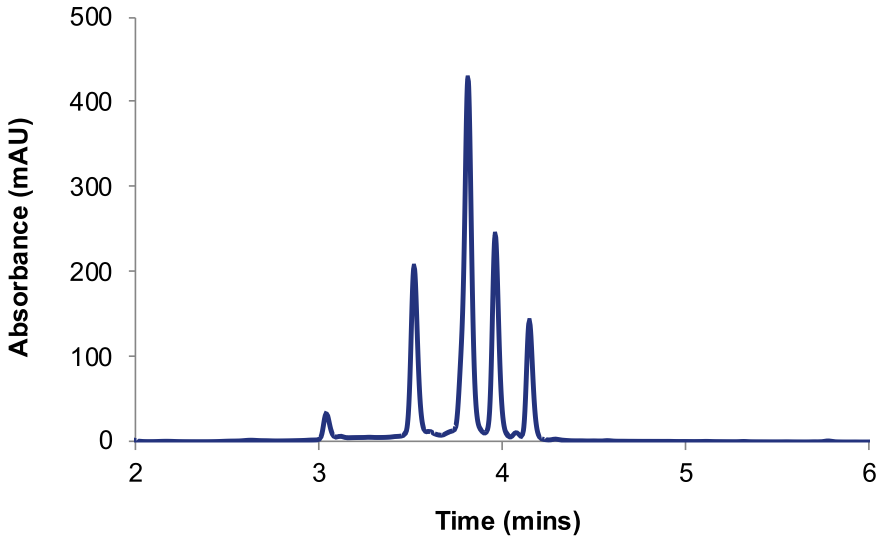

Figure S83. Analytical HPLC trace of crude ligation mixture after 10 minutes. Analytical gradient 10-100% B over 5 min, 280 nm.

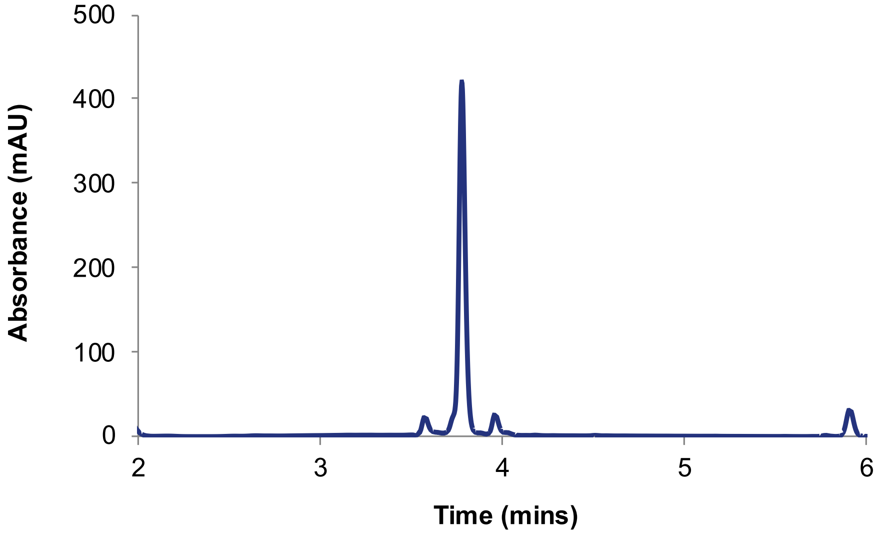

Figure S84. Analytical HPLC trace of crude ligation after addition of NH_2OH (10 eq). Analytical gradient 10-100% B over 5 min, 280 nm.

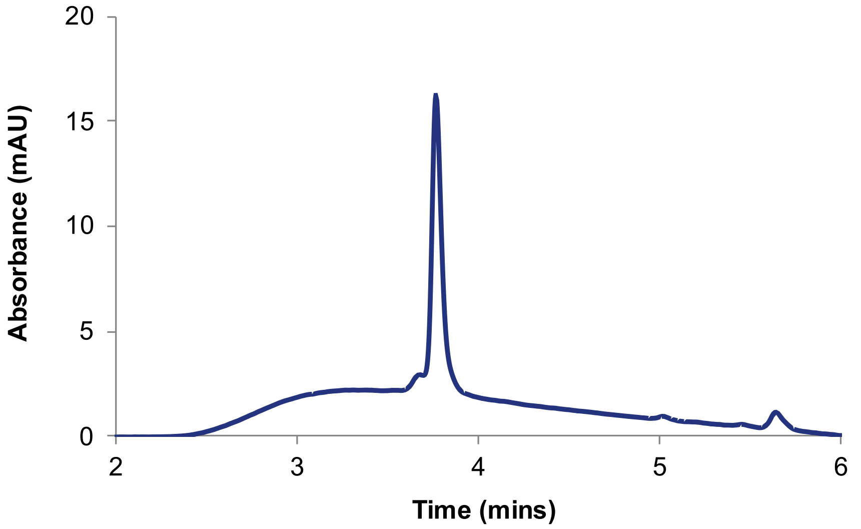

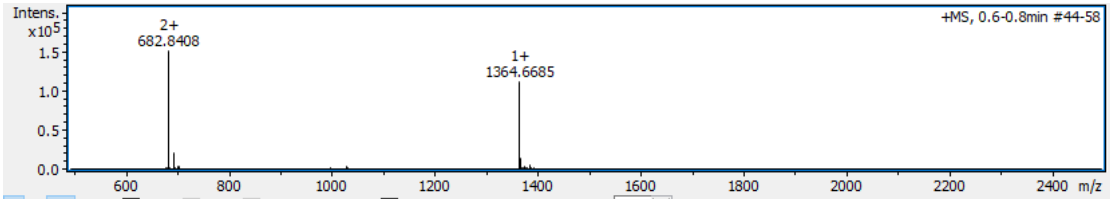

Figure S85. Analytical HPLC trace of purified ligated product (**35**). Analytical gradient 10-100% B over 5 min, 280 nm. Calculated Mass $[M+H]^+$: 1364.6672, $[M+2H]^{2+}$: 682.8375. Observed Mass $[M+H]^+$: 1364.6685, $[M+2H]^{2+}$: 682.8408.

Propargyl-TEMPO Conjugate (28)

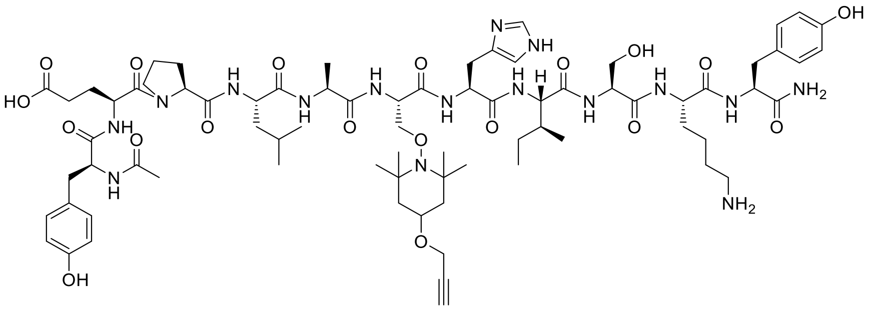

Synthesized following general protocol D using Ac-YEPLACHISKY-NH_2 (**35**) (4.0 mg, 2.93 μmol) followed by purification using semi-preparative RP-HPLC (15-80% B over 30 minutes) and lyophilisation to produce the desired conjugated peptide (3.0 mg, 1.95 μmol, 67% yield).

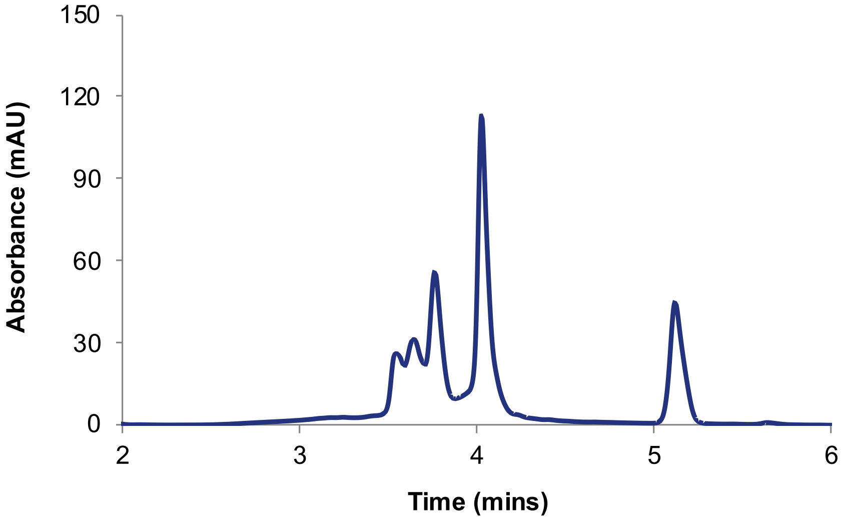

Figure S86. Analytical HPLC trace of 4 hours crude conjugation of Ac-YEPLACHISKY-NH_2 using propargyl-TEMPO (**28**). Analytical gradient 10-100% B over 5 min, 280 nm.

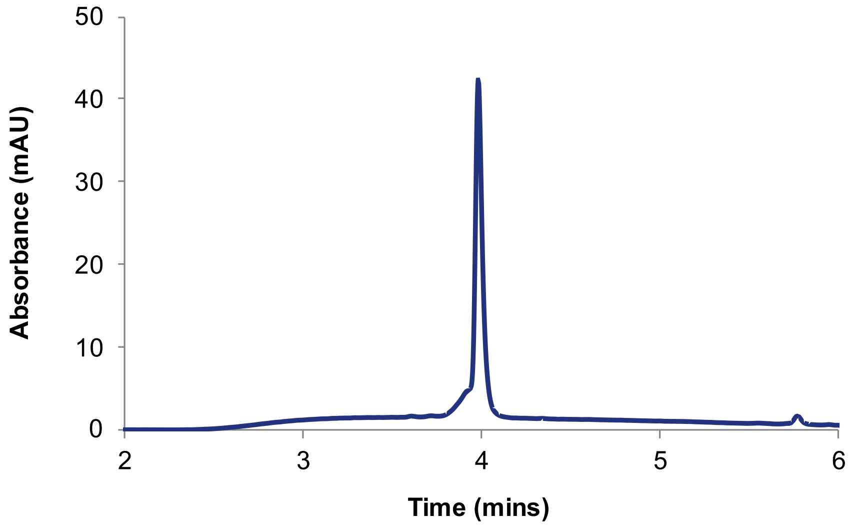

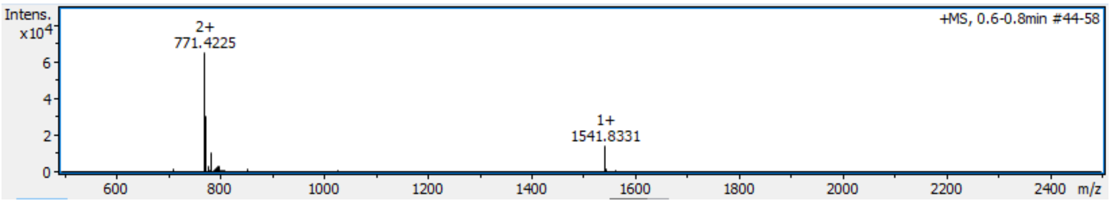

Figure S87. Analytical HPLC trace of pure conjugated Ac-YEPLACHISKY-NH_2 using propargyl-TEMPO (**28**). Analytical gradient 10-100% B over 5 min, 280 nm. Calculated Mass $[M+H]^+$: 1541.8367, $[M+2H]^{2+}$: 771.4223. Observed Mass $[M+H]^+$: 1541.8331, $[M+2H]^{2+}$: 771.4225.

Gemcitabine-TEMPO (31)

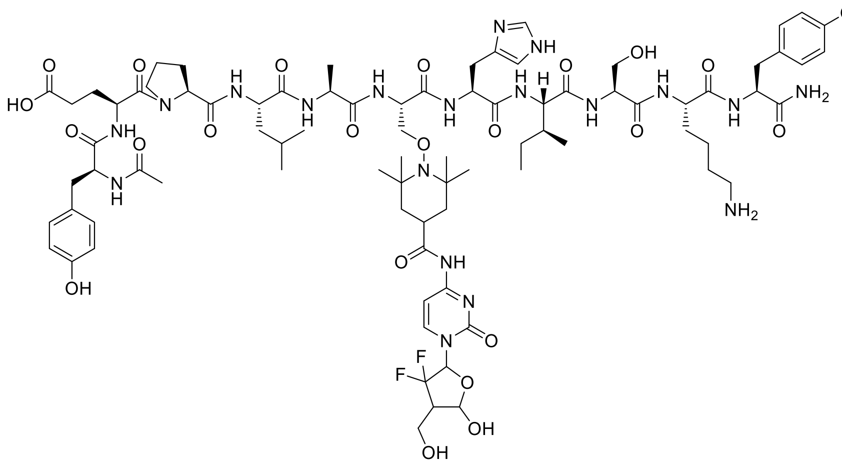

Synthesized following general protocol D using Ac-YEPLACHISKY-NH_2 (**35**) (4.0 mg, 2.93 μmol) followed by purification using semi-preparative RP-HPLC (15-80% B over 30 minutes) and lyophilisation to produce the desired conjugated peptide (3.2 mg, 1.81 μmol, 62% yield).

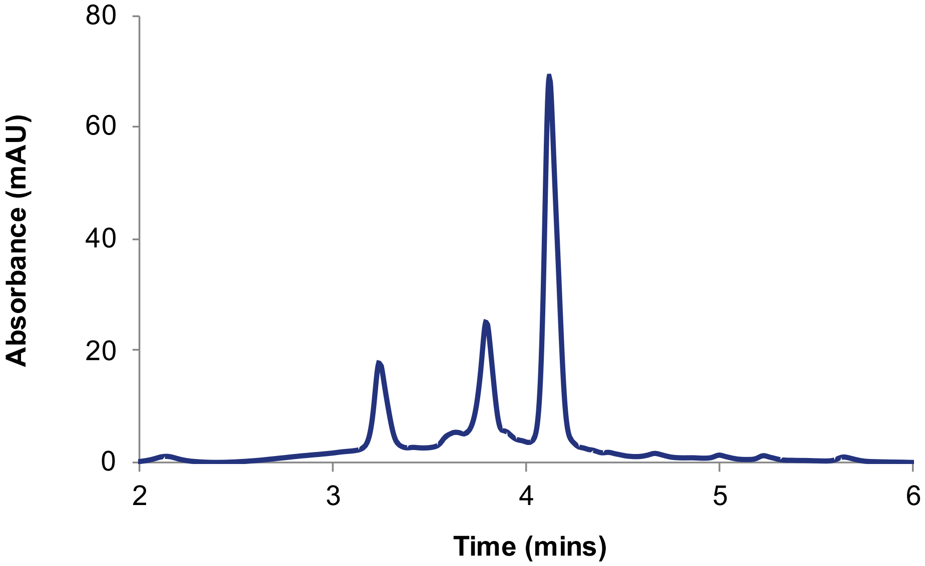

Figure S88. Analytical HPLC trace of 4 hours crude conjugation of Ac-YEPLACHISKY-NH_2 using gemcitabine-TEMPO (**31**). Analytical gradient 10-100% B over 5 min, 280 nm.

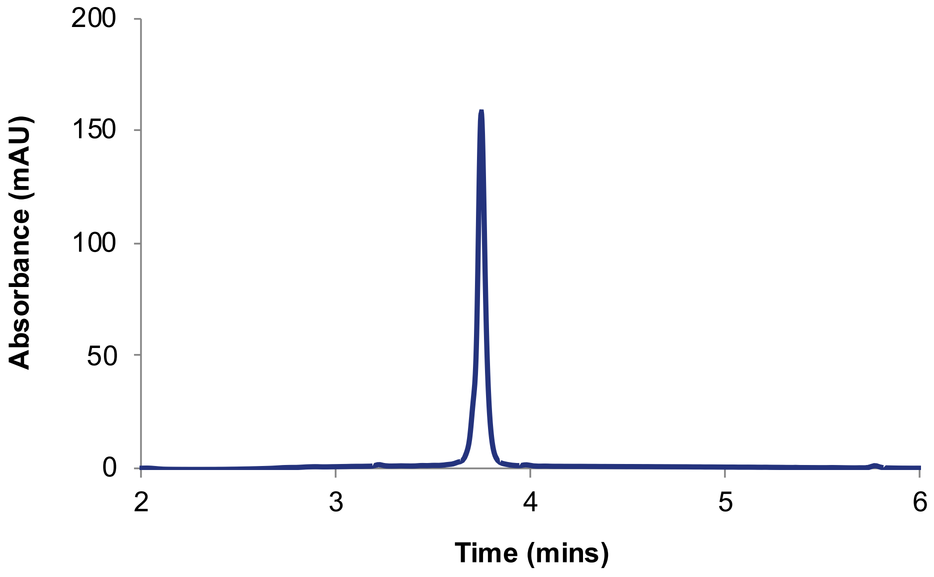

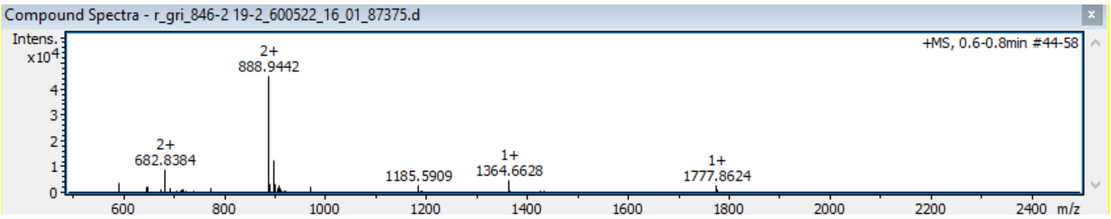

Figure S89. Analytical HPLC trace of pure conjugated of Ac-YEPLACHISKY-NH_2 using gemcitabine-TEMPO (**31**). Analytical gradient 10-100% B over 5 min, 280 nm. Calculated Mass $[M+2H]^{2+}$: 888.9426. Observed Mass $[M+2H]^{2+}$: 888.9442.

Reactions Showing Sec/Cys Selectivity

H-UHISCY-NH₂ Selective modification – Part 1 (36)

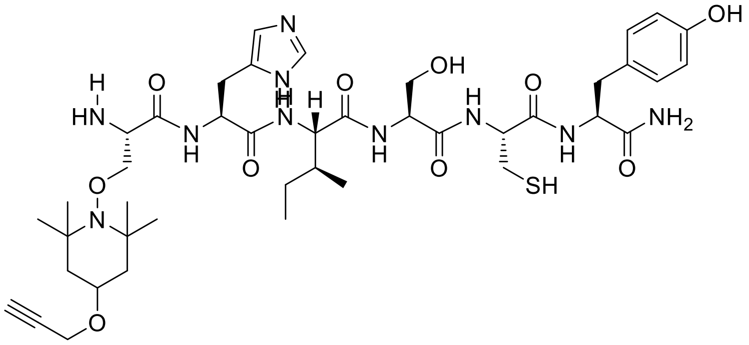

Synthesized following general protocol C using H-UHISCY-NH_2 (**25**) (3.0 mg, 3.89 μmol) followed by purification using semi-preparative RP-HPLC (15-80% B over 30 minutes) and lyophilisation to produce the desired conjugated peptide (2.4 mg, 2.66 μmol, 68% yield).

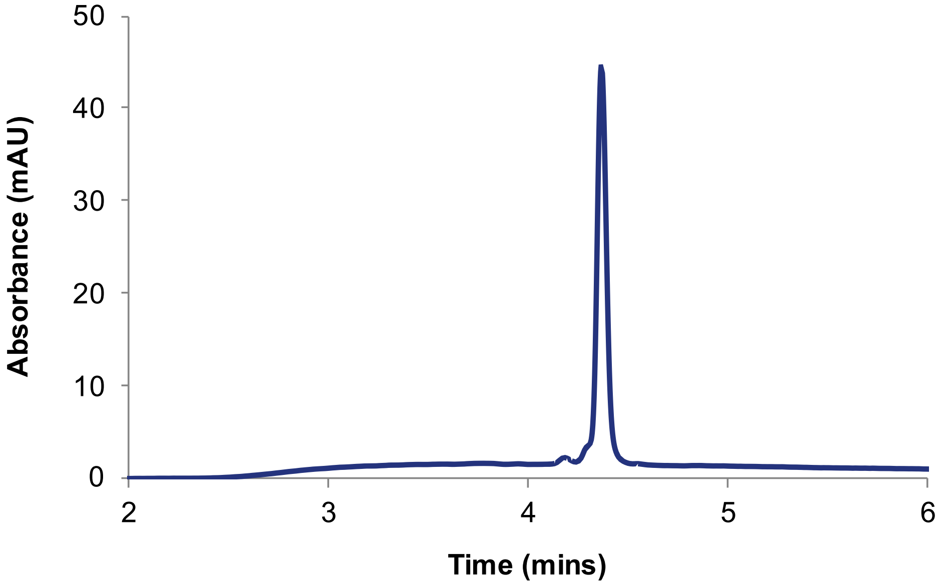

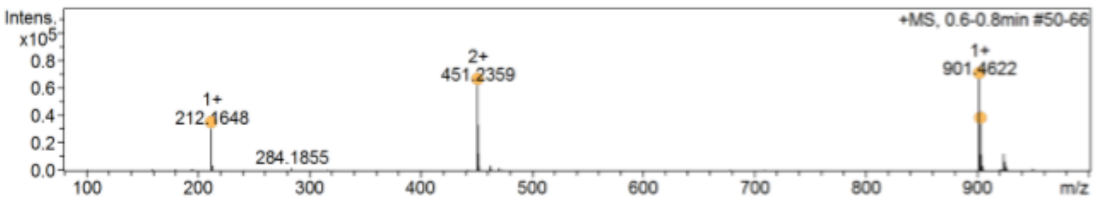

Figure S90. Analytical HPLC trace of pure conjugated H-UHISCY-NH_2 using propargyl-TEMPO (**36**). Analytical gradient 10-100% B over 5 min, 210 nm. Calculated Mass $[M+H]^+$: 901.4606, $[M+2H]^{2+}$: 451.2342. Observed Mass $[M+H]^+$: 901.4622, $[M+2H]^{2+}$: 451.2359.

H-UHISCY-NH$_2$ Selective modification – Part 2 (37)

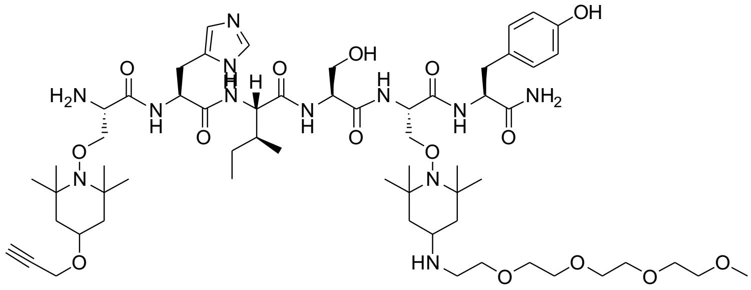

Synthesized following general protocol A using H-A(propargylTEMPO-HISCY-NH$_2$ (**36**) (2.4 mg, 2.66 µmol) followed by purification using semi-preparative RP-HPLC (10-80% B over 30 minutes) and lyophilisation to produce the desired conjugated peptide (2.1 mg, 1.71 µmol, 64% yield).

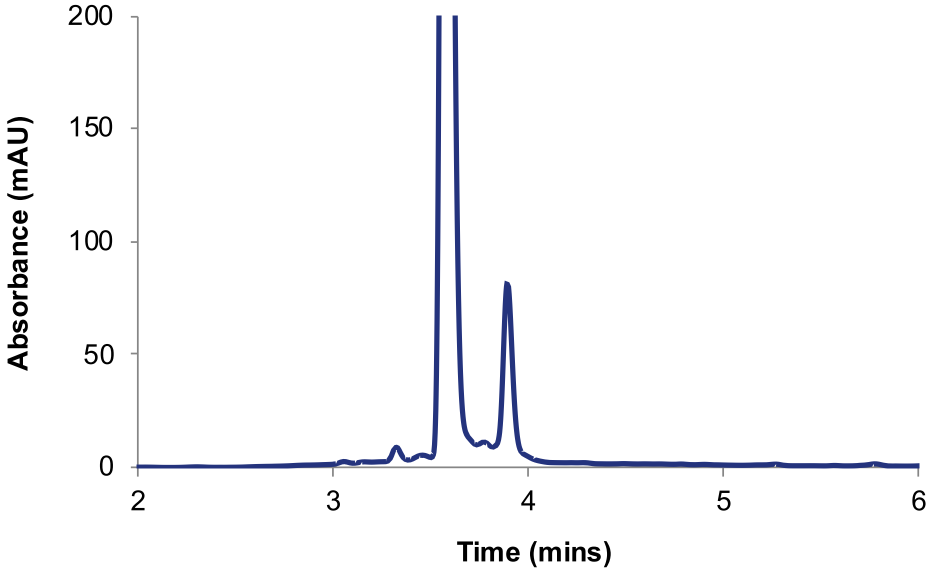

Figure S91. Analytical HPLC trace of 2 hours crude conjugation of **37** using tetra-EG-TEMPO (**37**). Analytical gradient 10-100% B over 5 min, 210 nm.

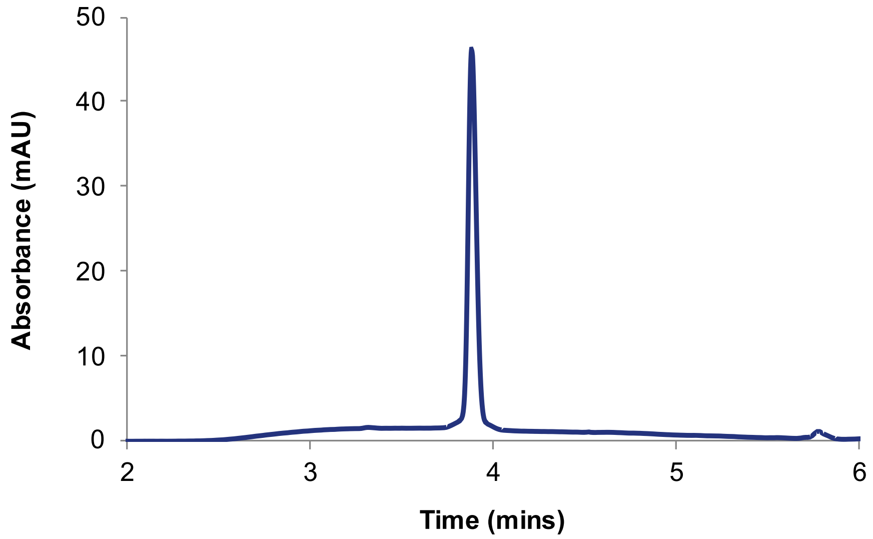

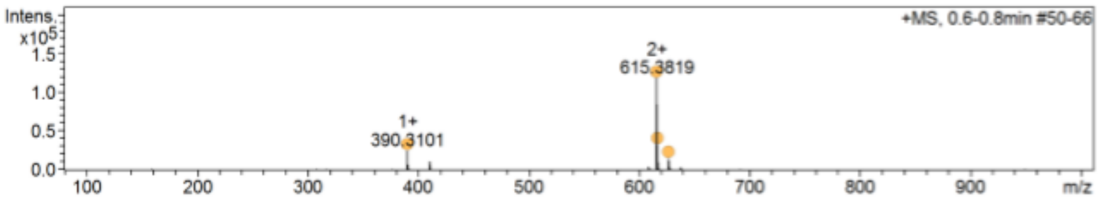

Figure S92. Analytical HPLC trace of pure conjugated **38** using tetra-EG-TEMPO. Analytical gradient 10-100% B over 5 min, 210 nm. Calculated Mass $[M+2H]^{2+}$: 615.3794. Observed Mass $[M+2H]^{2+}$: 615.3819.

Conjugate stability studies

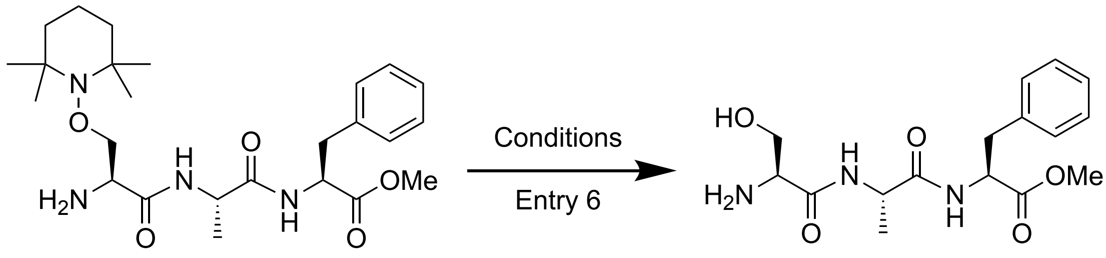

2a **60**

The TEMPO conjugate (**2a**) was dissolved in H_2O/MeOH 1:1 to a concentration of 12.5 mM, then diluted to a concentration of 2.5 mM with the reagent specified in the "Conditions" column of Table S15. For entries 1 and 2 a solution of 0.01 M HCl (pH 2) and 0.01 M NMM buffer (pH 9) was added to the conjugate stock respectively. The final pH of these was checked to be as specified in Table S12. For entry 6 the peptide was dissolved in H_2O: MeOH: AcOH 8:1:1 and powdered zinc was added directly to the peptide solution. The percentage degradation was based on comparing the starting material peak integration from HPLC analysis to any by-product peaks formed during the reaction.

Table S15. Conjugate stability studies on conjugated TEMPO product (**2a**).

**Entry**	**Conditions**	**Time/hr**	**% Degradation**
1	pH 2	16	0*
2	pH 9	16	0*
3	5 eq. GSH/DTT	16	0
4	50 °C	16	0
5	5 eq. V-50	16	0
6	50 eq. Zn/ 10% AcOH - 37 °C	16	100 (Conversion to Serine)

* Entry 1 and 2 showed methyl ester hydrolysis. Repeated on a larger model to ensure no degradation to TEMPO conjugate. Entry 6 was repeated on an isolatable scale to confirm findings.

H-A(TEMPO)HISKY-NH2 (38)

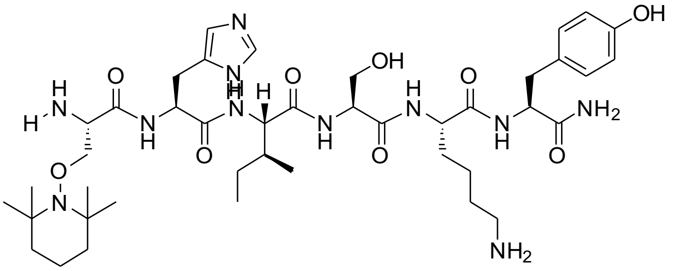

Synthesized using general protocol A using H-UHISKY-NH2 (**24**) (5 mg, 6.28 µmol). Purified by preparative RP-HPLC (10 to 80% B over 30 minutes) and lyophilized to produce the desired modified peptide (4.6 mg, 5.28 µmol, 84% yield).

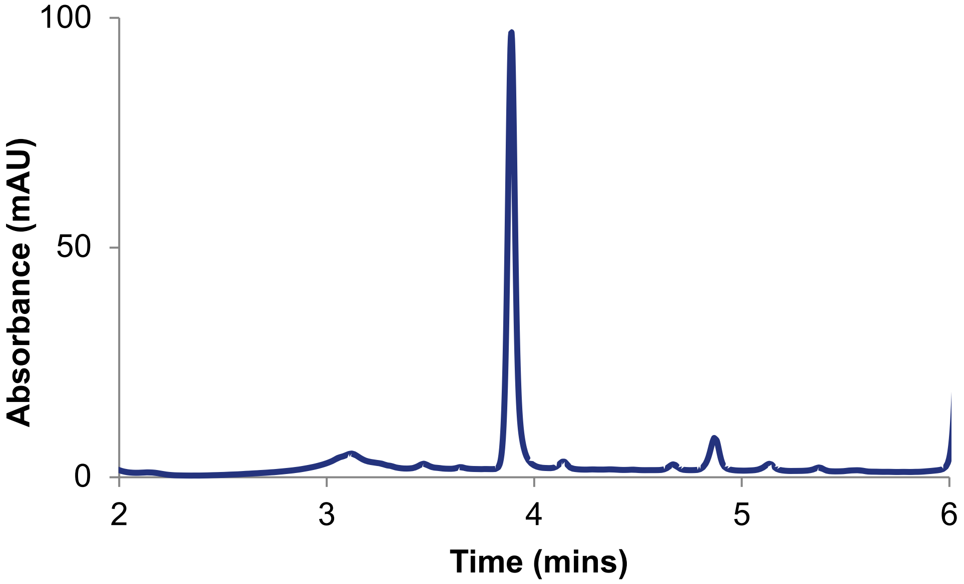

Figure S93. Analytical HPLC trace of crude TEMPO modified H-UHISKY-NH2 (**38**). Analytical gradient 10-70% B over 5 min, 280 nm.

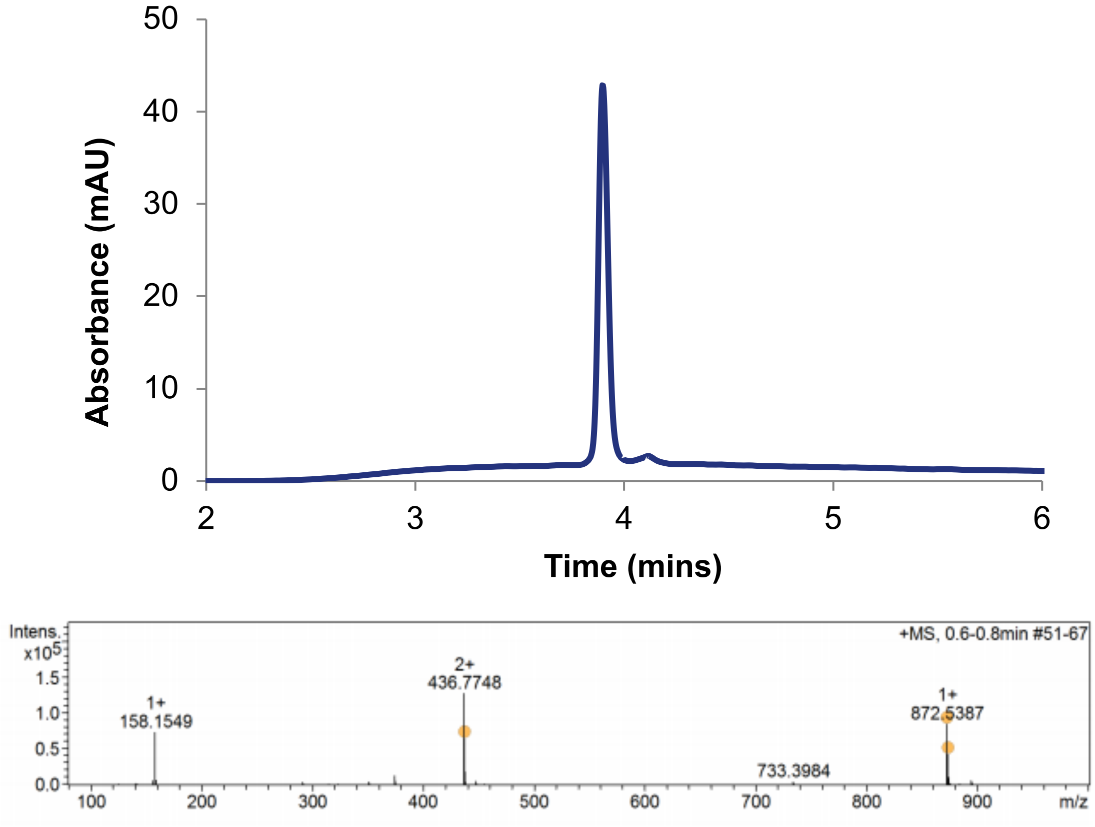

Figure S94. Analytical HPLC trace of pure TEMPO modified H-UHISKY-NH_2 (**38**). Analytical gradient 1070% B over 5 min, 280 nm. HRMS Calculated Mass $[M+H]^+$: 872.5358, $[M+2H]^{2+}$: 436.7718. Observed Mass $[M+H]^+$: 872.5387, $[M+2H]^{2+}$: 436.7748.

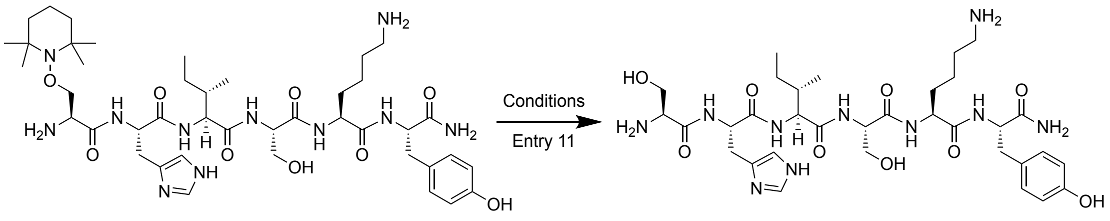

Table S16. Conjugate stability studies using H-A(TEMPO)HISKY-NH_2 (**38**) Model

Entry	**Conditions**	**Time/hr**	**% Degradation**
7	pH 2	16	0
8	pH 9	16	0
9	5 eq GSH	16	0
10	50 °C	16	0
11	254 nm	16	0
12	50 eq Zn/ 10% AcOH - 37 °C	16	100 (Conversion to Serine)

No degradation observed in entries 7 or 8, showing methyl ester hydrolysis in entries 1 and 2 was unrelated.

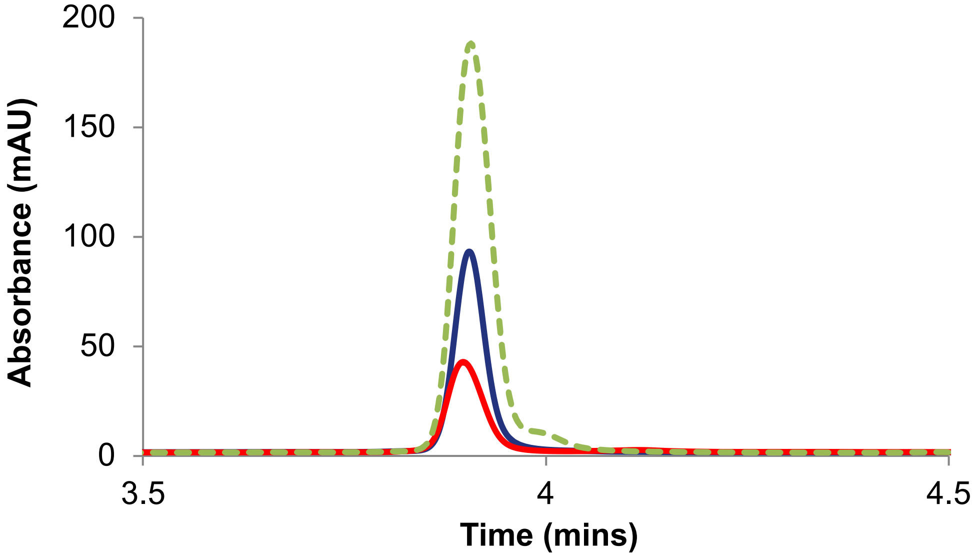

Figure S95. HPLC overlay comparing starting material (green dash), entry 7 (blue) and entry 8 (red), showing no degradation of TEMPO conjugate. Decrease in peak intensity not due to product degradation.

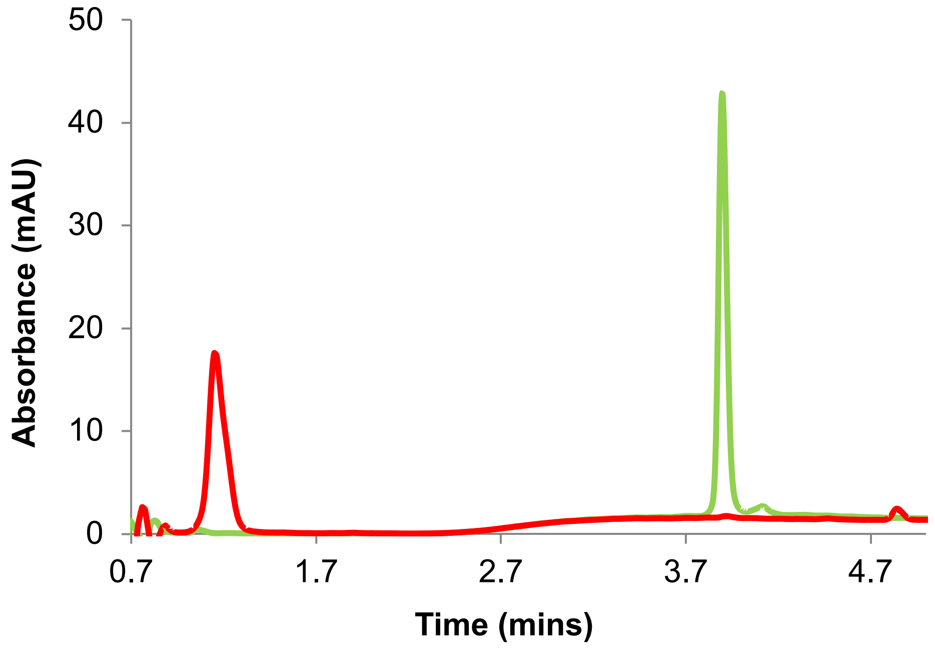

Figure S96. Entry 12 HPLC comparison of starting material (green) and reductively cleaved product (**61**) (red).

TEMPO conjugate reductive cleavage to Ser (60)

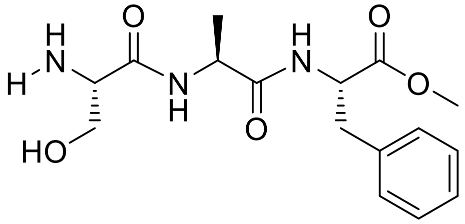

TEMPO conjugate H-(A(TEMPO)AF-OMe (**2a**) (5 mg, 0.0105 mmol) was dissolved in H_2O: MeOH: AcOH 8:1:1 (4.2 ml) to a concentration of 2.5 mM. Zinc dust (0.034 mg, 0.525 mmol) was added. The solution was stirred at 37 °C for 16 hours then purified by semi-preparative RP-HPLC (5-60% B over 30 minutes) and lyophilized to produce the desired peptide (3.4 mg, 0.010 mmol, 91% yield).^{1}H NMR (400 MHz, MeOD) δ 8.34 (d, *J* = 7.8 Hz, 1H), 7.34 – 7.16 (m, 5H), 4.71 – 4.59 (m, 1H), 4.41 (q, *J* = 7.2 Hz, 1H), 3.98 – 3.79 (m, 3H), 3.68 (s, 3H), 3.14 (dd, 1H), 3.00 (dd, *J* = 13.9, 8.3 Hz, 1H), 1.34 (d, *J* = 7.2 Hz, 3H). ^{13}C NMR (101 MHz, MeOD) δ 173.1, 171.8, 166.6, 136.6, 128.9, 128.1, 126.5, 60.4, 54.7, 54.0, 51.3, 49.0, 36.9, 16.7.

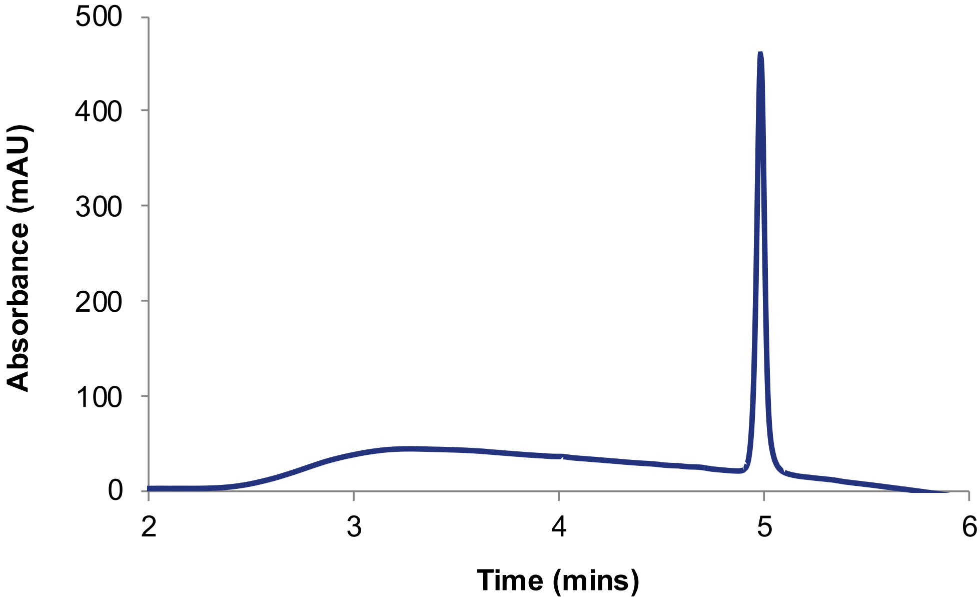

Figure S97. Analytical HPLC trace of H-A(TEMPO)AF-OMe crude with no Zn for 16 hours. Analytical gradient 10-70% B over 5 min, 210 nm.

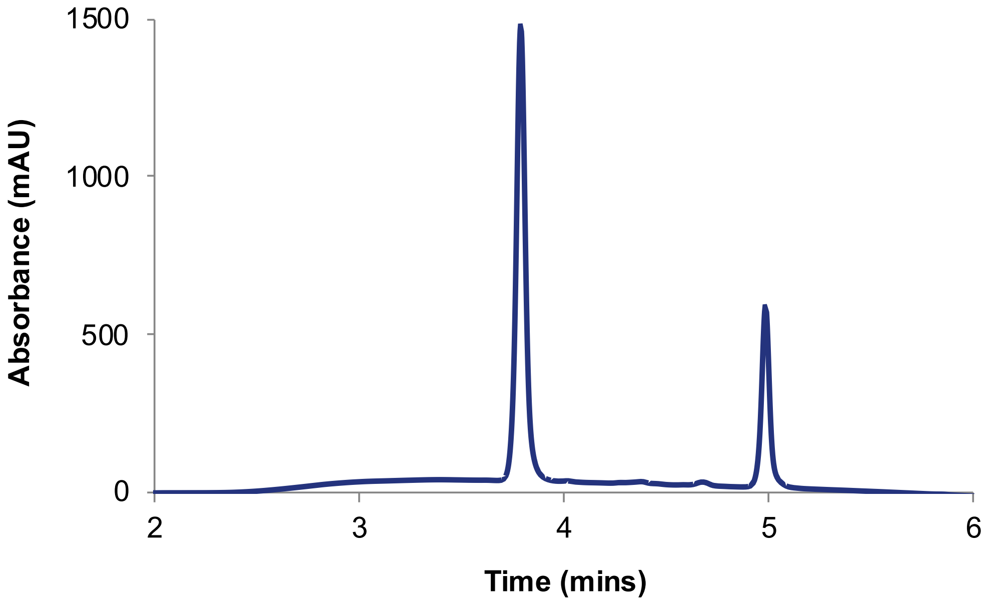

Figure S98. Analytical HPLC trace of H-A(TEMPO)AF-OMe crude with Zn for 1 hour. Analytical gradient 10-70% B over 5 min, 210 nm.

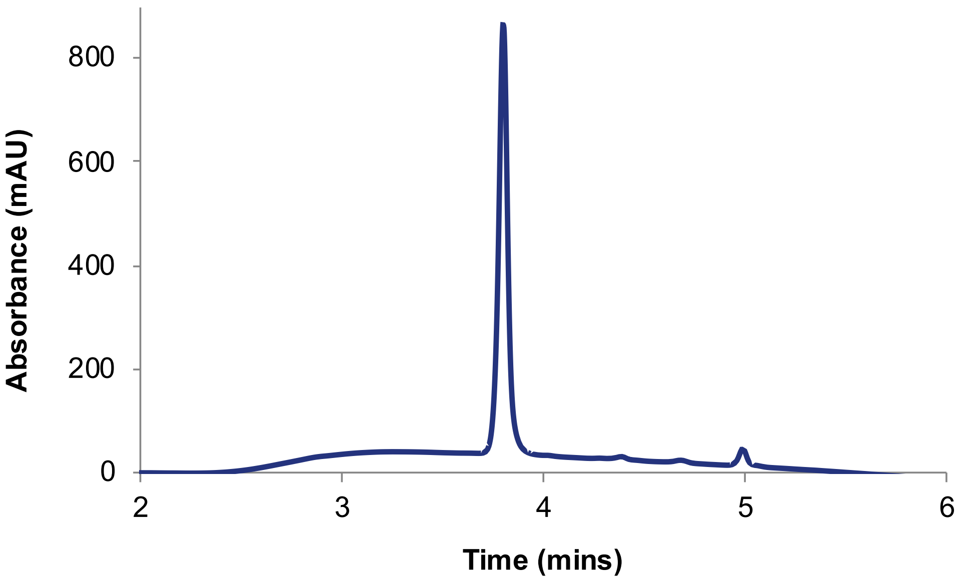

Figure S99. Analytical HPLC trace of H-A(TEMPO)AF-OMe crude with Zn for 16 hours. Analytical gradient 10-70% B over 5 min, 210 nm.

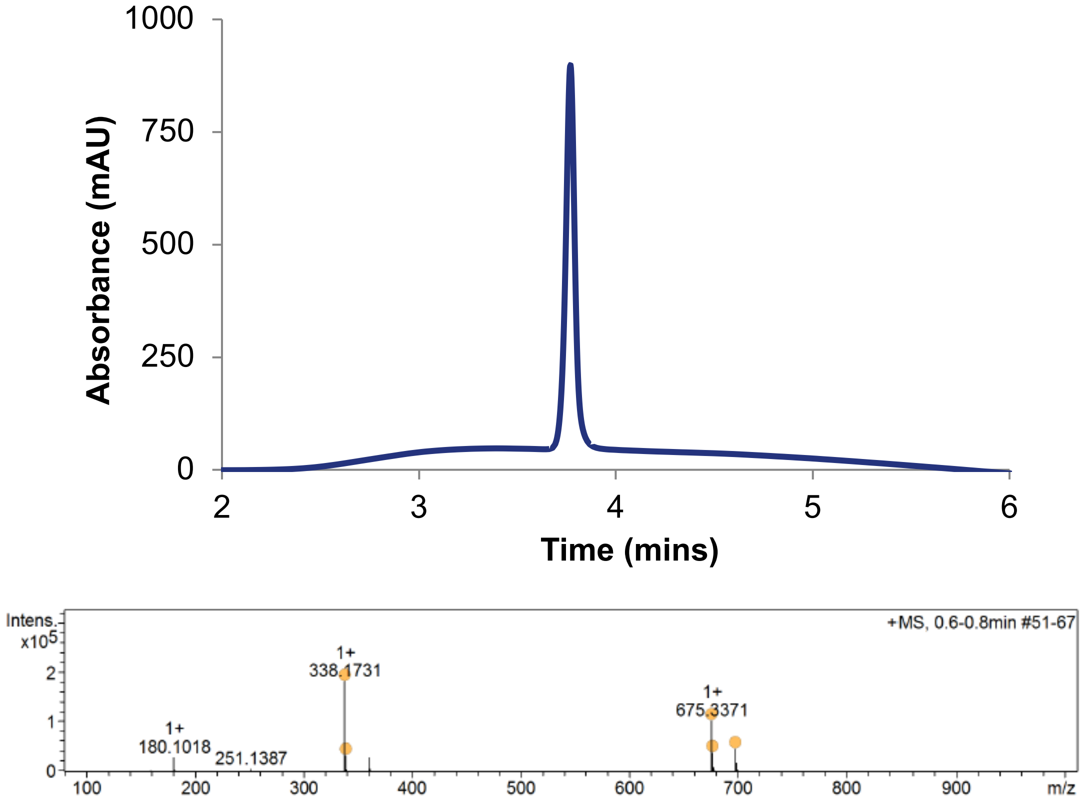

Figure S100. Analytical HPLC trace purified reductively cleaved product (**60**). Analytical gradient 10-70% B over 5 min, 210 nm. Calculated Mass $[M+H]^+$: 338.1716. Observed Mass $[M+H]^+$: 338.1731.

Ac-YEPLAA(propargyl-TEMPO)HISKY reductive cleavage (39)

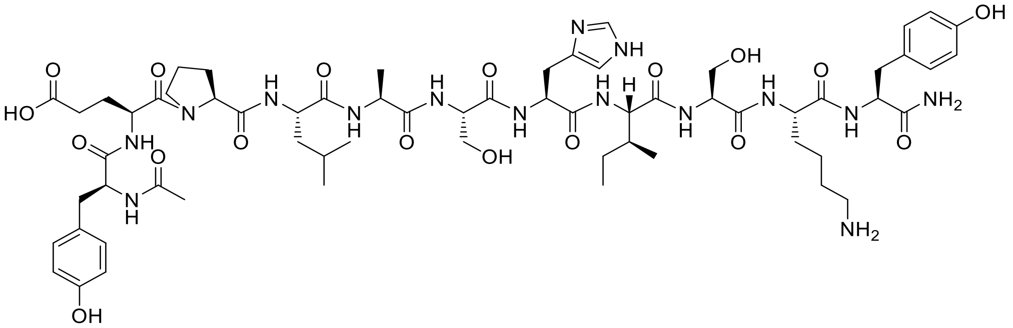

To conjugated product (**28**, 2.0 mg, 1.30 µmol) dissolved in H_2O: MeOH: AcOH 8:1:1 (0.52 ml, 2.5 mM peptide concentration) was added zinc powder (0.85 mg, 13.0 µmol, 10 eq). The reaction mixture was shaken at 37 °C for 16 hours before being purified by semi-preparative HPLC (5-60% B over 30 minutes) and lyophilized to produce the desired reductively cleaved peptide (1.6 mg, 1.19 µmol, 92% yield).

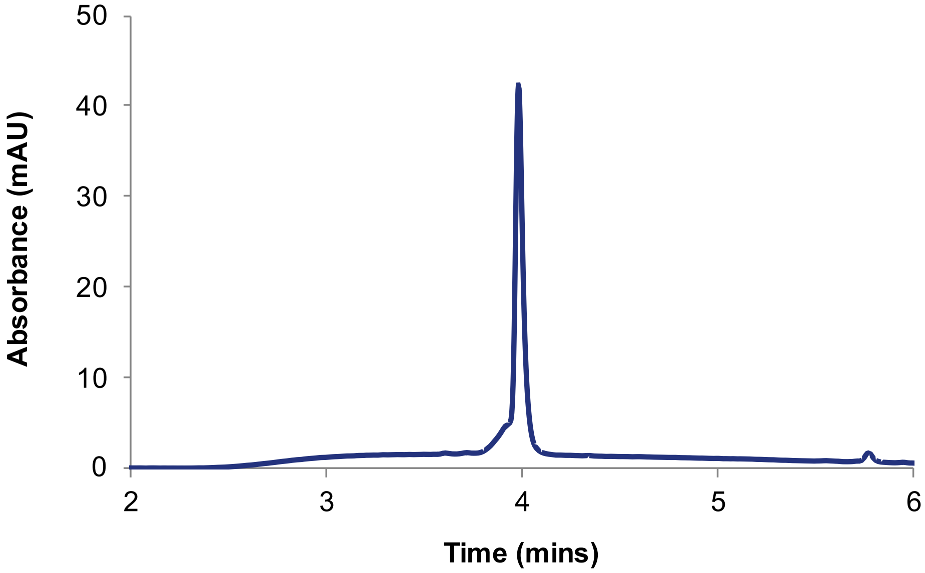

Figure S101. Analytical HPLC trace of **28**. Analytical gradient 10-100% B over 5 min, 280 nm.

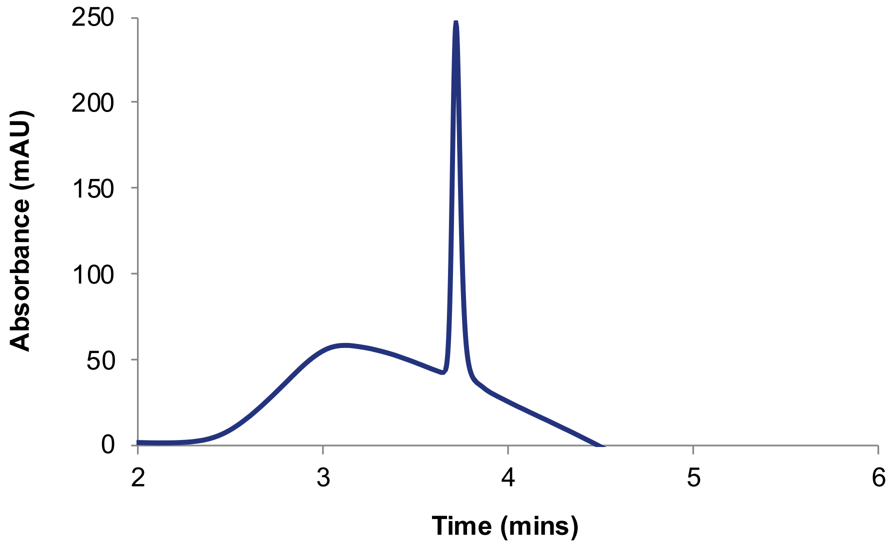

Figure S102. Analytical HPLC trace of 16 hours crude reaction mixture. Analytical gradient 10-100% B over 5 min, 280 nm.

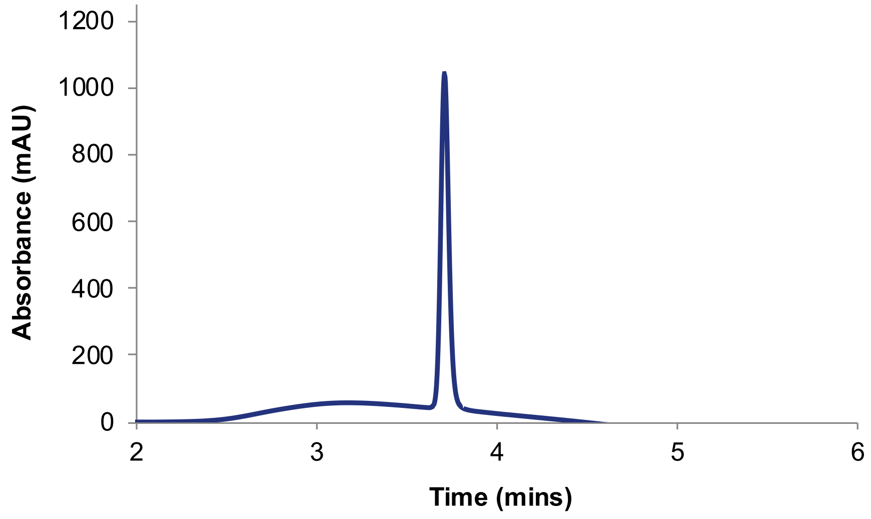

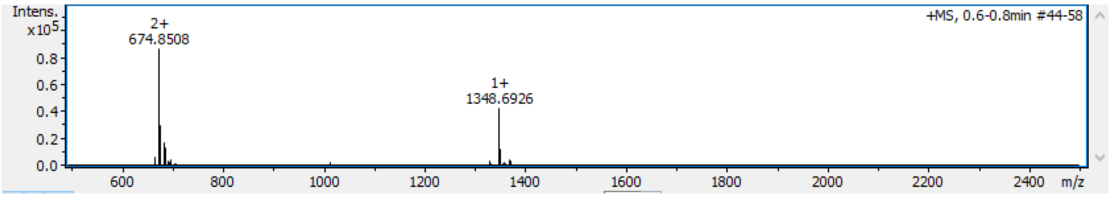

Figure S103. Analytical HPLC trace of pure reductively cleaved product (**39**). Analytical gradient 10-100% B over 5 min, 280 nm. Calculated Mass $[M+H]^+$: 1348.6901, $[M+2H]^{2+}$: 674.8490. Observed Mass $[M+H]^+$: 1348.6926, $[M+2H]^{2+}$: 674.8508.

Ac-YEPLAA(propargyl-TEMPO)HISKY-NH_2 control reductive cleavage – No Zinc

The same reaction conditions were applied to starting peptide **28**, without the addition of zinc as a control. The reaction mixture was shaken at 37 °C for 16 hours then analyzed by analytical RP-HPLC.

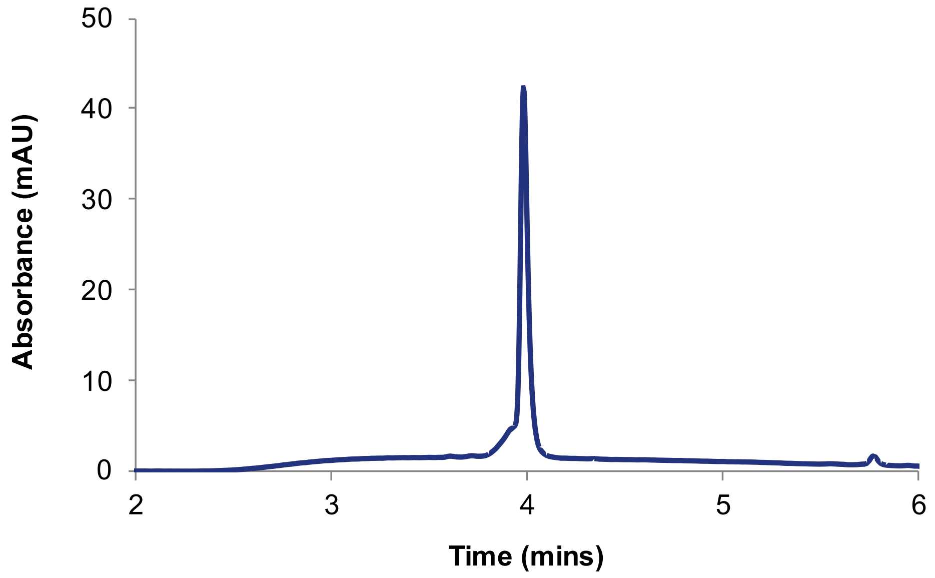

Figure S104. Analytical HPLC trace of 28. Analytical gradient 10-100% B over 5 min, 280 nm.

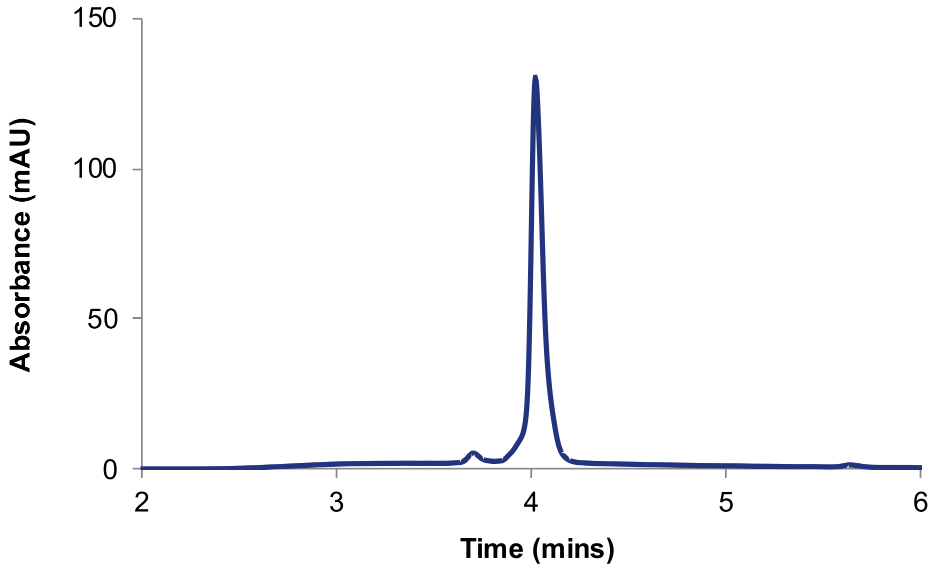

Figure S105. Analytical HPLC trace of 16 hours crude reaction mixture with no Zn added. Analytical gradient 10-100% B over 5 min.

One-pot ligation-conjugation-reductive cleavage – ligation at serine

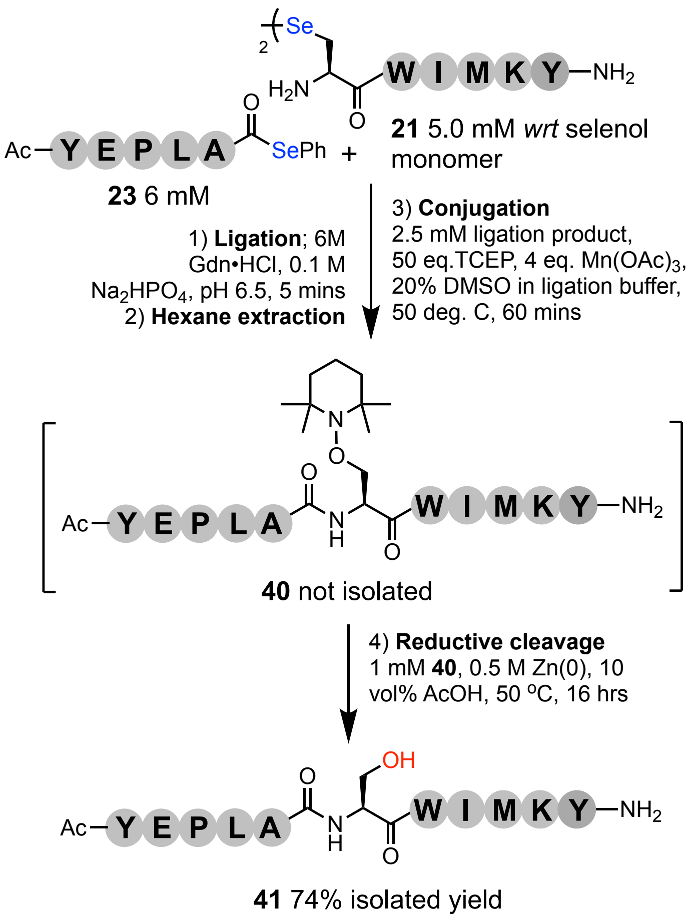

Synthesized following general one-pot ligation-conjugation protocol using H-UWIMKY-NH_2 (**21**) (1 mg, 1.12 µmol) and Ac-YEPLA-SePh (**23**) (1.04 mg, 1.35 µmol). To conjugation solution was added Zn dust (36.6 mg, 0.56 mmol) and glacial acetic acid and ligation buffer to a final composition of 10 vol% acetic acid, 1 mM wrt the conjugate product 40. The resulting solution was shaken at 50 °C for 16 hours before being purified by semi-preparative RP-HPLC (10-100% B over 30 minutes) and lyophilised to yield the desired peptide (1.2 mg, 0.83 µmol, 74% yield).

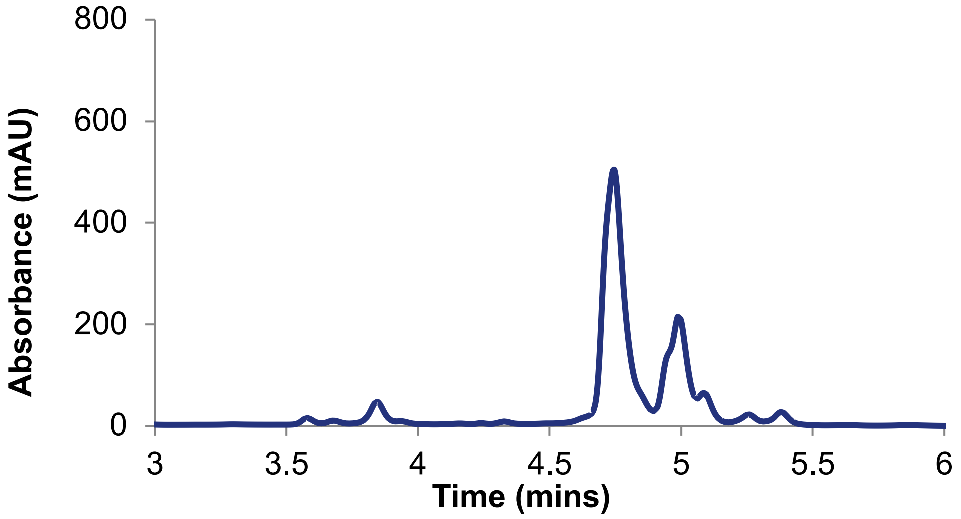

Figure S106. Analytical HPLC trace of crude ligation after 5 minutes. Analytical gradient 10-100% B over 5 minutes, 280 nm.

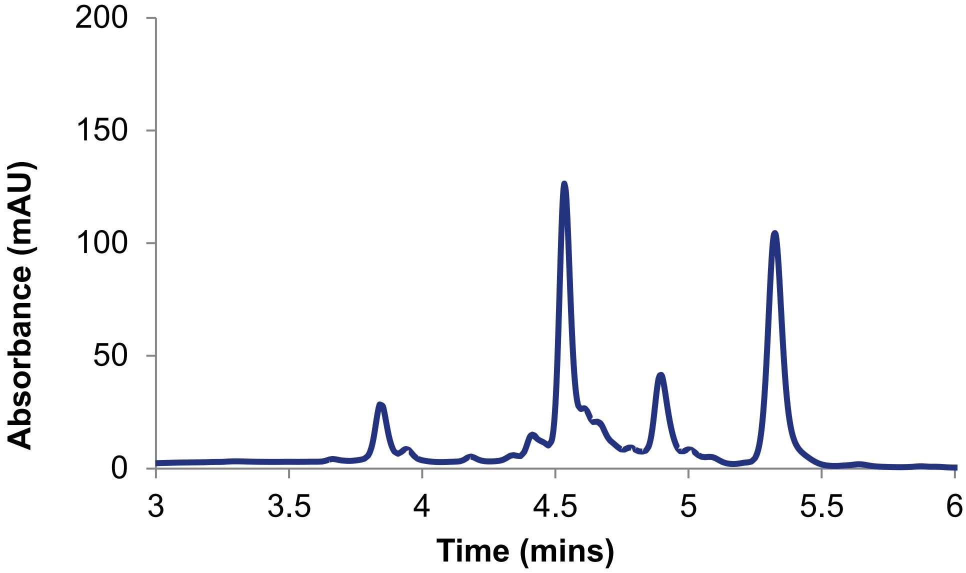

Figure S107. Analytical HPLC trace of the crude conjugation using TEMPO after 1 hour. Analytical gradient 10-100% B over 5 minutes, 280 nm.

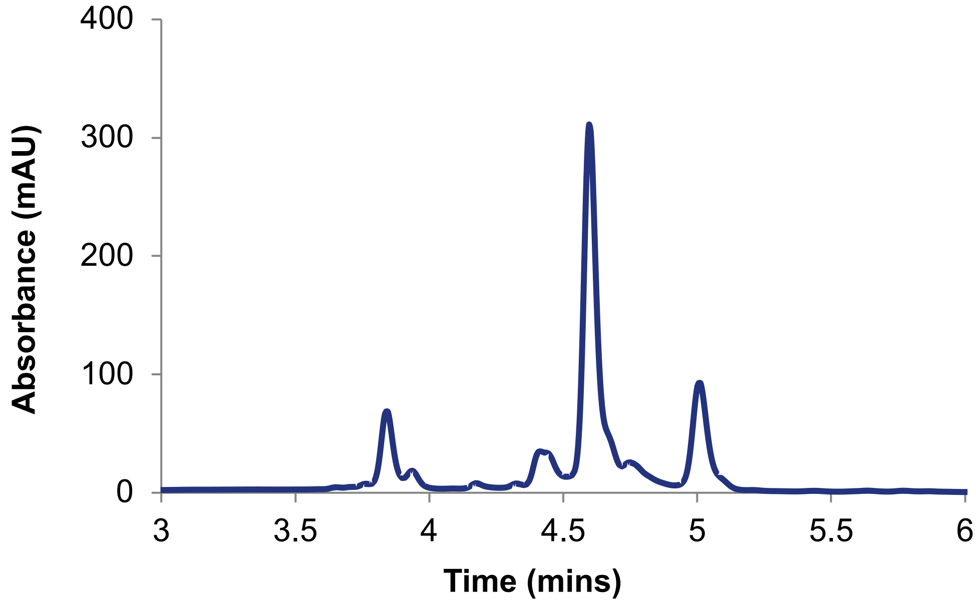

Figure S108. Analytical HPLC trace of the crude reductive cleavage after 16 hours. Analytical gradient 10-100% B over 5 minutes, 280 nm.

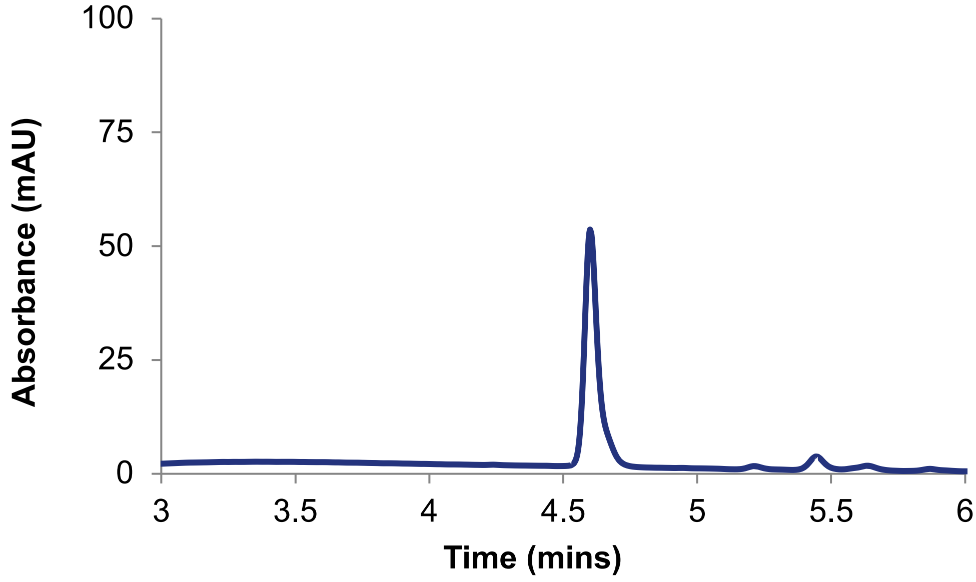

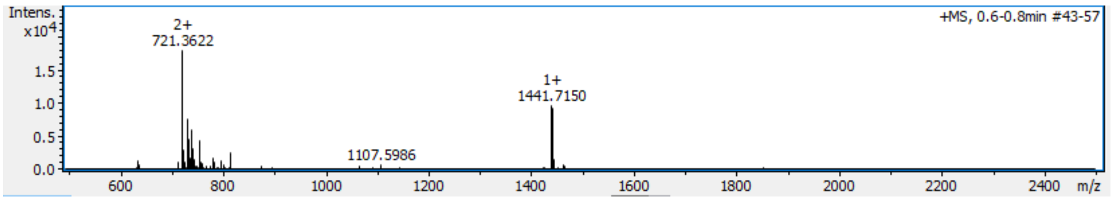

Figure S109. Analytical HPLC trace of pure reductively cleaved product **41** after semi-preparative HPLC purification. Analytical gradient 10-100% B over 5 minutes, 280 nm. Calculated Mass $[M+H]^{+}$: 1441.8189, [M+2H]2+: 721.3634. Observed Mass $[M+H]^{+}$: 1441.7150, [M+2H]2+: 721.3622. 1441.7189

One-pot ligation-oxidation deselenization (Sec to Ser) using Oxone-TCEP method

Synthesized following general ligation protocol using H-UWIMKY-NH_2 (**21**) (1 mg, 1.12 μmol) and Ac-YEPLA-SePh (**23**) (1.04 mg, 1.35 μmol). The ligation solution was diluted to 0.3 mM with ligation buffer. Next, equal volumes of TCEP stock (15 mM stock, 50 eq.) and Oxone stock (15 mM, 50 eq.) both in distilled water were added simultaneously, giving a final peptide concentration of 0.1 mM. This solution was agitated at room temperature for 16 hours before being purified by semi-preparative RP-HPLC (10-100% B over 30 minutes) and lyophilised to yield the desired peptide (1.1 mg, 0.76 μmol, 68% yield).

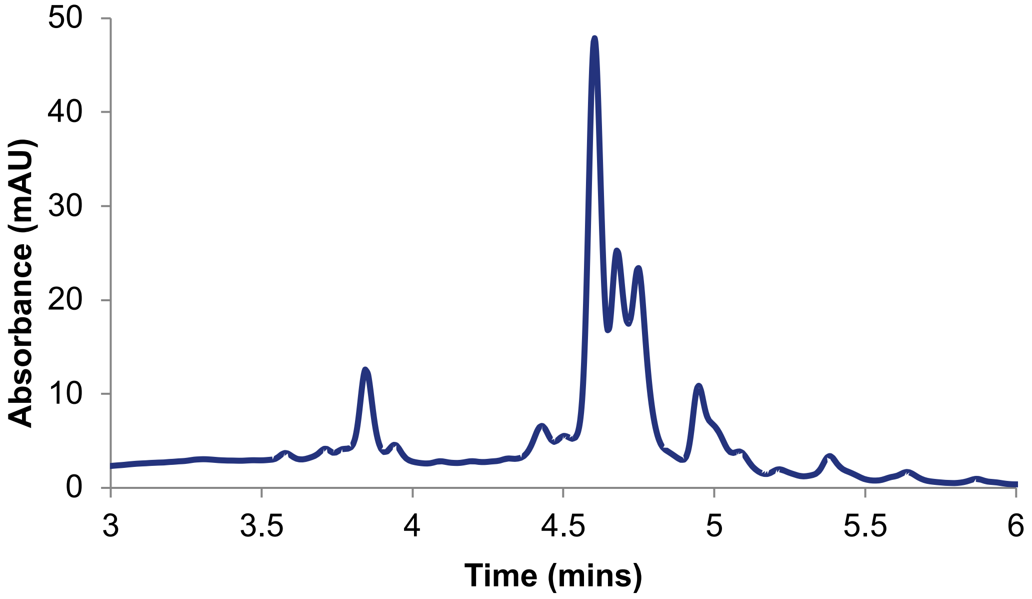

Figure S110. Analytical HPLC trace of crude oxidative ligation solution after 16 hours. Analytical gradient 10-100% B over 5 minutes, 280 nm.

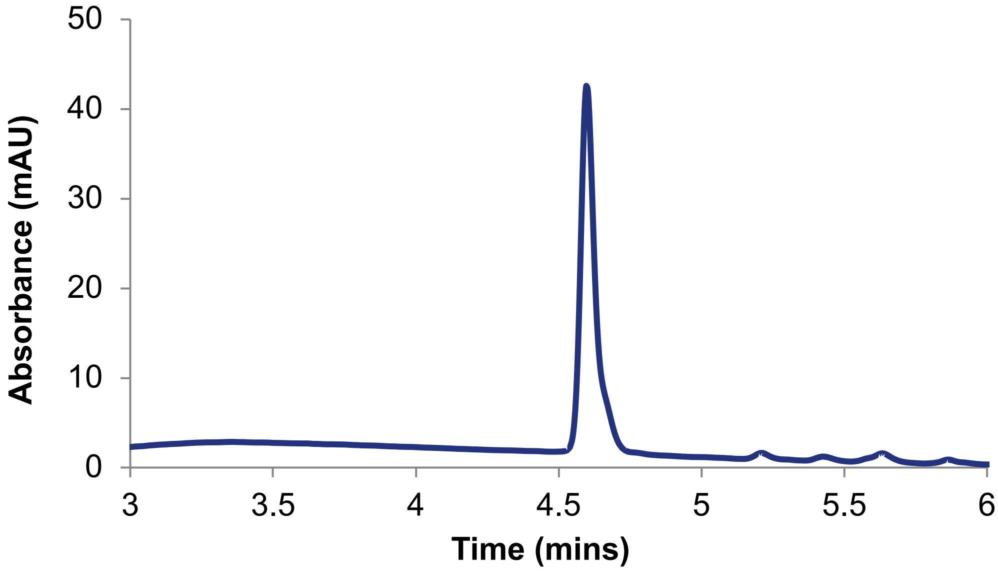

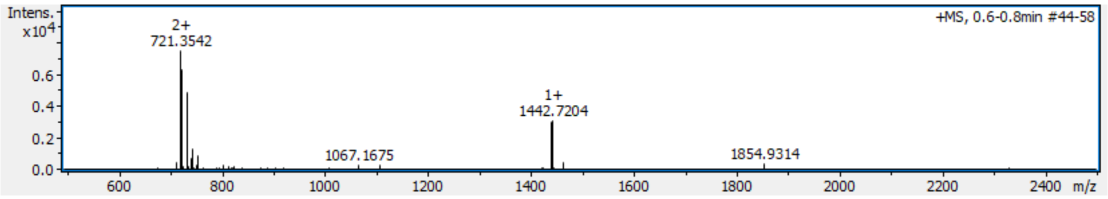

Figure S111. Analytical HPLC trace of pure ligated/oxidatively cleaved product **41**. Analytical gradient 10-100% B over 5 minutes, 280 nm. Calculated Mass $[M+H]^+$: 1441.8189, [M+2H]2+: 721.3634. Observed Mass $[M+H]^+$: 1441.7150, [M+2H]2+: 721.3622.

Synthesis and modification of ZEGFR:1907 via one-pot ligation-conjugation

Two step method - ligation followed by conjugation

Step 1- Ligation

ZEGFR-1 Selenoester (**42**) –

H-VDNKFNKEMWAAWEEIRNLPNLNGWQMT-SePh

ZEGFR-2 Selenopeptide (**43**) –

H-UFIASLVDDPSQSANLLAEAKKLNDAQAPK-OH

ZEGFR:1907(**62**)

H-VDNKFNKEMWAAWEEIRNLPNLNGWQMTUFIASLVDDPSQSANLLAEA KKLNDAQAPK

Synthesized using a two-step protocol, using the general additive-free ligation protocol followed by general protocol A. Firstly, ligation was performed using ZEGFR-1 selenoester (**42**) (3.9 mg, 1.09 µmol) and ZEGFR-2 selenopeptide (**43**) (2.92 mg, 0.910 µmol), followed by purification using semi-preparative RP-HPLC (25-60%B over 30 minutes) and lyophilisation to produce the desired ligated peptide (1.90 mg, 0.287 µmol, 32% yield).

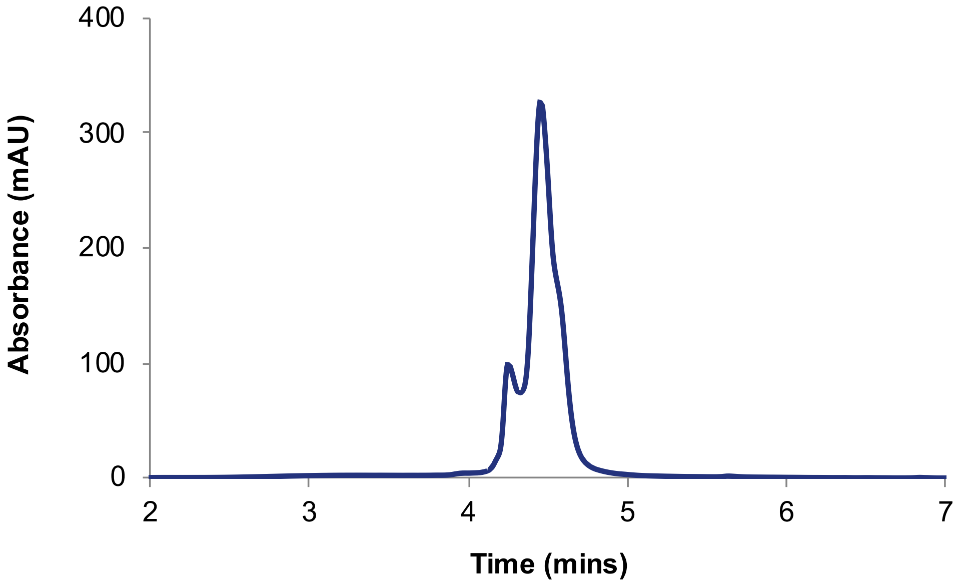

Figure S112. Analytical HPLC trace of crude ligated ZEGFR peptide (**62**). Analytical gradient 10-100% B over 5 min, 280 nm.

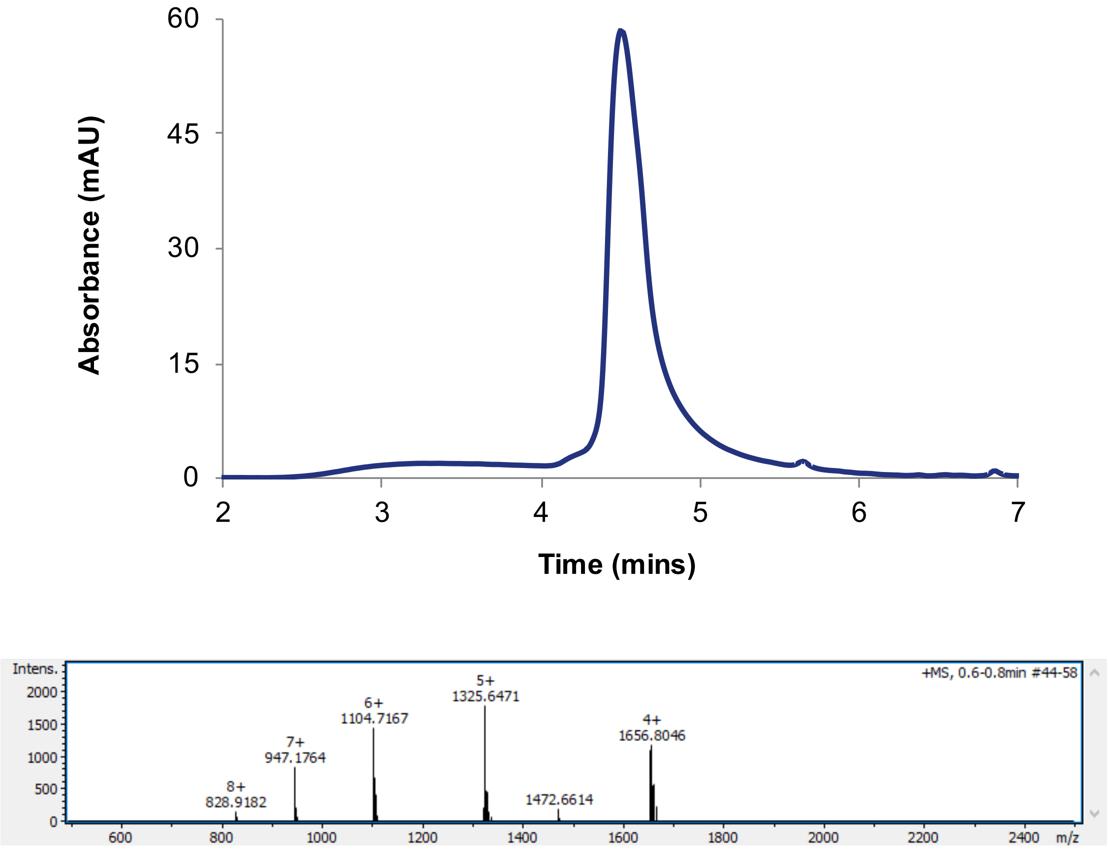

Figure S113. Analytical HPLC trace of pure ligated ZEGFR:1907 peptide (**62**). Analytical gradient 10-100% B over 5 min, 280 nm. Calculated Mass $[M+4H]^{4+}$: 1656.0528, $[M+5H]^{5+}$: 1325.0438, $[M+6H]^{6+}$: 1104.3711, $[M+7H]^{7+}$: 946.7478, $[M+8H]^{8+}$: 828.5303. Observed Mass $[M+4H]^{4+}$: 1656.8046, $[M+5H]^{5+}$: 1325.6471, $[M+6H]^{6+}$: 1104.7167, $[M+7H]^{7+}$: 947.1764, $[M+8H]^{8+}$: 828.9182.

Step 2 - Conjugation (44)

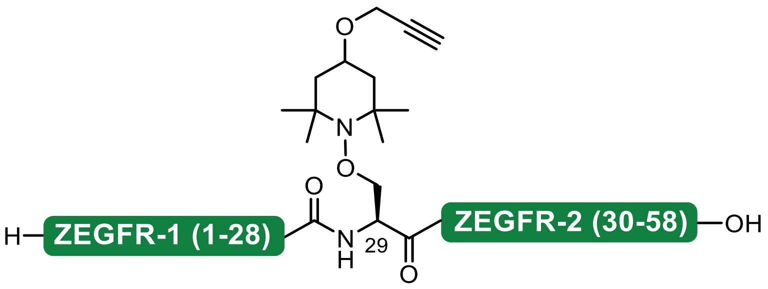

Synthesized following general protocol A using ZEGFR:1907 (**62**, 1.9 mg, 0.287 µmol), followed by purification using semi-preparative RP-HPLC (25-60%B over 30 minutes) and lyophilisation to produce the desired conjugated peptide (1.7 mg, 0.252 µmol, 88% yield).

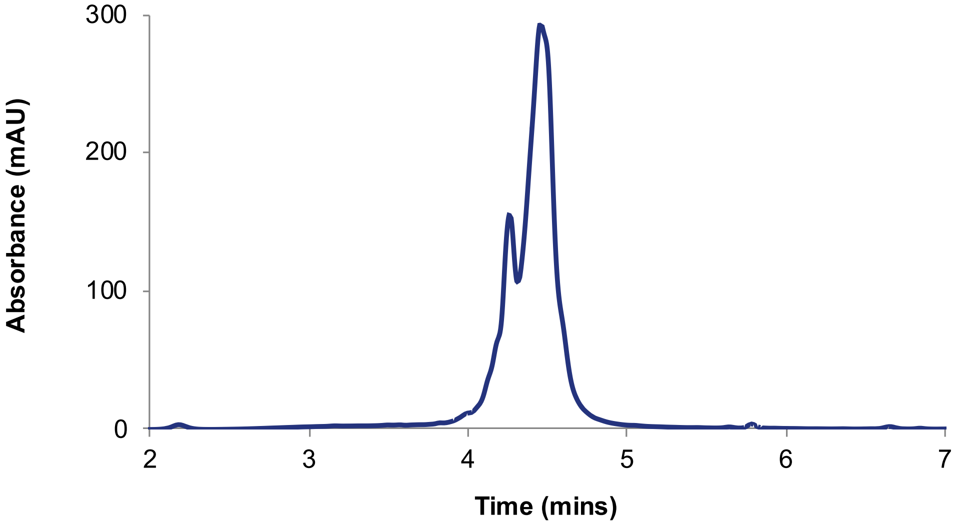

Figure S114. Analytical HPLC trace of crude conjugated ZEGFR:1907 peptide (**44**). Analytical gradient 10-100% B over 5 min, 280 nm.

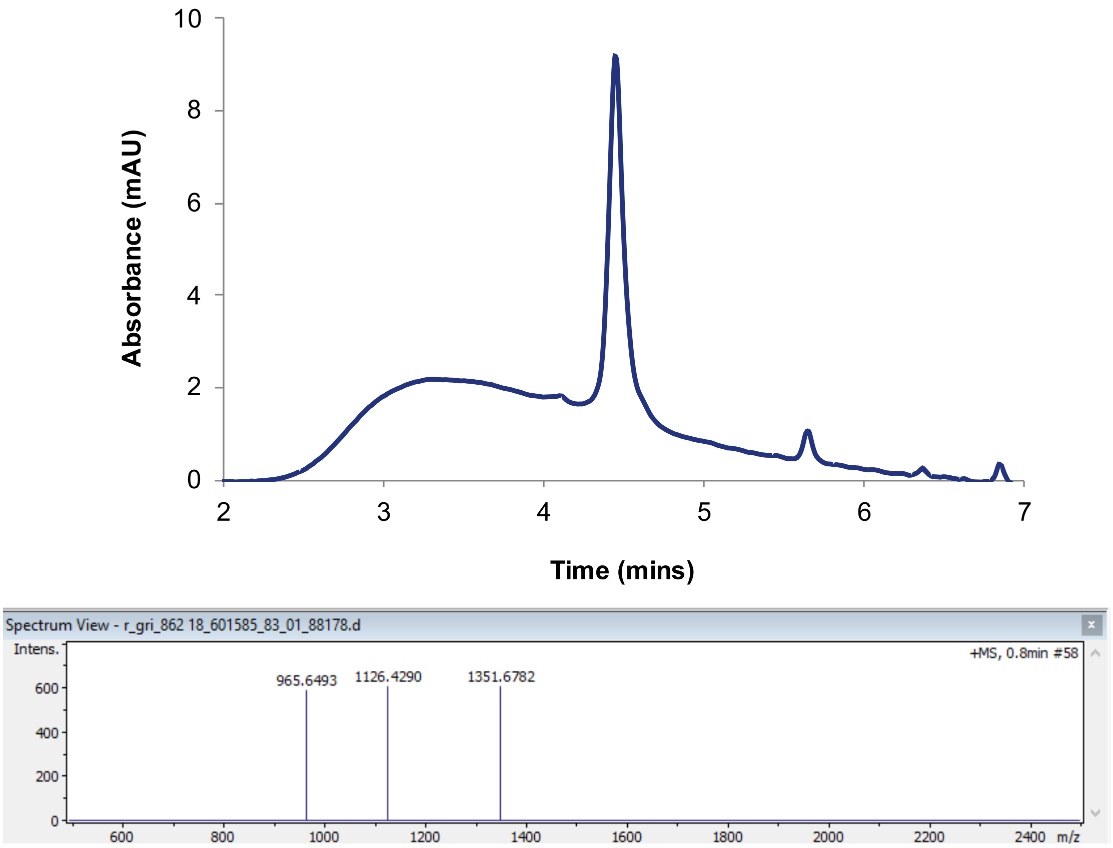

Figure S115. Analytical HPLC trace of pure conjugated ZEGFR:1907 peptide (**44**). Analytical gradient 10-100% B over 5 min, 280 nm. Calculated Mass $[M+5H]^{5+}$: 1350.6898, $[M+6H]^{6+}$: 1125.7428, $[M+7H]^{7+}$: 965.0664. Observed Mass $[M+5H]^{5+}$: 1351.6782, $[M+6H]^{6+}$: 1126.4290, $[M+7H]^{7+}$: 965.6493.

One-pot method to form ZEGFR:1907 conjugated product (44)

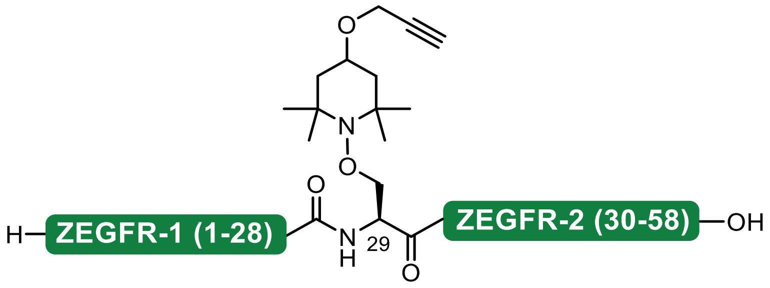

Synthesized following general protocol A with a one-pot general ligation method using ZEGFR-1 (**42**) (4.37 mg, 1.22 µmol) and ZEGFR-2 (**43**) (3.27 mg, 1.02 µmol) followed by purification using semi-preparative RP-HPLC (25-60%B over 30 minutes) and lyophilisation to produce the desired conjugated peptide (2.9 mg, 0.429 µmol, 42% yield).

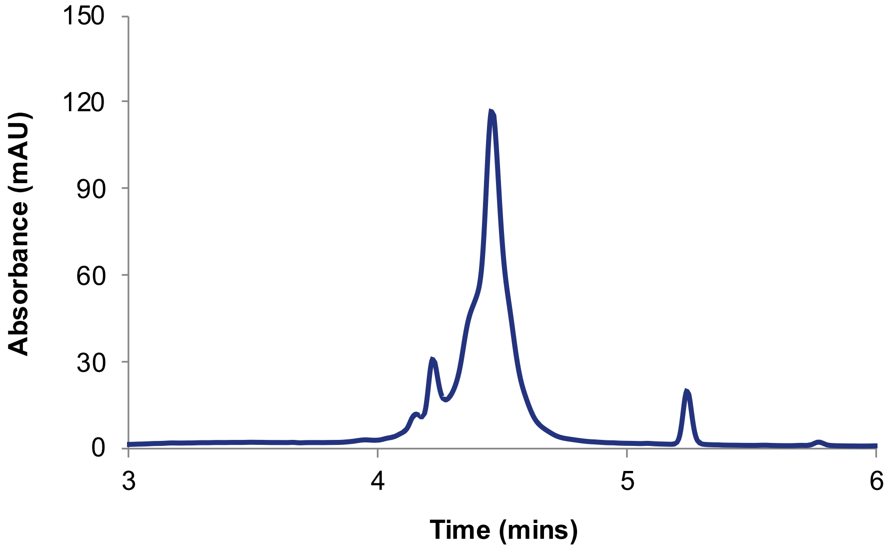

Figure S116. Analytical HPLC trace of crude one-pot ligation and conjugation of ZEGFR:1907 peptide (**44**) using propargyl-TEMPO. Analytical gradient 10-100% B over 5 min, 280 nm.

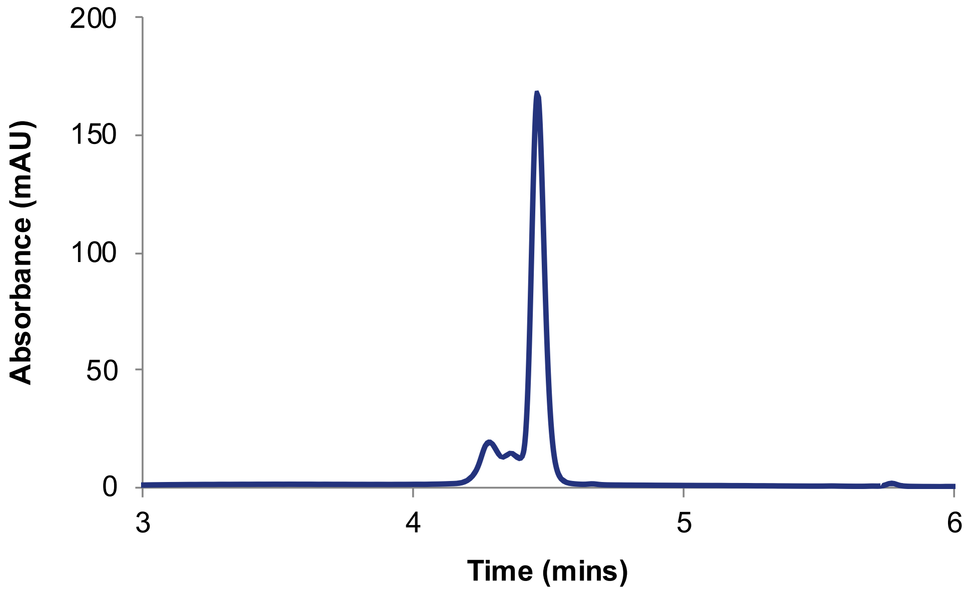

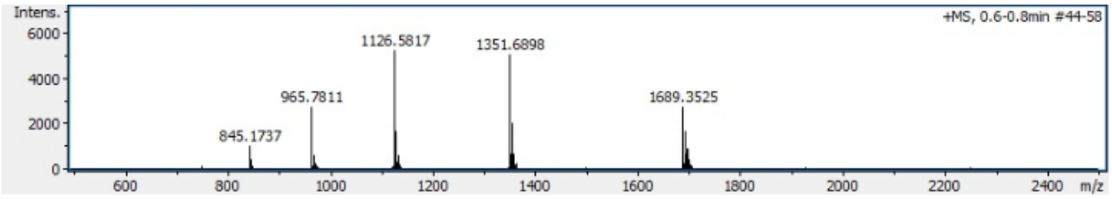

Figure S117. Analytical HPLC trace of pure one-pot ligated and conjugated ZEGFR:1907 peptide using propargyl-TEMPO (**44**). Analytical gradient 10-100% B over 5 min, 280 nm. Calculated Mass $[M+4H]^{4+}$: 1689.3458, $[M+5H]^{5+}$: 1351.6782, $[M+6H]^{6+}$: 1126.5665, $[M+7H]^{7+}$: 965.7724, $[M+8H]^{8+}$: 845.1768. Observed Mass $[M+4H]^{4+}$: 1689.3525, $[M+5H]^{5+}$: 1351.6898, $[M+6H]^{6+}$: 1126.5817, $[M+7H]^{7+}$: 965.7811, $[M+8H]^{8+}$: 845.1737.

Site-selective modification of ubiquitin K48C

Ubiquitin K48C mutant protein **45** was recombinantly expressed and purified as described in Garner *et al.* 2011 Biochemistry[3] with SDS PAGE analysis confirming ~99% purity (not shown).

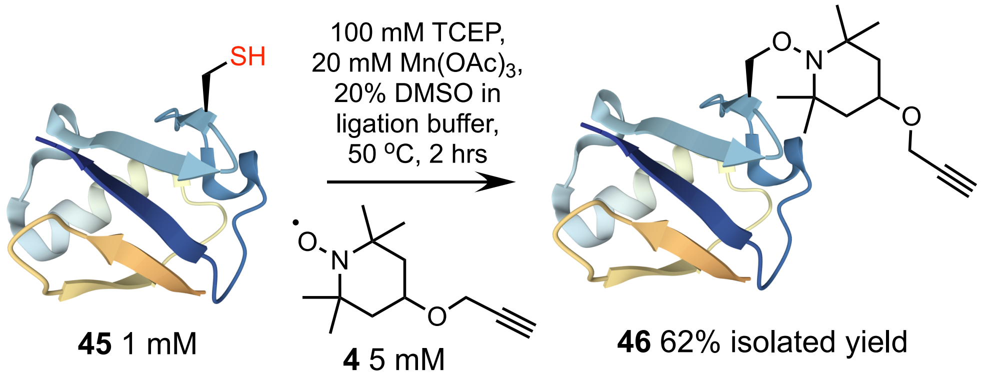

Ub K48C (45) Sequence – H-MQIFVKTLTGKTITLEVESSDTIDNVKSKIQDKEGIPPDQQRLIFAGCQLEDGRTLSDYNIQKESTLHLVLRLRGG-OH

Synthesized following general protocol E using K48C Ubiquitin mutant (**45**, 6.6 mg, 0.774 µmol) followed by purification using semi-preparative RP-HPLC (25-45% B over 30 minutes) and lyophilisation to produce the desired conjugated protein **46** (4.2 mg, 0.482 µmol, 62% yield).

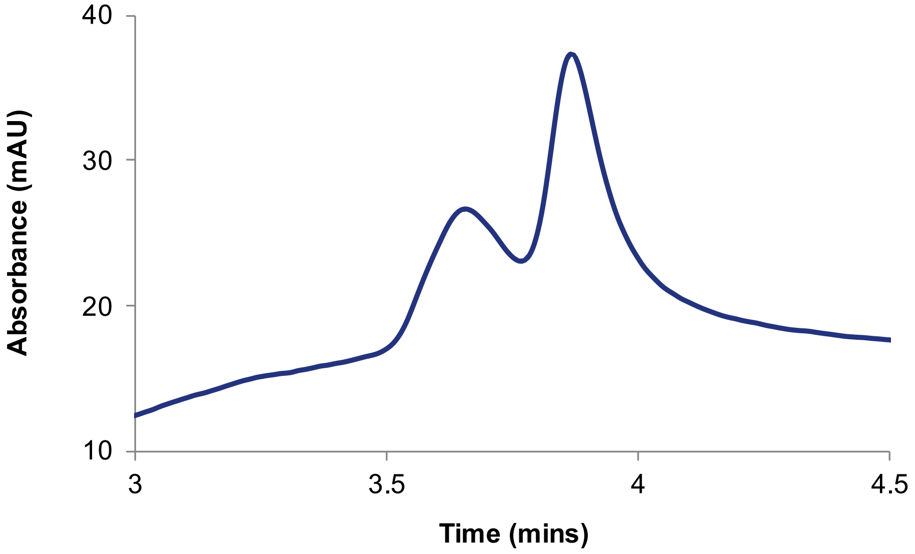

Figure S118. Analytical HPLC trace of 2 hours crude reaction mixture of Ub K48C modified with propargyl-TEMPO (**46**). Analytical gradient 25-60% B over 5 min, 210 nm.

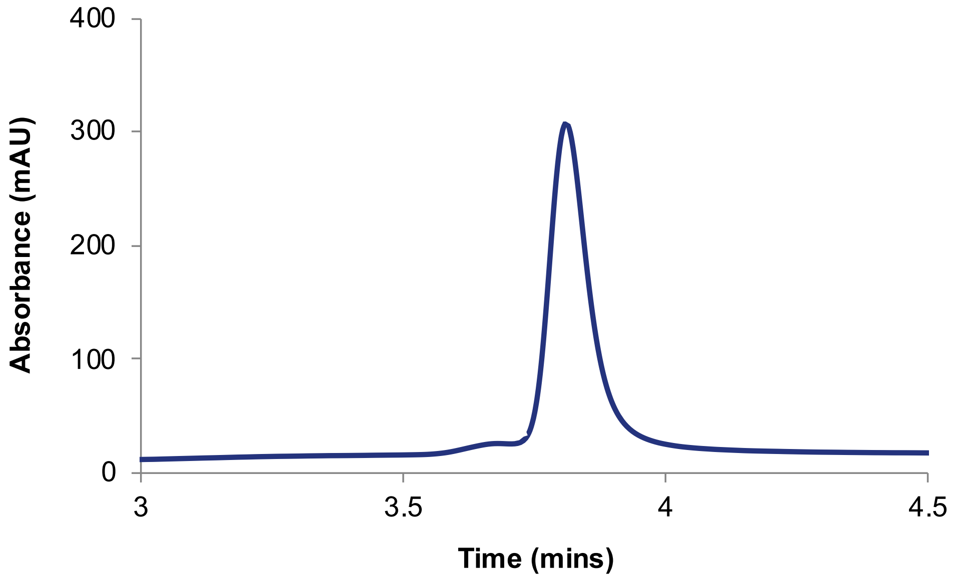

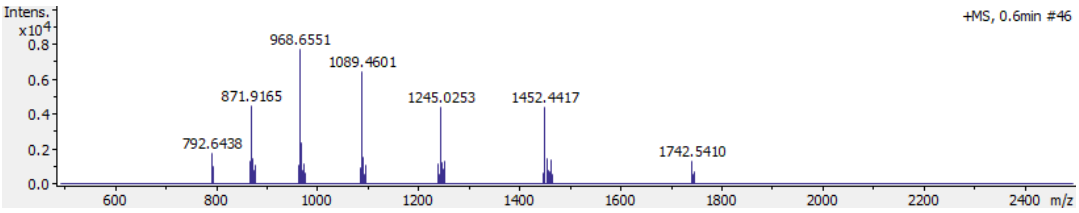

Figure S119. Analytical HPLC trace of pure Ub K48C modified with propargyl-TEMPO (**46**). Analytical gradient 25-60% B over 5 min, 210 nm. Calculated Mass $[M+5H]^{5+}$: 1743.1397, $[M+6H]^{6+}$: 1452.7844, $[M+7H]^{7+}$: 1245.3877, $[M+8H]^{8+}$: 1089.8402, $[M+9H]^{9+}$: 968.8588, $[M+10H]^{10+}$: 872.0737, $[M+11H]^{11+}$: 792.8859. Observed Mass $[M+5H]^{5+}$: 1742.5410, $[M+6H]^{6+}$: 1452.4417, $[M+7H]^{7+}$: 1245.3877, $[M+8H]^{8+}$: 1089.4601, $[M+9H]^{9+}$: 968.6551, $[M+10H]^{10+}$: 871.9165, $[M+11H]^{11+}$: 792.6438.

NMR analysis of folded Ub conjugate 46

Purified Ub-conjugate **46** and the started K48C protein **45** were dissolved in ligation buffer and dialysed into folding buffer (25 mM Na_2PO_4, 100 mM NaCl, pH 6.9) overnight. To each sample was added 10 vol% D_2O and the samples were analysed on an Avance III 800 MHz NMR system.

A TOCSY of both the K48C Ub mutant **45** and the conjugate Ub **46** were recorded and assigned by comparison to the published ubiquitin data (BMRB 68 and 4769). Overall, the spectra show that the fold of ubiquitin is intact. The upshifted methyl groups are all visible, albeit at a slightly different chemical shift due to the perturbation of Phe45 against which they pack. In the NH Hα region, which is the most sensitive to local perturbation, there is movement around the attachment site as would be expected, however the majority of the protein shows little chemical shift deviation. Whilst it was not possible to directly observe the attached moiety, the fact Gly53 show such a great deviation suggests it is there and the alkyne group is close to the glycine.

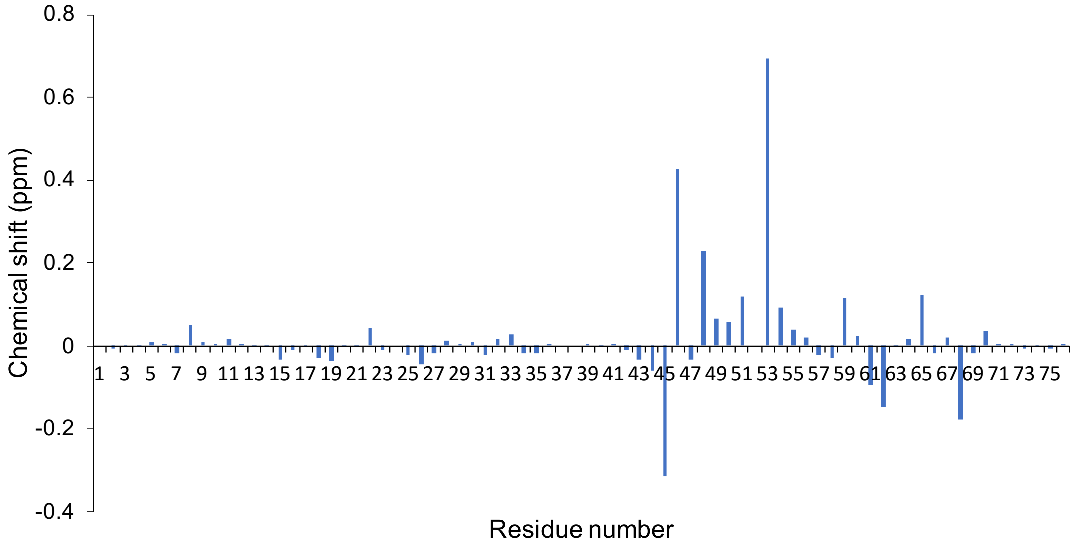

Figure S120. Δ Chemical shift (ppm) plot comparing NH signals for K48C Ub **45** and Ub conjugate **46.**

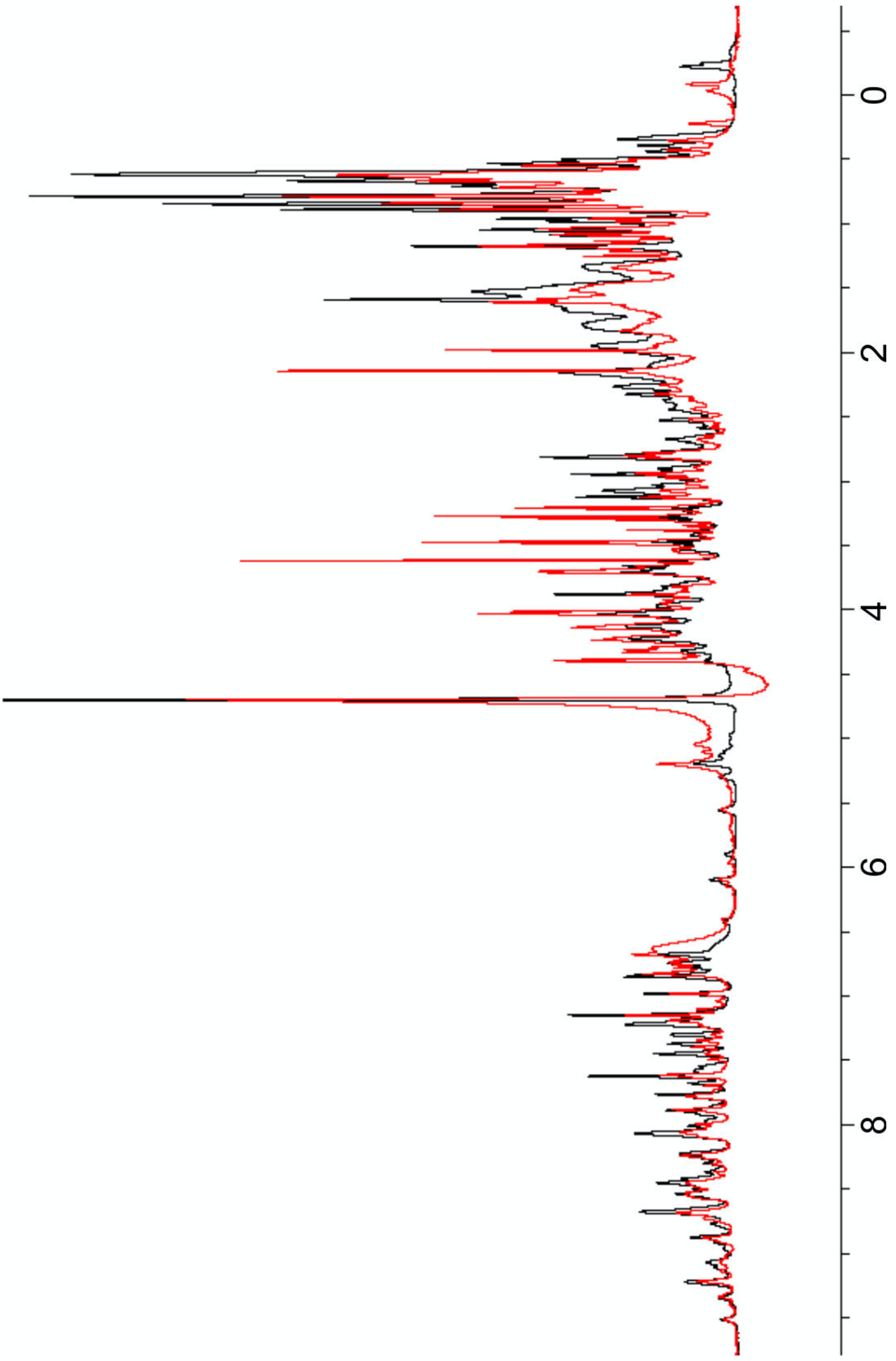

Figure S121. Overlay for ^{1}H NMR spectra of K48C Ub mutant **45** (black) and Ub conjugate **46** (red); NH region extended across 6.5-9.5 ppm indicating a tertiary fold.

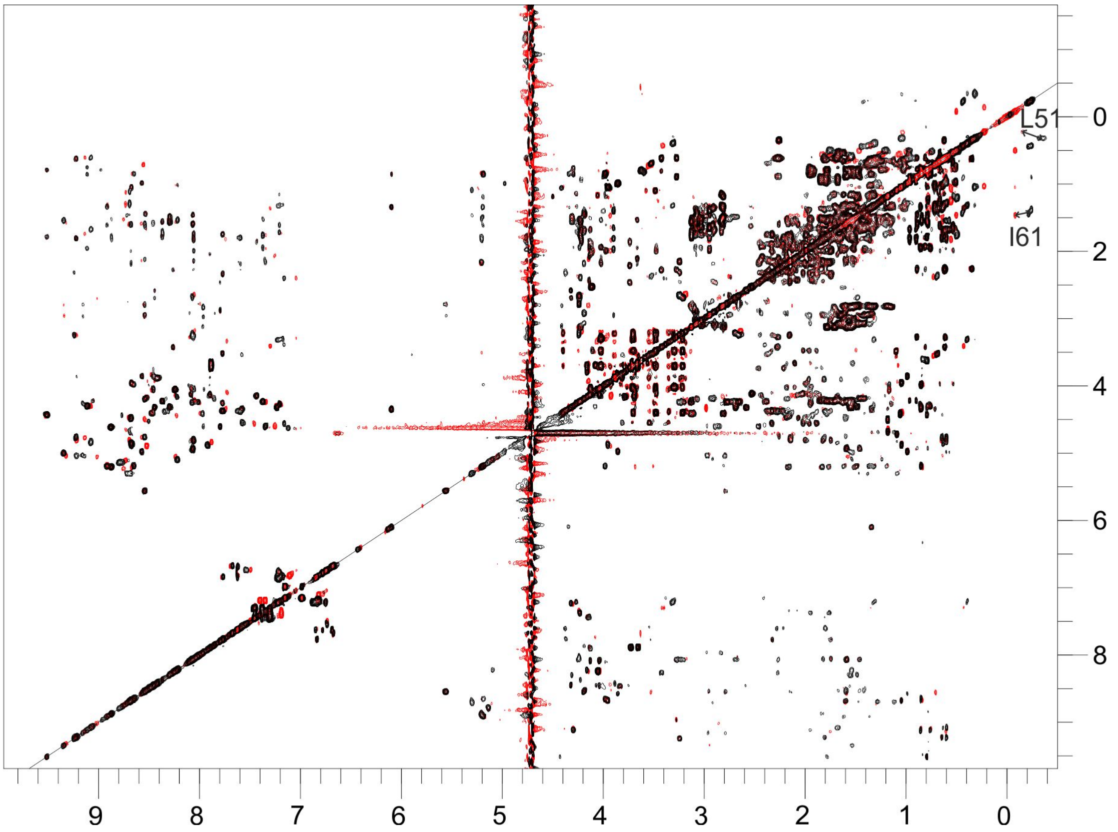

Figure S122. Overlay of TOSCY spectra of K48C Ub mutant **45** (black) and Ub conjugate **46** (red).

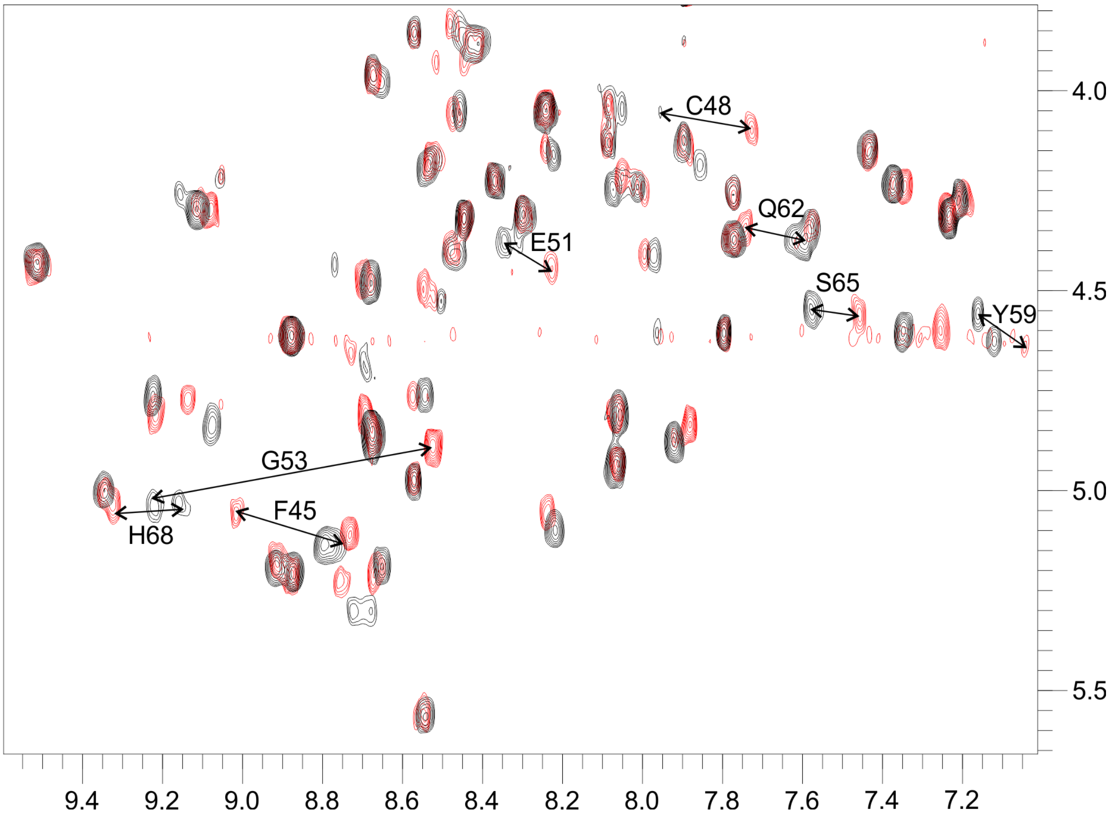

Figure S123. Expansion of NH region of the overlaid TOCSY spectra for K48C Ub mutant **45** (black) and Ub conjugate **46** (red); signal shifts around modified residue 48 highlighted.

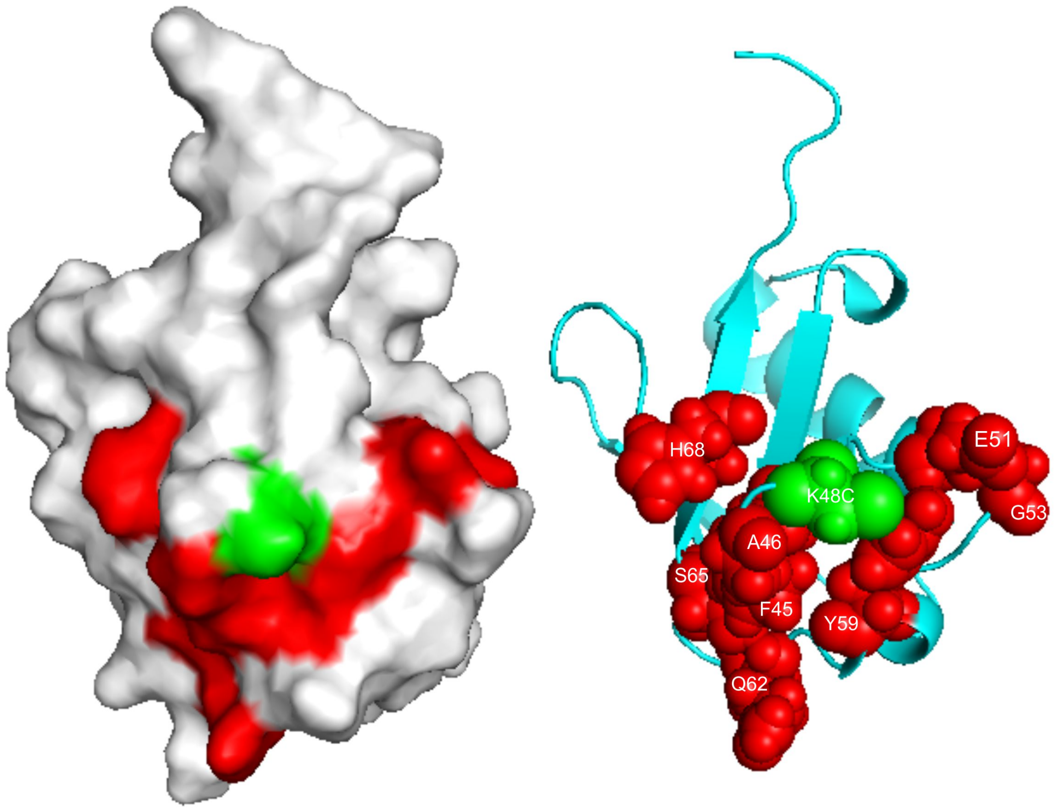

Figure S124. Space filling and ribbon models of Ub; modified residue at position 48 highlighted in green, residues that experience significant deviation in chemical shifts for NH signal highlighted in red.

References

1. M. B. Sims, J. L. Lessard, L. Bai, B. S. Sumerline, *Macromolecules* **2018**, 51, 16, 6380-6386.
2. M. Kunishima, C. Kawachi, J. Monta, K. Terao, F. Iwasaki, S. Tani, *Tetrahedron* **1999***,* 55, 46, 13159-13170.
3. T. P. Garner, J. Strachan, E. C. Shedden, J. E. Long, J. R. Cavey, Barry Shaw, R. Layfield, M. S. Searle, *Biochemistry* **2011**, 50, 42, 9076-9087.
4. Biological Magnetic Resonance Data Bank (BMRDB) http://www.bmrb.wisc.edu/ - entries 68 and 4769.

Appendix

H-UAF-OMe (1a)

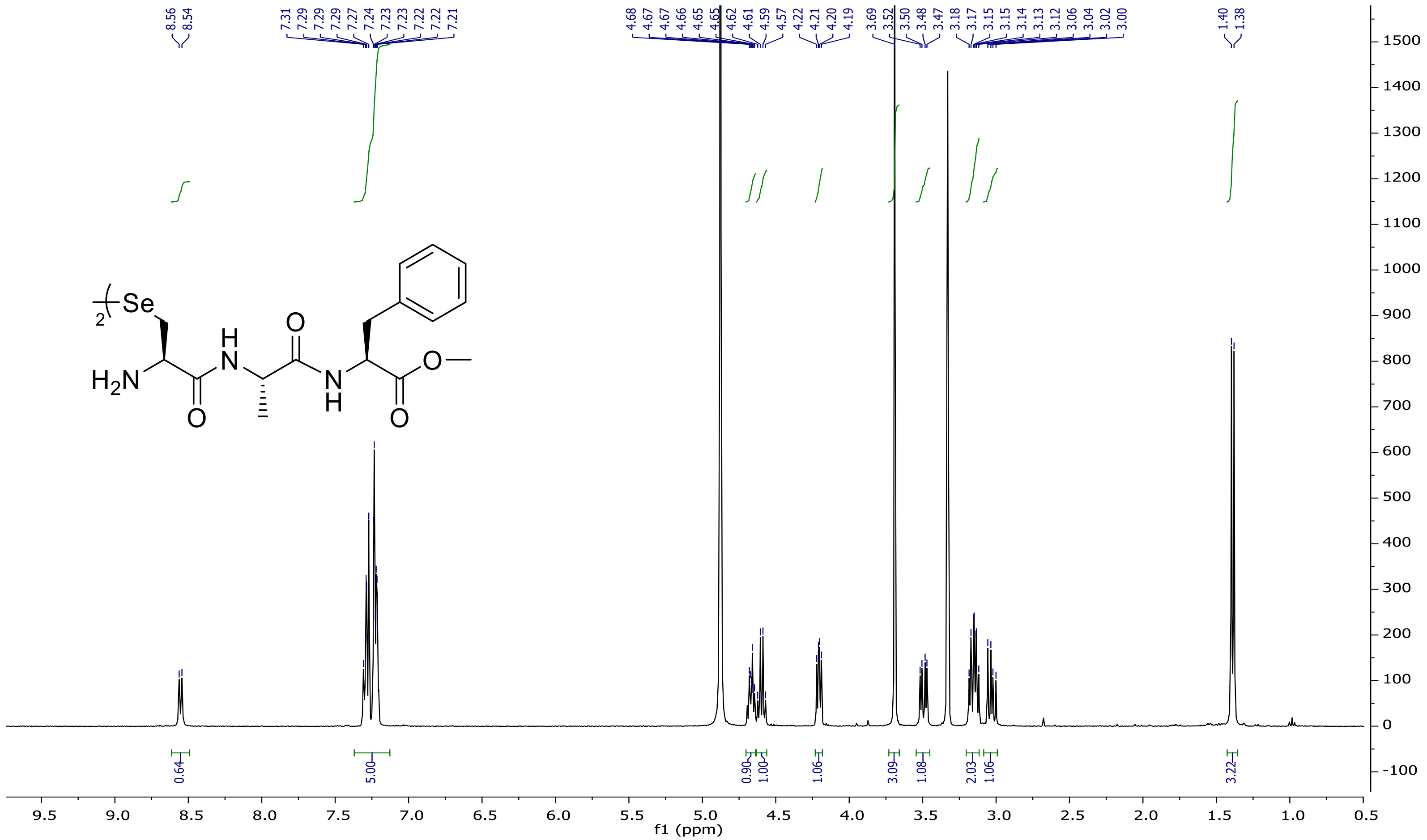

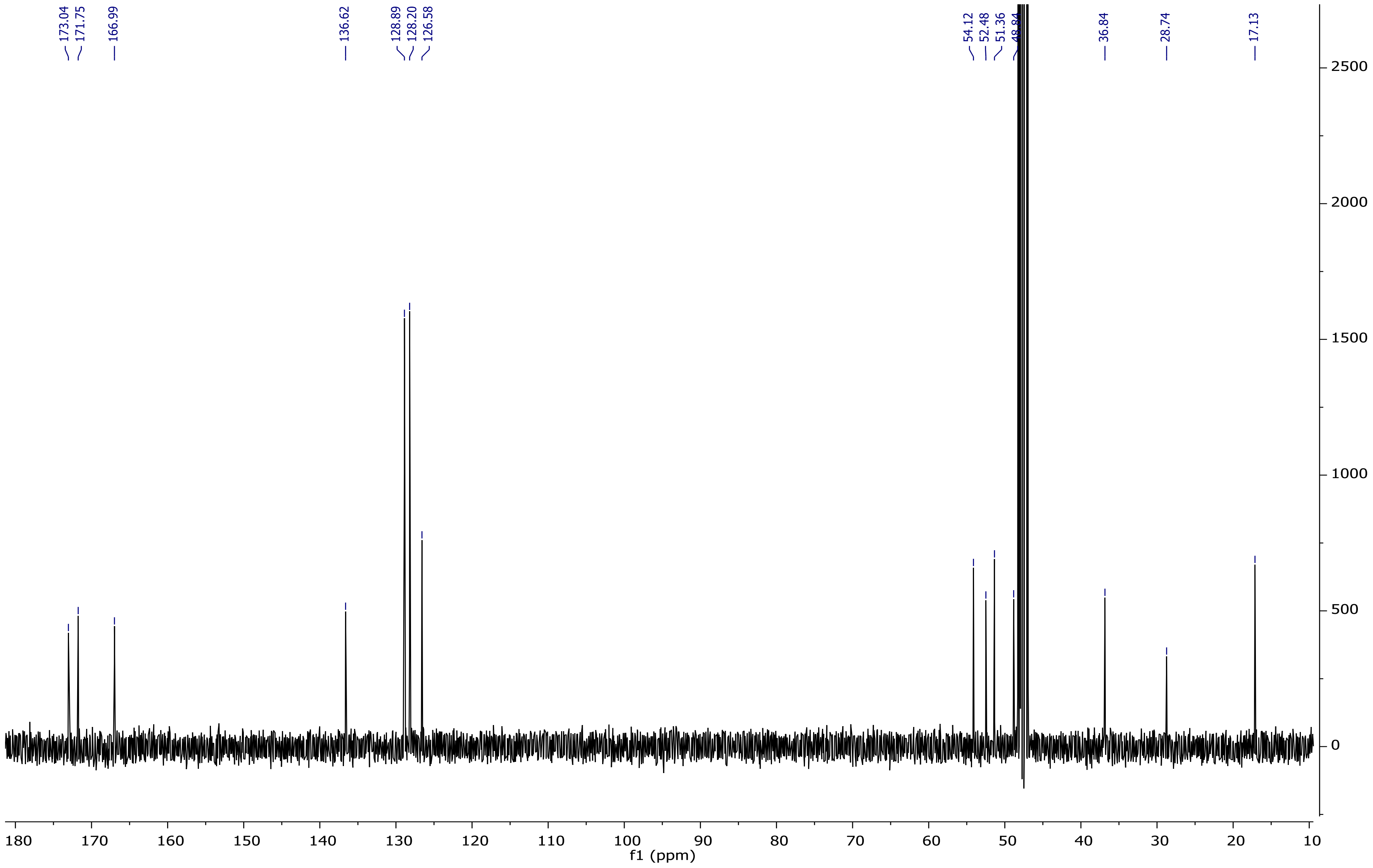
173.04
171.75
166.99
136.62
128.89
128.20
126.58
54.12
52.48
51.36
48.84
36.84
28.74
17.13
2500
2000
1500
1000
500
0
180 170 160 150 140 130 120 110 100 90 80 70 60 50 40 30 20 10
f1 (ppm)

H-D-UAF-OMe (1b)

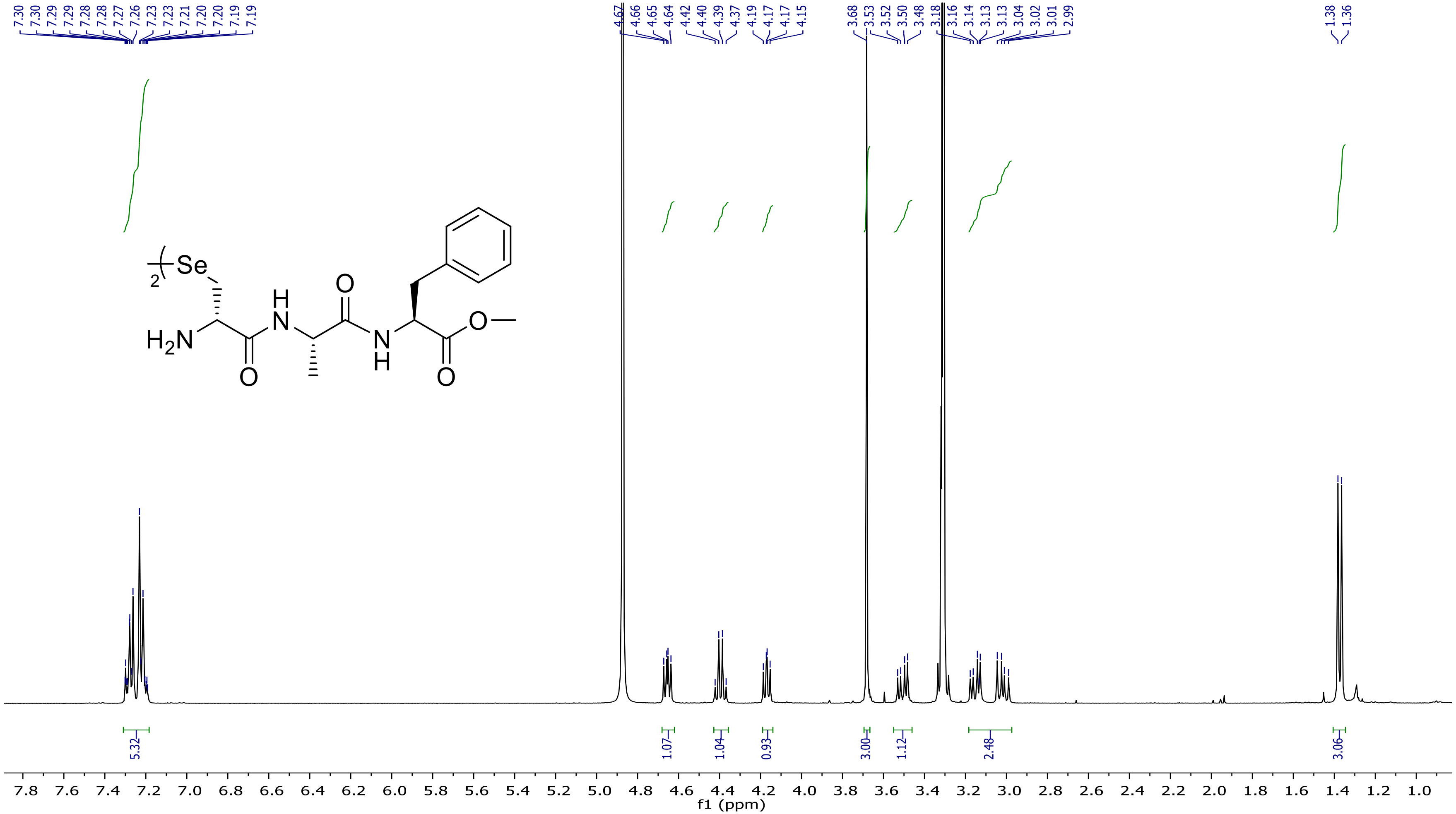
7.30 7.30 7.29 7.29 7.28 7.28 7.27 7.26 7.23 7.23 7.21 7.20 7.20 7.19 7.19
4.67 4.66 4.65 4.64 4.42 4.40 4.39 4.37 4.19 4.17 4.17 4.15
3.68 3.53 3.52 3.50 3.48 3.18 3.16 3.14 3.13 3.13 3.04 3.02 3.01 2.99
1.38 1.36
Se
2
H2N
H
N
O
O
N
H
O
O
5.32
1.07
1.04
0.93
3.00
1.12
2.48
3.06
7.8 7.6 7.4 7.2 7.0 6.8 6.6 6.4 6.2 6.0 5.8 5.6 5.4 5.2 5.0 4.8 4.6 4.4 4.2 4.0 3.8 3.6 3.4 3.2 3.0 2.8 2.6 2.4 2.2 2.0 1.8 1.6 1.4 1.2 1.0
f1 (ppm)

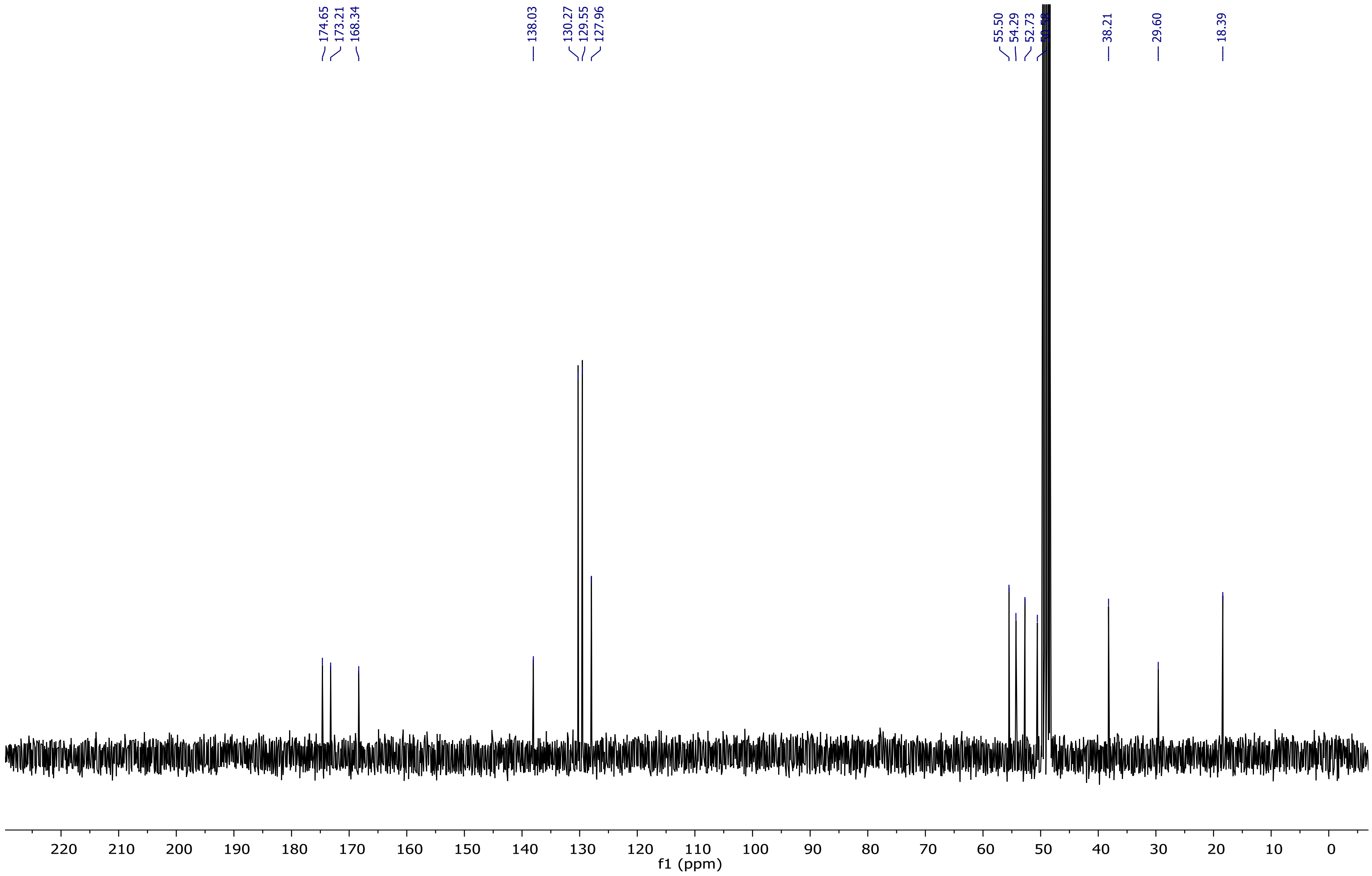
174.65
173.21
168.34
138.03
130.27
129.55
127.96
55.50
54.29
52.73
50.58
38.21
29.60
18.39
220 210 200 190 180 170 160 150 140 130 120 110 100 90 80 70 60 50 40 30 20 10 0
f1 (ppm)

H-UAF-OMe with TEMPO (2a)

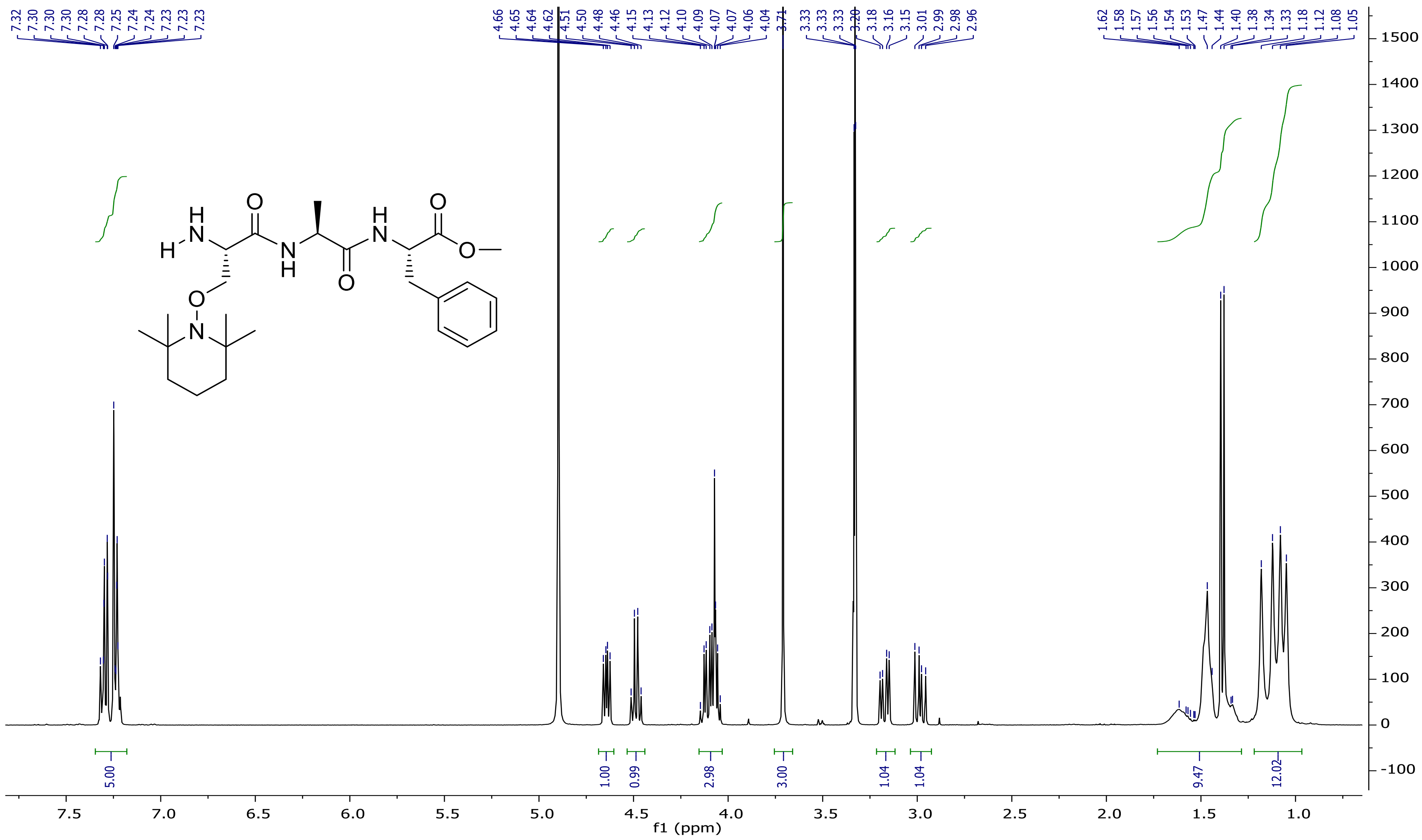

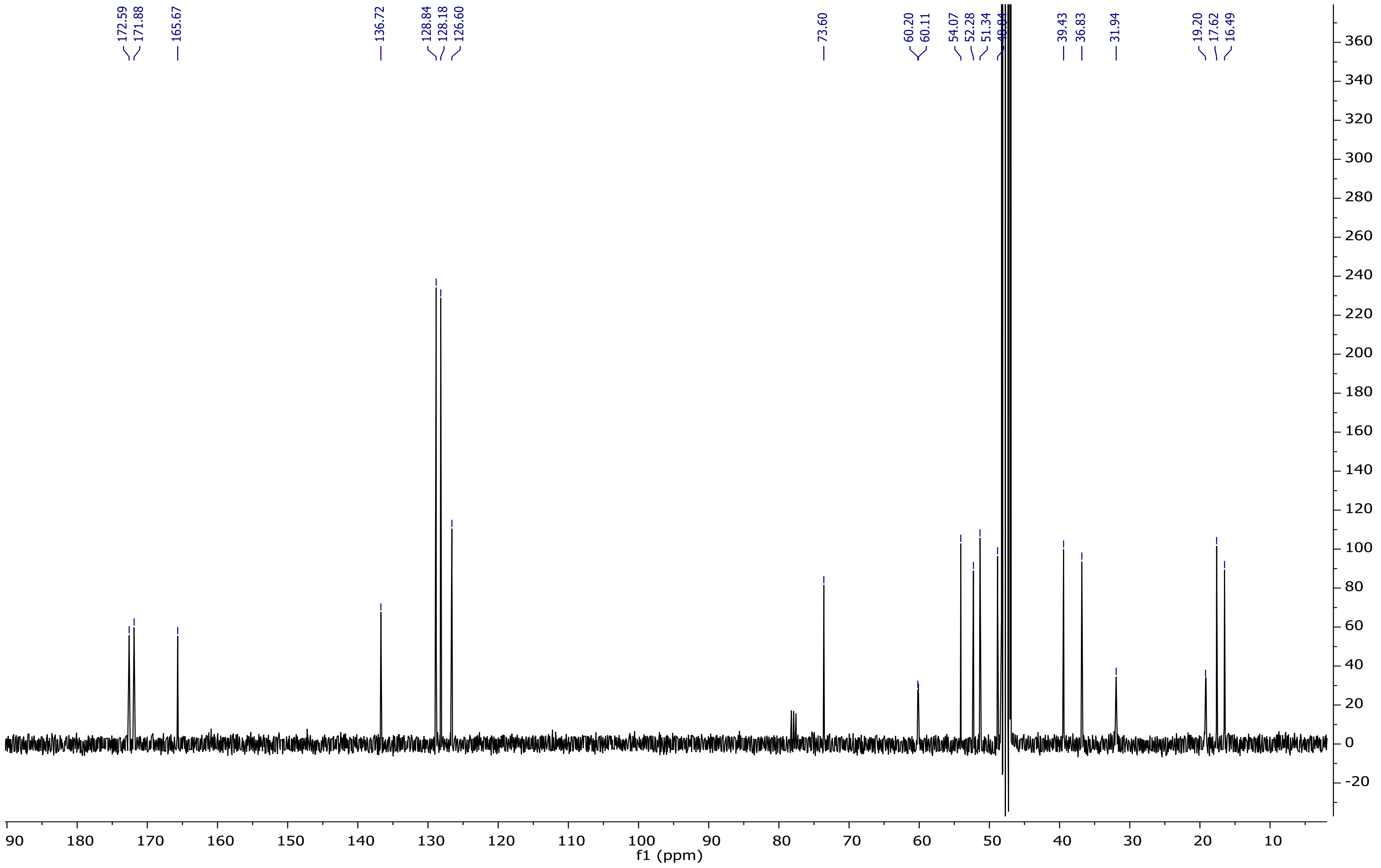
172.59
171.88
165.67
136.72
128.84
128.18
126.60
73.60
60.20
60.11
54.07
52.28
51.34
39.43
36.83
31.94
19.20
17.62
16.49
f1 (ppm)

H-D-UAF-OMe with TEMPO (2b)

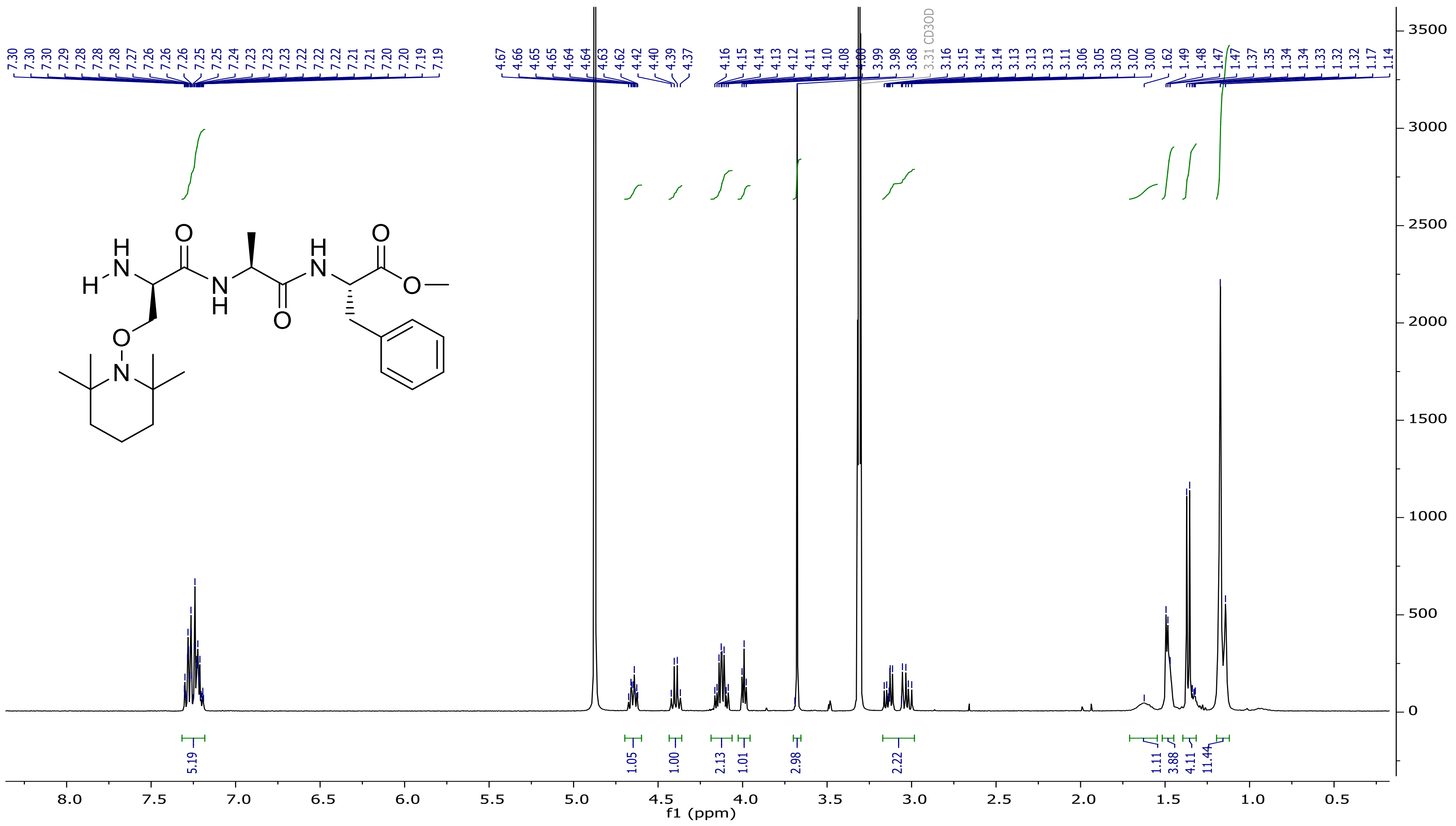

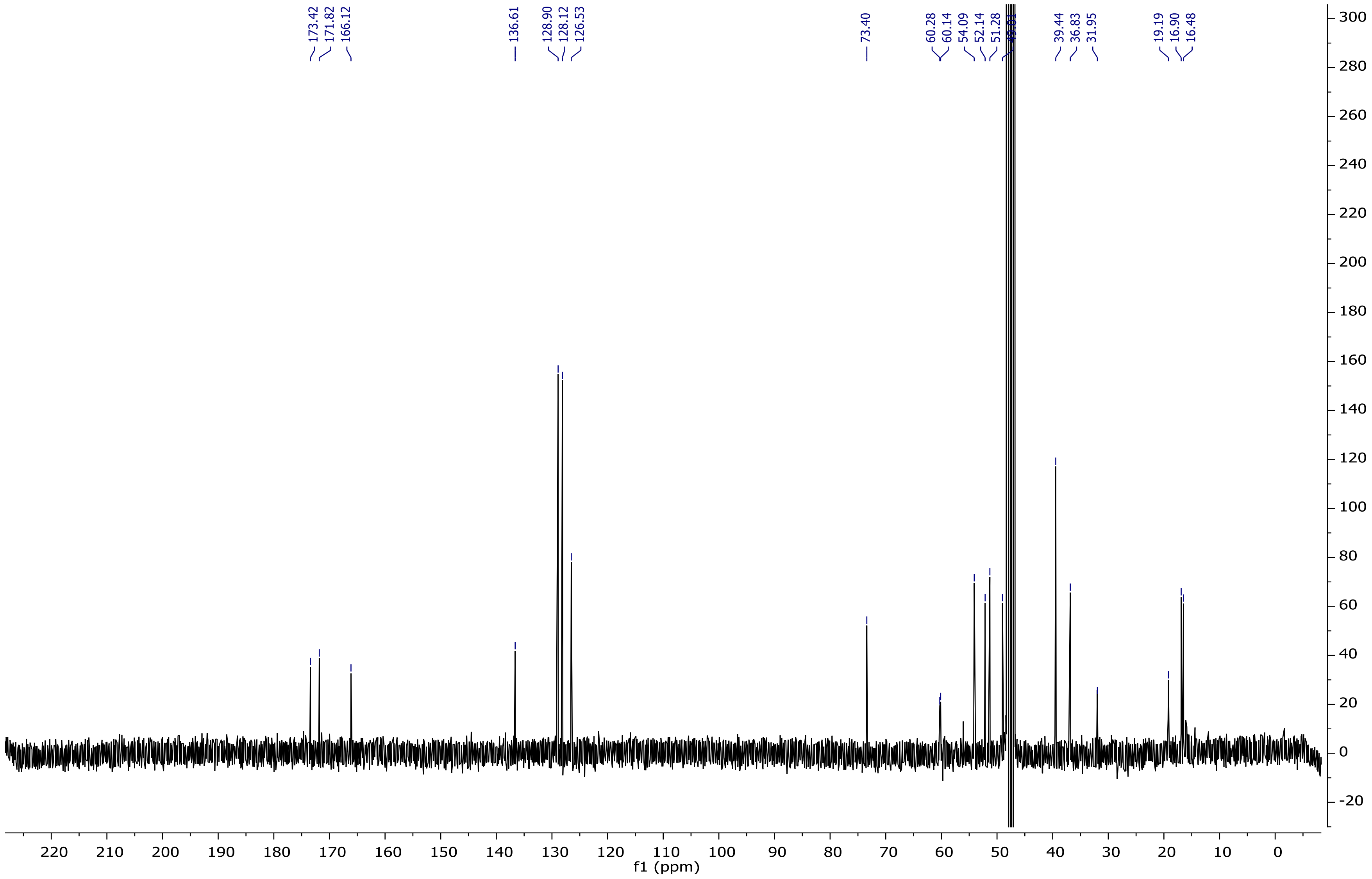
173.42
171.82
166.12
136.61
128.90
128.12
126.53
73.40
60.28
60.14
54.09
52.14
51.28
39.44
36.83
31.95
19.19
16.90
16.48
f1 (ppm)

Tetra EG-TEMPO (3)

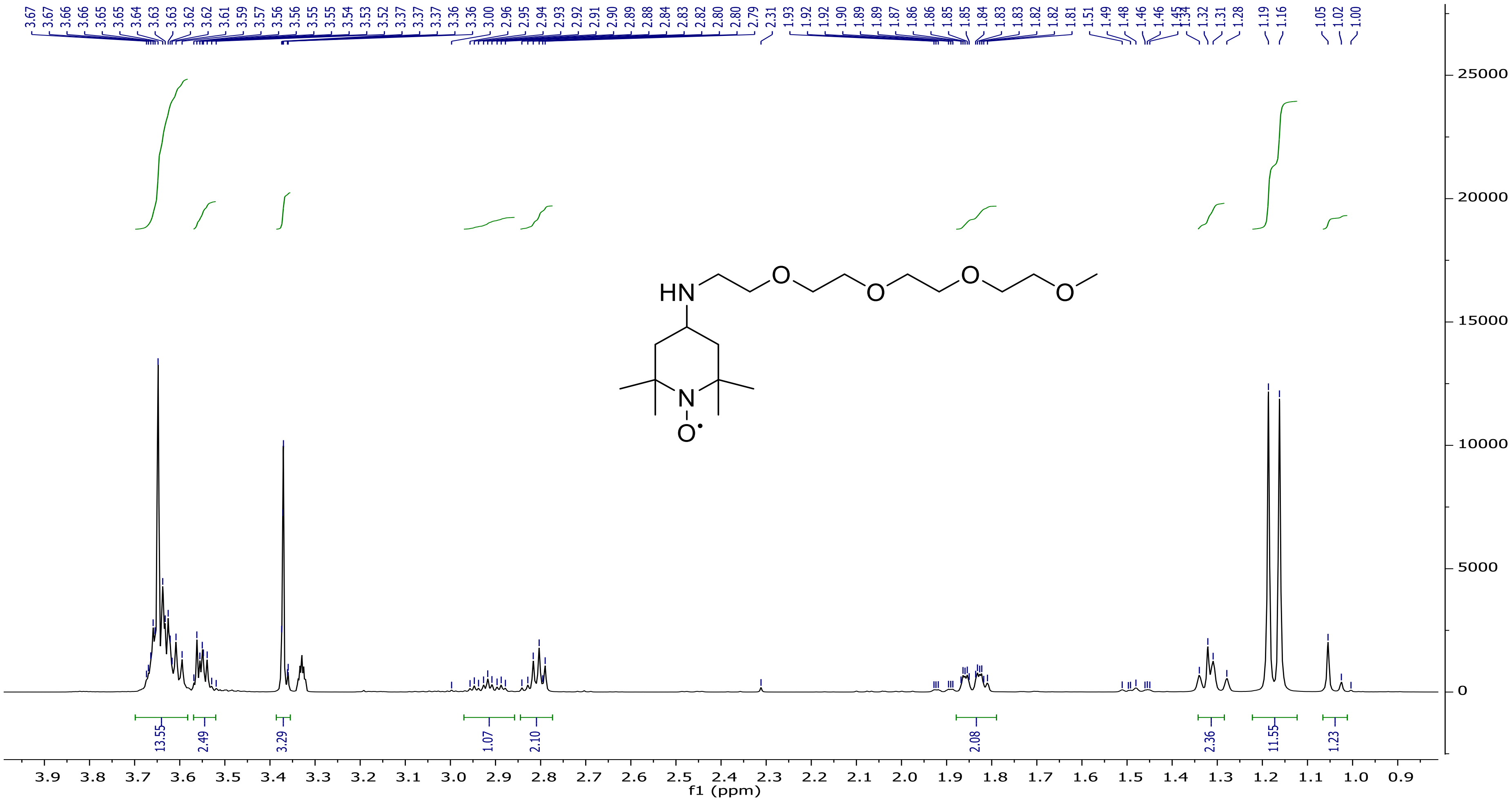

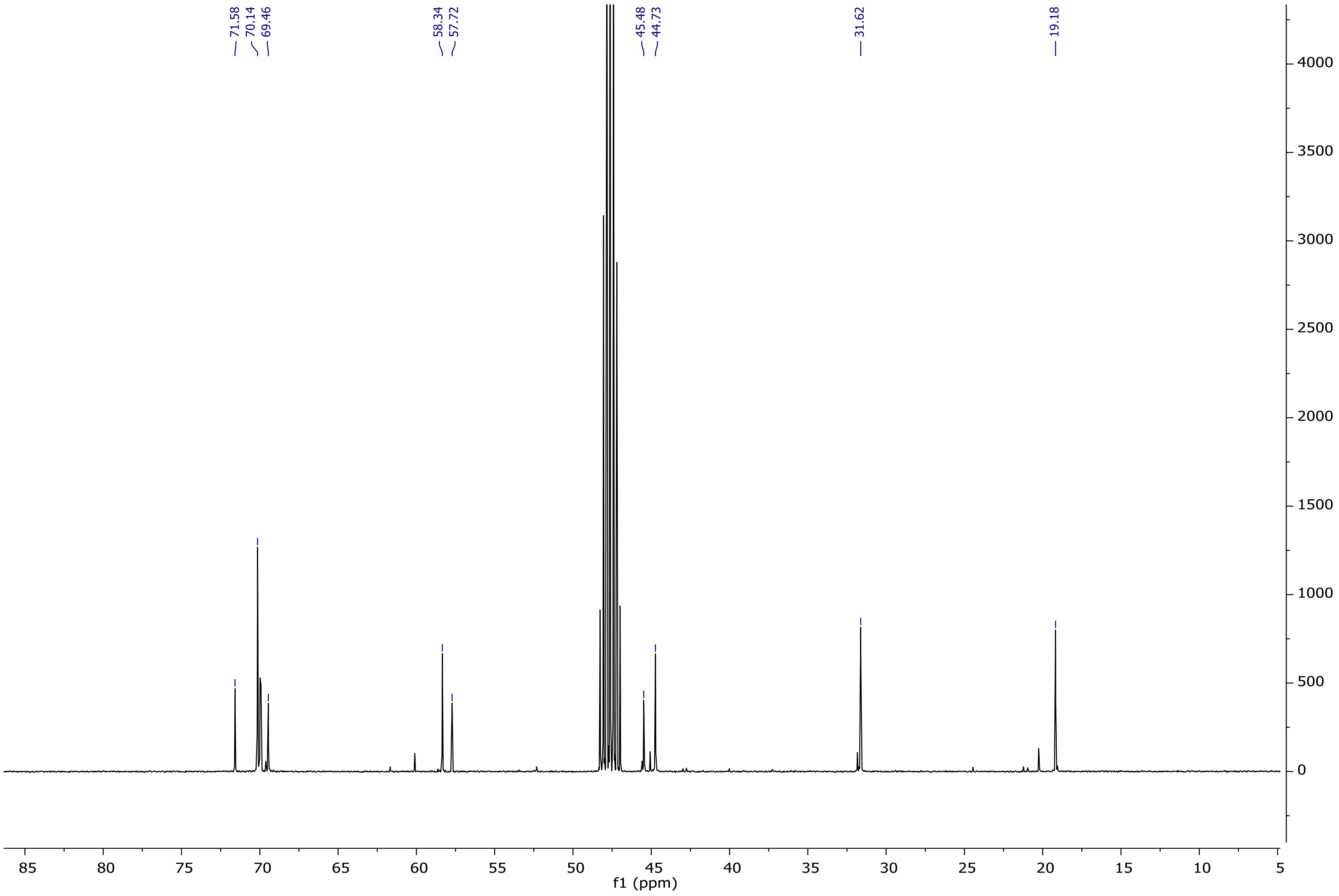

71.58
70.14
69.46
58.34
57.72
45.48
44.73
31.62
19.18
4000
3500
3000
2500
2000
1500
1000
500
0
85
80
75
70
65
60
55
50
45
40
35
30
25
20
15
10
5
f1 (ppm)

Propargyl TEMPO (4)

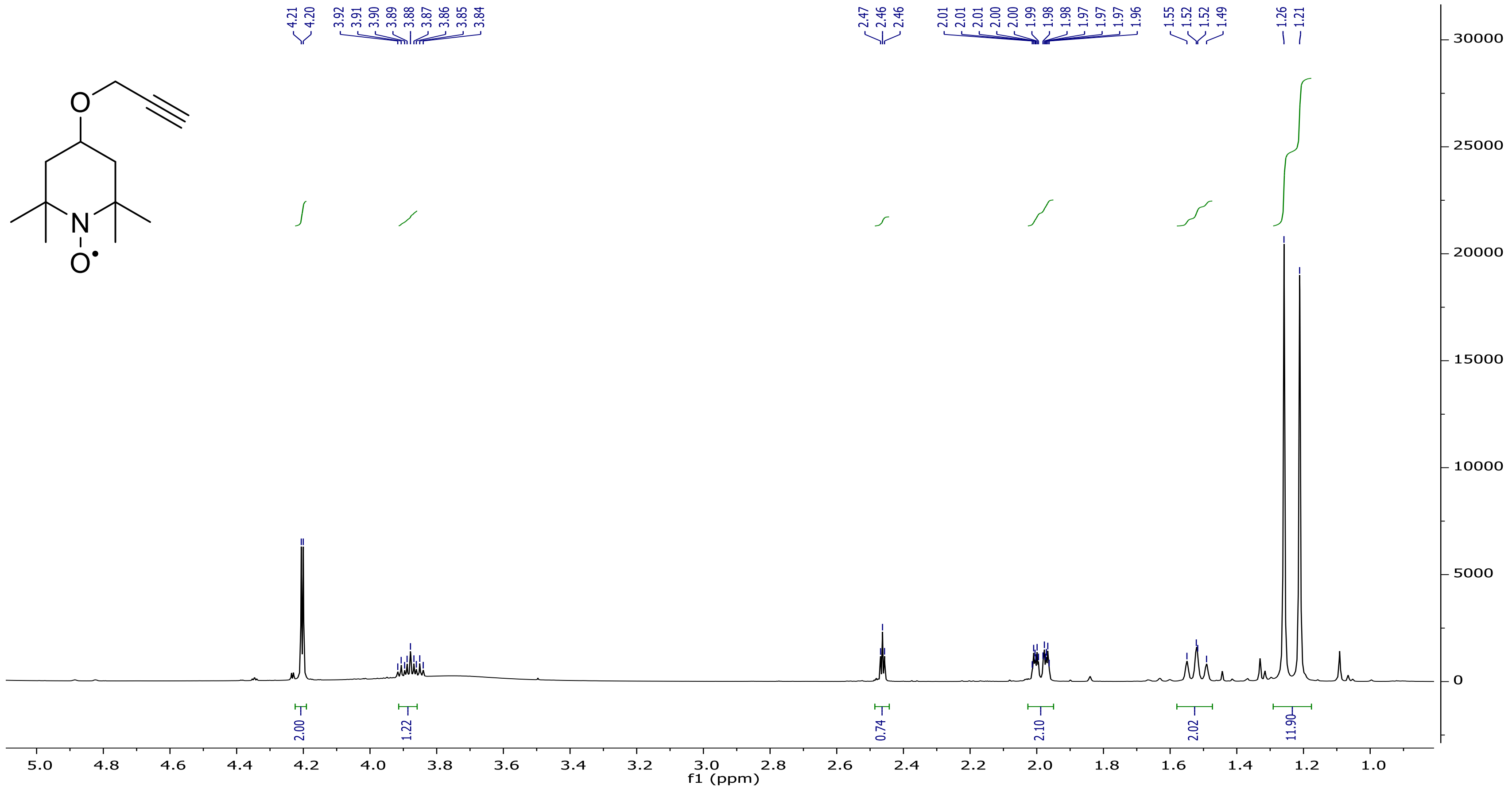

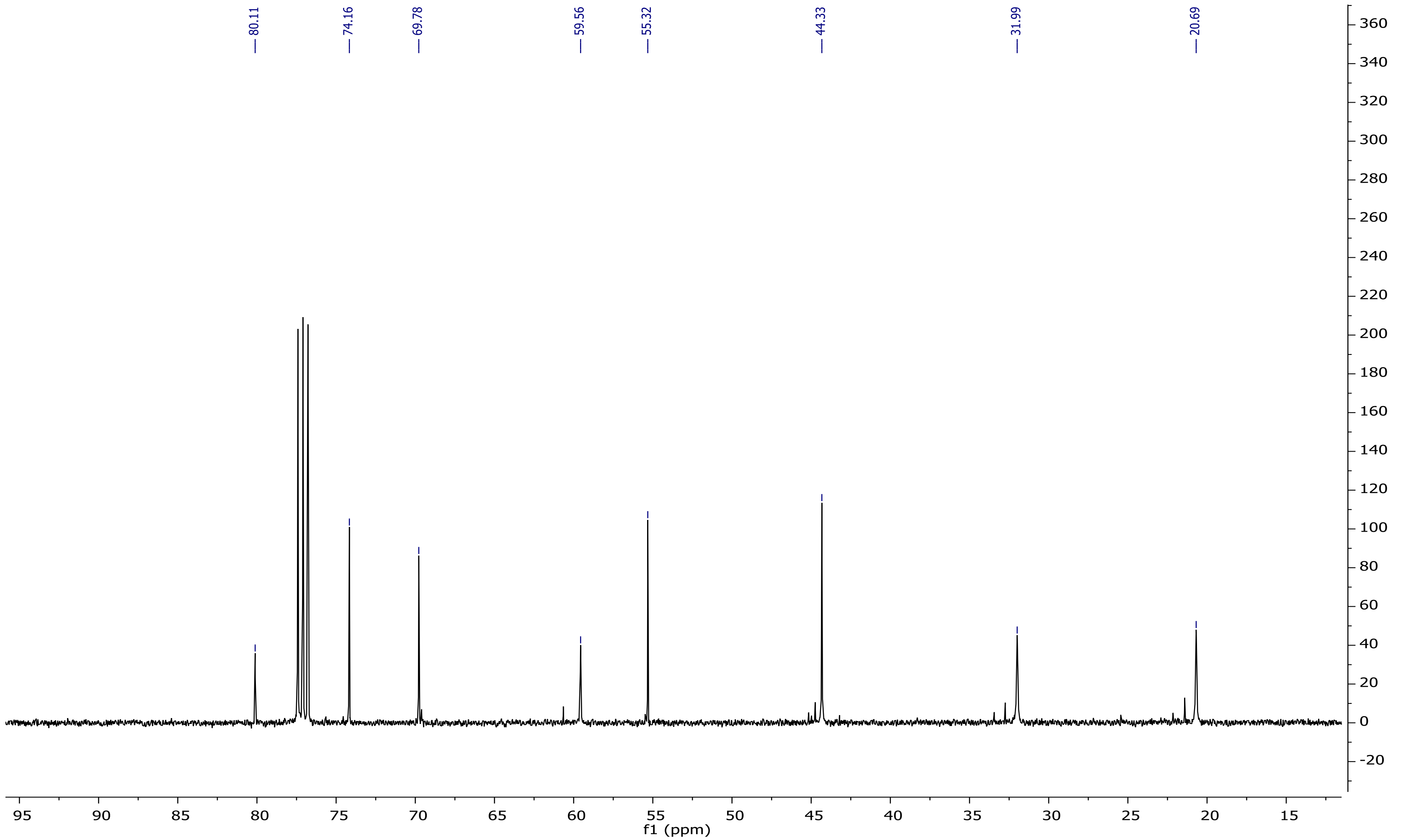
80.11
74.16
69.78
59.56
55.32
44.33
31.99
20.69
360
340
320
300
280
260
240
220
200
180
160
140
120
100
80
60
40
20
0
-20
95
90
85
80
75
70
65
60
55
50
45
40
35
30
25
20
15
f1 (ppm)

Fluorescein isothiocyanate-TEMPO (5)

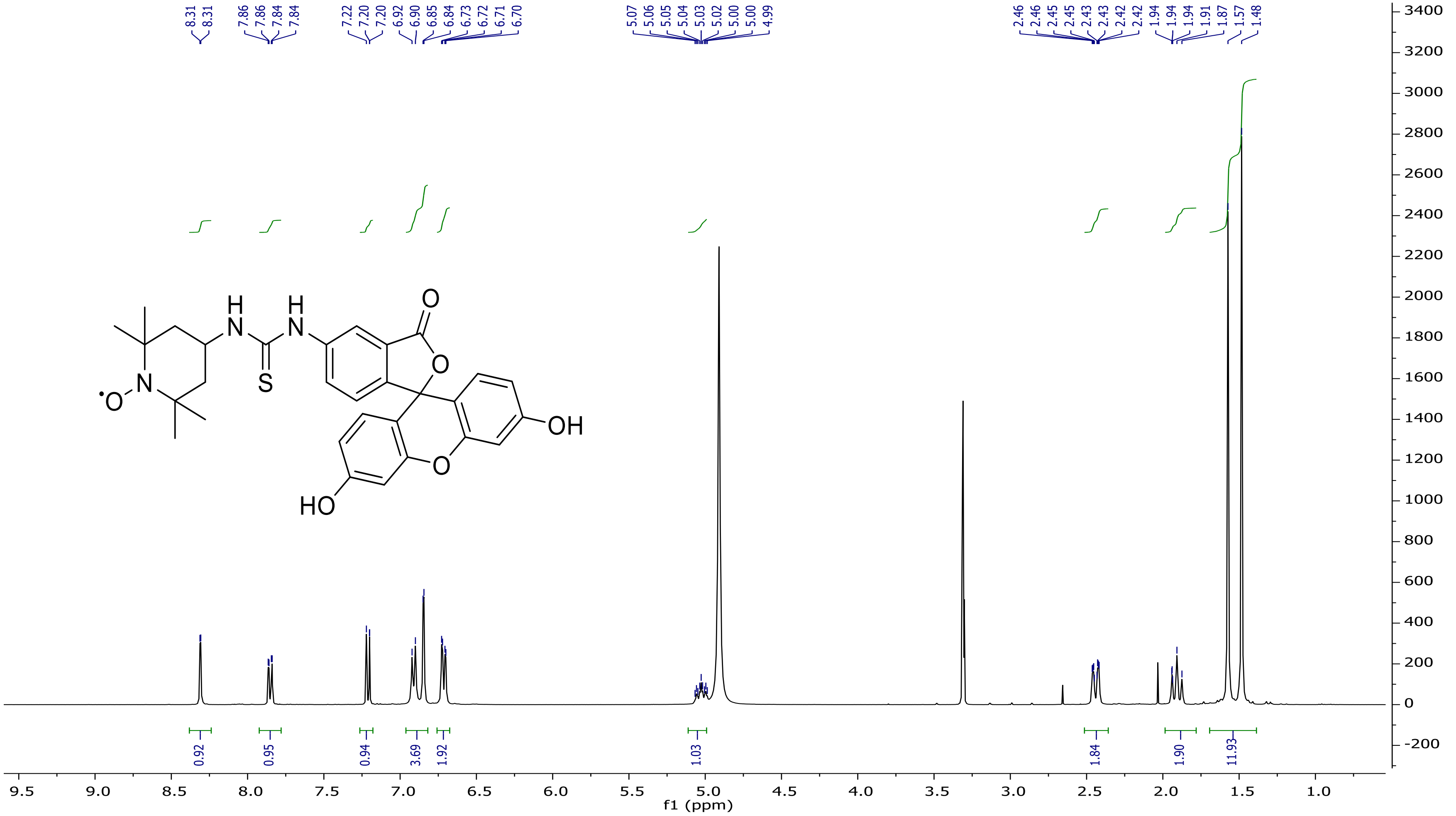

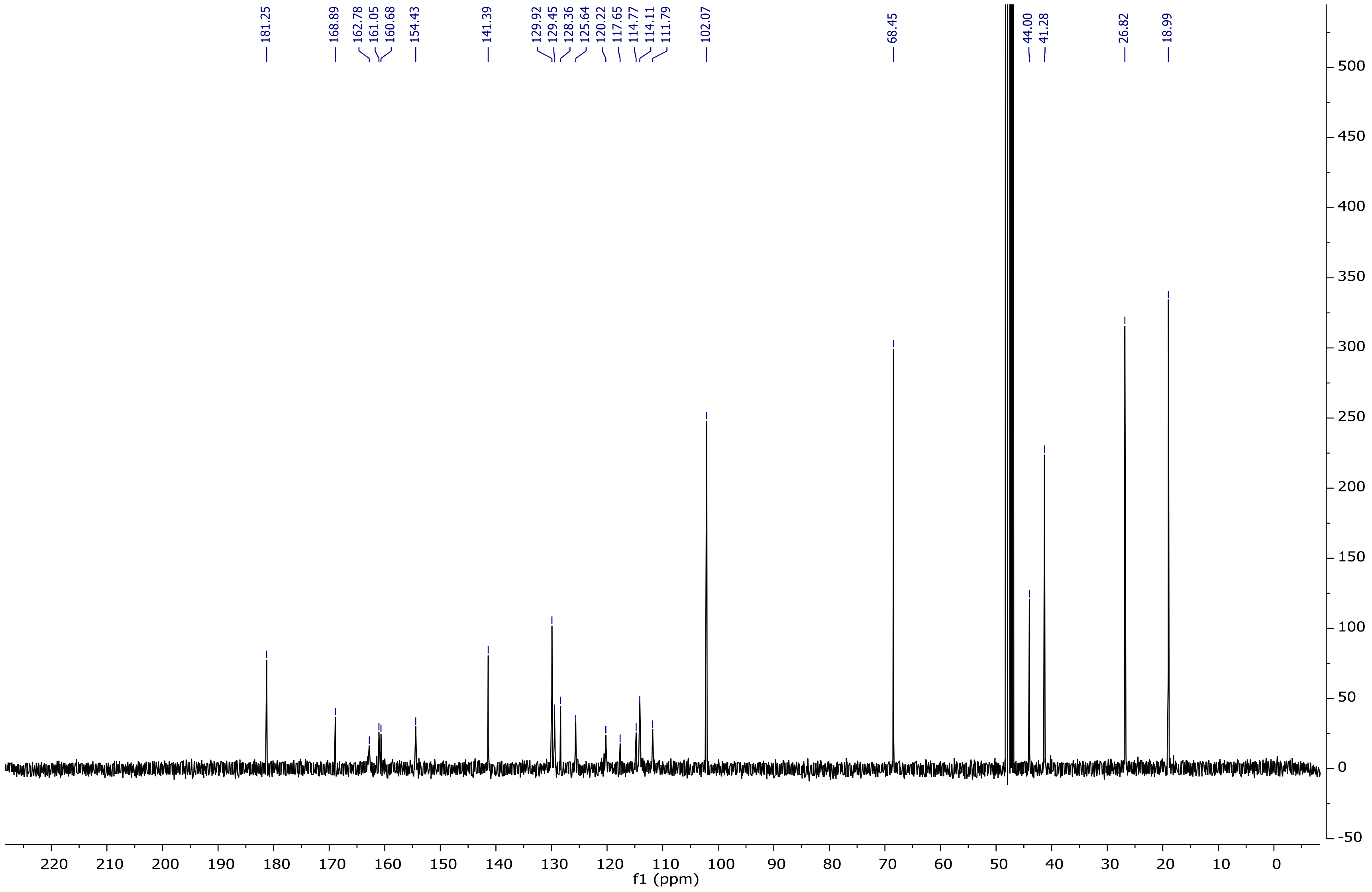
181.25
168.89
162.78
161.05
160.68
154.43
141.39
129.92
129.45
128.36
125.64
120.22
117.65
114.77
114.11
111.79
102.07
68.45
44.00
41.28
26.82
18.99
f1 (ppm)

Biotin-EDA-TEMPO (6)

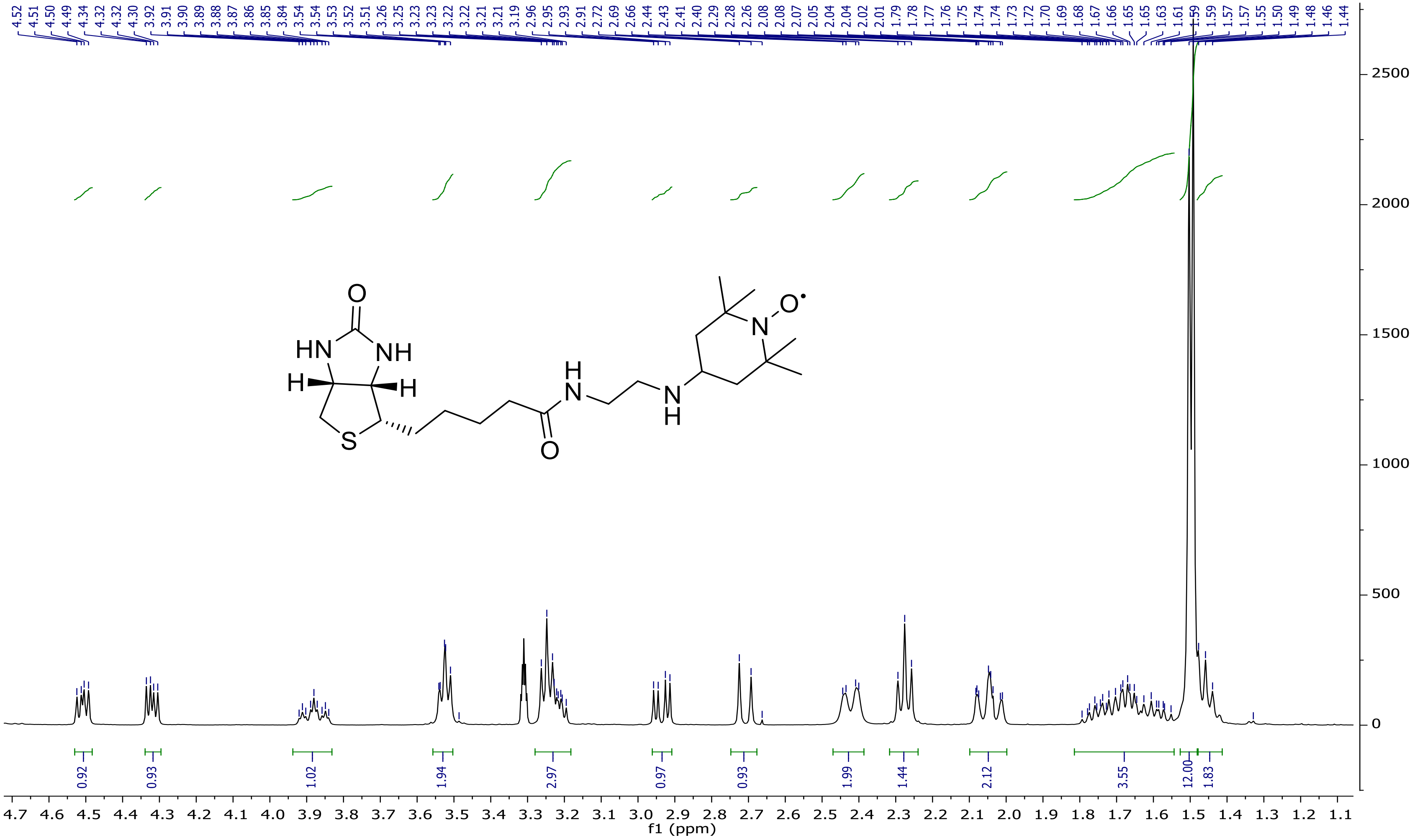

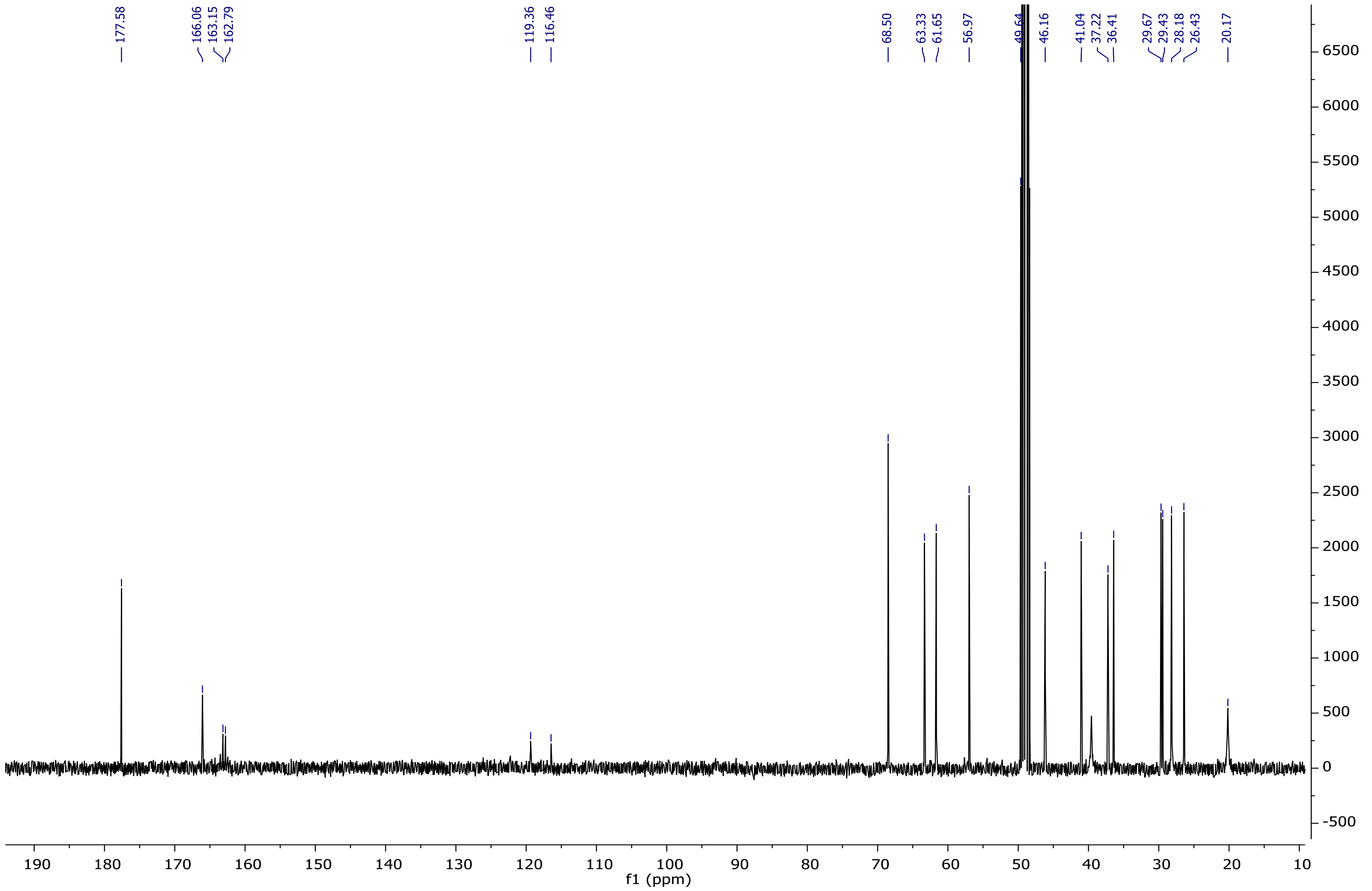
177.58
166.06
163.15
162.79
119.36
116.46
68.50
63.33
61.65
56.97
49.64
46.16
41.04
37.22
36.41
29.67
29.43
28.18
26.43
20.17
f1 (ppm)

Gemcitabine-TEMPO (7)

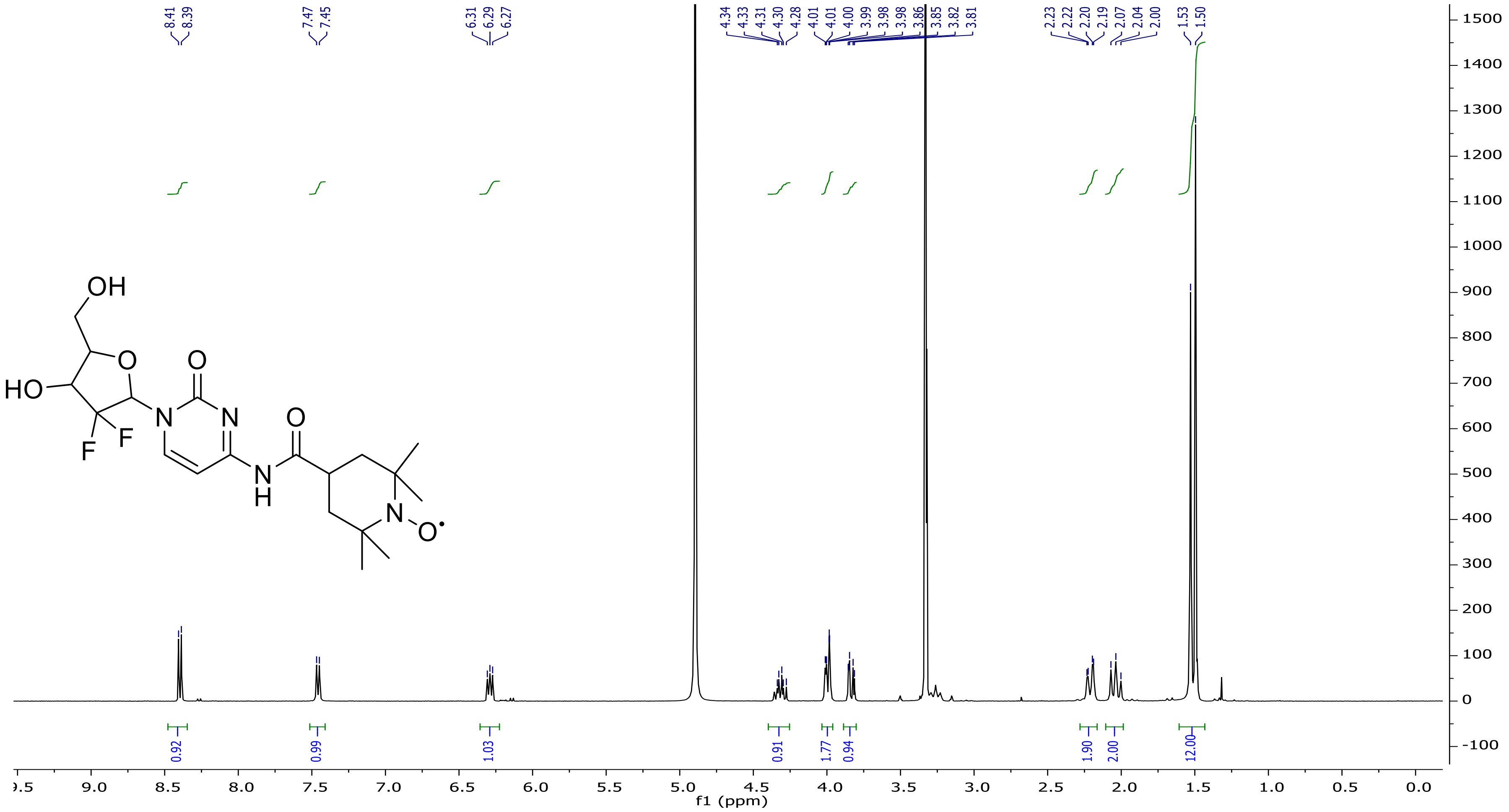

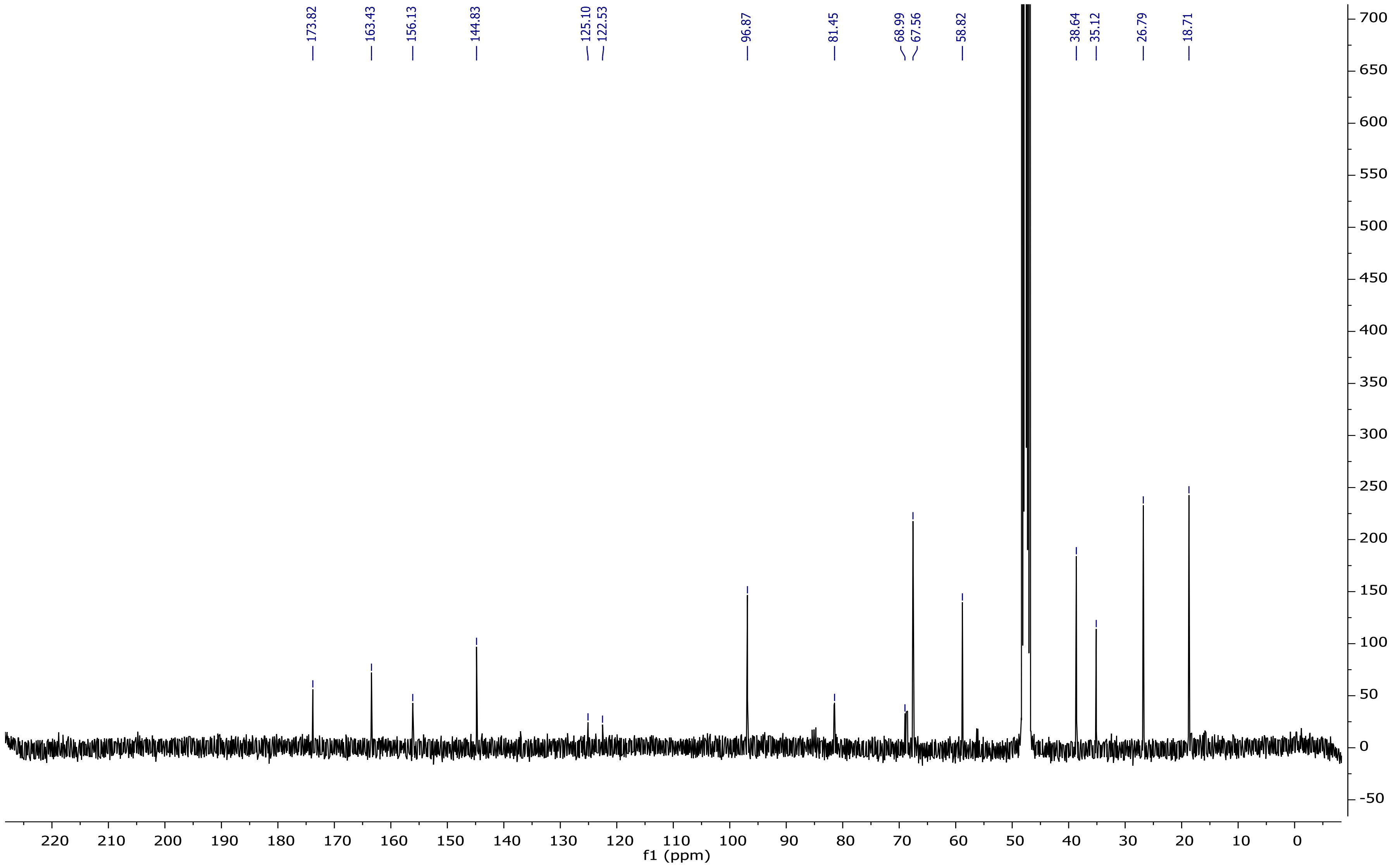
173.82
163.43
156.13
144.83
125.10
122.53
96.87
81.45
68.99
67.56
58.82
38.64
35.12
26.79
18.71
f1 (ppm)

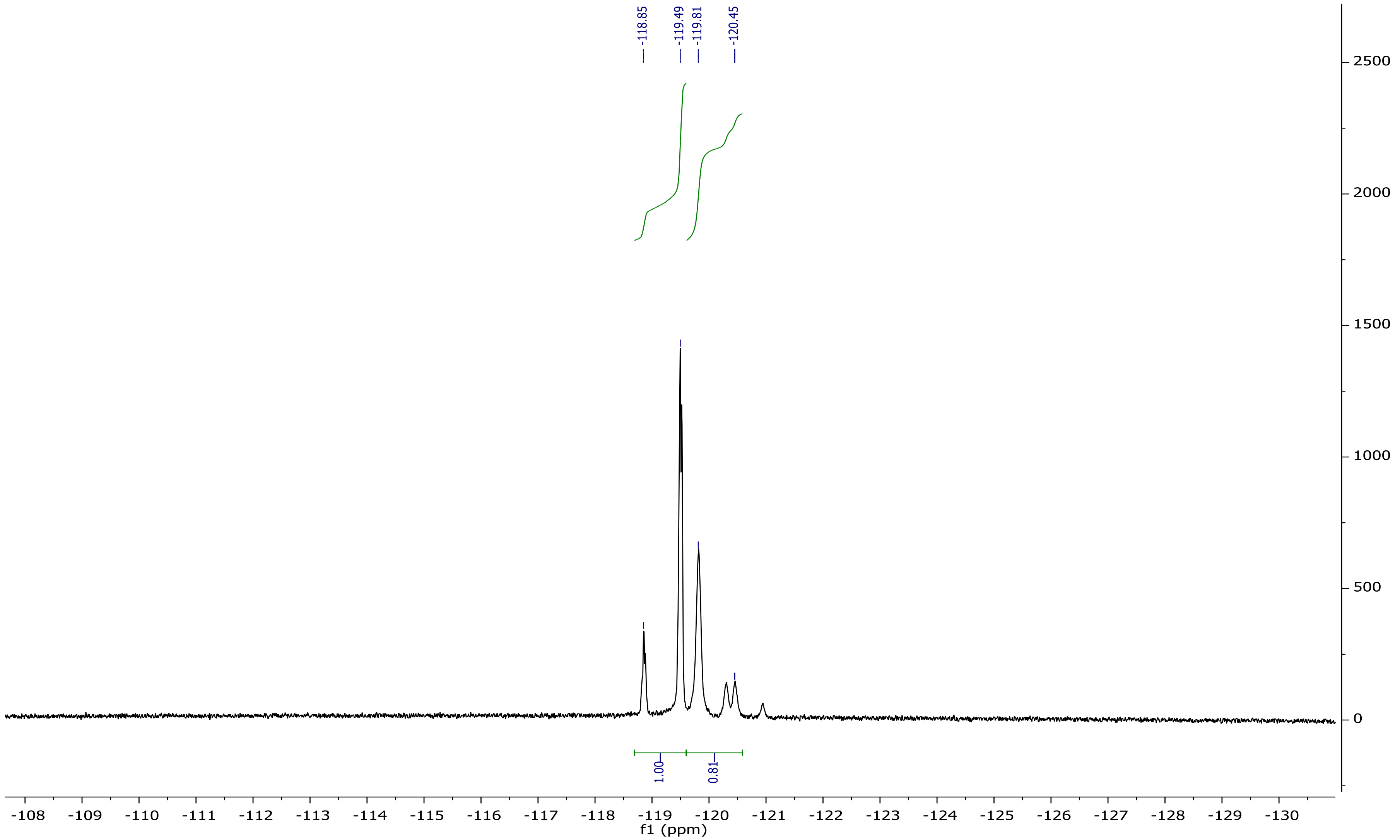

-118.85
-119.49
-119.81
-120.45
1.00
0.81
2500
2000
1500
1000
500
0
-108 -109 -110 -111 -112 -113 -114 -115 -116 -117 -118 -119 -120 -121 -122 -123 -124 -125 -126 -127 -128 -129 -130
f1 (ppm)

H-UAF-OMe with tetra-EG-TEMPO (8)

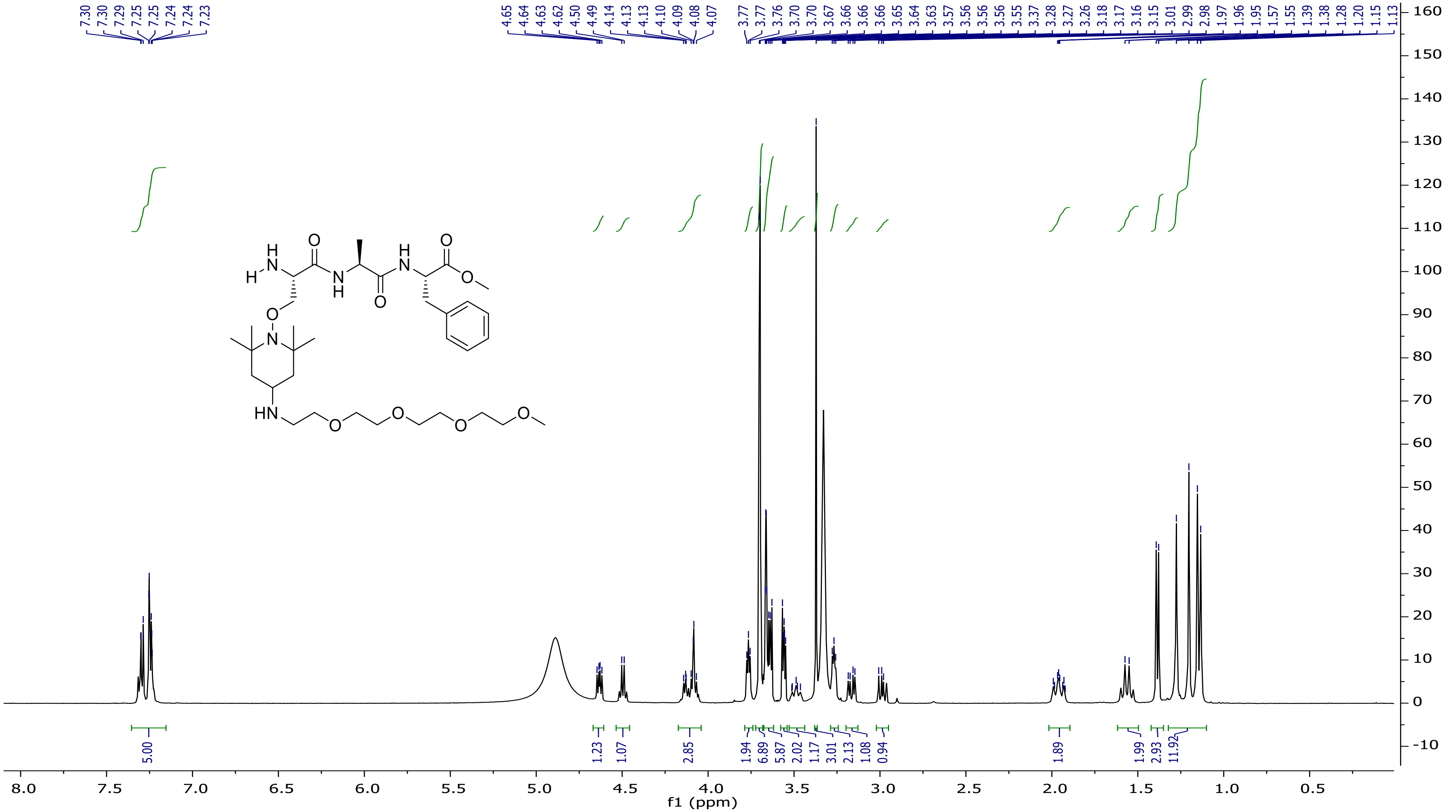

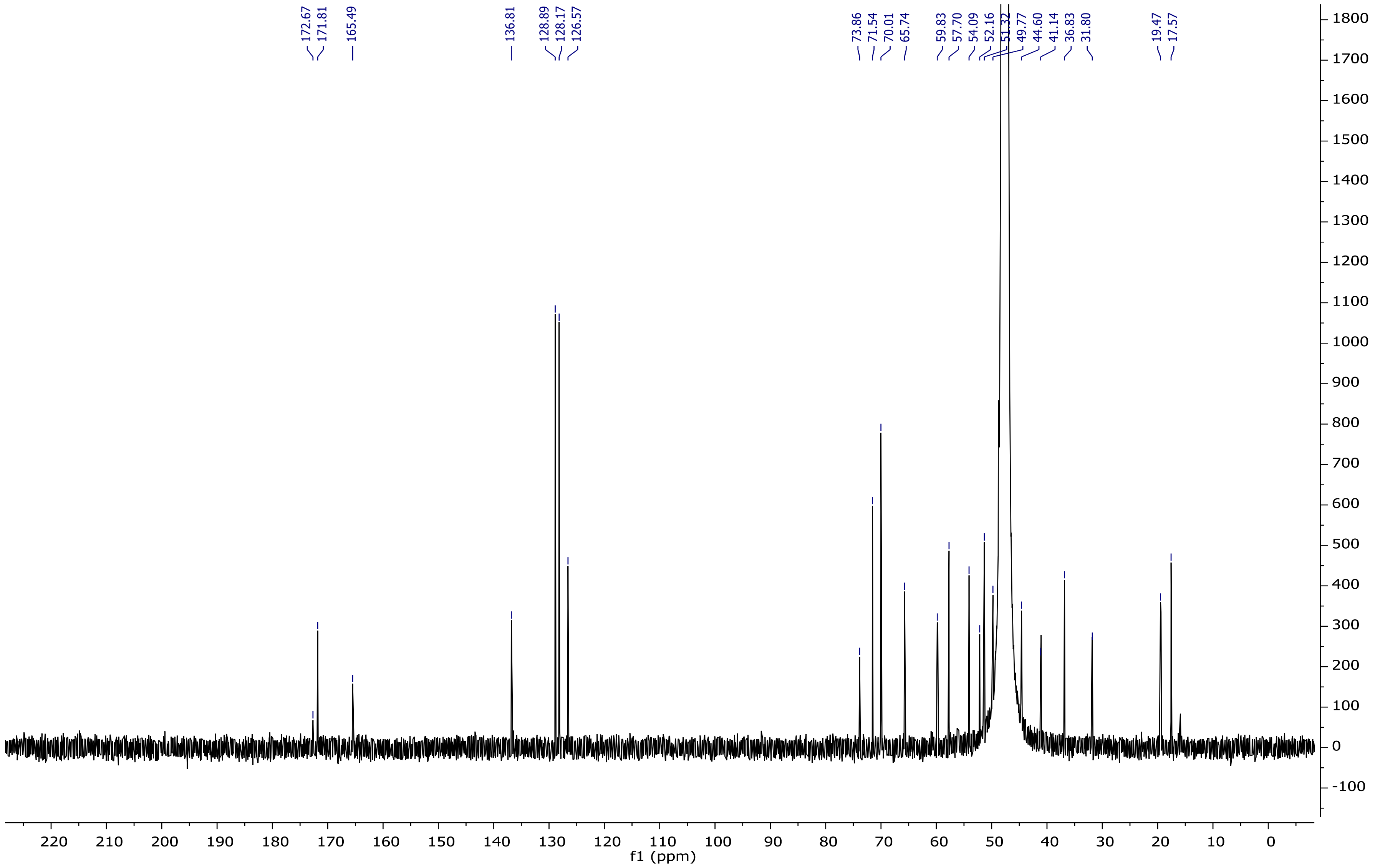

172.67
171.81
165.49
136.81
128.89
128.17
126.57
73.86
71.54
70.01
65.74
59.83
57.70
54.09
52.16
51.32
49.77
44.60
41.14
36.83
31.80
19.47
17.57
f1 (ppm)

H-UAF-OMe with Propargyl TEMPO (9)

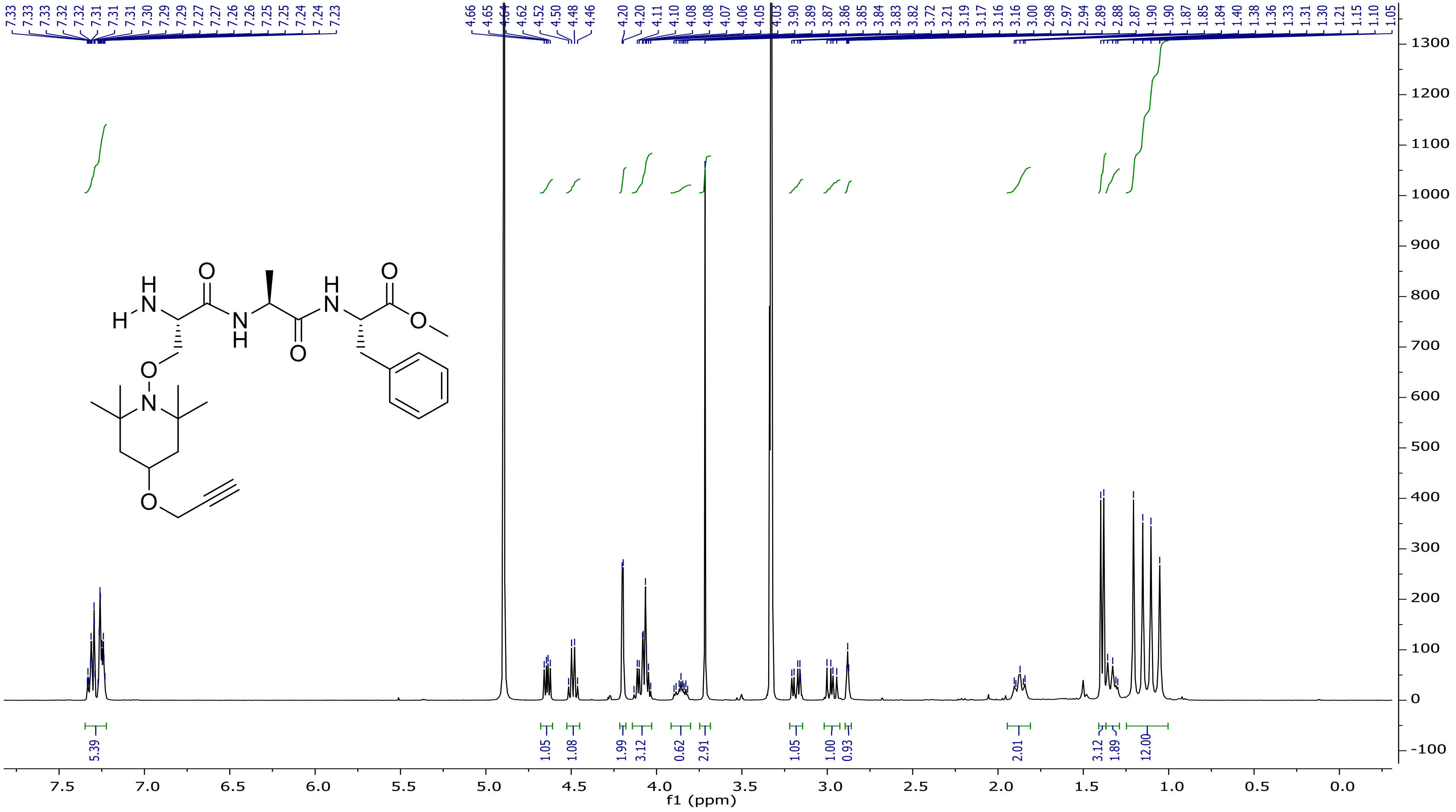

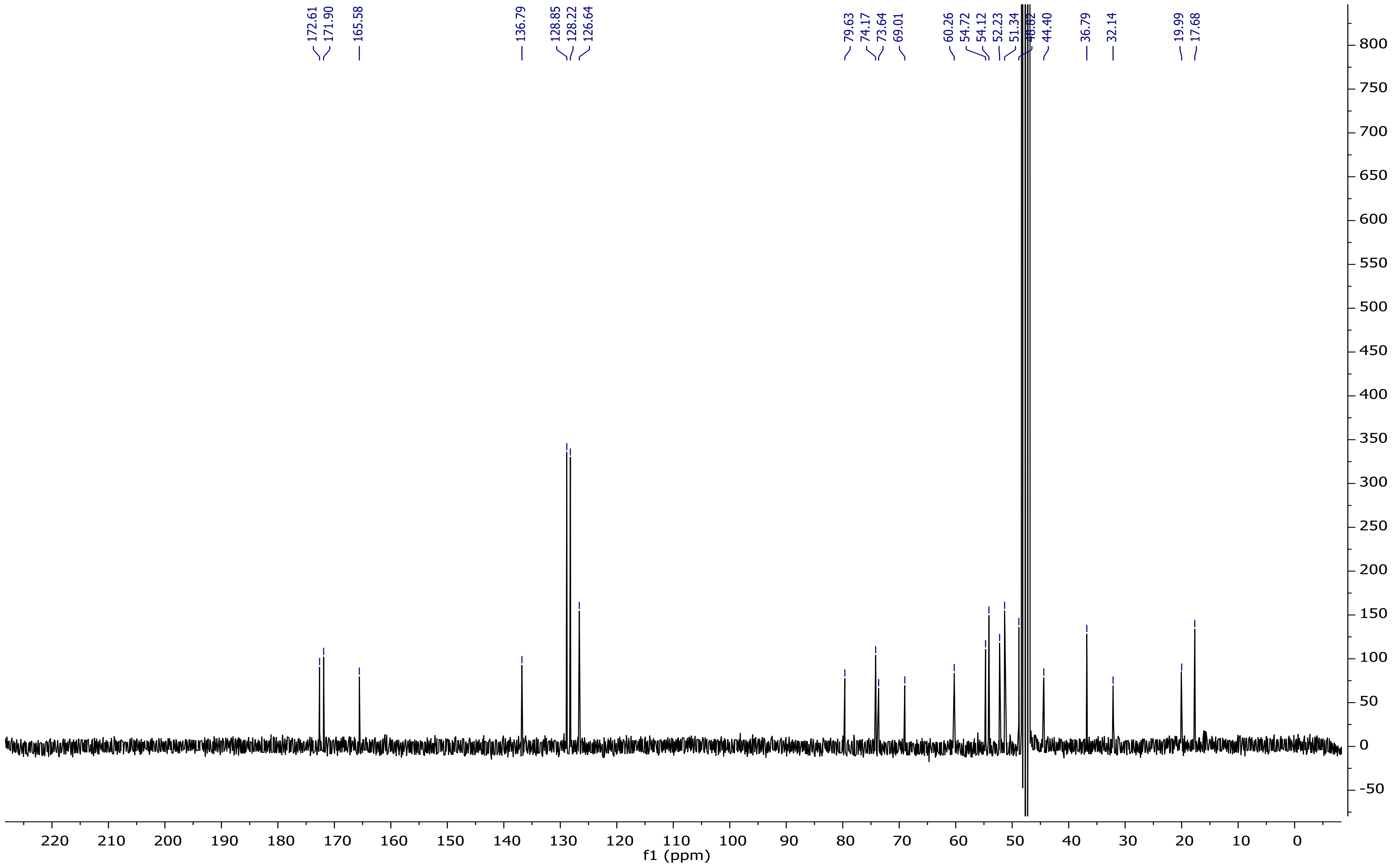

172.61
171.90
165.58
136.79
128.85
128.22
126.64
79.63
74.17
73.64
69.01
60.26
54.72
54.12
52.23
51.34
48.82
44.40
36.79
32.14
19.99
17.68
f1 (ppm)

H-UAF-OMe with FITC-TEMPO (10)

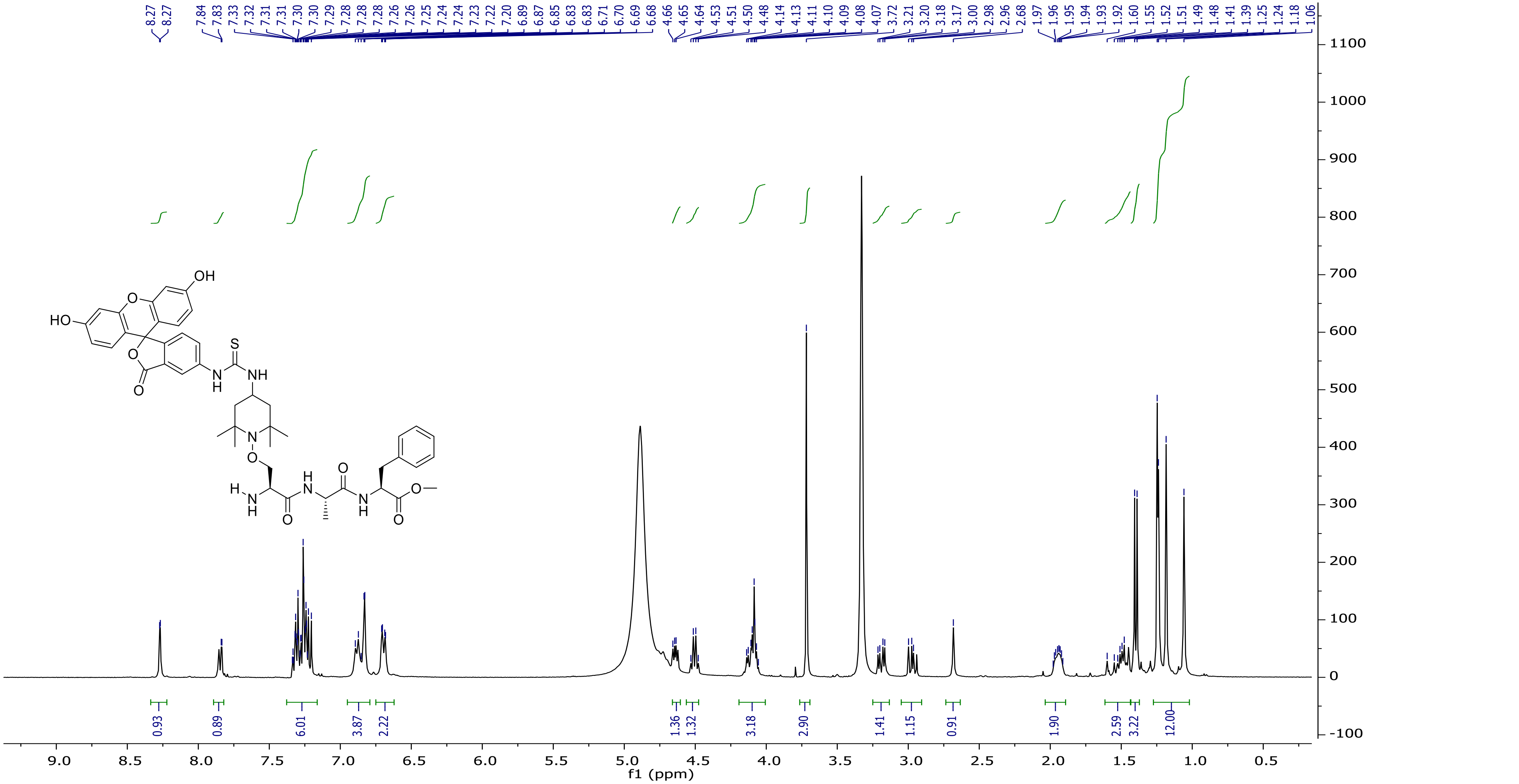

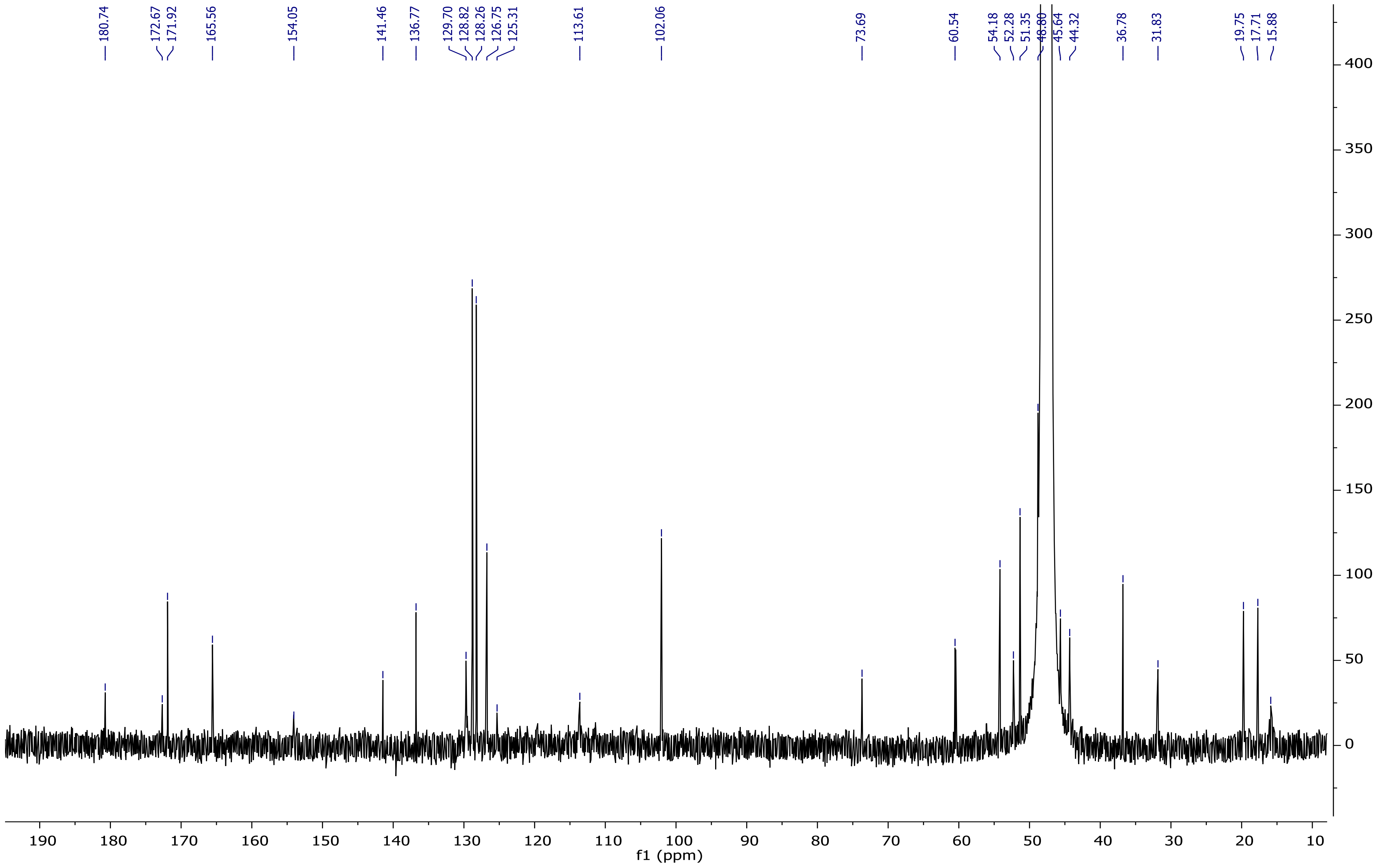

180.74
172.67
171.92
165.56
154.05
141.46
136.77
129.70
128.82
128.26
126.75
125.31
113.61
102.06
73.69
60.54
54.18
52.28
51.35
48.80
45.64
44.32
36.78
31.83
19.75
17.71
15.88
400
350
300
250
200
150
100
50
0
190
180
170
160
150
140
130
120
110
100
90
80
70
60
50
40
30
20
10
f1 (ppm)

H-UAF-OMe with Biotin-EDA-TEMPO (11)

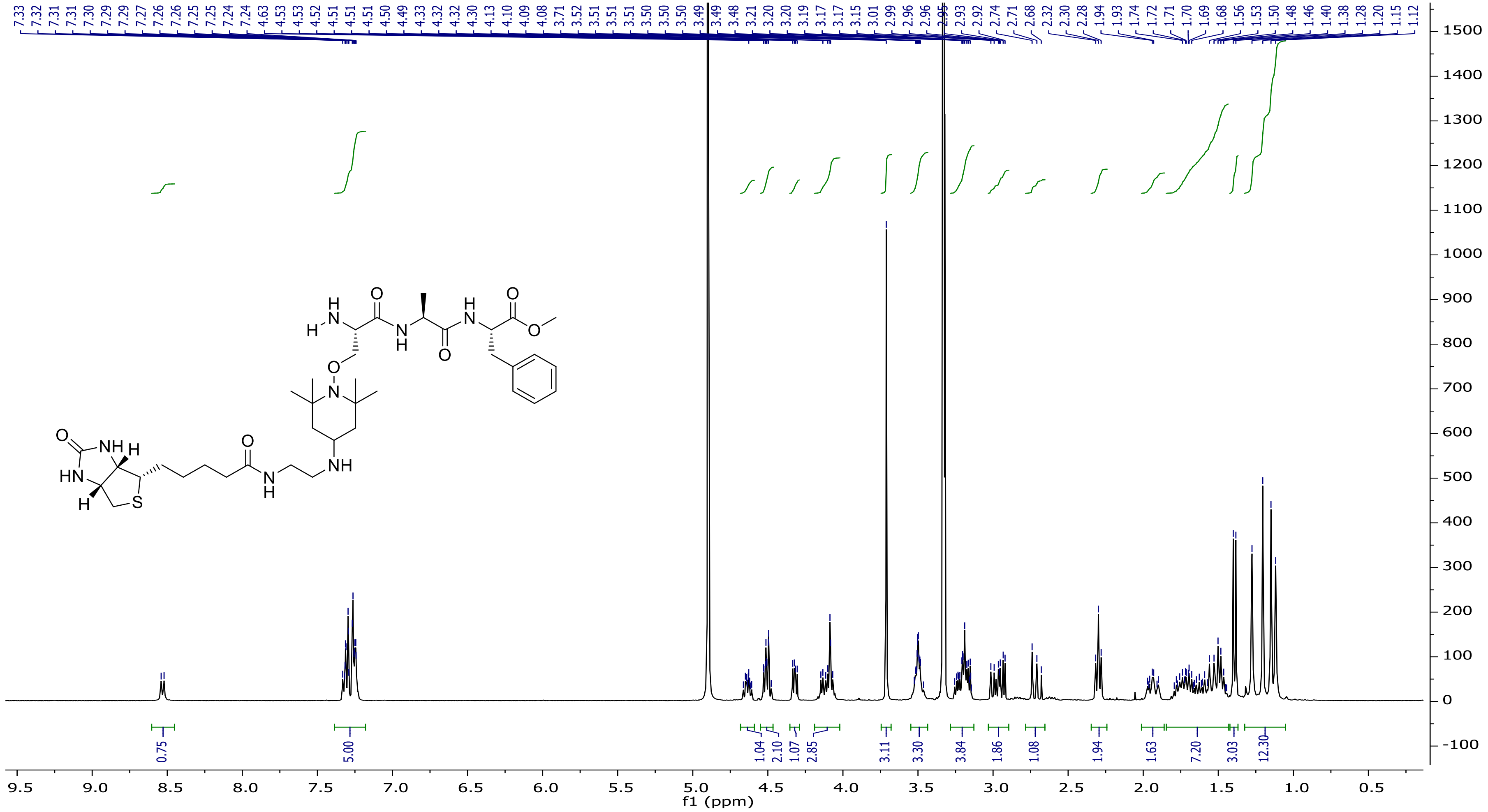

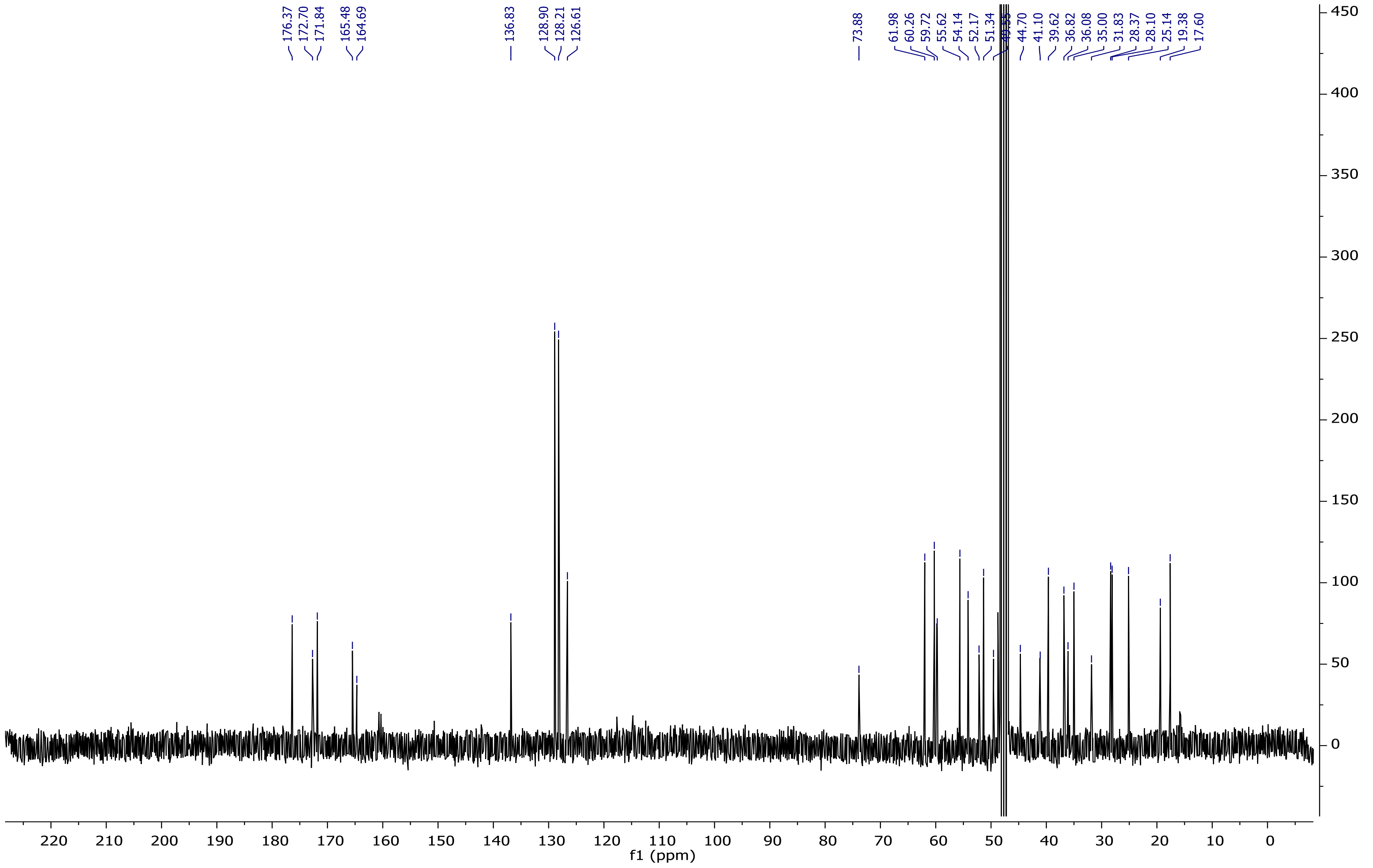
176.37
172.70
171.84
165.48
164.69
136.83
128.90
128.21
126.61
73.88
61.98
60.26
59.72
55.62
54.14
52.17
51.34
49.55
44.70
41.10
39.62
36.82
36.08
35.00
31.83
28.37
28.10
25.14
19.38
17.60
450
400
350
300
250
200
150
100
50
0
220 210 200 190 180 170 160 150 140 130 120 110 100 90 80 70 60 50 40 30 20 10 0
f1 (ppm)

H-UAF-OMe with Gemcitabine-TEMPO (12)

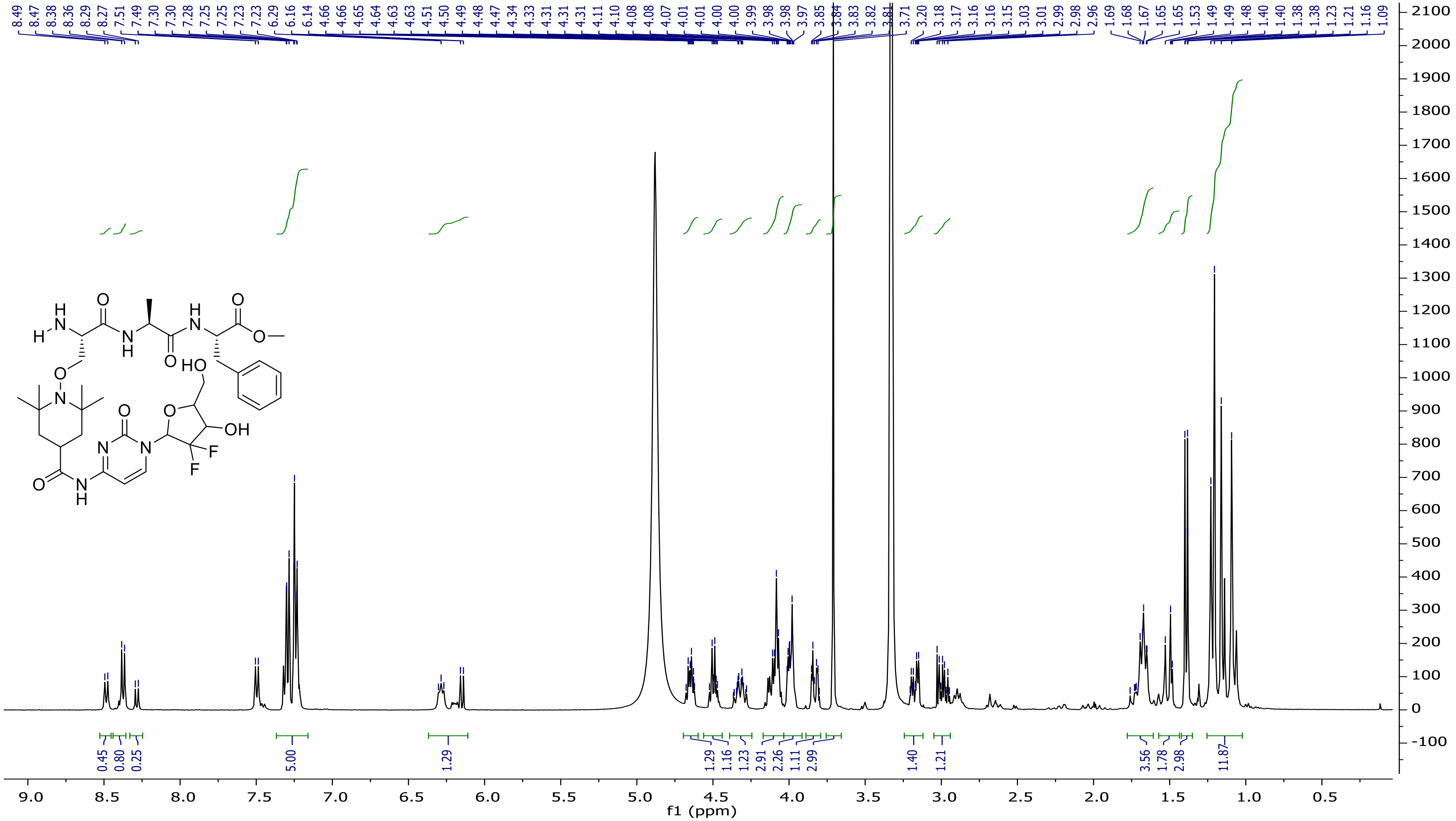

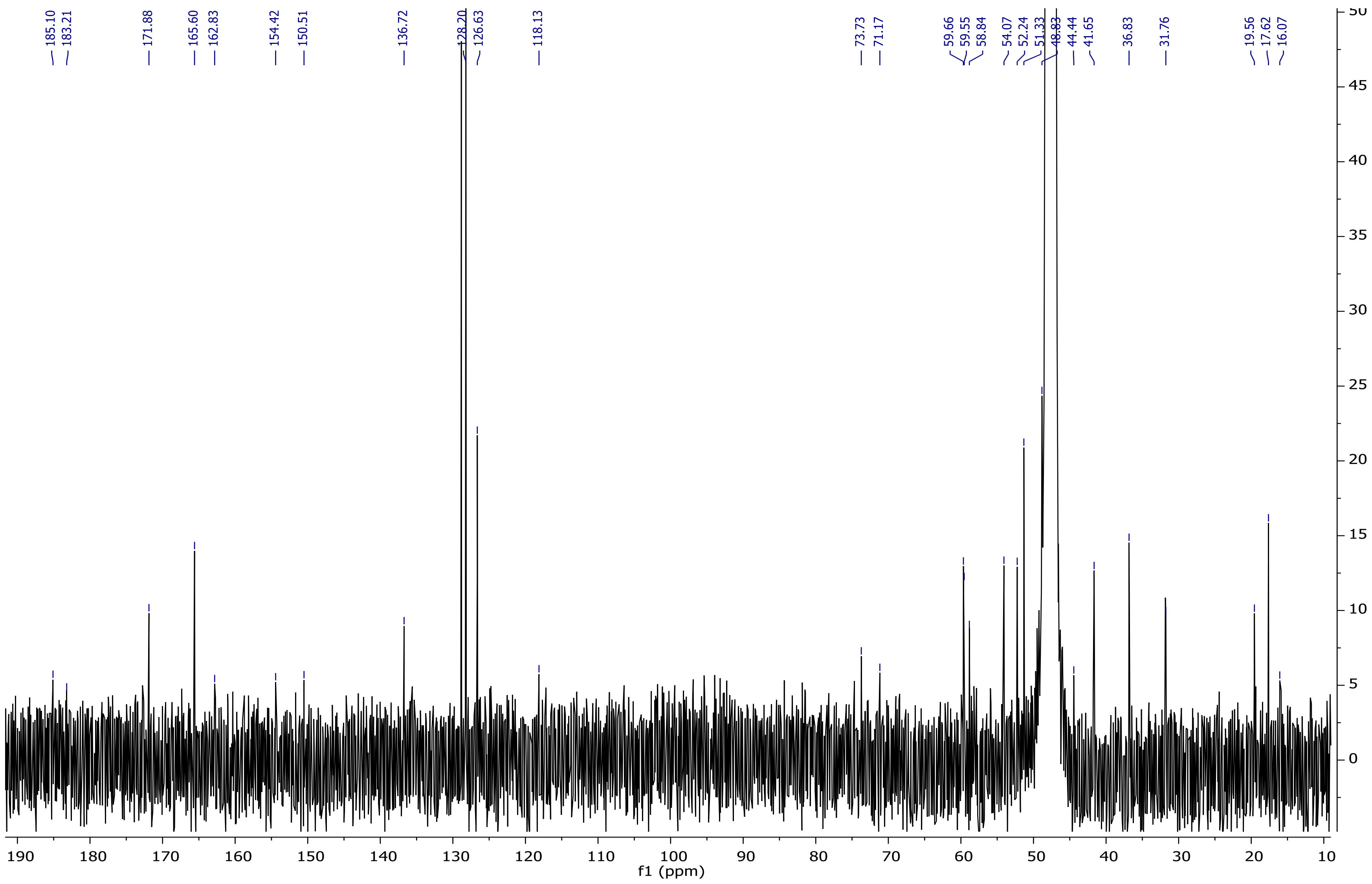
185.10
183.21
171.88
165.60
162.83
154.42
150.51
136.72
128.20
126.63
118.13
73.73
71.17
59.66
59.55
58.84
54.07
52.24
51.33
48.83
44.44
41.65
36.83
31.76
19.56
17.62
16.07
f1 (ppm)

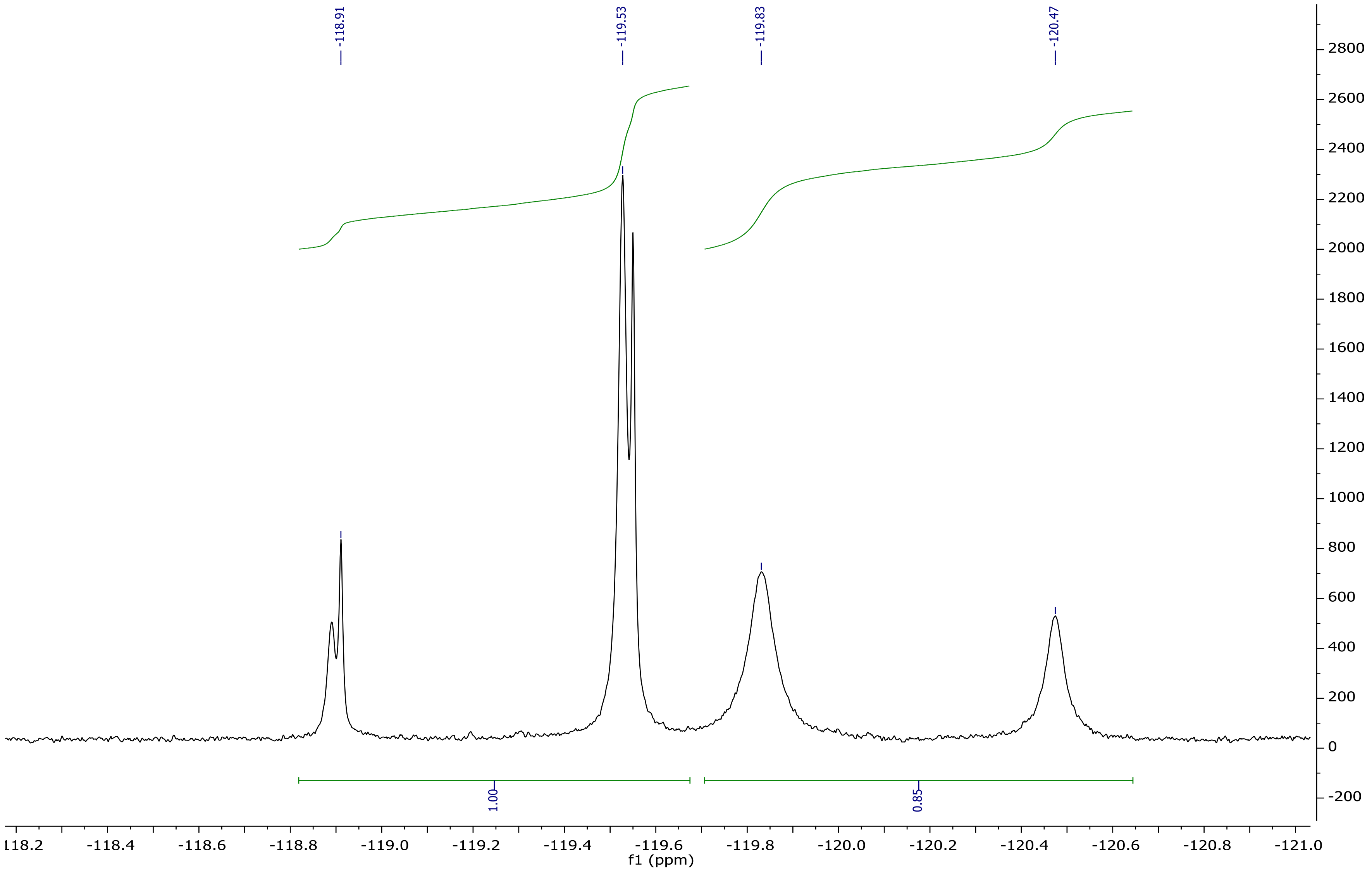

-118.91
-119.53
-119.83
-120.47
1.00
0.85
f1 (ppm)

H-CAF-OMe (13)

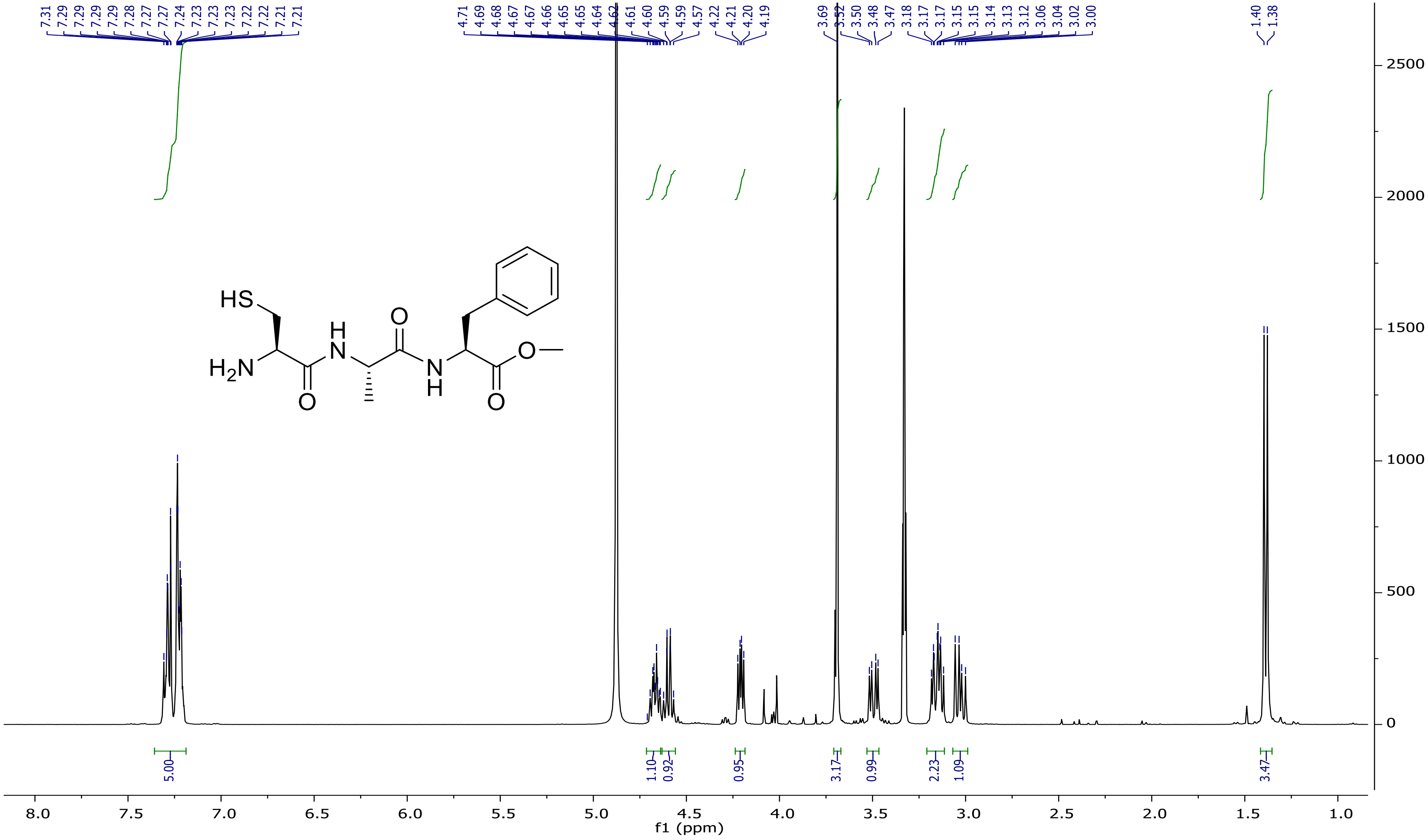

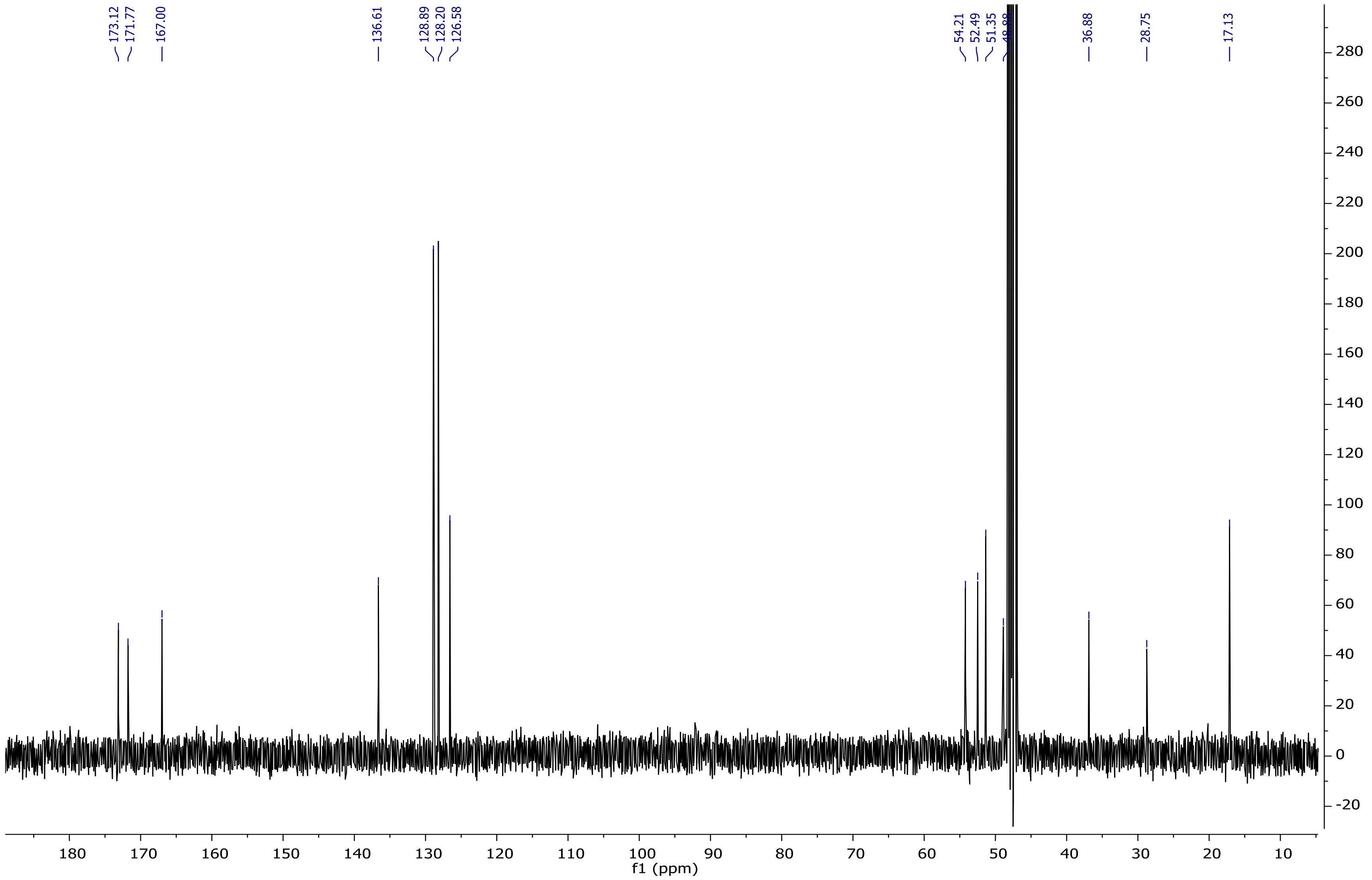
173.12
171.77
167.00
136.61
128.89
128.20
126.58
54.21
52.49
51.35
36.88
28.75
17.13
f1 (ppm)

H-CAF-OMe with TEMPO (14)

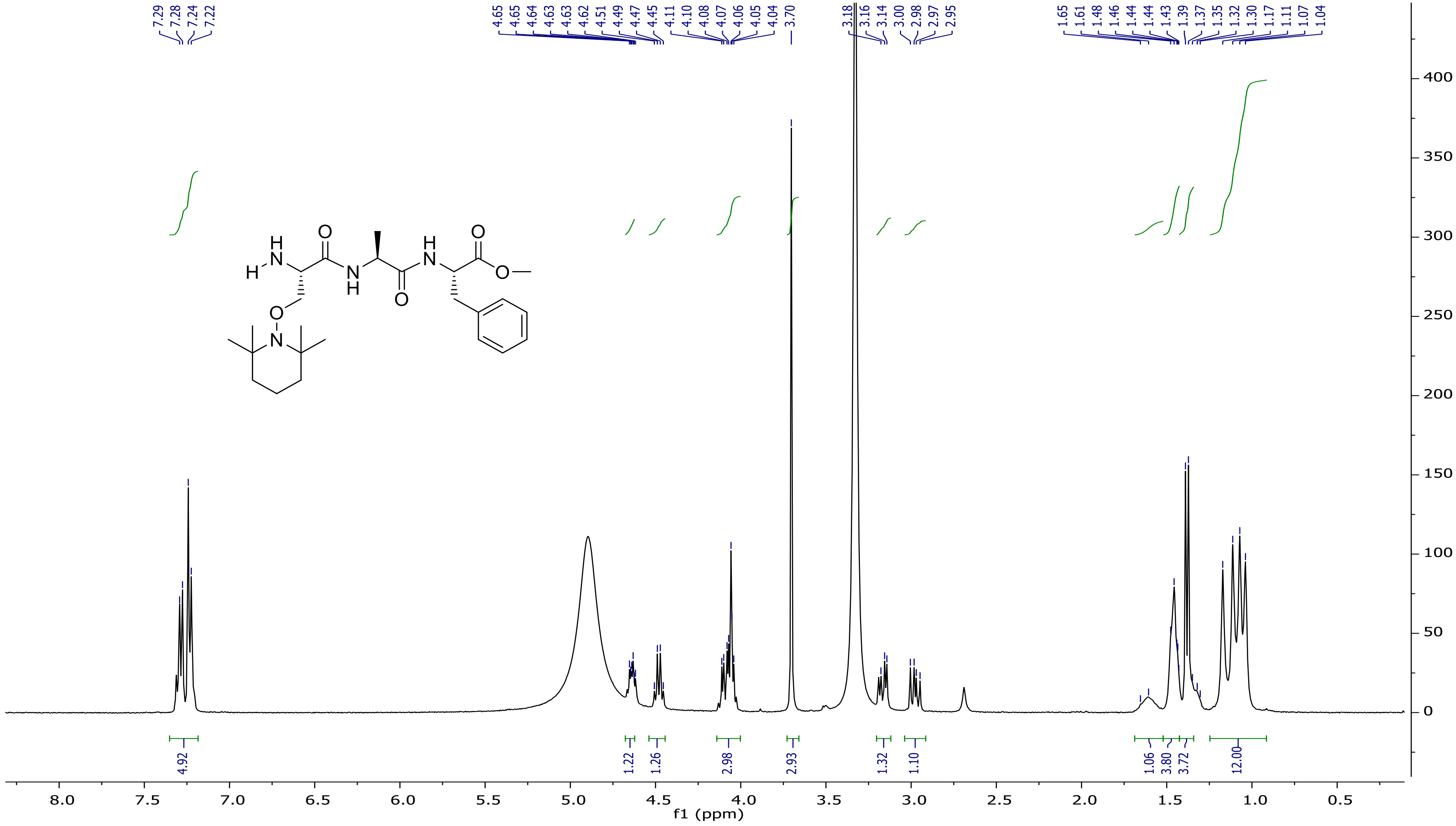

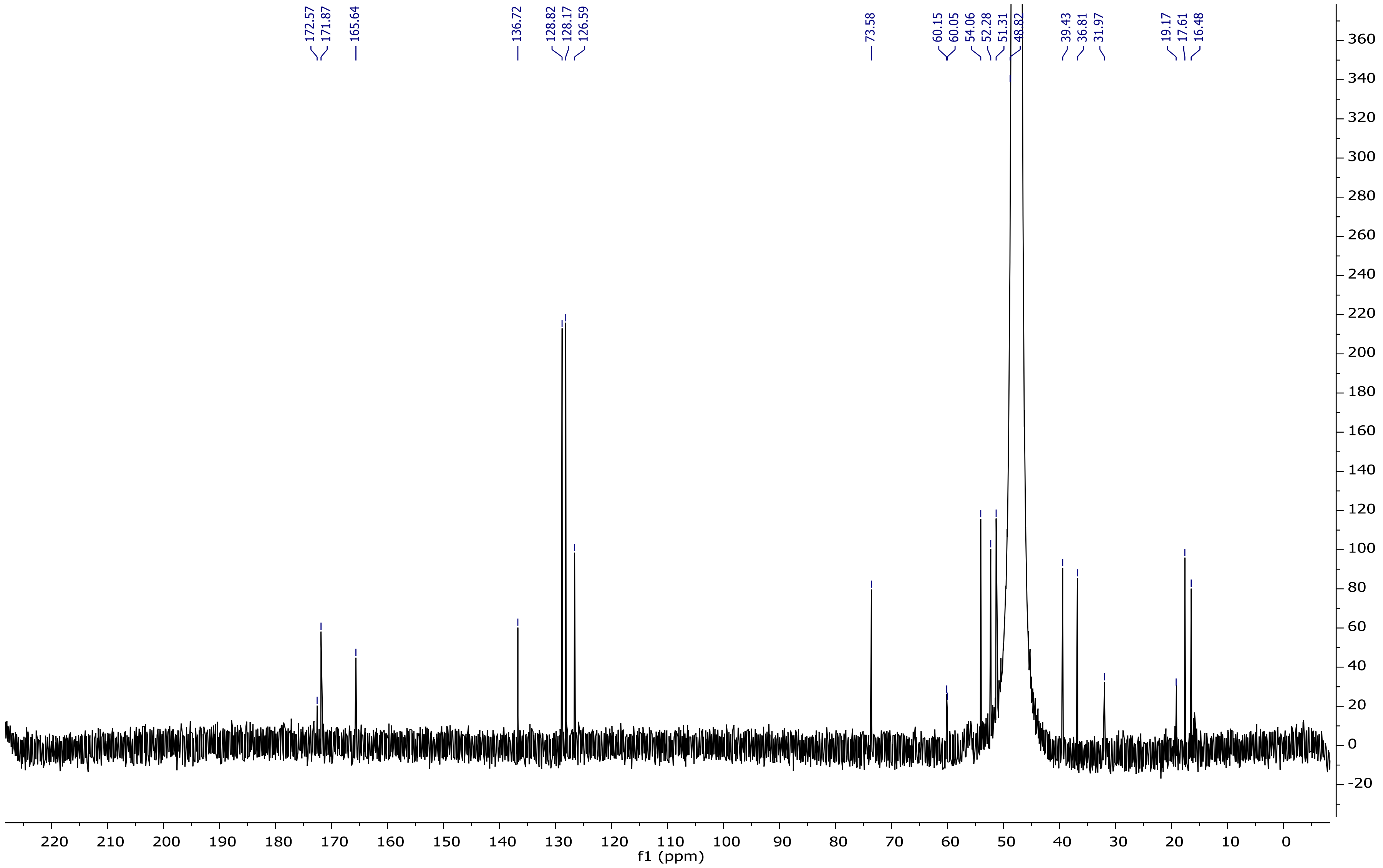
172.57
171.87
165.64
136.72
128.82
128.17
126.59
73.58
60.15
60.05
54.06
52.28
51.31
48.82
39.43
36.81
31.97
19.17
17.61
16.48
f1 (ppm)

Methyl 2-((tert-butoxycarbony)amine)-3-iodopropanoate (49)

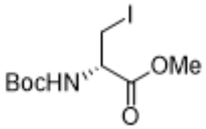

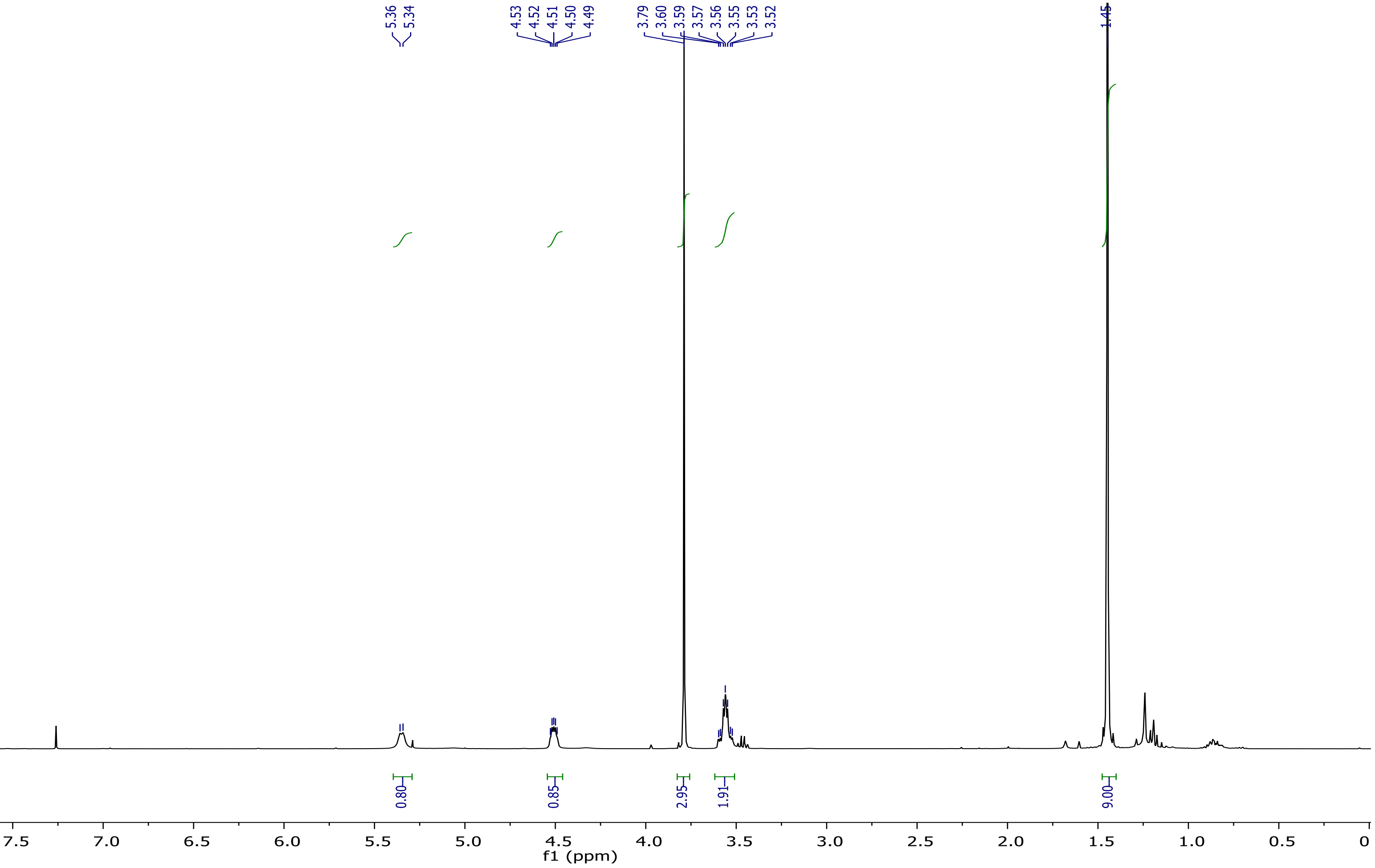

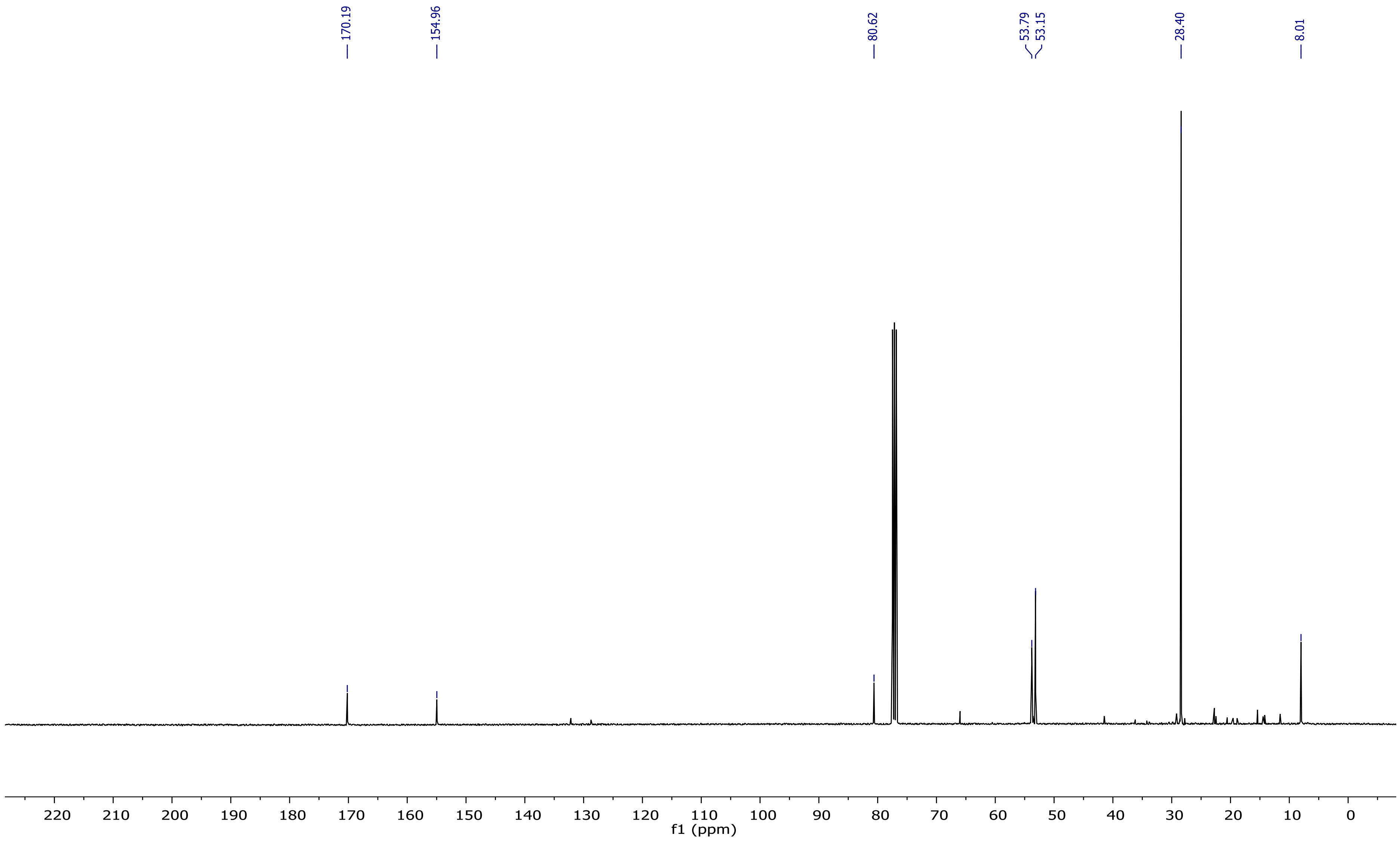
170.19
154.96
80.62
53.79
53.15
28.40
8.01
220 210 200 190 180 170 160 150 140 130 120 110 100 90 80 70 60 50 40 30 20 10 0
f1 (ppm)

Methyl-2-((tert-butoxycarbonyl)amino)-3-selenocyanatopropanoate (50)

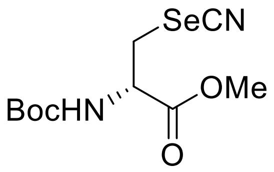

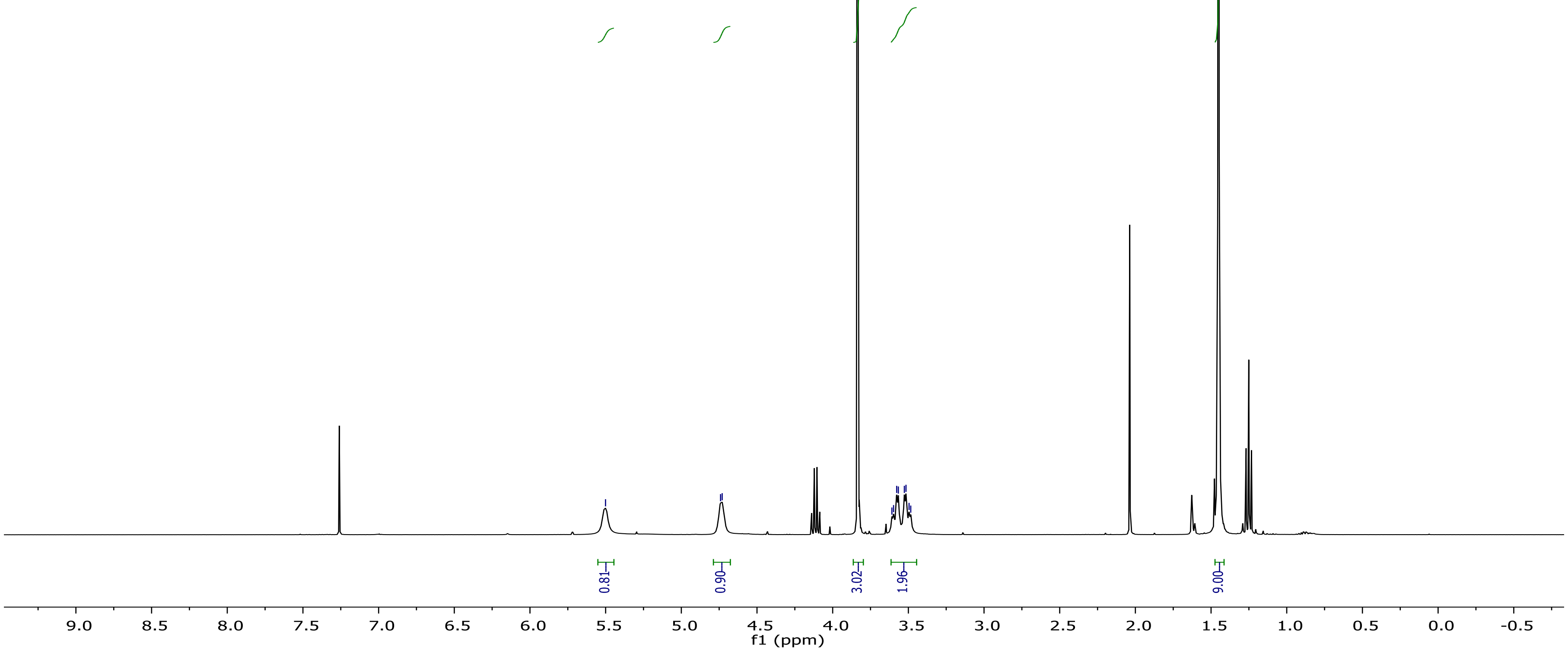

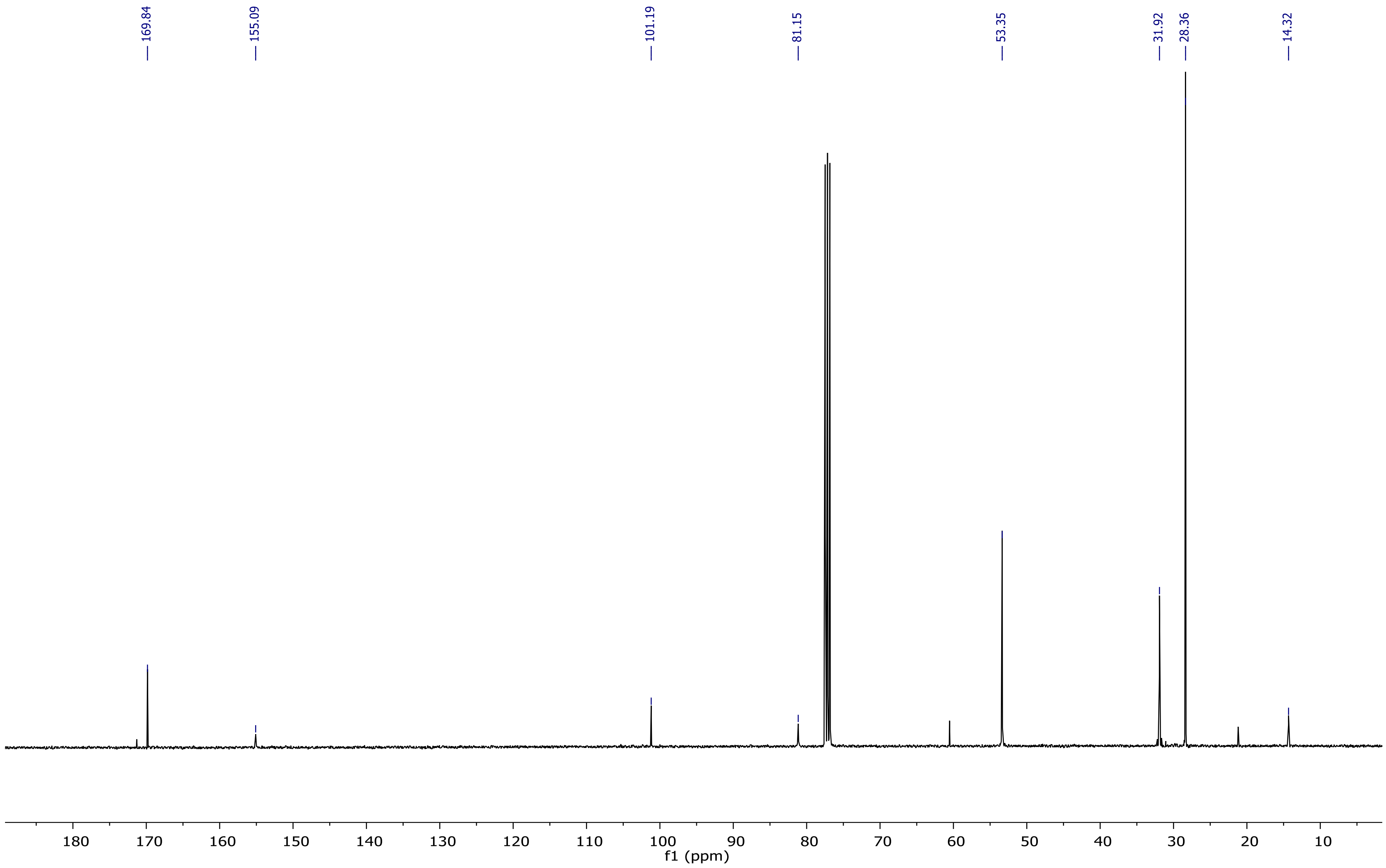
169.84
155.09
101.19
81.15
53.35
31.92
28.36
14.32
180 170 160 150 140 130 120 110 100 90 80 70 60 50 40 30 20 10
f1 (ppm)

Methyl-2-((*tert*-butoxycarbonyl)amino)-3-(methylselanyl)propanoate (51)

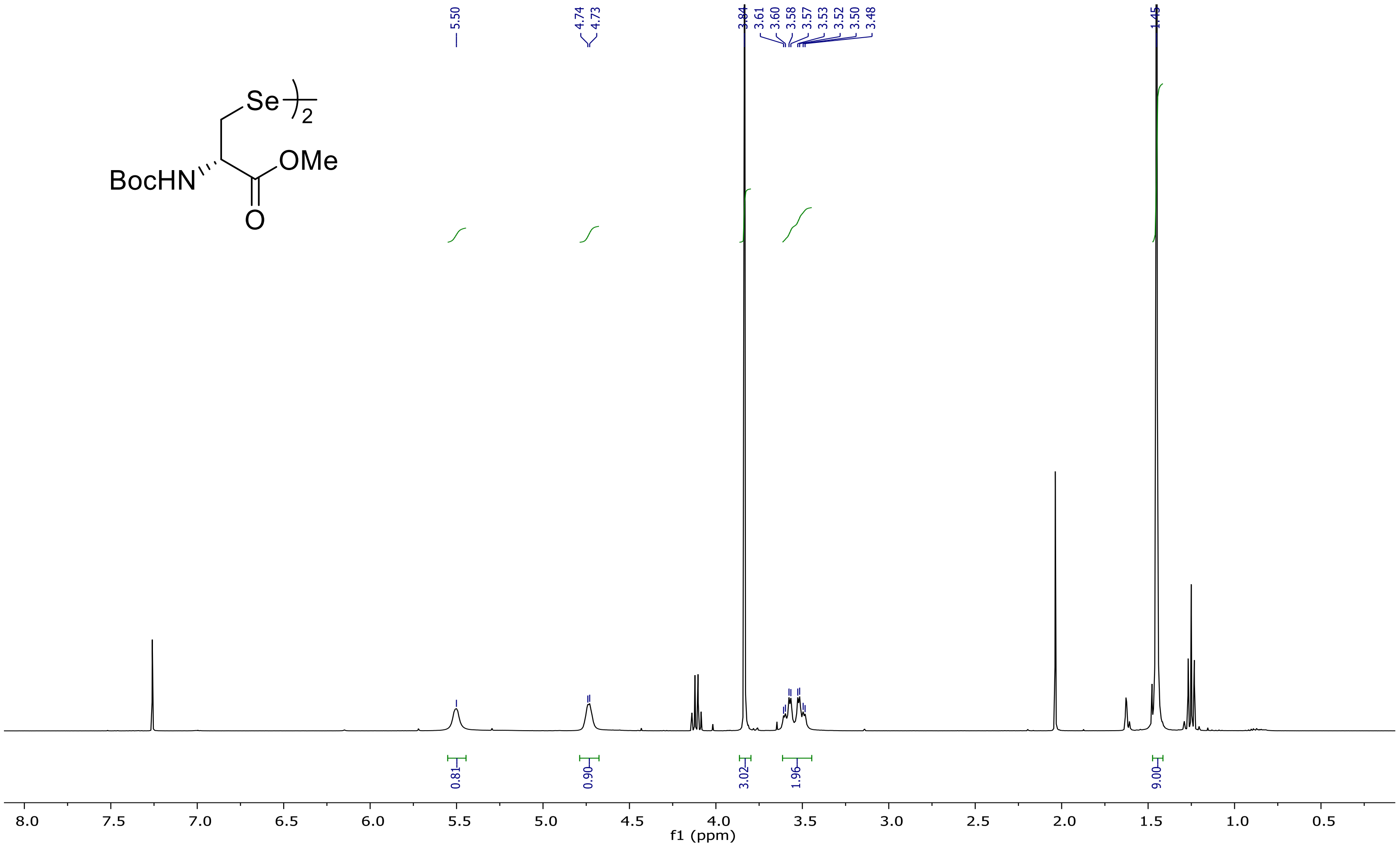

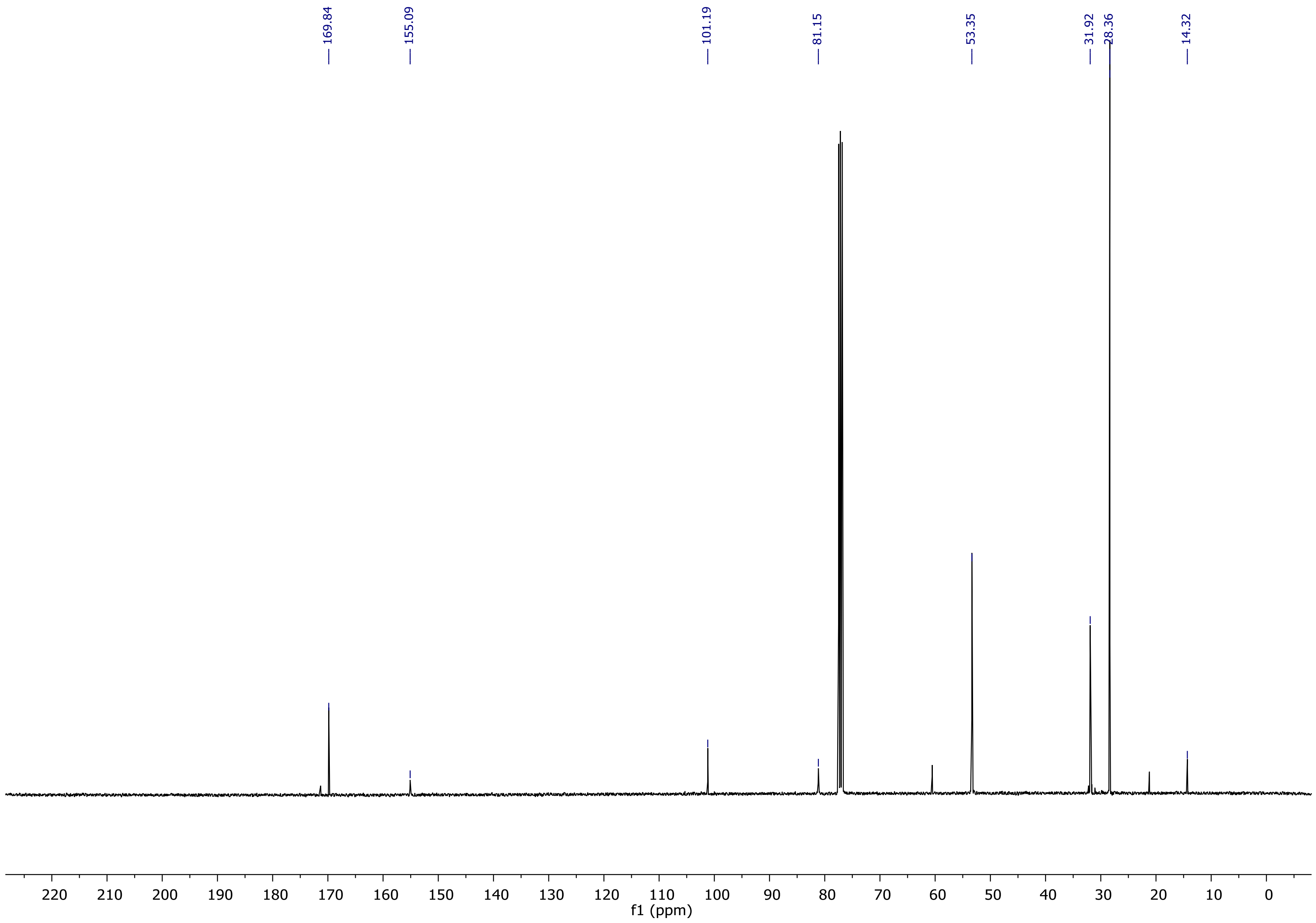
169.84
155.09
101.19
81.15
53.35
31.92
28.36
14.32
220 210 200 190 180 170 160 150 140 130 120 110 100 90 80 70 60 50 40 30 20 10 0
f1 (ppm)

H-AF-OMe (53)

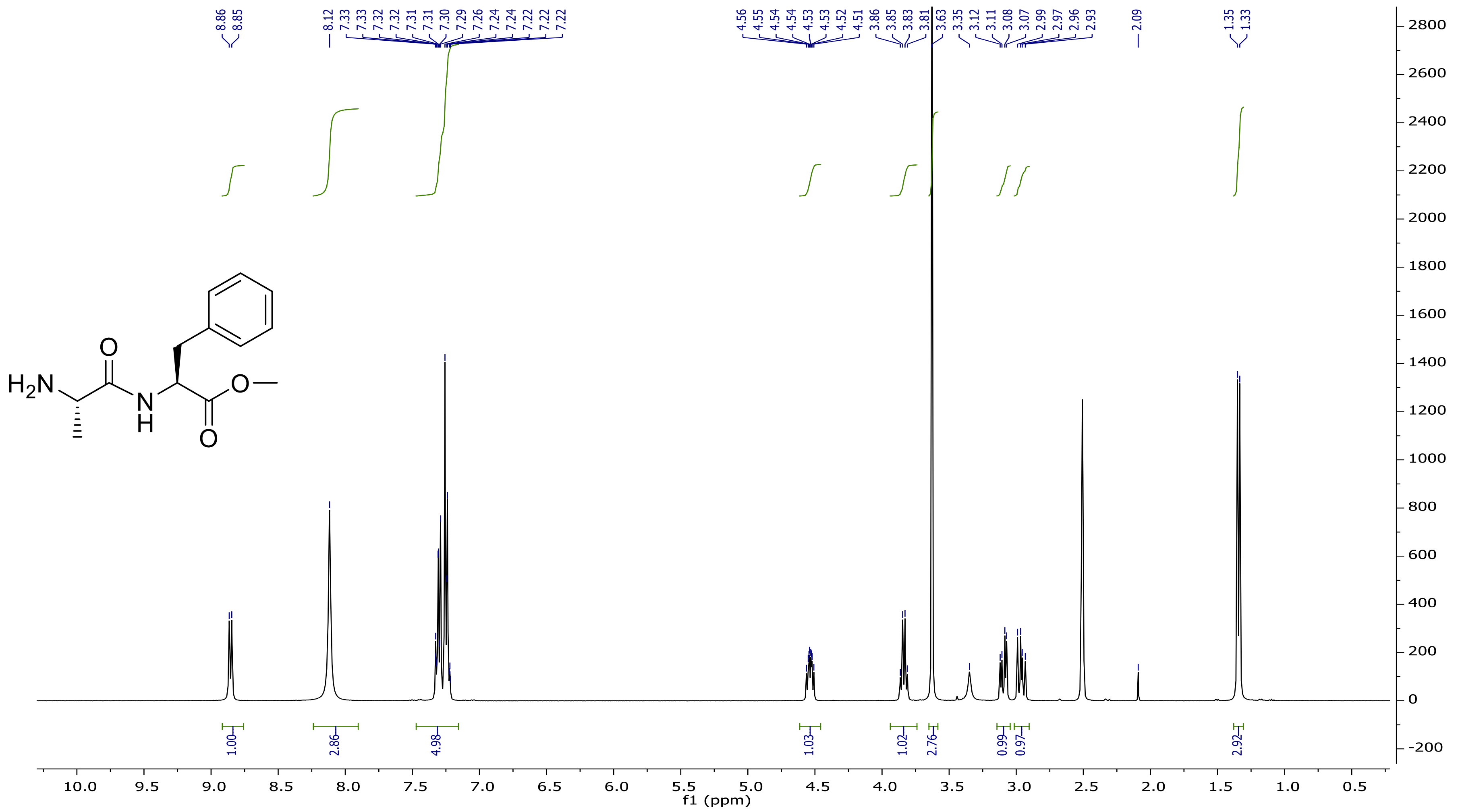

8.86
8.85
8.12
7.33
7.33
7.32
7.32
7.31
7.31
7.30
7.29
7.26
7.24
7.24
7.22
7.22
7.22
4.56
4.55
4.54
4.54
4.53
4.53
4.52
4.51
3.86
3.85
3.83
3.81
3.63
3.35
3.12
3.11
3.08
3.07
2.99
2.97
2.96
2.93
2.09
1.35
1.33
1.00
2.86
4.98
1.03
1.02
2.76
0.99
0.97
2.92
f1 (ppm)

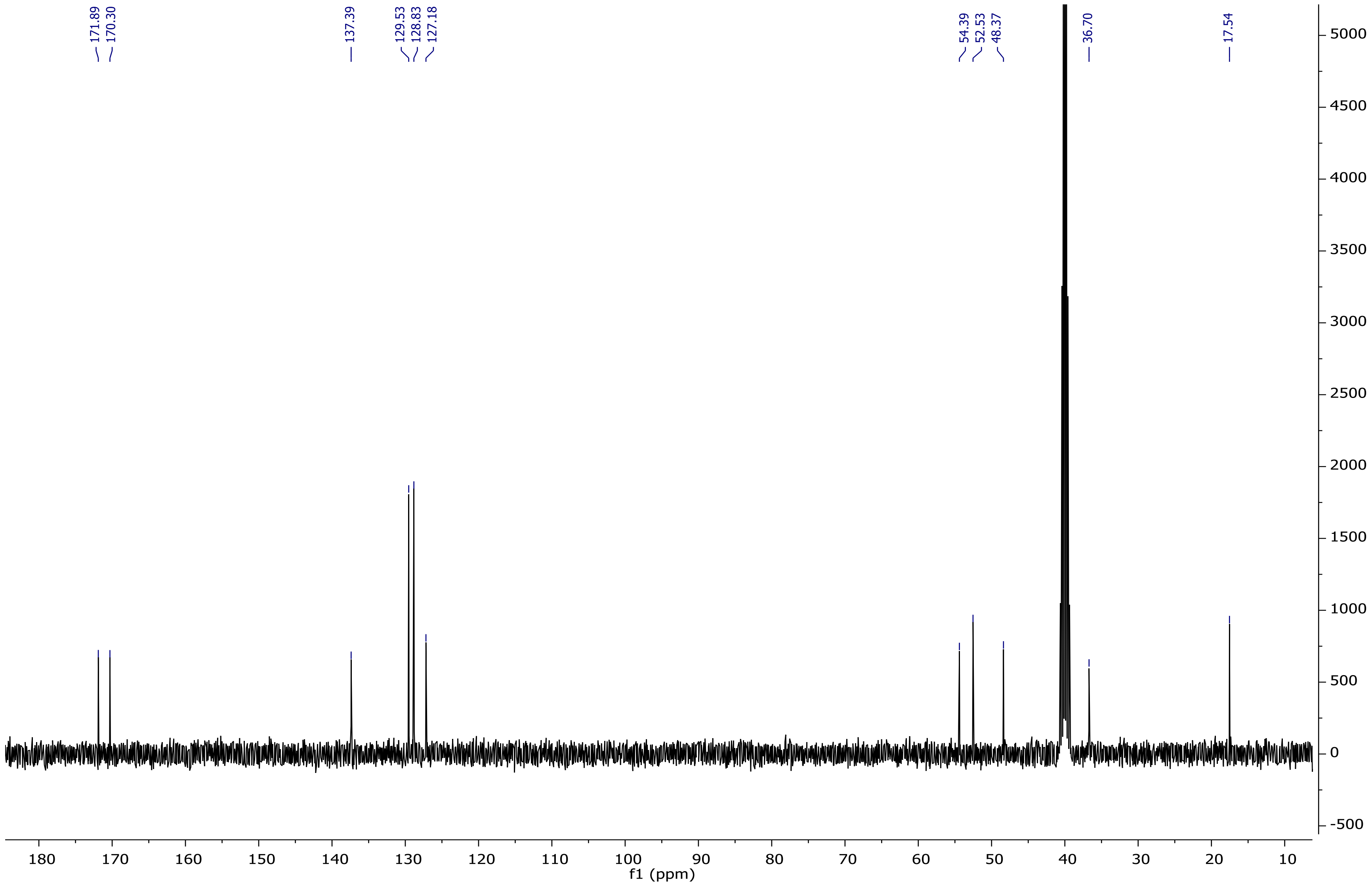
171.89
170.30
137.39
129.53
128.83
127.18
54.39
52.53
48.37
36.70
17.54
5000
4500
4000
3500
3000
2500
2000
1500
1000
500
0
-500
180 170 160 150 140 130 120 110 100 90 80 70 60 50 40 30 20 10
f1 (ppm)

H-A(TEMPO)AF-OMe reductive cleavage to Ser (60)

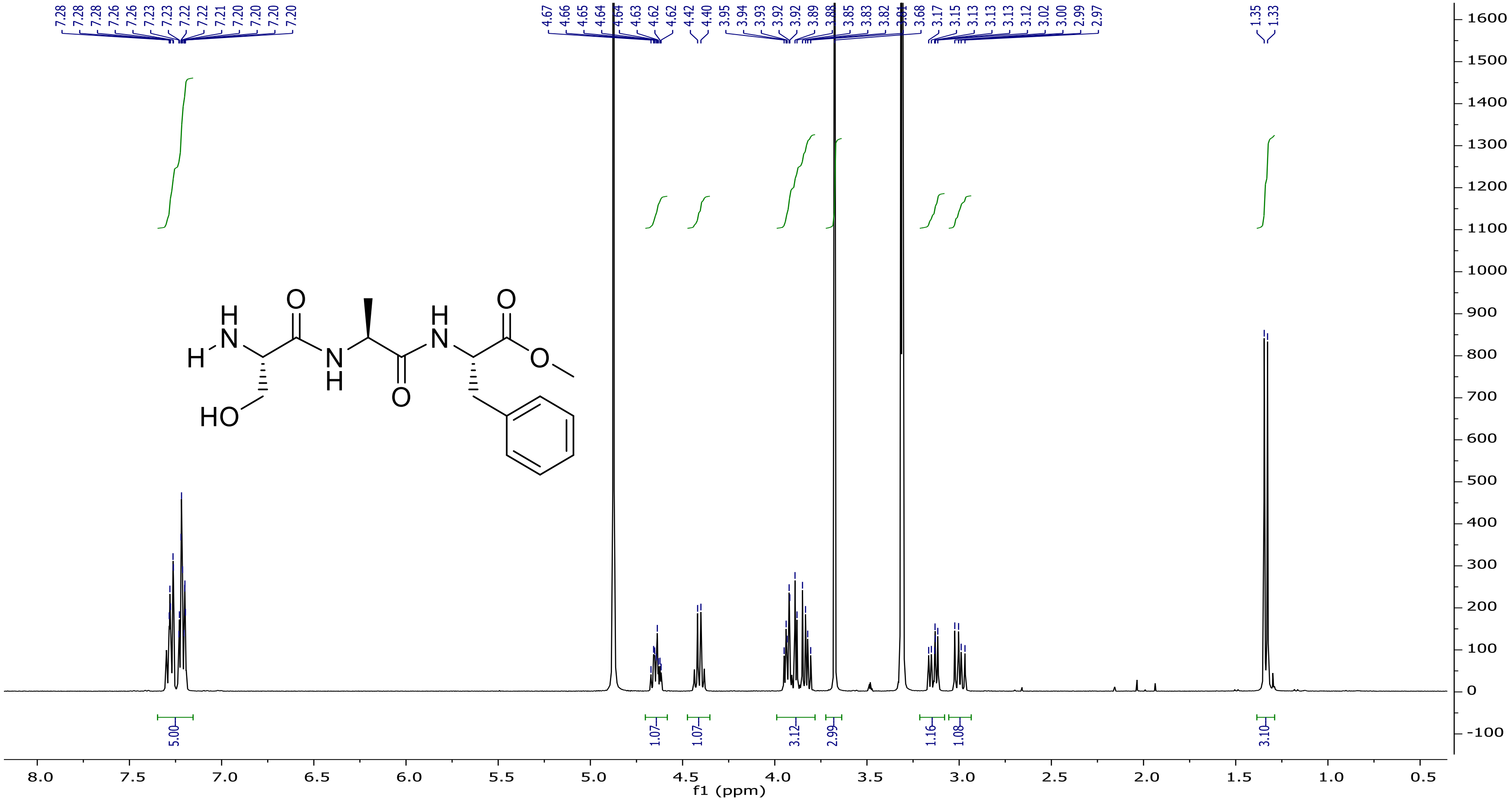

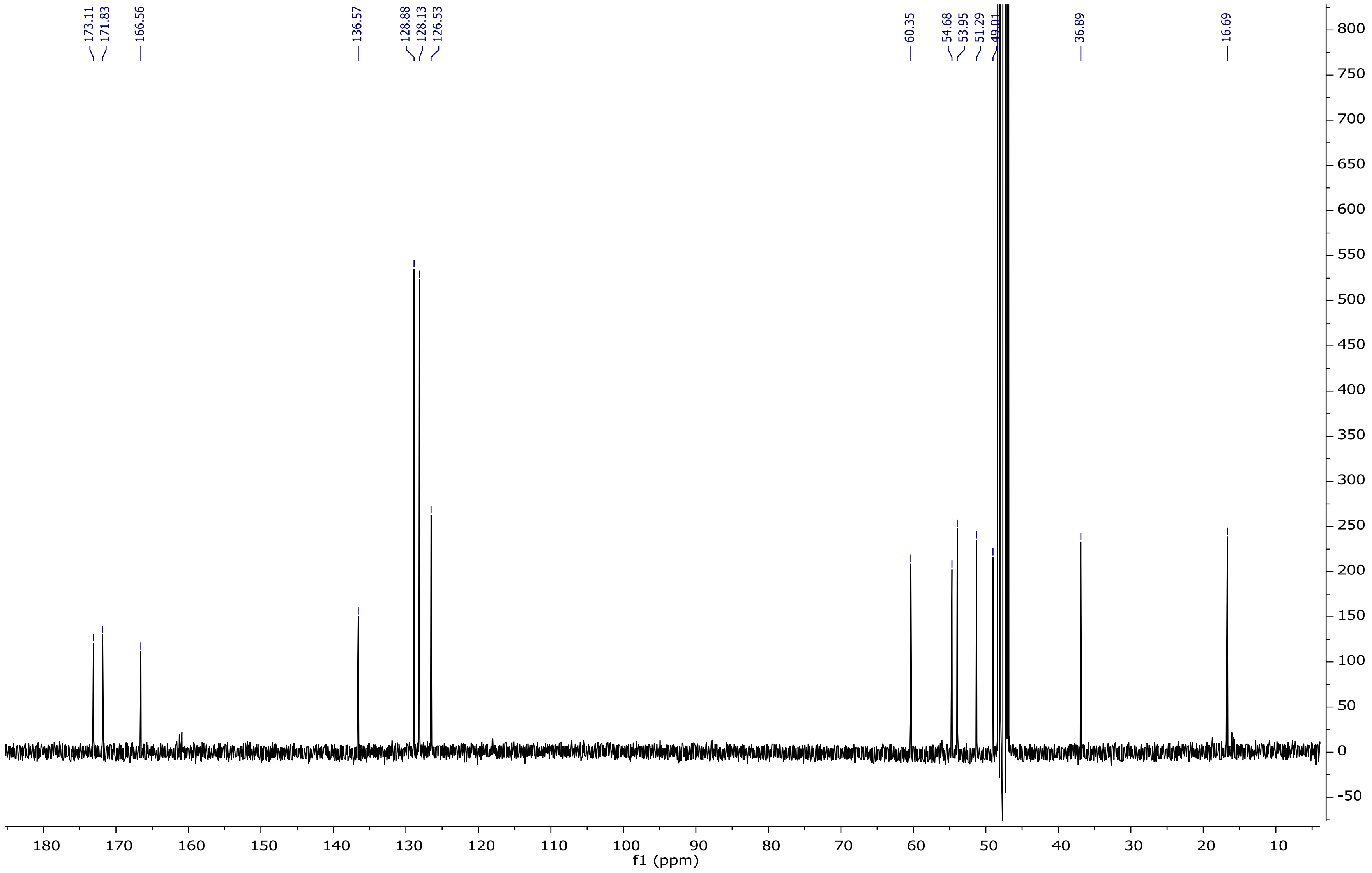
173.11
171.83
166.56
136.57
128.88
128.13
126.53
60.35
54.68
53.95
51.29
49.01
36.89
16.69
f1 (ppm)
